# A Sulfonated Tweezer-Shaped Receptor Selectively Recognizes Caffeine in Water

**DOI:** 10.1021/acs.joc.1c02620

**Published:** 2022-02-02

**Authors:** Oscar Francesconi, Andrea Ienco, Francesco Papi, Marta Dolce, Andrea Catastini, Cristina Nativi, Stefano Roelens

**Affiliations:** ‡ Department of Chemistry “Ugo Schiff” DICUS and INSTM, Polo Scientifico e Tecnologico, University of Florence, I−50019 Firenze, Italy; † Istituto di Chimica dei Composti Organometallici, Consiglio Nazionale delle Ricerche (CNR), Via Madonna del Piano, I-50019 Firenze, Italy

## Abstract

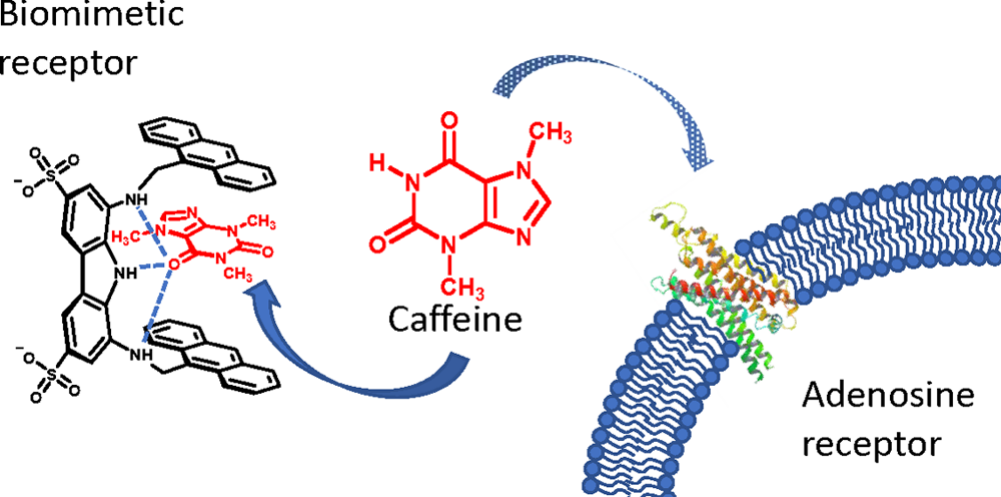

The selective recognition of caffeine in water among structurally related xanthines and purine or pyrimidine bases was achieved by a simple tweezer-shaped receptor featuring sulfonate hydrosolubilizing groups. The remarkable affinity for caffeine, among the highest reported thus far in the literature and larger than that shown by adenosine receptors of all subtypes, stems from a synergistic combination of hydrogen bonding, CH−π, and π-stacking interactions.

## Introduction

The effective molecular recognition of biologically relevant targets by biomimetic receptors through noncovalent interactions in physiological media represents a main challenge for supramolecular chemists due to the strong competition from water molecules.^1^ Purine bases are among the most studied biological guests because of their pervasive occurrence as constituents of nucleotides and their key role in many metabolic and signaling processes.^2^ Among purine alkaloids, caffeine is the most widely consumed psychostimulant drug in the world and, in addition to its central stimulant effects, exerts various beneficial pharmacological activities as a competitive inhibitor of adenosine receptors.^3^ Caffeine also plays multiple roles as a drug for its antibronchospastic properties and is used as an analgesic adjuvant for pain treatment.^4^ Other attractive effects of caffeine have been observed in the prevention of neurodegenerative diseases and cancer immunotherapy.^5^ The use of artificial receptors that effectively recognize caffeine in water can therefore find a wide range of applications in biomedical, technological, and analytical fields.^6,7^

**Figure 1:**
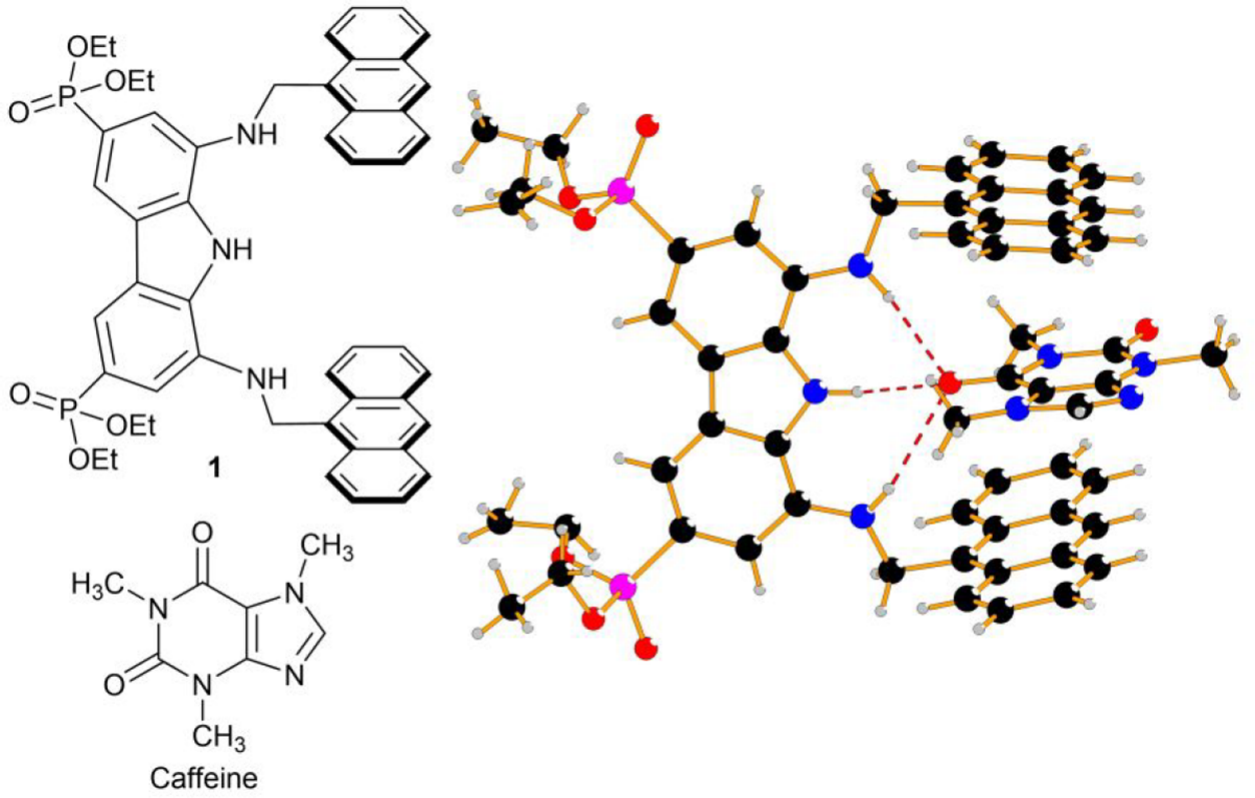
(Left) Structures of the liposoluble receptor **1** and caffeine. (Right) X-ray structure of the **1**·caffeine complex crystallized from chloroform (hydrogen bonds are depicted as dashed lines).

In a recent paper we reported that the diaminocarbazole tweezer-shaped receptor **1** (Figure 1, left) recognizes caffeine in chloroform with a 26 μM affinity, showing a sixfold selectivity versus theobromine and a nearly fivefold selectivity versus theophylline, the natural-occurring metabolites of caffeine.^8^ The X-ray structure of the complex between **1** and caffeine (Figure 1, right) shows that the binding ability of the receptor mainly relies on the hydrogen-bonding interaction established between the tridentate diaminocarbazole unit of the receptor^9^ and the O-6 of caffeine, which is reinforced by CH−π interactions between the methyl groups of the xanthine and the two anthracene units of the receptor as well as π-stacking between caffeine and the anthracene rings. Despite the excellent binding properties of receptor **1** in organic media,^10^ at present the receptor cannot be leveraged in an aqueous or physiological environment where most useful applications concerning caffeine can be envisaged, from biomedical devices to analytical applications.

## Results and Discussion

**Scheme 1:**
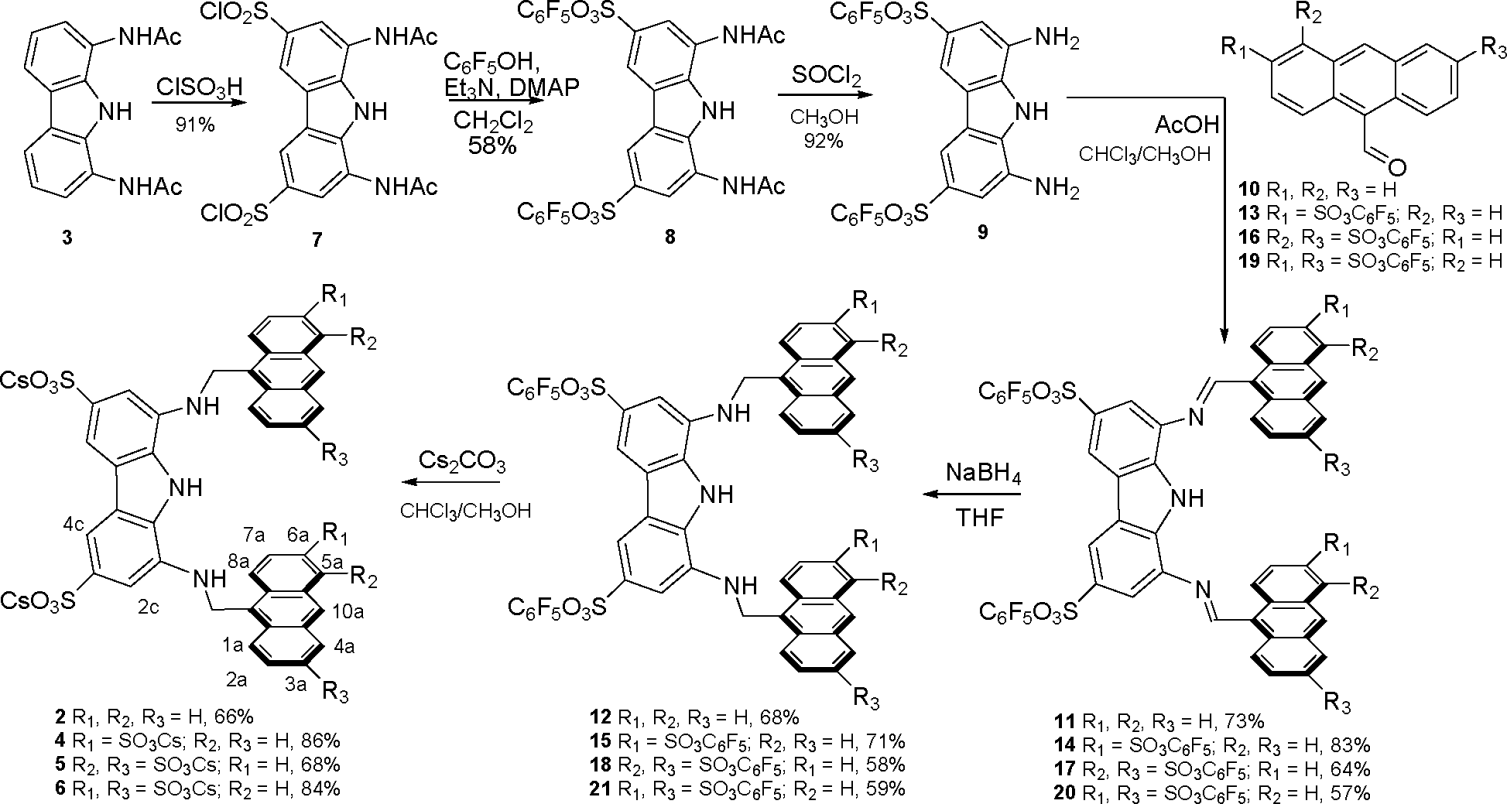
Synthesis of Receptors **2**, **4**, **5**, and **6** with Proton Labeling

To achieve caffeine recognition in aqueous media, we developed the water-soluble analogue of receptor **1** (**2**, Scheme 1) featuring sulfonate groups on the diaminocarbazole unit. Sulfonates are convenient hydrosolubilizing groups because they fully dissociate in a wide range of pH and protrude outward the binding cleft into the bulk water. Receptor **2** was easily prepared in six steps with a 16% overall yield from 1,8- diacetamidocarbazole **3** (Scheme 1). The new compound was obtained as the cesium salt that was freely soluble in water, giving a neutral solution of the receptor.

**Figure 2:**
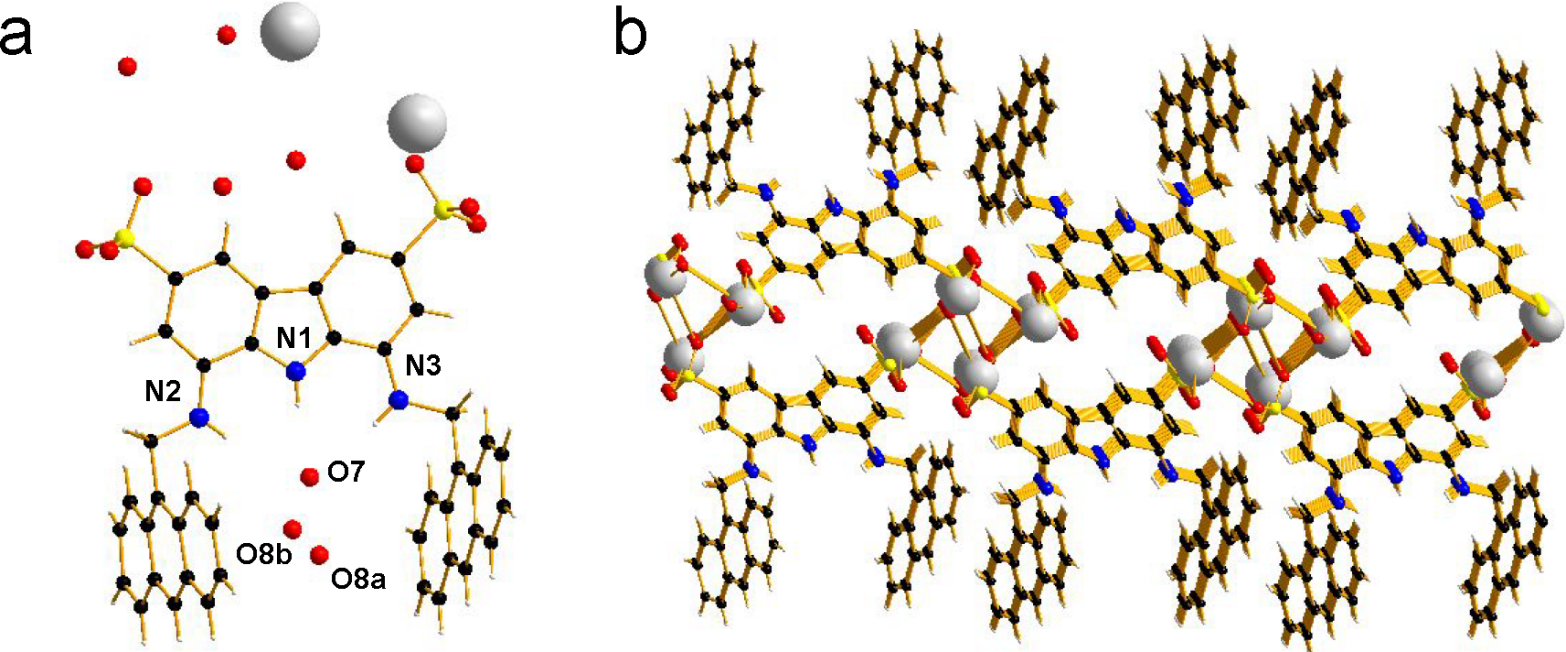
X-ray structure of receptor **2** crystallized from water showing (a) the asymmetric unit with cocrystallized water molecules and (b) the crystal packing.

The ^1^H NMR spectrum of receptor **2** in water showed broad signals above 0.4 mM, with marked variations of the chemical shifts depending on the concentration, suggesting the occurrence of self-association phenomena. Dilution experiments fitted a self-association model in which dimeric and tetrameric species were prevalent (log β_dim_ = 4.68 ± 0.12 and log β_tetram_ = 12.1 ± 0.3). X-ray analysis of the crystals obtained by the slow evaporation of a solution of **2** in water (Figure 2) showed a crystal packing dominated by electrostatic forces in which carbazole moieties were grafted on opposite sides of a cesium sulfonate layer, with anthracene residues pointing outward in a π-stacking disposition that left the binding cleft unoccupied. Interestingly, one of the cocrystallized water molecules found within the receptor cleft is hydrogen-bonded to the tridentate diaminocarbazole unit of the receptor in a way that is reminiscent of that observed between the O-6 of caffeine and the lipophilic receptor **1**.

**Figure 3:**
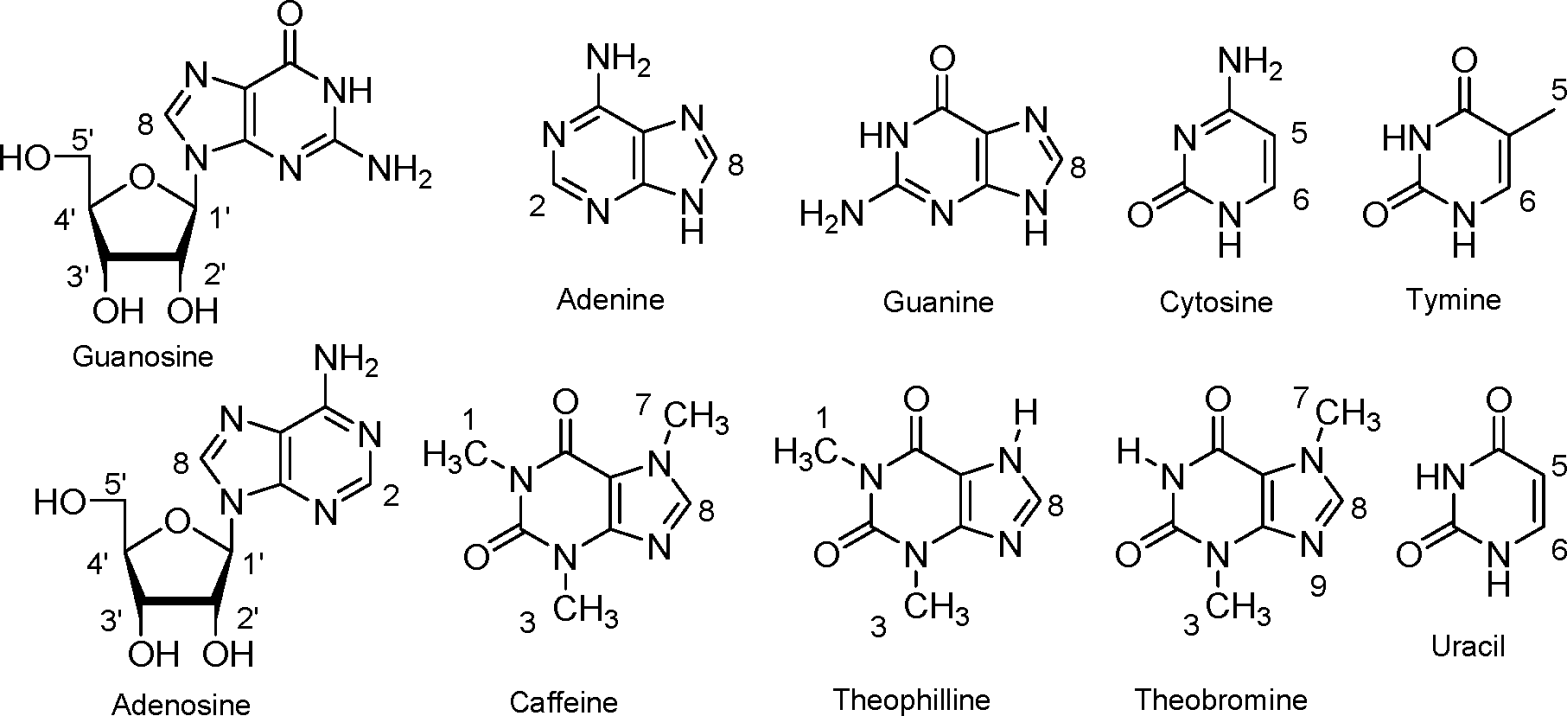
Chemical structure of the investigated ligands with proton labeling.

A quantitative investigation of the binding properties of receptor **2** was then carried out by ^1^H NMR titrations in water toward a set of purine and pyrimidine bases, including the xanthines caffeine, theophylline, and theobromine, together with adenine, cytosine, thymine, and uracil (Figure 3). Because of the poor solubility of guanine, the nucleoside guanosine was used instead. Correspondingly, adenosine was used in binding studies in addition to adenine for comparison. Nonlinear regression analysis of binding data gave the cumulative association constants reported in Table 1. Due to the strong self-association of receptor **2**, complexes with a stoichiometry higher than 1:1 were dominant to such an extent that the 1:1 association constant of theobromine became undetectable. In addition, dilution studies carried out on the three xanthines in water showed weak dimerization for caffeine (log β_dim_ = 0.78 ± 0.01), theophylline (log β_dim_ = 0.81 ± 0.02), and theobromine (log β_dim_ = 0.75 ± 0.33), which were set as invariant in the binding data analysis.

**Table 1:** Cumulative Formation Constants (log β_n_)^a^ and Intrinsic Median Binding Concentrations (BC_50_^0^, μM)^b^ for Receptor to Guest (R:G) Complexes of **2** and **6** with Purine and Pyrimidine Bases^c^

receptor		**2** (NMR)		**2** (ITC)		**6** (NMR)		**6** (ITC)	
ligands	R:G	log β	BC_50_^0^	log β	BC_50_^0^	log β	BC_50_^0^	log β	BC_50_^0^
caffeine	1:1	4.74 ± 0.09	4.2 ± 1.1	5.35 ± 0.21	5.8 ± 3.3	4.94 ± 0.03	11.5 ± 0.8	4.82 ± 0.06	15.0 ± 2.0
	2:1	10.9 ± 0.2		9.64 ± 0.31					
	1:2					7.10 ± 0.04			
theophylline	1:1	4.04 ± 0.07	26.0 ± 5.4	4.96 ± 0.23	15.7 ± 8.3	4.48 ± 0.03	32.8 ± 0.3	4.60 ± 0.03	25.2 ± 1.8
	2:1	9.59 ± 0.12		9.45 ± 0.25					
	4:1	19.1 ± 0.2							
	1:2					6.44 ± 0.05			
theobromine	1:1	n.d.^d^	35.6 ± 7.1	n.d.^d^	77.3 ± 18.1	4.67 ± 0.01	21.3 ± 0.5	4.67 ± 0.06	21.1 ± 2.8
	2:1	9.03 ± 0.06		9.26 ± 0.06					
	4:1	19.2 ± 0.1							
	1:2					6.47 ± 0.10			
adenine	2:1	8.23 ± 0.11	499 ± 143						
	4:1	16.5 ± 0.2							
adenosine	2:1	8.30 ± 0.04	548 ± 147						
guanosine	2:1	8.34 ± 0.01	524 ± 135						
thymine	2:1	7.80 ± 0.10	1403 ± 499						
	4:1	15.8 ± 0.3							
cytosine		n.d.^d^							
uracil		n.d.^d^							

a Formation constants were obtained by nonlinear least-squares regression analysis of NMR and ITC data.

b Calculated from the log β values using the “BC50 Calculator” program.^11^

c Measured at 298 K from NMR data in D_2_O at pD 7.4 and from ITC data in H_2_O at pH 7.4. Dimerization constants (**2**, log β_dim_ = 4.68 ± 0.13; caffeine, log β_dim_ = 0.78 ± 0.01; theophylline, log β_dim_ = 0.81 ± 0.02; and theobromine, log β_dim_ = 0.75 ± 0.33) and the receptor **2** tetramerization constant (log β_tetra_ = 12.1 ± 0.3) were set as invariant in the nonlinear regression analysis of NMR and isothermal titration calorimetry (ITC) data.

d Not detectable.

Because of the occurrence of multiple binding constants, overall affinities were assessed through the BC_50_^0^ (intrinsic median binding concentration) parameter,^11^ which was calculated from the measured constants and reported in Table 1. When only the 1:1 association is present, the BC_50_^0^ parameter coincides with the thermodynamic *K*_d_. Remarkably, receptor **2** showed a 4 μM affinity for caffeine, which is among the highest values reported thus far in the literature, exceeding those shown by human adenosine receptors of all subtypes (ranging between 9.6 and 13.3 μM).^12^ It is noteworthy that receptor **2** showed an improved affinity for caffeine in water with respect to the liposoluble receptor **1** in chloroform despite the competitive contribution from water for polar interactions. Selectivity versus its metabolites was also improved, as theophylline and theobromine were bound with sixfold and more than eightfold lower affinities, respectively.

Receptor **2** was markedly selective in the recognition of caffeine over other purine and pyrimidine bases. Indeed, all purines were bound with affinities more than two orders of magnitude smaller as both bases and nucleosides, suggesting that the glycosidic residue was not involved in binding. Concerning pyrimidines, thymine was poorly bound, whereas no variation of the chemical shift could be detected for cytosine and uracil.

UV–vis and fluorescence spectrophotometric techniques could not be used to measure reliable affinities because of a poor change in absorbance upon complexation and the internal quench of fluorescence between the two fluorophores that occurred upon addition of caffeine, respectively (Figures S56–S58).

Reliable binding constants could instead be obtained by isothermal titration calorimetry (ITC) as an independent technique. The generally good agreement between ITC and NMR results reported in Table 1 supported the binding affinity values measured by NMR spectroscopy. The discrepancy between the association models obtained from the two techniques is due to the intrinsically lower sensitivity of the ITC technique to the presence of multiple equilibria, which blurs the deconvolution of the binding isotherm with respect to the NMR technique. Furthermore, the presence of self-association equilibria involving both the receptor and the xanthines prevented an accurate determination of thermodynamic parameters.

To avoid the self-association phenomena that affected receptor **2**, a set of analogous structures sulfonated on the anthracene units were synthesized following the idea that additional hydrosolubilizing groups may prevent the clustering of the aromatic moieties. The tetrasulfonate **4** (Scheme 1), functionalized in position 3a of the anthracene rings, and the hexasulfonates **5** and 6, functionalized in positions 3a and 5a and 3a and 6a, respectively, were obtained by the same synthetic pathway used for receptor **2** using the corresponding anthracenecarbaldehydes in the condensation reaction with the diaminocarbazole unit (Scheme S1). While a dimerization constant (log β_dim_ = 2.04 ± 0.04) was still measurable for the tetrasulfonate receptor **4**, self-association phenomena could not be revealed for the hexasulfonate receptors **5** and **6**, which showed sharp invariant NMR signals in a wide range of concentration.

Binding measurements carried out by ^1^H NMR titrations of caffeine with receptors **5** and **6** to give simplified association models devoid of multinuclear species in the receptor. However, the symmetrically substituted **6** gave larger affinities than **5** and was therefore selected for the investigation. From the results reported in Table 1 it can be appreciated that with the slight increase in the 1:1 binding constant for receptor **6** with respect to that of **2**, a decrease in the overall affinity occurs, which can be ascribed to the lack of contribution from the 2:1 complex for the hexasulfonate receptor; however, strong binding is achieved by the 1:1 species alone. The comparable affinities (BC_50_^0^) of **2** and **6** for caffeine support the conclusion that the self-association of receptor **2** does not significantly affect recognition, as anticipated from the X-ray structure of the receptor. Selectivity for caffeine versus other xanthines was still observed, although it was shallower than that of **2**, as theophylline and theobromine were bound with three- and twofold lower affinities, respectively.

Binding affinities measured by NMR were confirmed by ITC measurements, which gave results in good agreement with the spectroscopic technique. In the absence of self-association equilibria, reliable thermodynamic parameters could also be obtained (Table 2), showing that recognition was enthalpically driven for xanthines with an adverse entropic contribution, suggesting that hydrogen bonding and CH−π interactions, rather than solvophobic effects, were the driving forces for recognition.

**Table 2:** Thermodynamic Parameters (kJ mol^–1^) for the Formation of the 1:1 Complexes between Receptor **6** and Caffeine, Theophyllline, and Theobromine in H_2_O at 298 K

	–Δ*G*°	–Δ*H*°	*T*Δ*S*°
caffeine	27.5 ± 0.3	47.2 ± 0.9	–19.7 ± 1.2
theophylline	26.2 ± 0.2	44.9 ± 0.6	–18.7 ± 0.7
theobromine	26.7 ± 0.3	42.2 ± 1.0	–15.5 ± 1.3

**Figure 4:**
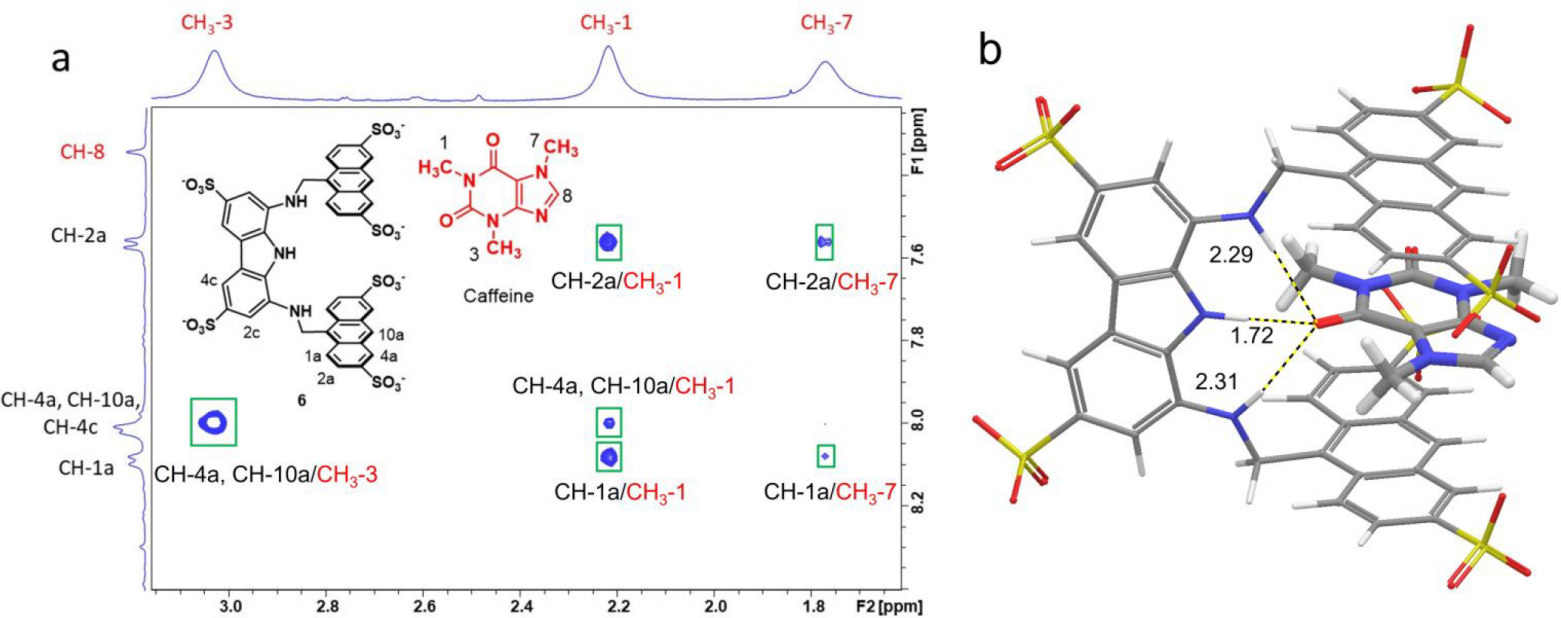
(a) The 500 MHz NOESY spectrum of an equimolar mixture of **6** and caffeine (10 mM each) in D_2_O at 298 K. Intermolecular NOE cross peaks are highlighted by squares. (b) Global minimum energy structure of the **6**·caffeine complex obtained from a conformational search (OPLS_2005 force field, implicit water, Monte Carlo method, and 1000 steps). Intermolecular hydrogen-bonding interactions found in the calculated structure, with corresponding oxygen and hydrogen distances (Å), are indicated as dashed lines.

Unfortunately, our efforts to obtain crystals of the caffeine·**6** complex suitable for X-ray analysis failed. However, a 3D picture of the binding mode in solution could be obtained by combining NMR spectroscopic data with molecular modeling calculations using a well-established protocol.^13^ The chemical shift differences of the caffeine methyl group signals between the free and the bound state showed that CH_3_-1 and CH_3_-7 were strongly upfield-shifted (Δδ = 1.41 and 1.54 ppm, respectively; see Table S1) while the shielding effect of anthracene on CH_3_-3 was less pronounced (Δδ = 0.34 ppm), suggesting that, in contrast to CH_3_-3, CH_3_-1 and CH_3_-7 were located inside the cleft between the two anthracene rings. NOESY spectra (Figure 4a), which were recorded out on equimolar mixture of **6** and caffeine, supported this geometry, showing the NOE contacts of CH_3_-1 and CH_3_-7 of caffeine with the CH-2a and CH-1a of anthracene and of those of CH_3_-3 with the CH-4a and CH-10a protons. The minimum-energy structure obtained from a conformational search of the caffeine·**6** complex (Figure 4b), apart from an evident π-stacking between the aromatic moieties of caffeine and anthracene, agrees with the proximities inferred by NOE contacts (Figure S65), showing the caffeine O-6 oxygen hydrogen-bonded to the tridentate diaminocarbazole unit in a geometry similar to that observed in the crystal structure of the complex with the lipophilic receptor **1** (see for reference Figures 1 and 4b). Interestingly, the presence of a water molecule bound to the basic N-9, as observed in the crystal structure of monohydrated caffeine,^14^ agrees with the X-ray structure of **2** and with the calculated model of **6**, showing that in both complexes the N-9 nitrogen prefers to be pointing out toward the bulk water rather than toward the binding site of the receptor. On the other hand, the interaction of the N-9 nitrogen with the proximal sulfonate groups does not seem to play a significant role because receptors **2** and **6** show comparable 1:1 logβ values, even though sulfonate groups on the anthracene moieties are missing in the latter receptor. Thus, hydrogen-bonding plays a pivotal role in the complex, whereas CH−π interactions between CH_3_-1 or CH_3_-7 and the anthracene rings, together with π-stacking interactions between the aromatics of caffeine and the receptor, reinforce the interaction and determine the selectivity among xanthines. The selective recognition of xanthines over purine bases can reasonably be ascribed to the lack of CH−π contributions for the latter, whereas the decreased contribution from π–π interactions is a factor that additionally affects pyrimidines.

## Conclusion

In conclusion, in this Article we have shown that very effective recognition of caffeine can be achieved by the tweezer-shaped receptor **2** and its hexasulfonate analogue **6**. The affinities measured for caffeine, the natural antagonist of human adenosine receptors, challenge those of the biological target. Affinities and selectivities were assessed by NMR and calorimetric techniques, while NOE values and molecular modeling calculations provided a description of the complex binding mode whereby hydrogen-bonding, CH−π, and π-stacking interactions play central roles in governing affinities and selectivities. The results represent a significant step forward in the molecular recognition of caffeine, encouraging further developments and applications.

## Supporting Information Available

General methods, materials, synthetic procedures, NMR spectra, data tables, result pages, plots of NMR and ITC titrations, plots of the UV–vis and fluorescence experiments, molecular modeling methods, and the crystallographic table and details (PDF)

## Accession Codes

CCDC 2106332 contains the supplementary crystallographic data for this paper. These data can be obtained free of charge via www.ccdc.cam.ac.uk/data_request/cif, or by emailing data_request@ccdc.cam.ac.uk, or by contacting The Cambridge Crystallographic Data Centre, 12 Union Road, Cambridge CB2 1EZ, UK; fax: +44 1223 336033.
