# Supplementary material for: A Sulfonated Tweezer-Shaped Receptor Selectively Recognizes Caffeine in Water
Source: J Org Chem. 2022 Feb 2;87(5):2662–7. doi: 10.1021/acs.joc.1c02620 (PMC8902752; doi:10.1021/acs.joc.1c02620)
Supplement: Supplementary file 1 — jo1c02620_si_001.pdf [file jo1c02620_si_001.pdf]

A sulphonated tweezers-shaped receptor selectively recognizes caffeine in water.

Oscar Francesconi,[*,‡] *Andrea Ienco,*[†] *Francesco Papi*[‡] *Marta Dolce,*[‡] *Andrea Catastini,*[‡] *Cristina Nativi*[‡] *and Stefano Roelens*[*,‡]

[‡] Department of Chemistry "Ugo Schiff" DICUS and INSTM, University of Florence, Polo Scientifico e Tecnologico, I–50019 Sesto Fiorentino, Firenze, (Italy).

[†] Istituto di Chimica dei Composti Organometallici, CNR, Via Madonna del Piano, I-50019 Sesto Fiorentino, Firenze, (Italy)

oscar.francesconi@unifi.it

stefano.roelens@unifi.it

SUPPORTING INFORMATION

Synthesis and characterization of chemical materials **p. S2**
- **General** **p. S2**
- **Materials** **p. S2**
- **NMR Spectra** **p. S16**

Binding studies **p. S52**
- **NMR titrations and data analysis** **p. S52**
- **Ultraviolet/visible (UV/Vis) experiments** **p. S121**
- **Fluorescence experiments** **p. S123**
- **Calorimetric titrations and data analysis** **p. S124**

Structural studies **p. S156**
- **Molecular modeling methods** **p. S156**

Crystallographic data **p. S157**

References **p. S159**

Synthesis and characterization of chemical materials.

General. ESI-MS analyses were performed in negative and positive ion mode and were recorded on an LCQ-Fleet Ion Trap equipped with a standard Ionspray interface. ^{1}H NMR spectra were obtained at 400 MHz and 500 MHz. Chemical shifts are reported in part per million (*δ*) using the residual solvent line as internal reference (7.26 ppm, 2.50 ppm and 2.05 ppm for spectra run in $CDCl_3$, $DMSO_{d6}$, $Acetone_{d6}$ respectively). ^{13}C NMR spectra were obtained at 125 MHz and 100 MHz in $CDCl_3$ and $DMSO_{d6}$. Chemical shifts are reported in *δ* using the central solvent line as internal reference at 77.16 ppm for $CDCl_3$, 39.52 ppm for $DMSO_{d6}$. Proton and carbon assignments were carried out by means of selective 1D-NOESY spectra and 2D NMR spectra, *i.e.* COSY and HSQC.

Materials. Reagents were purchased from commercial suppliers and used without purification. 9-anthracenecarbaldehyde **10**, adenosine, guanosine and all purine and pyrimidine bases used in binding studies were commercial samples. 1,8-diacetamido-9*H*-carbazole **3** was prepared according to known methods.[S1]

1,8-diacetamido-9H-carbazole-3,6-disulphonyldichloride (7).

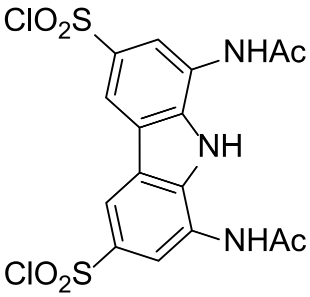

At 10 mL of freshly distilled $ClSO_3H$ cooled at 0 °C, **3** (3.03 g, 10.8 mmol) was gradually added. The mixture was heated at 60 °C with an oil bath for 2 h, then poured into icy water. The formed precipitate was filtered, washed with water and dried to give **7** (4.70 g, 9.83 mmol, 91%) as a grey powder that was used in the next step without further purification. ^{1}H NMR (DMSO$_{d6}$, 500 MHz): δ 10.84 (bs, 1H), 10.07 (bs, 2H), 8.08 (s, 2H), 7.98 (s, 2H), 2.21 (s, 6H).

Bis(pentafluorophenyl)-1,8-diacetamido-9H-carbazole-3,6-disulphonate (8).

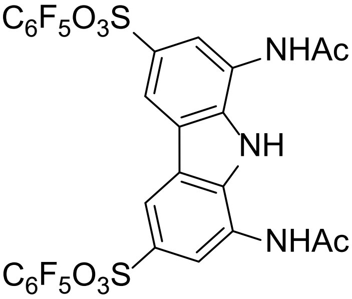

Under a nitrogen atmosphere, at a solution of pentafluorophenol (3.70, 20.0 mmol) in CH_2Cl_2 (50 mL), were added Et_3N (2.91 g, 28.8 mmol) and DMAP (242 mg, 1.98 mmol). After 10 min stirring, **7** (4.59 g, 9.59 mmol) was added and the mixture stirred for further 1.5 h at room temperature. The mixture was diluted with CH_2Cl_2 (150 mL) and washed with HCl 1M. The organic layer was dried over Na_2SO_4, filtered and the solvent evaporated. The crude was purified by filtration on silica gel (MeOH 10% in $CHCl_3$) to give pure **8** (4.27 g, 5.52 mmol, 58%) as a pink powder. Mp: > 245 °C (dec). ^{1}H NMR (DMSO$_{d6}$, 500 MHz): δ 11.83 (s, 1H), 10.44 (s, 2H), 9.01 (d, J = 0.72 Hz, 2H), 8.40 (s, 2H), 2.27 (s, 6H). ^{13}C{^{1}H} NMR (DMSO$_{d6}$, 125 MHz): δ 169.4, 141.8 (d, J = 251.44 Hz), 139.9 (d, J = 248.08 Hz), 137.6 (d, J = 252.28 Hz), 136.1, 124.8, 124.46, 123.7-123.5 (m), 123.6, 120.0, 118.4, 23.8. ESI-MS m/z: $[M+Na]^+$ 796.08 (100%).

Bis(pentafluorophenyl)-1,8-diamino-9H-carbazole-3,6-disulphonate (9).

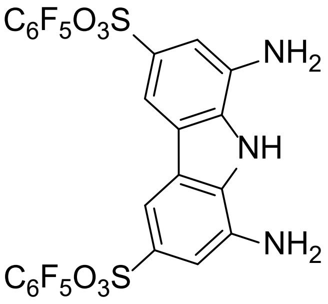

To a suspension of **8** (3.74 g, 4.83 mmol) in CH_3OH (30 mL), $SOCl_2$ (1.56 g, 13.1 mmol) was added dropwise. The mixture was heated to reflux with an oil bath for 1.5 h, then cooled at room temperature and poured into a saturated solution of $NaHCO_3$ (300 mL). The precipitate was filtered and washed with water, then dried under vacuum to give **9** (3.05 g, 4.42 mmol, 92%) of a pink solid. Mp: >200 °C (dec); ^{1}H NMR ($DMSO_{d6}$, 500 MHz): δ 11.86 (s, 1H), 8.22 (d, J = 0.8 Hz, 2H), 7.29 (d, J = 0.9 Hz, 2H), 5.83 (s, 4H). $^{13}C\{^1H\}$ NMR ($DMSO_{d6}$, 125 MHz): δ 141.9 (d, J = 253.57 Hz), 139.7 (d, J = 252.71 Hz), 137.5 (d, J = 249.29 Hz), 135.6, 132.8, 124.6, 124.0-123.8 (m), 122.7, 112.3, 107.4. ESI-MS m/z: $[M-H]^-$ 688.25 (100%).

Compound (11).

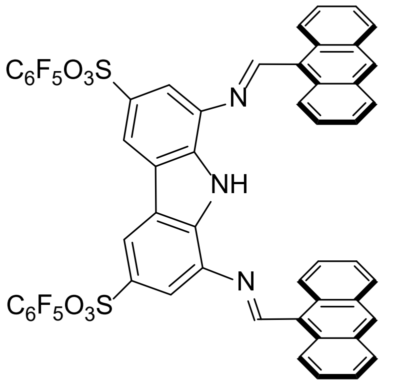

To a suspension of **9** (914 mg, 1.33 mmol) in a 1:1 mixture of $CHCl_3/CH_3OH$ (20 mL), **10** (558 mg, 2.85 mmol) and acetic acid (262 mg, 4.37 mmol) were added. The mixture was heated to reflux with an oil bath for three days adding each day acetic acid (262 mg, 4.37 mmol). The suspension was then filtered, washed with CH_2Cl_2 and dried to give **11** (1.03 g, 0.966 mmol, 73%) as an orange powder that was used for the next step without further purifications. ^{1}H NMR ($DMSO_{d6}$, 500 MHz): δ 12.92 (bs, 1H), 9.94 (s, 2H), 9.28 (d, J = 1.61 Hz, 2H), 8.82-8.80 (m, 4H), 8.77 (s, 2H), 8.13-8.11 (m, 4H), 8.06 (d, J = 1.61 Hz, 2H), 7.57-7.51 (m, 8H).

Compound (12).

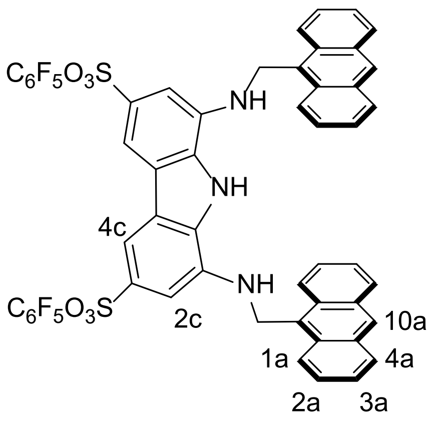

To a suspension of **11** (1.025 g, 0.962 mmol) in THF (15 mL), $NaBH_4$ (297 mg, 7.85 mmol) was added. The mixture was stirred at room temperature for 8 h, then poured into a saturated solution of NH_4Cl (60 mL). The aqueous layer was extracted with ethyl acetate and the combined organic layers dried over Na_2SO_4, filtered and evaporated to give a yellow powder that was washed with CH_3OH to yield pure **12** (697 mg, 0.651 mmol, 68%) as a white powder. Mp: >171 °C (dec); ^{1}H NMR (CD_3OD 3% in $CDCl_3$, 500 MHz): δ 8.33 (s, 2H, C*H*-10a), 8.13 (d, *J* = 1.48 Hz, 2H, C*H*-4c), 8.08-8.06 (m, 4H, C*H*-1a), 7.90-7.88 (m, 4H, C*H*-4a), 7.45 (d, *J* = 1.48 Hz, 2H, C*H*-2c); 7.40-7.34 (m, 8H, C*H*-2a, C*H*-3a), 5.16 (s, 4H, C*H*2NH). ^{13}C{^{1}H} NMR (CD_3OD 3% in $CDCl_3$, 125 MHz): δ 142.6 (d, *J* = 253.80 Hz), 140.3 (d, *J* = 265.37 Hz), 138.0 (d, *J* = 253.18 Hz), 135.3, 132.2, 131.30 130.3, 129.0 (*C*H-4a), 128.1 (*C*H-10a), 126.8, 126.8 (*C*H-2a), 125.3 (*C*H-3a), 124.8-124.7 (m), 123.7 (*C*H-1a), 122.5, 113.0 (*C*H-4c), 103.4 (*C*H-2c), 50.3, 40.1 (*C*H_2-NH). ESI-MS m/z: $[M-H]^-$ 1068.11 (100%).

Receptor (2).

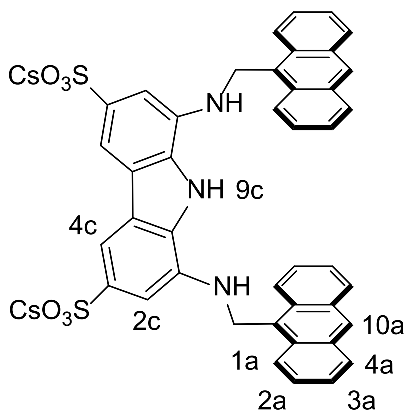

To a suspension of **12** (601 mg, 0.562 mmol) in a mixture of 15% CH_3OH in $CHCl_3$ (20 mL), Cs_2CO_3 (1.50 g, 4.59 mmol) was added. The suspension was stirred and heated to reflux with an oil bath for 4 h then filtered. The solid was washed with a mixture of 15% CH_3OH in $CHCl_3$, then with CH_3OH, and dried to give a yellow solid. Receptor **2** was purified suspending 672 mg of the crude in

50 mL of CH_3OH at reflux and gradually adding 13 mL of water. The suspension was cooled, filtered, washed with CH_3OH and then with a mixture of 20% H_2O in CH_3CN (50 mL) to give pure **2** (255 mg, 0.255 mmol, 66%) as a yellow powder. Mp: >230 °C (dec); ^{1}H NMR (DMSO$_{d6}$, 500 MHz): δ 10.23 (bs, 1H, N*H*-9c), 8.53 (s, 2H, C*H*-10a), 8.22 (m, *J* = 8.58 Hz, 4H, C*H*-1a), 8.02 (m, *J* = 8.21 Hz, 4H, C*H*-4a), 7.77 (d, *J* = 0.91 Hz, 2H, C*H*-4c), 7.49-7.43 (m, 8H, C*H*-15, C*H*-3a), 7.33 (d, *J* = 0.71 Hz, 2H, C*H*-2c), 5.17 (d, *J* = 4.58 Hz, 4H, C*H*$_2$-NH), 5.01 (t, *J* = 4.58 Hz, 2H, N*H*-CH$_2$). ^{13}C{^{1}H} NMR (DMSO$_{d6}$, 125 MHz): δ 140.8, 133.7, 130.9, 130.1, 129.4, 128.7 (*C*H-4a), 128.2, 127.4 (*C*H-10a), 126.4 (*C*H-2a), 125.2 (*C*H-3a), 124.2 (*C*H-1a), 121.8, 107.0 (*C*H-4c), 103.3 (*C*H-2c), 40.2 (*C*H$_2$-NH); ESI-MS m/z: $[M]^{-2}$ 367.67 (100%). HRMS m/z: $[M-H]^-$ Calcd. for $C_{42}H_{30}N_3O_6S_2$, 736.1582; Found 736.1568.

Compound (14).

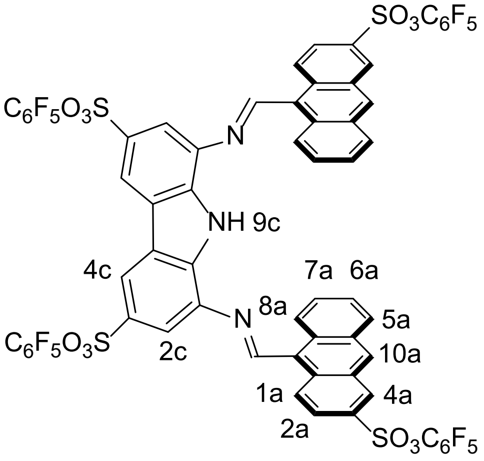

To a suspension of **9** (500 mg, 0.725 mmol) in 7 mL of $CHCl_3$, **13** (724 mg, 1.6 mmol) and acetic acid (218 mg, 3.63 mmol) were added. The mixture was heated to reflux with an oil bath for three days, then the suspension was filtered, washed with $CHCl_3$ and dried to give **14** (933 mg, 0.599 mmol, 83%) as a yellow powder that was used for the next step without further purifications. ^{1}H-NMR (DMSO$_{d6}$/THF, δ = 2.50/3.60, 500 MHz): δ 12.92 (s, 1H, N*H*-9c), 9.99 (s, 2H, C*H*NH), 9.32 (s, 2H, C*H*-10a), 9.14 (s, 1H, C*H*-4c), 9.11 (d, *J* = 9.56 Hz, 2H, C*H*-1a), 8.96 (s, 2H, C*H*-4a), 8.80 (d, *J* = 8.88 Hz, 2H, C*H*-8a), 8.20 (d, *J* = 8.36 2H, C*H*-5a), 8.16 (s, 2H,C*H*-2c), 7.89 (d, *J* = 9.54 2H, C*H*-2a), 7.72-7.71 (m, 2H, C*H*-7a), 7.68-7.65 (m, 2H, C*H*-6a). ESI-MS m/z (%):1556.98 (100%) $[M-H]^-$.

Compound (15).

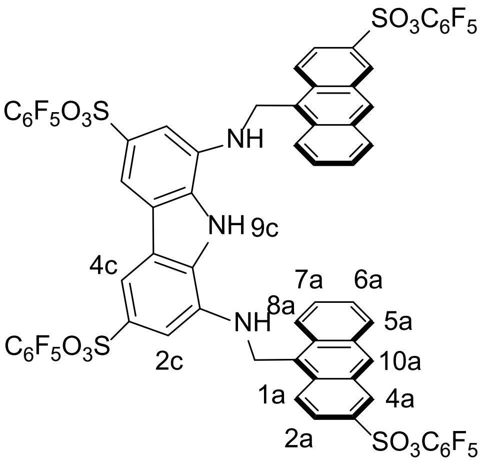

To a suspension of **14** (500 mg, 0.32 mmol) in THF (6.5 mL) cooled at 0 °C, $NaBH_4$ (50 mg, 1.29 mmol) was added. The mixture was stirred at 0 °C for 30 min, then poured into a saturated solution of NH_4Cl. The aqueous layer was extracted with ethyl acetate and the combined organic layers dried over Na_2SO_4, filtered, and evaporated to give a yellow powder that was purified by flash chromatography (MeOH 1% in $CHCl_3$) to give pure **15** (357 mg, 0.229 mmol, 71%) as a yellow powder. Mp: >268 °C (dec); ^{1}H NMR (DMSO$_{d6}$, 500 MHz): δ 11.30 (s, 1H, N*H*-9), 8.96 (s, 2H, C*H*-10a), 8.89 (d, *J* = 2.01 Hz, 2H, C*H*-4a), 8.57 (d, *J* = 1.47 Hz, 2H, C*H*-4c), 8.54 (d, *J* = 9.52 Hz, 2H, C*H*-1a), 8.30 (d, *J* = 9.01 Hz, 2H, C*H*-8a), 8.13 (d, *J* = 8.37 Hz, 2H, C*H*-5a), 7.82 (dd, *J* = 2.10 Hz, d, *J* = 9.76 Hz, 2H, C*H*-2a), 7.66-7.64 (m, 2H, C*H*-7a), 7.62-7.59 (m, 2H, C*H*-6a), 7.48 (d, *J* = 1.40 Hz, 2H, C*H*-2c), 6.17 (t, *J* = 4.53 Hz, 2H, N*H*-CH_2), 5.26 (d, *J* = 4.24 Hz, 4H, C*H*$_2$-NH). $^{13}C\{^1H\}$ NMR (DMSO$_{d6}$, 125 MHz): δ 141.8 (dd, *J* = 252.51 Hz, *J* = 30.9 Hz), 139.9 (dd, *J* = 252.51 Hz, *J* = 19.8 Hz), 137.2 (dt, *J* = 252.51 Hz, *J* = 14.16 Hz), 136.0 (Cq), 133.4 (C-22), 132.7 (Cq), 132.4 (Cq), 131.8 (Cq), 131.5 (C-10a), 131.0 (Cq), 139.4 (Cq), 129.3 (C-5a), 128.7 (C-7a), 128.5 (Cq), 127.6 (C-1a), 126.7 (C-16), 125.3 (Cq), 124.6 (C-18), 124.0 (t), 123.4 (t), 122.1 (Cq), 120.8 (C-2a), 113.6 (C-4c), 103.8 (C-2c), 40.1 (CH_2-NH). ESI-MS m/z (%):1584.33 (100%) $[M+Na]^+$; 1600.17 (18%) $[M+K]^+$; 1560.58 (100%) $[M-H]^-$

Receptor (4).

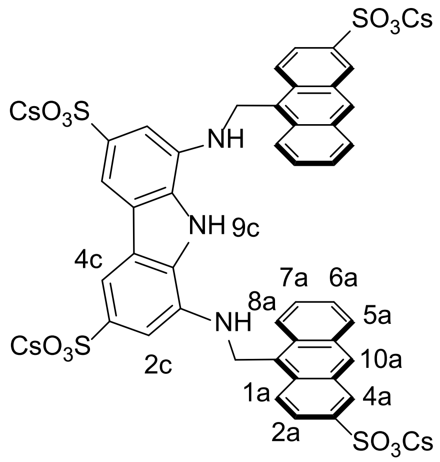

To a suspension of **15** (500 mg, 0.320 mmol) in a mixture of 15% CH_3OH in $CHCl_3$ (15 mL), Cs_2CO_3 (976 mg, 3.00 mmol) was added. The suspension was vigorously stirred and heated to reflux with an oil bath for two days then filtered. The solid was washed with a mixture of 15% CH_3OH in $CHCl_3$, then with CH_3OH, and dried to give pure **4** (393 mg, 0.276 mmol, 86%) as a brown solid. Mp: 312-318 °C; ^{1}H NMR (DMSO$_{d6}$, 500 MHz): δ 10.23 (s, 1H, N*H*-9c), 8.59 (s, 2H, C*H*-10a), 8.21 (s, 2H, C*H*-4a), 8.21-8.19 (m, 4H, C*H*-1a, C*H*-8a), 8.03 (d, *J* = 7.06 Hz, 2H, C*H*-5a), 7.75 (s, 2H, C*H*-4c), 7.64 (d, *J* = 8.63, 2H, C*H*-2a), 7.49-7.46 (m, 4H, C*H*-7a, CH-6a), 7.30 (s, 2H, C*H*-2c), 5.16 (s, 4H, C*H*$_2$-NH), 5.08 (s, 2H, N*H*-CH$_2$). ^{13}C{^{1}H} NMR (DMSO$_{d6}$, 125 MHz): δ 144.3 (Cq), 140.7 (Cq), 133.8 (Cq), 131.3 (Cq), 128.8 (C-5a), 128.3 (Cq), 128.3 (C-10a), 126.6 (C-7a), 125.3 (C-6a), 124.7 (C-2a), 124.5 (C-4a), 124.4 (C-8a), 124.2 (C-1a), 121.8 (Cq), 107.0 (C-4c), 103.3 (C-2c), 40.1 (CH_2-NH). ESI-MS m/z (%): 298.25 (100%) $[M-3H]^{3-}$, 447.83 (50%) $[M-2H]^{2-}$. HRMS m/z: $[M-H]^-$ Calcd. for $C_{42}H_{28}N_3O_{12}S4$, 298.0191; Found 298.0179.

Compound (17).

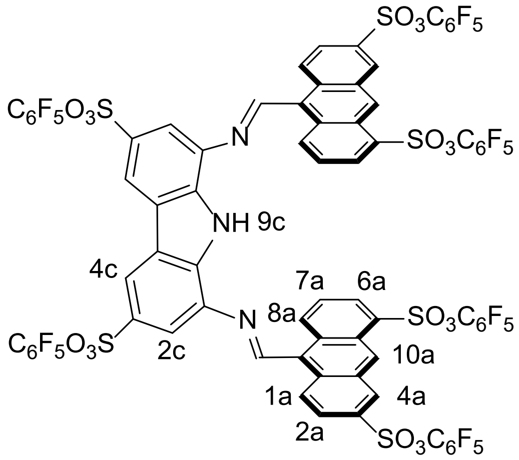

To a suspension of **9** (500 mg, 0.725 mmol) in 7 mL of $CHCl_3$, **16** (1.13 g, 1.62 mmol) and acetic acid (221 mg, 3.67 mmol) were added. The mixture was heated to reflux with an oil bath for 18 h, then the solution was washed with a saturated solution of $NaHCO_3$, dried over Na_2SO_4, and evaporated to give a crude that was purified by flash chromatography ($CHCl_3$) to give pure **17** (950 mg, 0.463 mmol, 64%) as an orange powder. Mp: 259-261 °C; ^{1}H NMR ($CDCl_3$, 500 MHz):δ 10.28 (s, 1H, N*H*-9c), 9.99 (s, 2H, C*H*-NH), 9.45 (s, 2H, C*H*-10a), 9.03 (d, *J* = 9.53 Hz, 2H, C*H*-8a), 8.95 (d, *J* = 8.62 Hz, 2H, C*H*-1a), 8.70 (s, 2H, C*H*-2c), 8.34-8.33 (m, 4H, CH-6a, CH-4a), 8.15 (s, 2H, CH-4c), 7.88 (d, *J* = 9.07 Hz, 2H, C*H*-2a), 7.77-7.74 (m, 2H, C*H*-7a). ESI-MS m/z (%):2048.87 (100%) $[M-H]^-$

Compound (18).

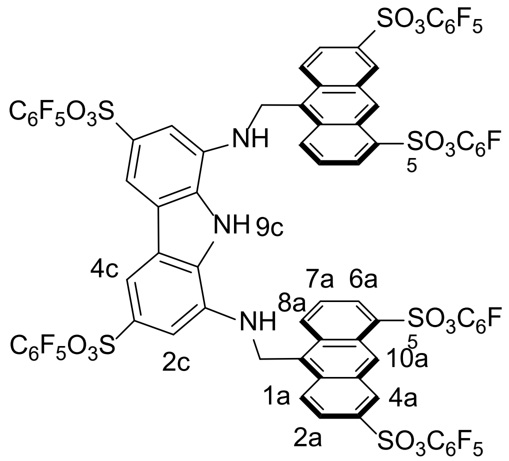

To a solution of **17** (510 mg, 0.25 mmol) in THF (5 mL) cooled at 0 °C, $NaBH_4$ (80 mg, 2.06 mmol) was added. The mixture was stirred at 0 °C for 5 min, then poured into a saturated solution of NH_4Cl. The aqueous layer was extracted with ethyl acetate and the combined organic layers dried over Na_2SO_4, filtered, and evaporated to give a yellow powder that was purified by flash chromatography (ethyl acetate 10% in $CHCl_3$) to give pure **18** (298 mg, 0.145 mmol, 58%) as a yellow powder. Mp: >273 °C (dec); ^{1}H-NMR **(**DMSO$_{d6}$, 500 MHz): δ 11.20 (s, 1H, N*H*-9c), 9.57 (s, 2H, C*H*-10a), 9.19 (s, 2H, C*H*-4a), 8.87 (d, *J* = 9.03 Hz, 2H, C*H*-8a), 8.66 (d, *J* = 9.45 Hz, 2H, C*H*-1a), 8.61 (s, 2H, C*H*-4c), 8.39 (d, *J* = 6.09 Hz, 2H, C*H*-6a), 8.00 (d, *J* = 8.65 Hz, 2H, C*H*-2a), 7.81-7.78 (m, 2H, C*H*-7a), 7.56 (s, 2H, C*H*-2c), 6.43 (t, 2H, N*H*-CH_2), 5.39 (d, 4H, C*H*$_2$-NH). ^{13}C{^{1}H} NMR (DMSO$_{d6}$, 125 MHz): δ 141.4 (dd, *J* = 252.71 Hz, *J* = 11.15 Hz), 141.7 (dt, *J* = 250.39 Hz, *J* = 14.74 Hz), 141.6 (dd, *J* = 257.34 Hz, *J* = 14.74 Hz), 140.0 (d, *J* = 238.79 Hz), 137.5 (d, *J* = 250.39 Hz), 135.9 (Cq), 134.5 (C-8a), 133.9 (C-4a), 133.9 (C-6a), 133.1 (Cq), 132.7 (Cq), 132.5 (Cq), 130.2 (Cq), 129.9 (Cq), 128.0 (C-1a), 127.8 (C-10a), 126.1 (C-7a), 125.4 (Cq), 125.3 (Cq), 124.0 (t), 123.4 (t), 122.5 (C-2a), 122.2 (Cq), 114.0 (C-4c), 104.3 (C-2c), 40.3 (*C*H_2-NH). ESI-MS *m/z* (%):2076.21 (100%) $[M+Na]^+$;2052.90 (100%) $[M-H]^-$

Receptor (5).

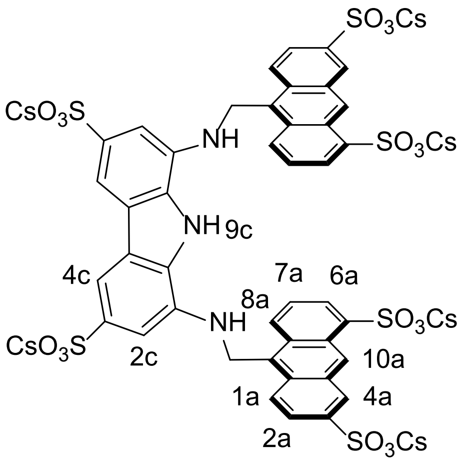

To a suspension of **18** (270 mg, 0.13 mmol) in a mixture of 15% CH_3OH in $CHCl_3$ (7 mL), Cs_2CO_3 (576 mg, 1.77 mmol) was added. The suspension was vigorously stirred and heated to reflux with an oil bath for two days then filtered. The solid was washed with a mixture of 15% CH_3OH in $CHCl_3$, then with CH_3OH, and dried to give pure **5** (164 mg, 0.089 mmol, 68 %) as a brown solid. Mp: 350-351 °C. ^{1}H NMR (DMSO$_{d6}$, 500 MHz): δ 10.20 (s, 1H, N*H*-9c), 9.46 (s, 2H, C*H*-10a), 8.20-8.16 (m, 6H, C*H*-6a, C*H*-1a, C*H*-4a), 7.90 (d, *J* = 6.84 Hz, 2H, CH-8a), 7.71 (d, *J* = 0.72 Hz, 2H, CH-4c), 7.66-7.63 (m, 2H, C*H*-2a), 7.40-7.38 (m, 2H, C*H*-7a), 7.27 (s, 2H, CH-2c), 5.15 (d, *J* = 4.05 Hz, 4H, C*H*$_2$-NH), 4.88 (t, 2H, N*H*-CH$_2$). ^{13}C{^{1}H} NMR (DMSO$_{d6}$, 125 MHz): δ 144.3 (Cq), 144.2 (Cq), 140.7 (Cq), 133.7 (Cq), 131.1 (Cq), 129.6 (Cq), 129.4 (Cq), 129.2 (Cq), 128.4 (C-10a), 128.2 (Cq), 127.1 (Cq), 126.0(C-6a), 125.1 (C-17, Cq), 125.1 (C-1a), 124.7 (C-2a), 124.0 (C-4a), 123.9 (C-8a), 121.7 (Cq), 106.9 (C-4c), 103.0 (C-2c), 39.5 (CH$_2$-NH). ESI-MS (positive ion mode) m/z (%): 1584.33 (100%) $[M+Na]^+$; 1600.17 (18%) $[M+K]^+$; ESI-MS (negative ion mode) m/z (%): 1560.58 (100%) $[M-H]^-$. HRMS m/z: $[M-H]^-$ Calcd. for $C_{42}H_{26}N_3O_{18}S_6$, 210.3913; Found 210.3909.

Compound (20).

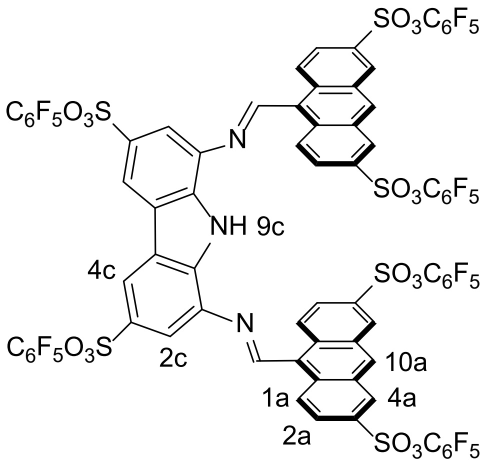

To a suspension of **9** (202 mg, 0.293 mmol) in 6 mL of $CHCl_3$, **19** (419 mg, 0.600 mmol) and trifluoroacetic acid (10 mg, 85 μmol) were added. The mixture was heated to reflux with an oil bath for 15 h, then the precipitate was filtered and washed with $CHCl_3$ to give pure **20** (343 mg, 0.167 mmol, 57%) as a yellow powder. ^{1}H-NMR ($CDCl_3$, 500 MHz): δ 12.94 (s, 1H, N*H*-9c), 10.00 (s, 2H, C*H*-NH), 9.39 (s, 2H, C*H*-10a), 9.31 (s, 2H, C*H*-4c), 9.09 (d, *J* = 8.62 Hz, 4H, C*H*-1a), 8.99 (s, 4H, C*H*-4a), 8.23 (s, 2H, C*H*-2c), 8.04 (d, *J* = 9.07 Hz, 4H, C*H*-2a).

Compound (21).

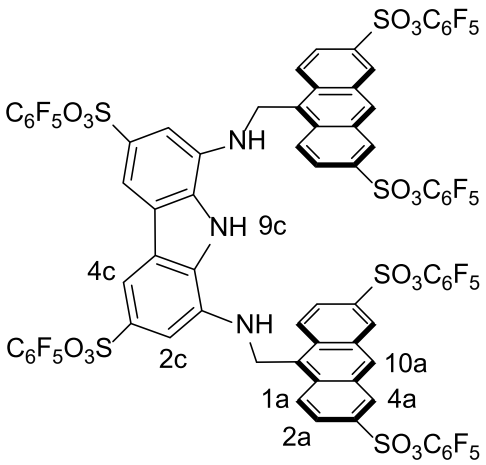

To a solution of **20** (322 mg, 0.157 mmol) in THF (20 mL), $NaBH_4$ (13 mg, 0.344 mmol) was added. The mixture was stirred for 1 min then leaved unstirred at room temperature for 2 h. The suspension was filtered and washed with THF. The mother liquor was concentrated, redissolved in ethyl acetate and washed with water. The organic layers were dried over Na_2SO_4 and evaporated to give a crude that was purified by flash chromatography (ethyl acetate 5% in $CHCl_3$) to give pure **20** (189 mg, 0.0920 mmol, 59%) as a yellow powder. Mp: 286-287 °C. ^{1}H NMR(DMSO$_{d6}$, 500 MHz): δ 11.25 (s, 1H, N*H*-9c), 9.27 (s, 2H, C*H*-10a), 8.96 (d, *J* = 9.03 Hz, 4H, C*H*-4a), 8.63 (d, *J* = 9.45 Hz,

4H, C*H*-1a), 8.59 (s, 2H, C*H*-4c), 7.99 (d, *J* = 6.09 Hz, 2H, C*H*-2a), 7.55 (s, 2H, C*H*-2c), 6.30 (s, 2H, N*H*-CH_2), 5.33 (s, 4H, C*H*$_2$-NH). $^{13}C\{^1H\}$ NMR (125 MHz, $DMSO_{d6}$): δ 141.6 (d, J = 249.86 Hz), 140.0 (d, J = 243.37 Hz), 137.6 (d, J = 249.86 Hz), 136.6 (Cq), 135.0 (C-10), 133.6 (Cq), 133.6 (Cq), 133.5 (2Cq), 132.5 (C-4a), 131.7 (Cq), 130.9 (2Cq), 129.5 (2Cq), 128.2 (C-1a, Cq), 125.4 (Cq), 123.3 (t), 123.3 (C-2a), 122.2 (Cq), 114.0 (C-4c), 104.3 (C-2c), 59.8 (CH_2-NH).

Receptor (6).

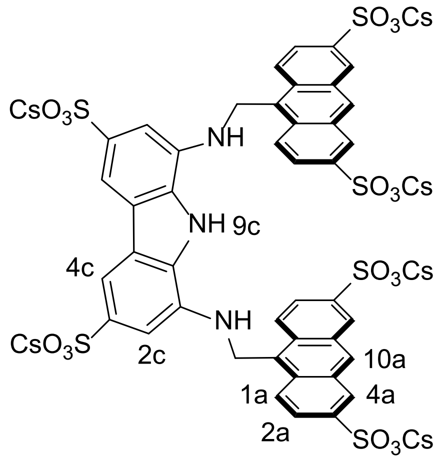

To a suspension of **21** (100 mg, 48.7 μmol) in a mixture of 15% CH_3OH in $CHCl_3$ (2.5 mL), Cs_2CO_3 (115 mg, 0.35 mmol) was added. The suspension was vigorously stirred and heated to reflux with an oil bath for two days then filtered. The solid was washed with a mixture of 15% CH_3OH in $CHCl_3$, then with CH_3OH, and dried to give pure **6** (76 mg, 41.1 μmol, 84 %) as a brown solid. Mp: 344 °C (dec). ^1H NMR ($DMSO_{d6}$, 500 MHz): δ 10.19 (s, 1H, N*H*-9c), 8.67 (s, 2H, C*H*-10), 8.24 (s, 4H, C*H*-4a), 8.18 (d, *J* = 6.84 Hz, 4H, C*H*-1a), 7.76 (s, 2H, C*H*-4c), 7.66 (d, *J* = 4.05 Hz, 4H, C*H*-2a), 7.30 (s, 2H, C*H*-2c), 5.15 (s, 6H, C*H*$_2$-NH, CH_2-N*H*). $^{13}C\{^1H\}$ NMR ($DMSO_{d6}$, 125 MHz): δ 144.3 (Cq), 140.6 (Cq), 133.9 (Cq), 130.4 (Cq), 130.2 (Cq), 129.3 (Cq), 129.3 (C-10), 128.4 (C-2a), 124.8 (C-4a), 124.7 (C-1a), 124.3 (Cq), 121.8 (Cq), 107.0 (C-4c), 103.2 (C-2c), 40.3 (CH_2-NH). HRMS m/z: $[M-2H]^{2-}$ Calcd. for $C_{42}H_{25}N_3O_{18}Cs_4S_6$, 791.2855; Found 791.2826.

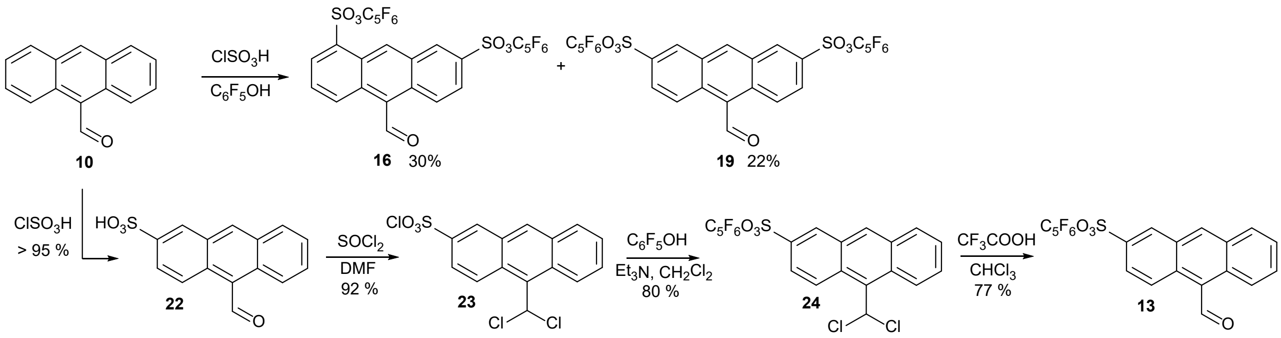

Scheme S1. Synthesis of the sulphonate anthracenecarboxaldehydes **13**, **16** and **19**.

Bis(pentafluorophenyl)-9-formylanthracene-3,5-disulphonate (16) and Bis(pentafluorophenyl)-9-formylanthracene-3,6-disulphonate (19)

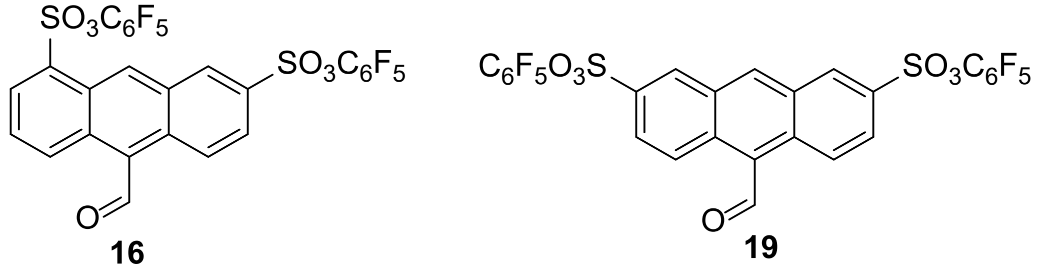

Solid 9-anthracenecarboxaldehyde **10** (1.00 g, 4.85 mmol) was slowly added to 5 mL of anhydrous $ClSO_3H$ cooled at 0 °C, then the mixture was heated to 60 °C with an oil bath. After 2 h, pentafluorophenol (2.68 g, 14.6 mmol) was added and reaction was kept at 60 °C for other 2 h. The mixture was then dropped in icy water and the precipitate formed was extracted with ethyl acetate. The combined organic layers were washed with a saturate solution of Na_2CO_3 and with water, then were dried over Na_2SO_4, filtered and evaporated to give 3.03 g of a crude that was purified by flash chromatography (ethyl acetate 30% in petroleum ether) to give pure **16** (1.03 g, 1.47 mmol, 30%) and pure **19** (739 mg, 1.06 mmol, 22%) as yellow powders. **16**: mp: 156-158 °C. ^{1}H NMR (DMSO$_{d6}$, 500 MHz): δ 11.47 (s, 2H, C*H*O), 9.88 (s, 1H, C*H*-10), 9.42-9.38 (m, 2H, C*H*-4,8), 9.19 (d, *J* = 10.24 1H, C*H*-1), 8.48 (d, *J* = 7.83, 1H, C*H*-6), 8.21 (d, *J* = 10.24, 1H, C*H*-2), 8.00 (d, *J* = 9.60, 1H, C*H*-7). ^{13}C{^{1}H} NMR (DMSO$_{d6}$, 125 MHz): δ 194.6 (*C*HO), 141.6 (dt, *J* = 252.40 Hz, *J* = 13.35 Hz), 140.1 (dt, *J* = 254.28 Hz, *J* = 14.46 Hz), 137.6 (dq, *J* = 248.63 Hz, *J* = 13.35 Hz), 134.0 (*C*-6,4), 133.2 (*C*-8), 133.0 (*C*-10), 132.7 (*C*-5’), 131.8 (*C*-1’), 131.4 (*C*-4’), 130.3 (*C*-8’), 129.9 (*C*-3), 128.6 (*C*-9), 128.2 (*C*-7), 127.0 (*C*-1), 125.2 (*C*-5), 124.8 (*C*-2), 123.4 (t). ESI-MS m/z (%): 698.96 (100%) $[M+H]^{+}$. **19**: mp: 156-158 °C. ^{1}H NMR (DMSO$_{d6}$, 500 MHz): *δ* 11.45 (s, 2H, C*H*O), 9.61 (s, 1H, C*H*-10), 9.29 (d, *J* = 8.71, 2H, C*H*-1,8), 9.11 (s, 2H, C*H*-4,5), 8.23 (dd, *J* = 9.88, *J* = 6.62, 2H, C*H*-2,7). ^{13}C{^{1}H} NMR (DMSO$_{d6}$, 125 MHz): δ 193.9 (*C*HO), 141.6 (dd, *J* = 251.4 Hz, *J* = 11.24 Hz), 141.0 (*C*-10), 140.1 (dt, *J* = 252.8 Hz, *J* = 16.06 Hz), 137.6 (dt, *J* = 251.4 Hz, *J* = 14.45 Hz), 133.8

(*C*-3,4), 131.3 (*C*-1'), 129.5 (*C*-4'), 127.1 (*C*-1), 126.8 (*C*-9), 125.4 (*C*-2), 123.3 (t). ESI-MS m/z (%): 698.96 (100%) $[M+H]^+$.

9-formylanthracene-3-sulphonic acid (22)

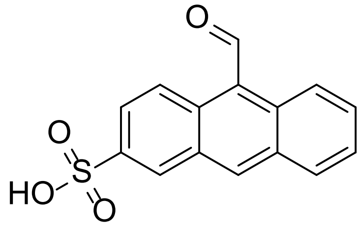

Solid 9-anthracenecarboxaldehyde **10** (4.15 g, 20.3 mmol) was slowly added to 40 mL of anhydrous $ClSO_3H$ cooled at 0 °C, then the mixture was stirred at room temperature for 2 h. The mixture was then dropped in icy water and the precipitate formed was extracted with ethyl acetate. The combined organic layers were dried under vacuum to give **22** (5.69 g, 20.0 mmol, >95%) that was used for the next step without further purifications. Mp: 113-115 °C. ^1H NMR **(**$DMSO_{d6}$, 400 MHz): δ 11.43 (s, 1H), 9.08 (s, 1H), 9.00 (s, 1H), 8.98 (s, 1H), 8.42 (d, *J* = 1.61 Hz, 1H), 8.19 (d, J = 8.58 Hz, 1H), 7.91 (dd, *J* = 9.31 Hz, *J* = 1.80 Hz, 1H), 7.74-7.70 (m, 1H), 7.63-7.59 (m, 1H). $^{13}C\{^1H\}$ NMR ($DMSO_{d6}$, 100 MHz): δ 194.5, 144.6, 136.4, 131.8, 131.2, 131.2, 130.1, 129.7 , 129.6, 127.4, 126.2, 125.3, 124.6, 123.7 (2C). ESI-MS m/z (%): 285.42 (100%) $[M-H]^-$.

9-(dichloromethyl)anthracene-3-sulphonyl chloride (23)

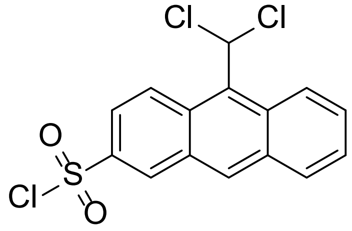

To a suspension of **22** (1.35 g, 4.75 mmol) in 14 mL of $SOCl_2$, a catalytic amount (4 drops) of anhydrous DMF was added. The mixture was heated with an oil bath for 2 h at 60 °C, then $SOCl_2$ excess was removed with a flux of nitrogen. The crude was dried under vacuum to give **23** (1.57 g, 4.37 mmol, 92%) that was used in the next steps without further purifications. ^1H NMR ($Acetone_{d6}$, 400 MHz): δ 9.26 (s, 1H, C*H*-10), 9.21 (bs, 1H), 9.09 (d, *J* = 2.08 Hz, 1H, C*H*-8), 8.84 (s, 1H, C*H*Cl2), 8.81 (bs, 1H), 8.34 (d, *J* = 8.48 Hz, 1H, C*H*-5), 8.19 (app. d, 1H, C*H*-4), 7.94-7.91 (m, 1H, C*H*-7), 7.80-7.76 (m, 1H, C*H*-6).

Pentafluorophenyl 9-(dichloromethyl)anthracene-3-sulphonate (24)

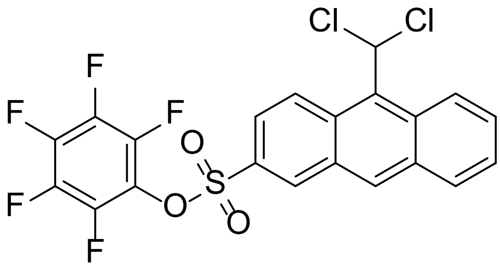

To a solution of 4-dimethylaminopyridine (300 mg, 2.41 mmol), pentafluorophenol (4.43 g, 24.06 mmol) and triethylamine (3.9 g, 8 mmol) in 100 mL of CH_2Cl_2, **23** (7.9 g, 21.88 mmol) was added. The mixture was stirred at room temperature for 2 h, then washed with HCl 1M, dried over Na_2SO_4 and evaporated to give **24** (8.91 g, 17.6 mmol, 80%). Mp: 186-189. ^{1}H NMR ($CDCl_3$, 500 MHz): δ 9.33 (bs, 1H), 8.75 (d, J = 2 Hz, 1H, C*H*-8), 8.72 (s, 1H, C*H*-10), 8.28 (bs, 1H), 8.25 (s, 1H, C*H*Cl2), 8.12 (d, J = 9.28, 1H, C*H*-4), 8.02 (d, J = 9.87 1H, C*H*-5), 7.79 (t, J = 8.14, J = 15.67, 1H, C*H*-2), 7.64 (t, J = 8.14, J = 15.67, 1H, C*H*-7). ^{13}C{^{1}H} NMR ($CDCl_3$, 125 MHz): δ 142.5 (dd, J = 255.93 Hz, J = 10.09 Hz), 140.5 (dt, J = 255.93 Hz, J = 13.87 Hz), 138.1 (dt, J = 249.69 Hz, J = 12.61 Hz), 134.1 (C-10), 133.5 (C-8), 131.3 (Cq), 130.3 (Cq), 130.1 (C-4), 129.5 (bs, C-2), 126.9 (C-7), 124.2 (t), 121.8 (C-5), 65.8 ($CHCl_2$). ESI-MS 471.42 (100%) $[M-Cl+H]^+$, 224.28 (100%) $[M+H-SO_3C_6F_5-Cl]^+$.

Pentafluorophenyl 9-formylanthracene-3-sulphonate (13)

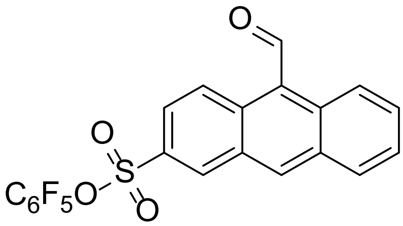

To a solution of **24** (1.3 g, 2.87 mmol) in 18 mL of $CHCl_3$, trifluoroacetic acid (3.28 g, 28.7 mmol) was slowly added. The reaction mixture was heated with an oil bath to 60°C overnight, then washed with water and the organic layers dried on Na_2SO_4 and evaporated to give a crude that was purified by flash chromatography (ethyl acetate 20% in petroleum ether) to give pure **13** (3.98 g, 2.21 mmol, 77%) as a yellow powder. Mp: 166-170 °C. ^{1}H NMR ($CDCl_3$, 500 MHz): δ 11.50 (s, 1H, CHO), 9.23 (d, J = 9.50 Hz, 1H, C*H*-1), 8.91 (d, J = 9.00 Hz, 1H, CH-8), 8.87 (s, 1H, C*H*-10), 8.75 (d, J = 2.00 Hz, 1H, C*H*-4), 8.15 (d, J = 8.50 Hz, 1H, C*H*-5), 8.02 (dd, J = 2.11 Hz, J = 9.55 Hz, 1H, C*H*-2), 7.85-7.81 (m, 1H, C*H*-7), 7.74-7.67 (m, 1H, C*H*-6). ^{13}C{^{1}H} NMR ($CDCl_3$, 125 MHz): δ 192.4 (CHO), 142.4 (dd, J = 259.38 Hz, J = 13.2 Hz), 140.5 (dt, J = 259.38 Hz, J = 13.99 Hz), 138.0 (dt, J = 253.74 Hz, J = 12.99 Hz), 137.6 (C-10), 134.4 (C-q), 132.9 (C-4), 133.0 (C-q), 131. 7 (C-q), 131.3 (C-7), 130.0 (C-5, C-q), 128.8 (C-q), 127.2 (C-6), 126.8 (C-1), 125.9 (C-q), 124.2 (C-2), 124.2 (t), 123.4 (C-8). ESI-MS m/z (%): 453.17 (100%) $[M+H]^+$.

NMR spectra.

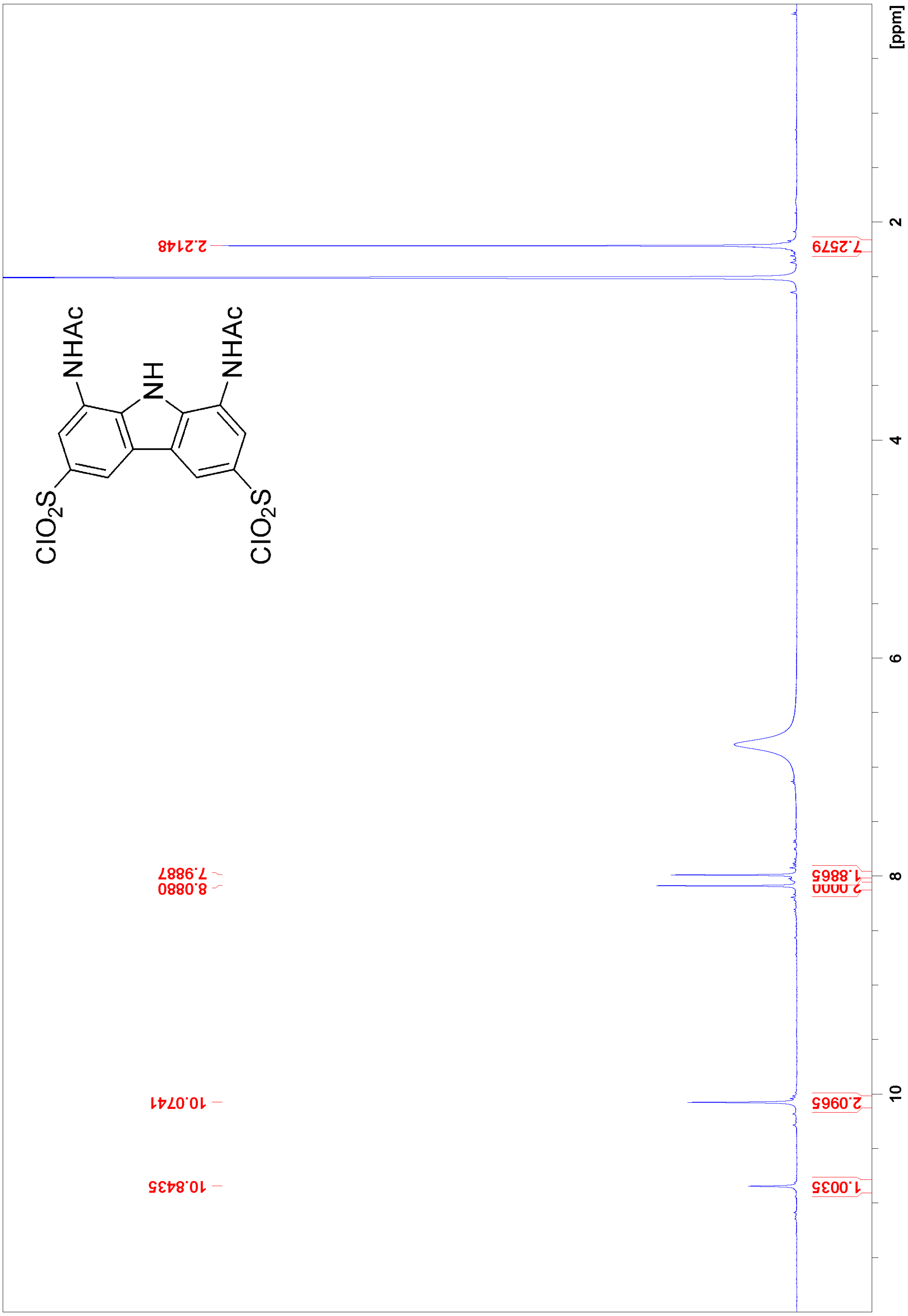

Figure S1. ^{1}H NMR spectrum of compound **7** (500 MHz, DMSO$_{d6}$).

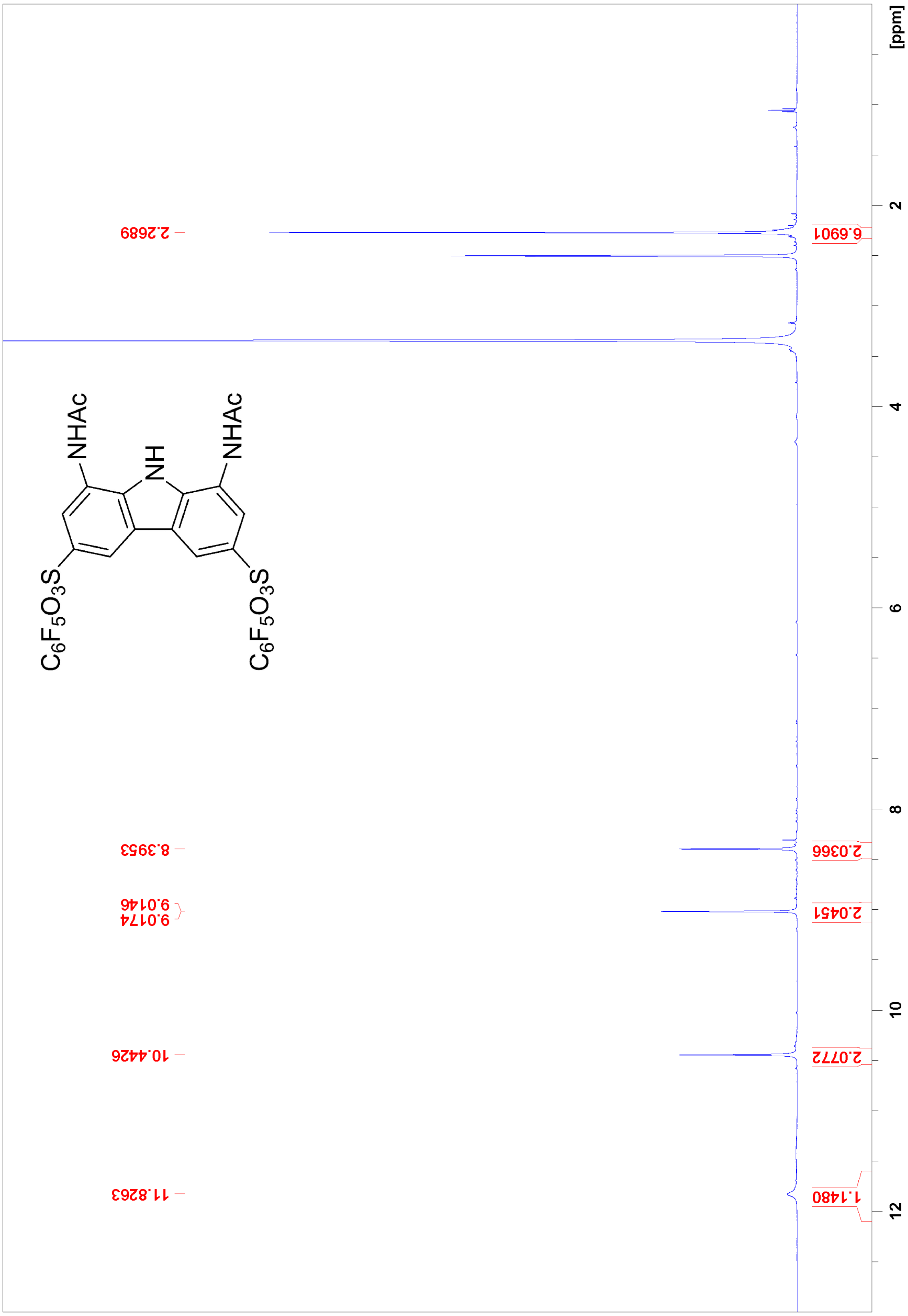

Figure S2. ^{1}H NMR spectrum of compound **8** (500 MHz, $DMSO_{d6}$).

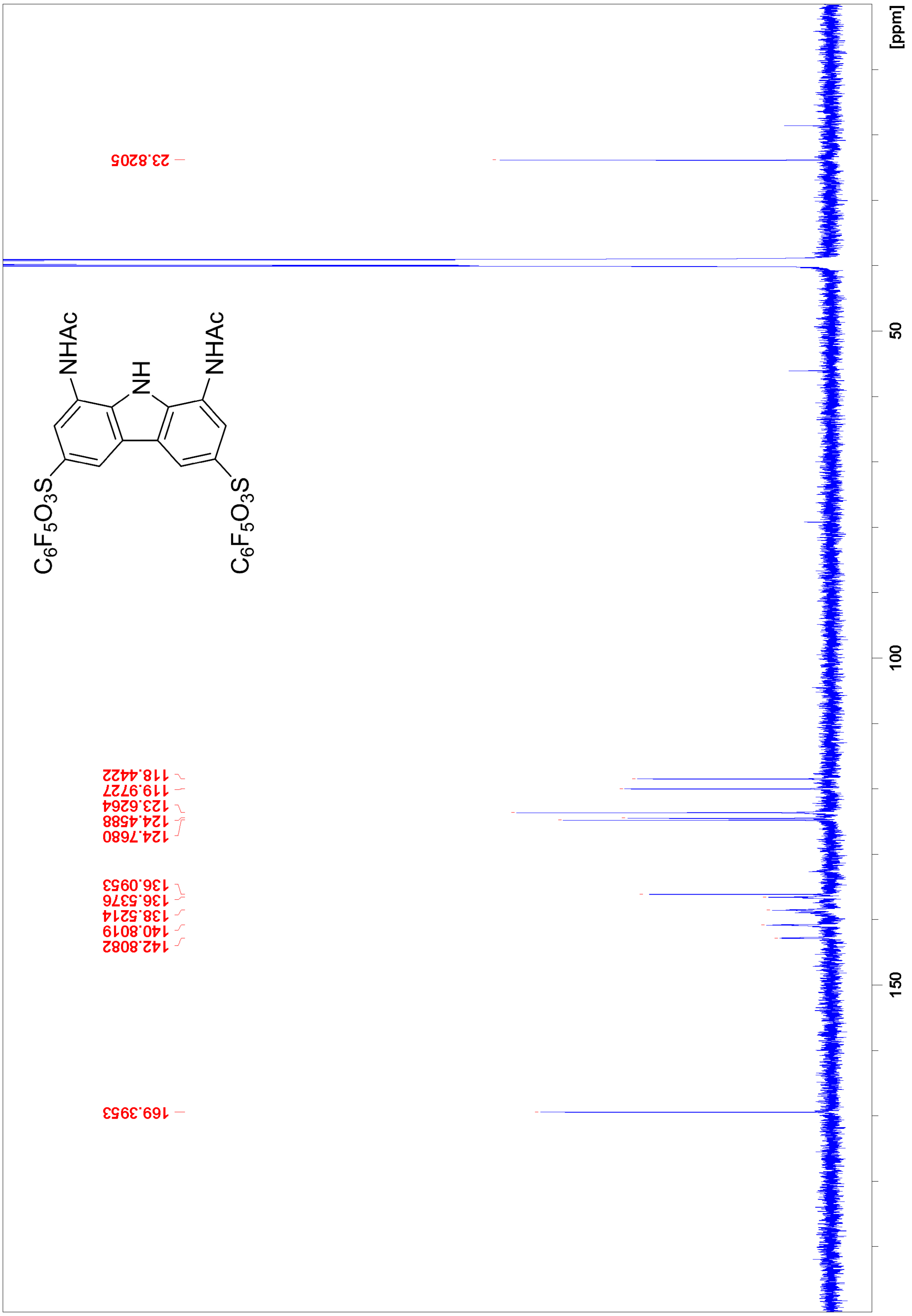

Figure S3. $^{13}C\{^1H\}$ NMR spectrum of compound **8** (125 MHz, $DMSO_{d6}$).

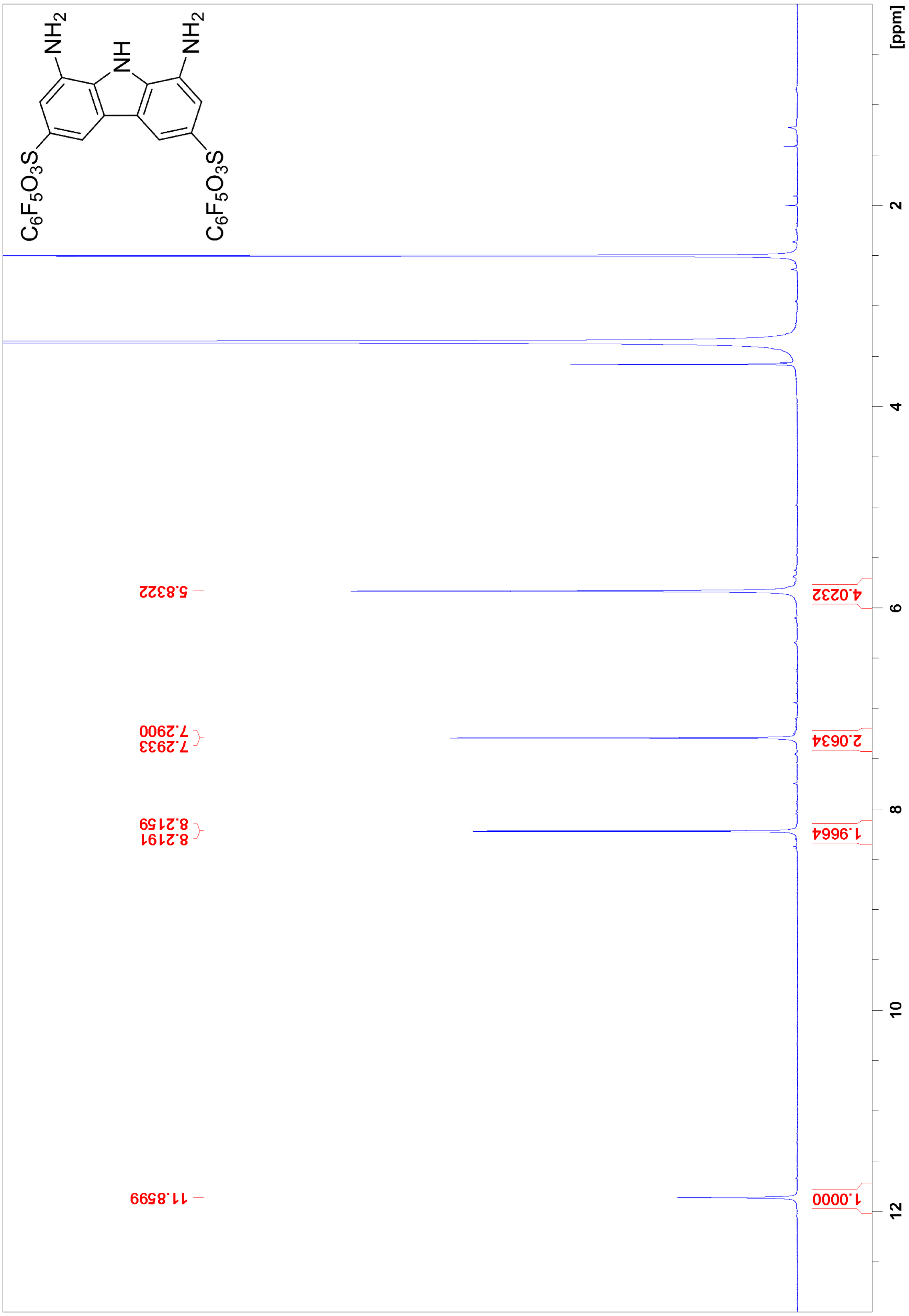

Figure S4. ^{1}H NMR spectrum of compound **9** (500 MHz, $DMSO_{d6}$).

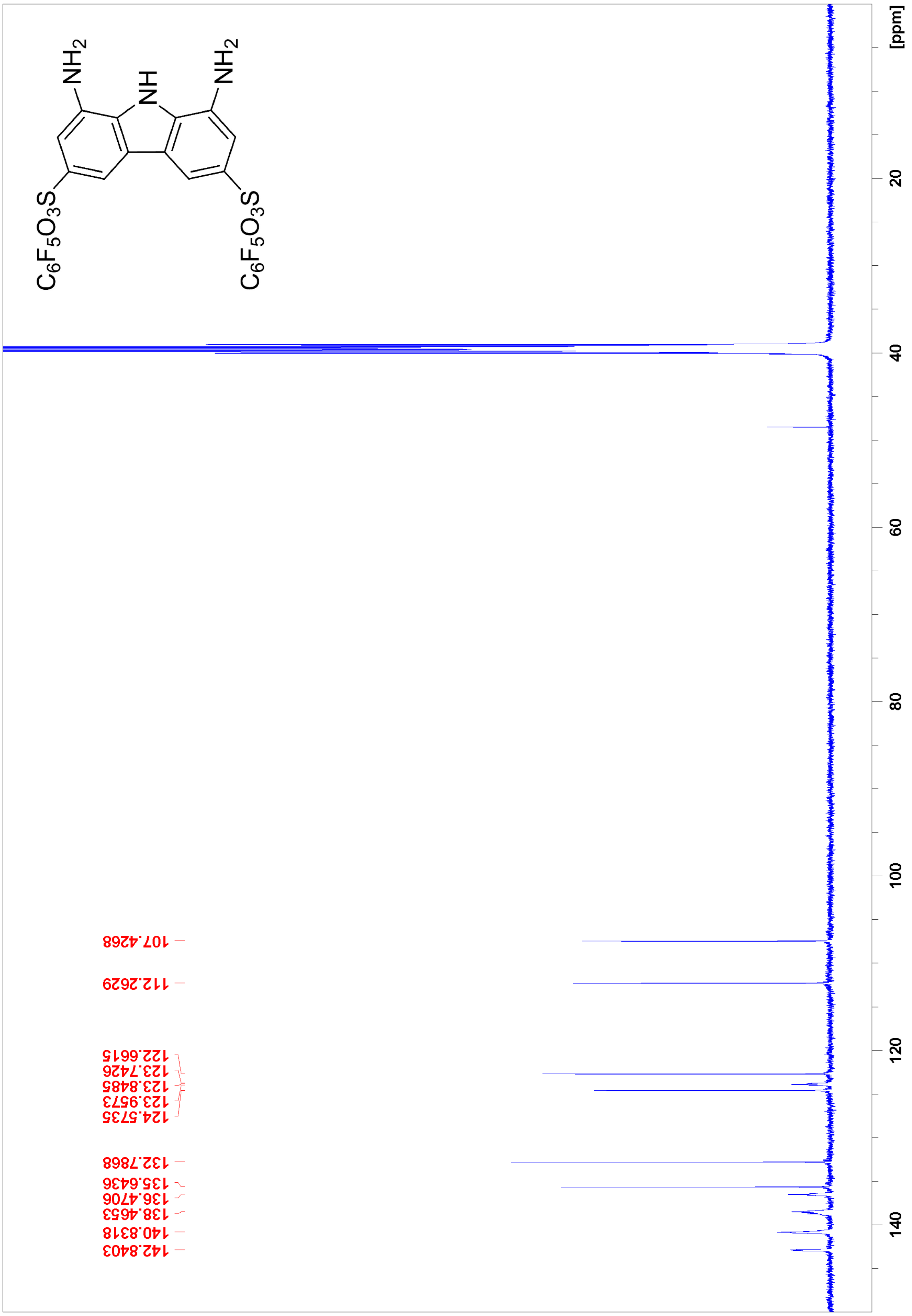

Figure S5. $^{13}C\{^{1}H\}$ NMR spectrum of compound **9** (125 MHz, DMSO$_{d6}$).

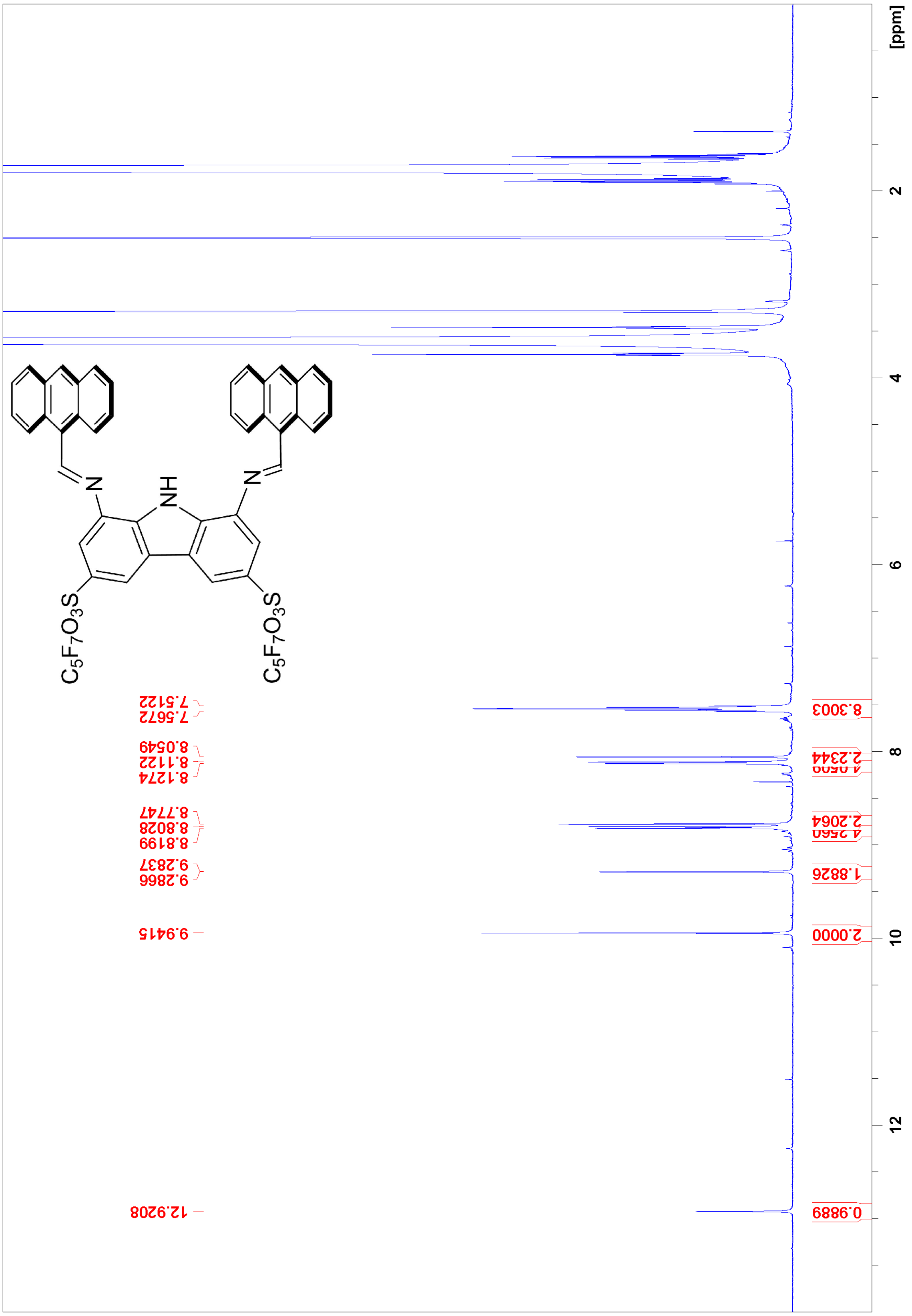

Figure S6. ^{1}H NMR spectrum of compound **11** (500 MHz, $DMSO_{d6}$).

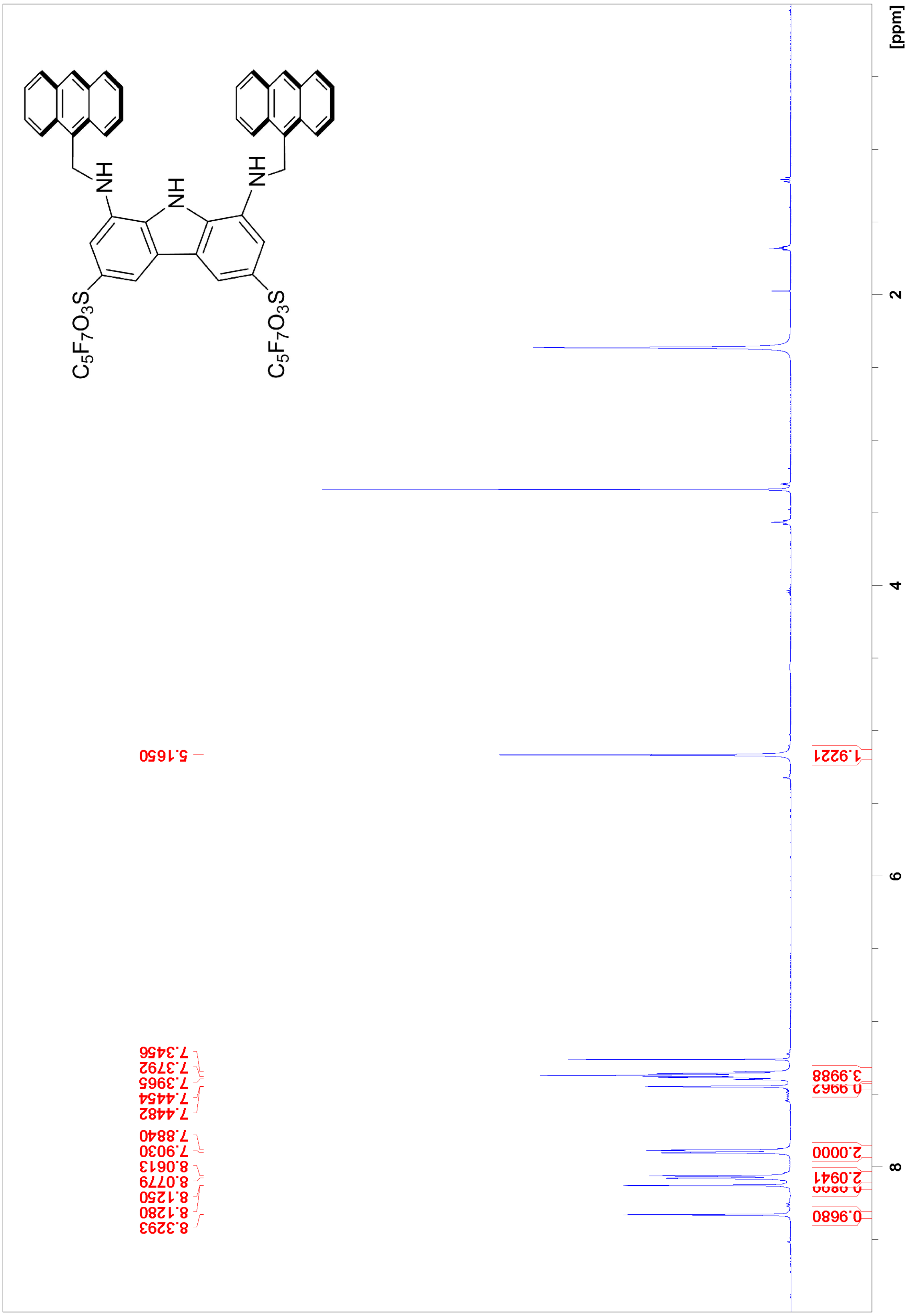

Figure S7. ^{1}H NMR spectrum of compound **12** (500 MHz, CD_3OD 3% in $CDCl_3$).

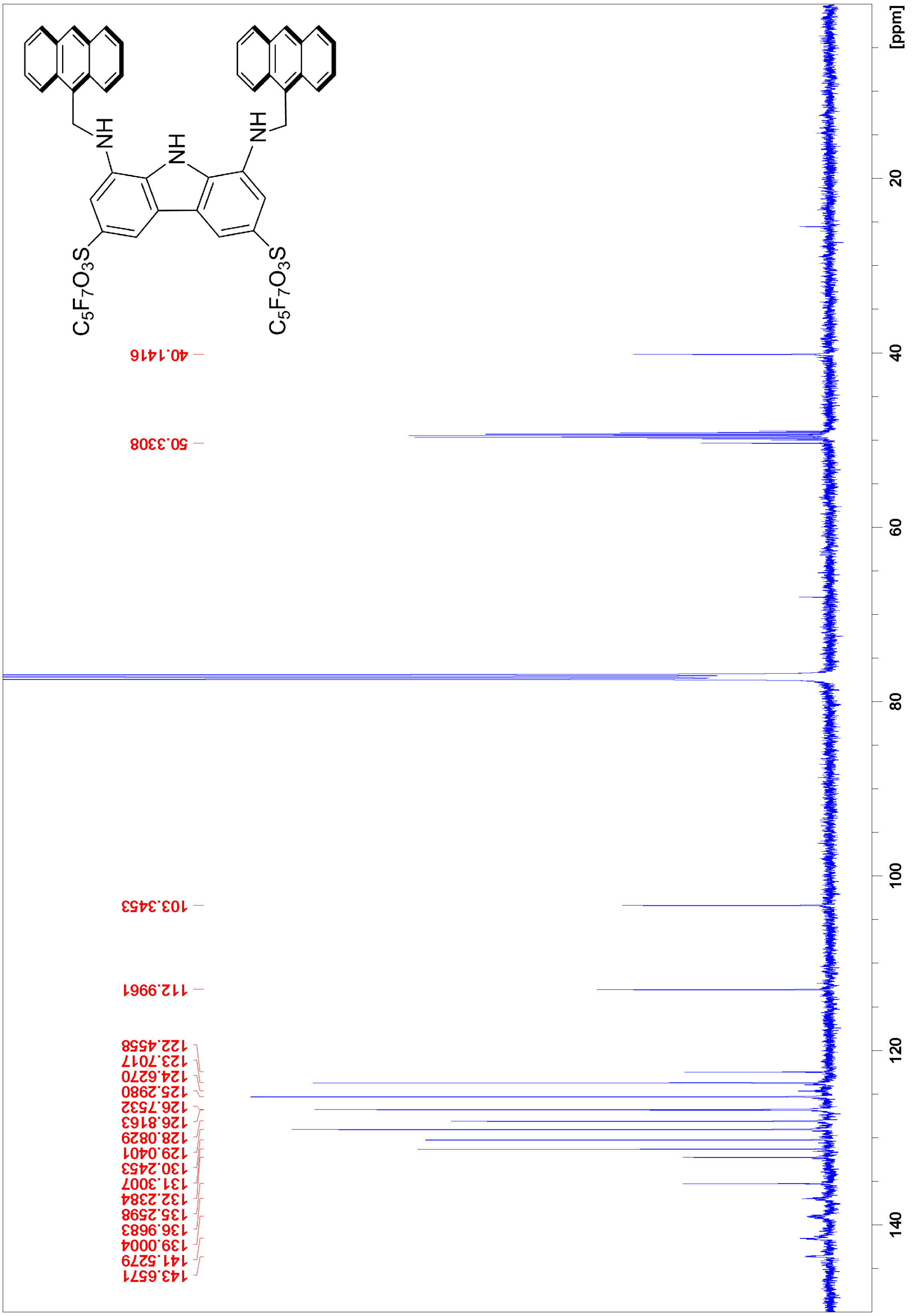

Figure S8. $^{13}C\{^{1}H\}$ NMR spectrum of compound **12** (125 MHz, CD_3OD 3% in $CDCl_3$).

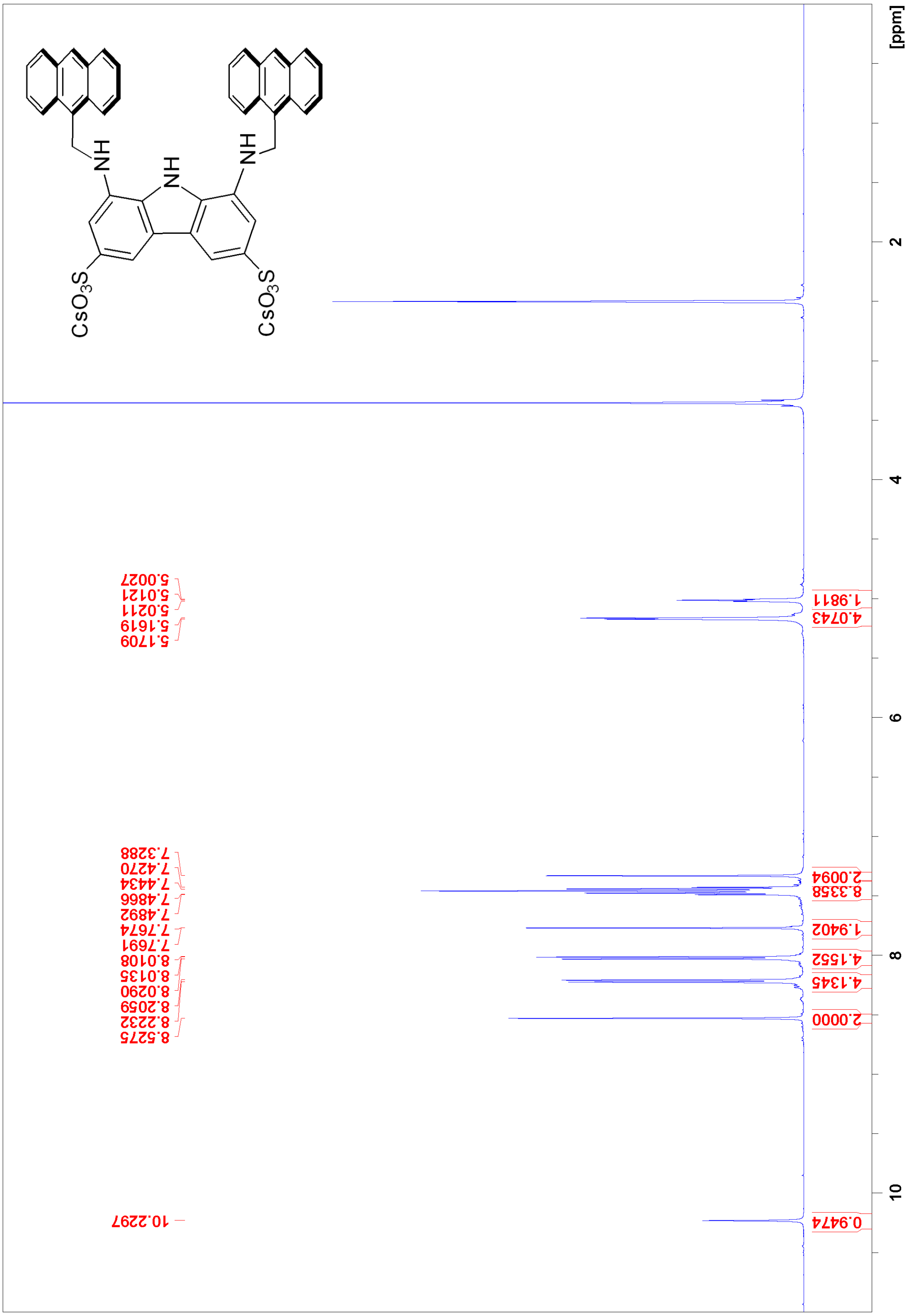

Figure S9. ^{1}H NMR spectrum of receptor **2** (500 MHz, $DMSO_{d6}$).

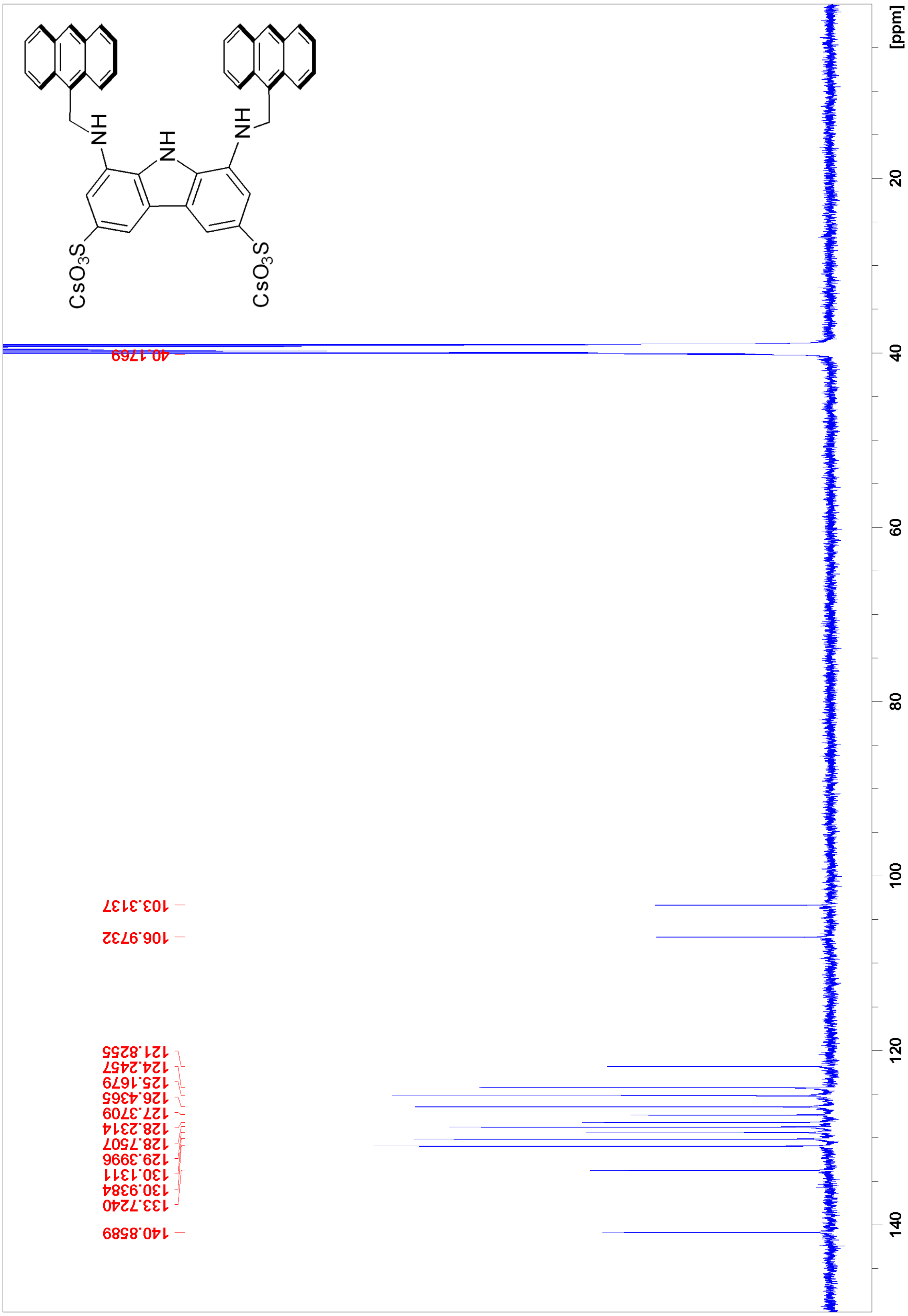

Figure S10. $^{13}C\{^1H\}$ NMR spectrum of receptor **2** (125 MHz, $DMSO_{d6}$).

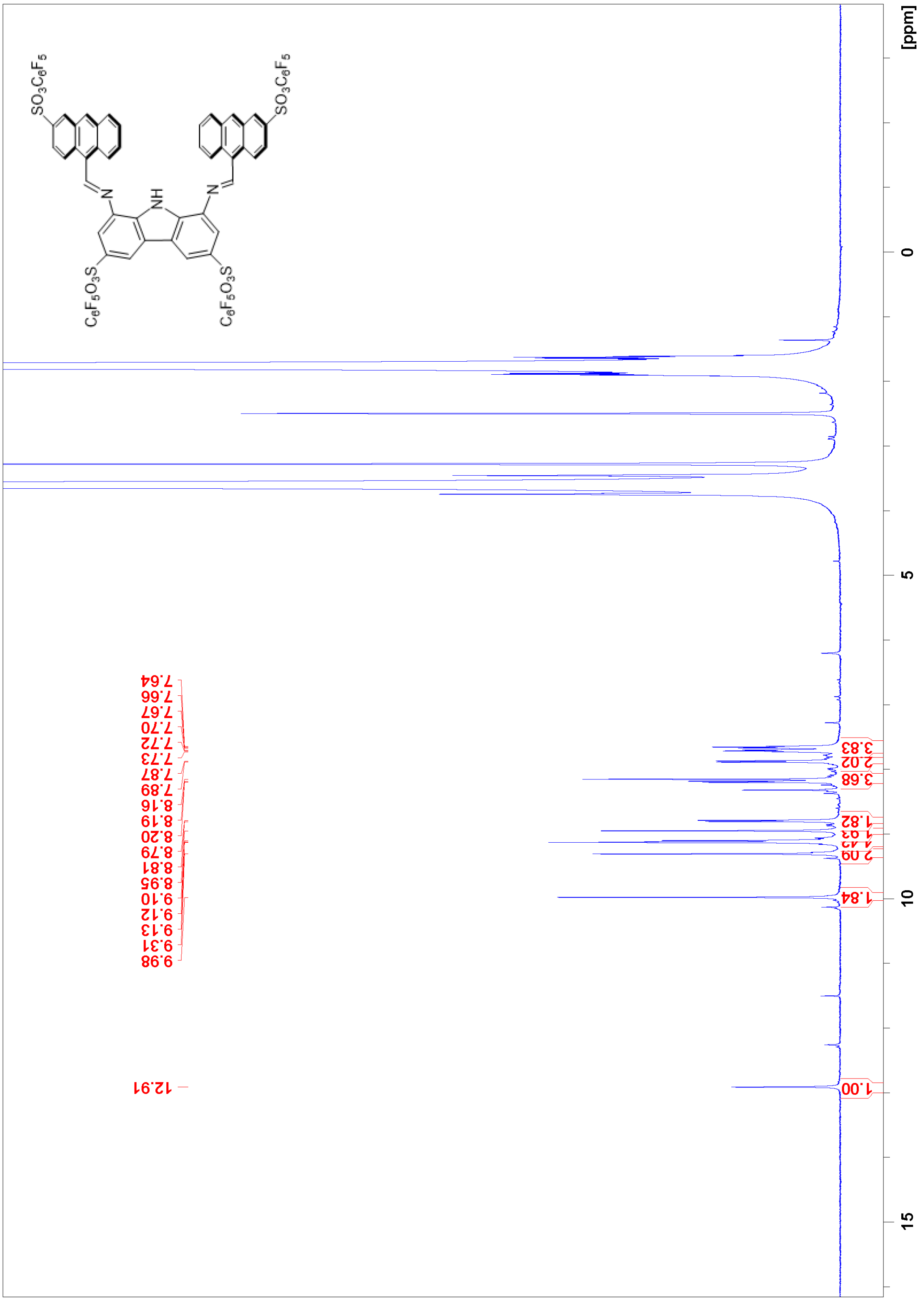

Figure S11. ^{1}H NMR spectrum of receptor **14** (500 MHz, $DMSO_{d6}$/THF).

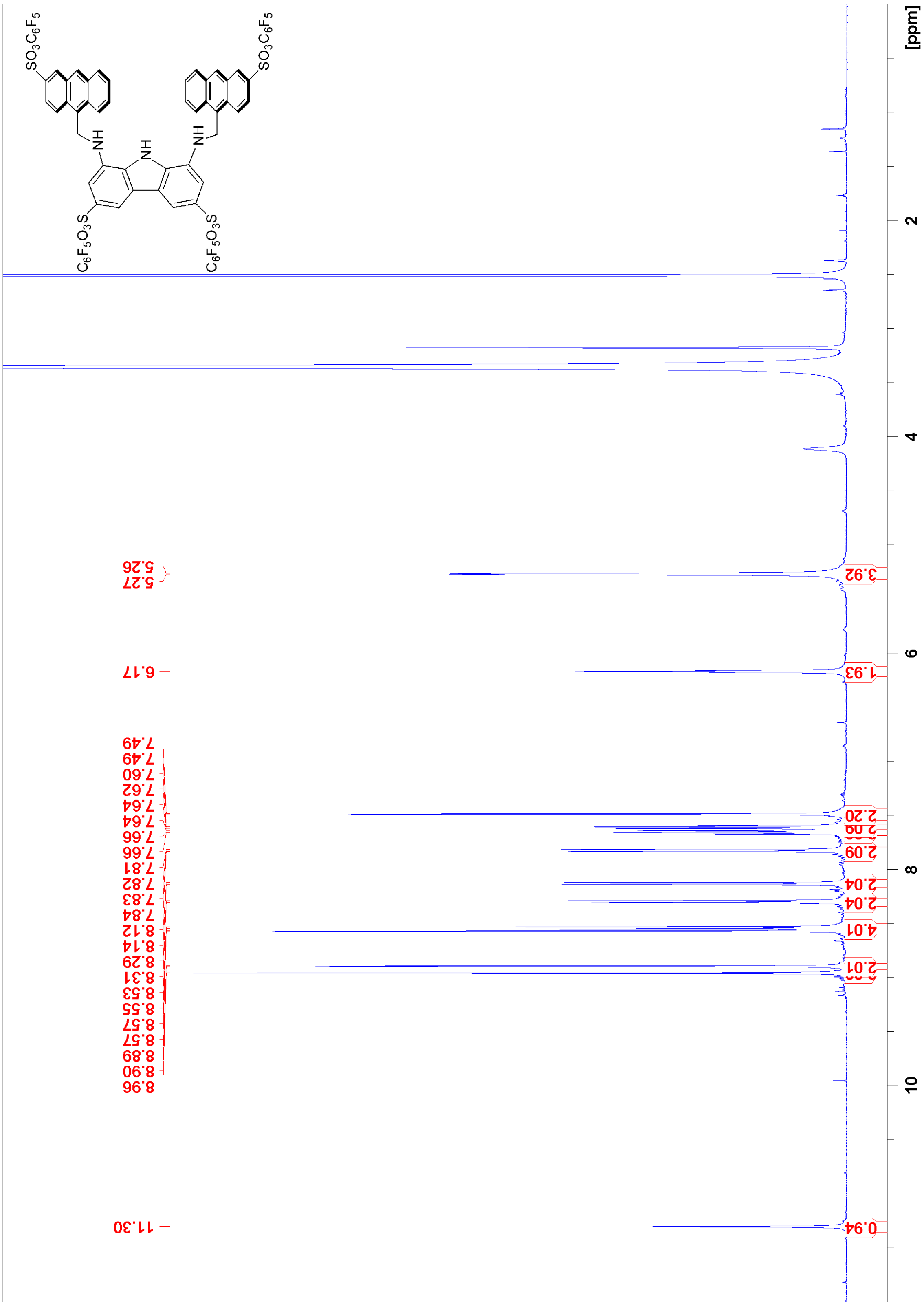

Figure S12. ^{1}H NMR spectrum of receptor **15** (500 MHz, DMSO$_{d6}$).

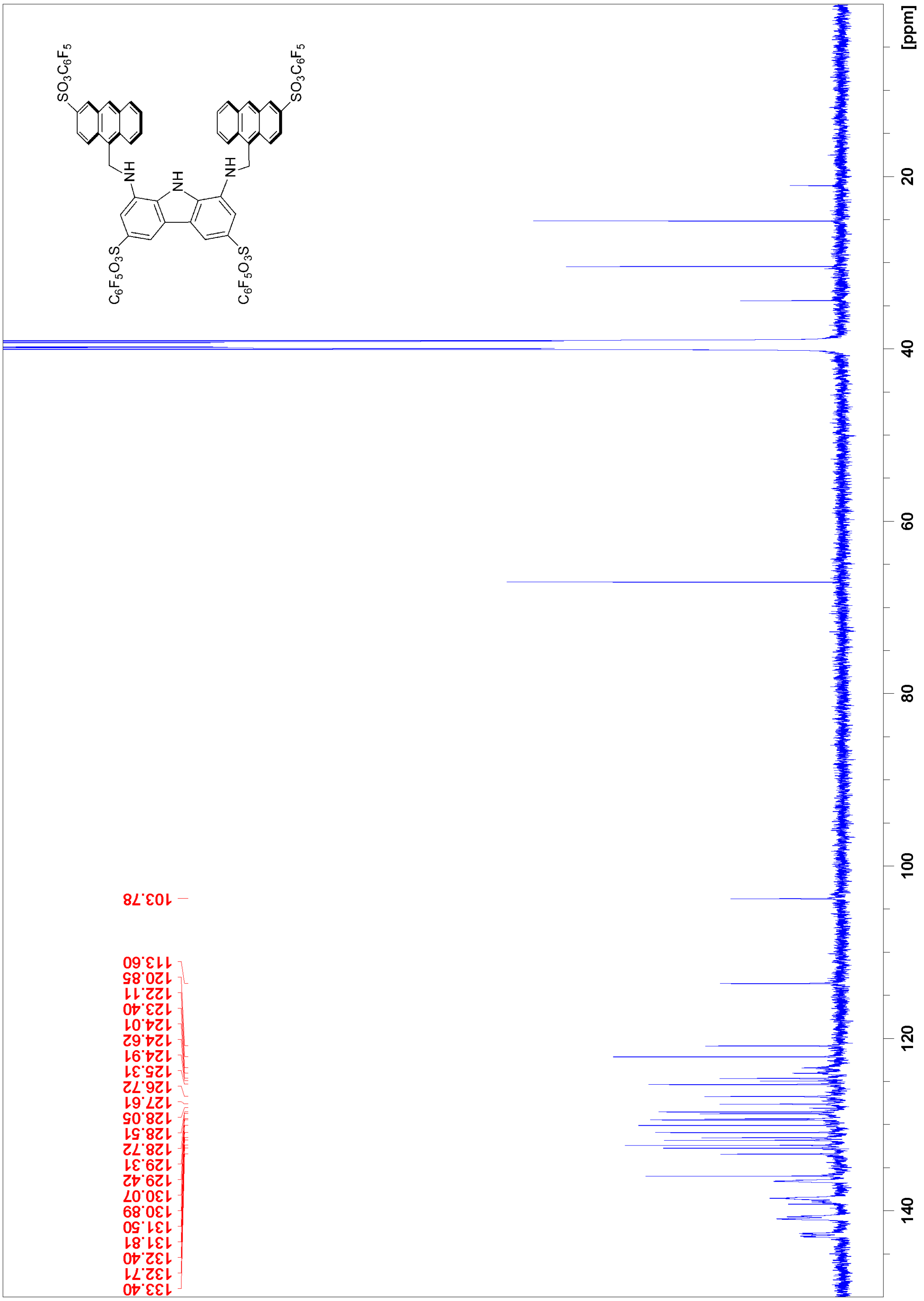

Figure S13. $^{13}C\{^{1}H\}$ NMR spectrum of receptor **15** (125 MHz, DMSOd6).

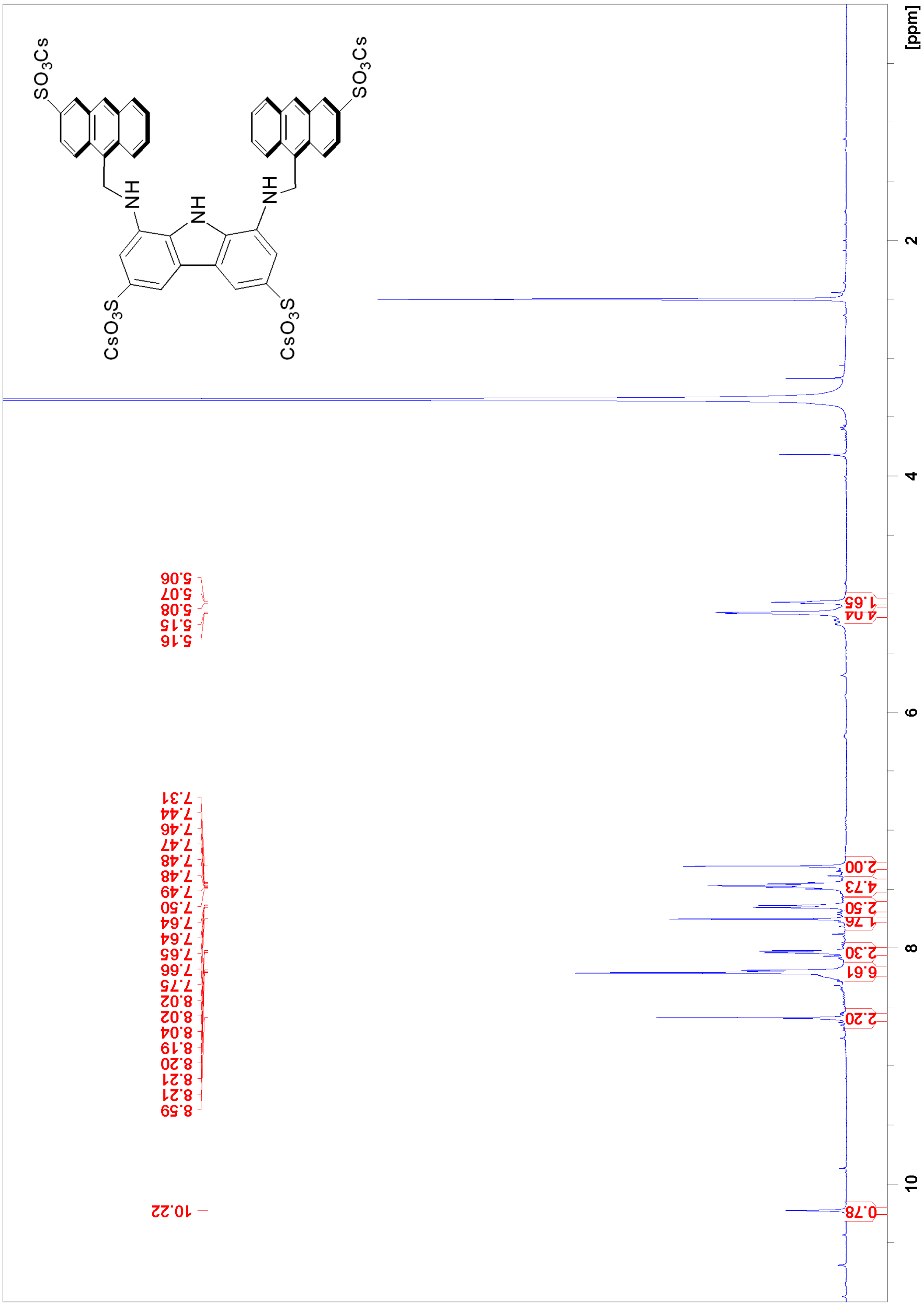

Figure S14. ^{1}H NMR spectrum of receptor **4** (500 MHz, DMSO$_{d6}$).

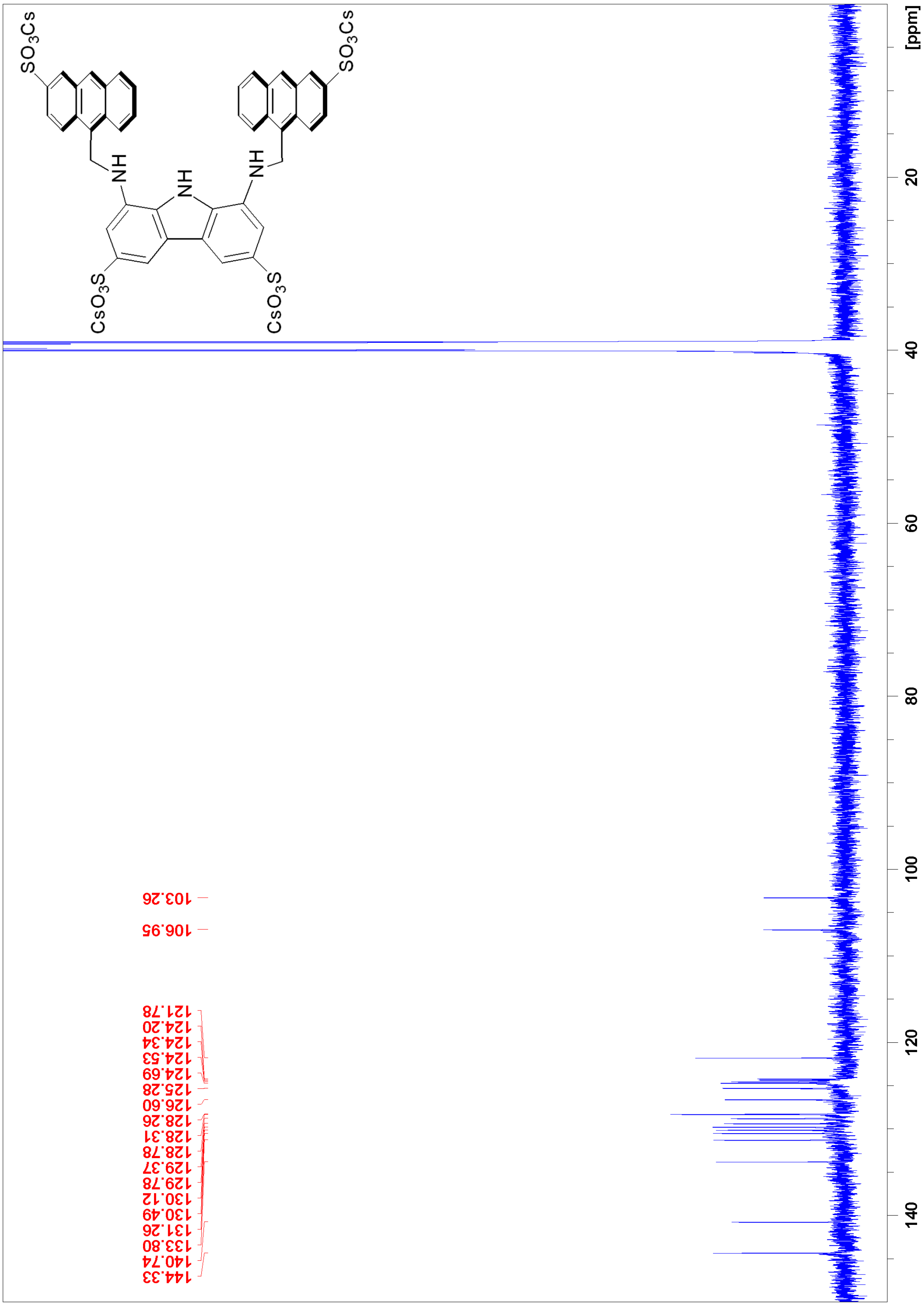

Figure S15. $^{13}C\{^{1}H\}$ NMR spectrum of receptor **4** (125 MHz, $DMSO_{d6}$)

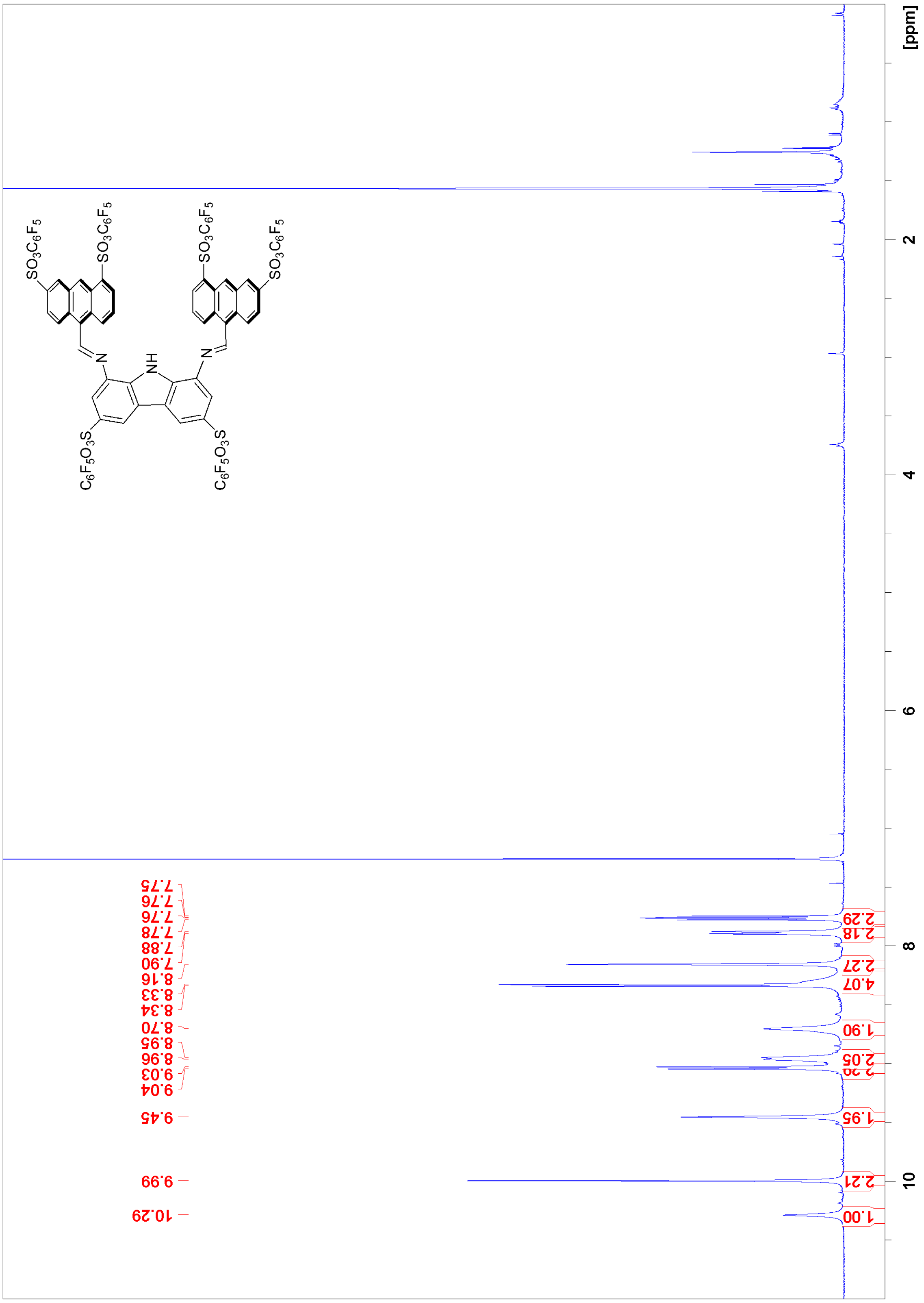

Figure S16. ^{1}H NMR spectrum of receptor **17** (500 MHz, $CDCl_3$).

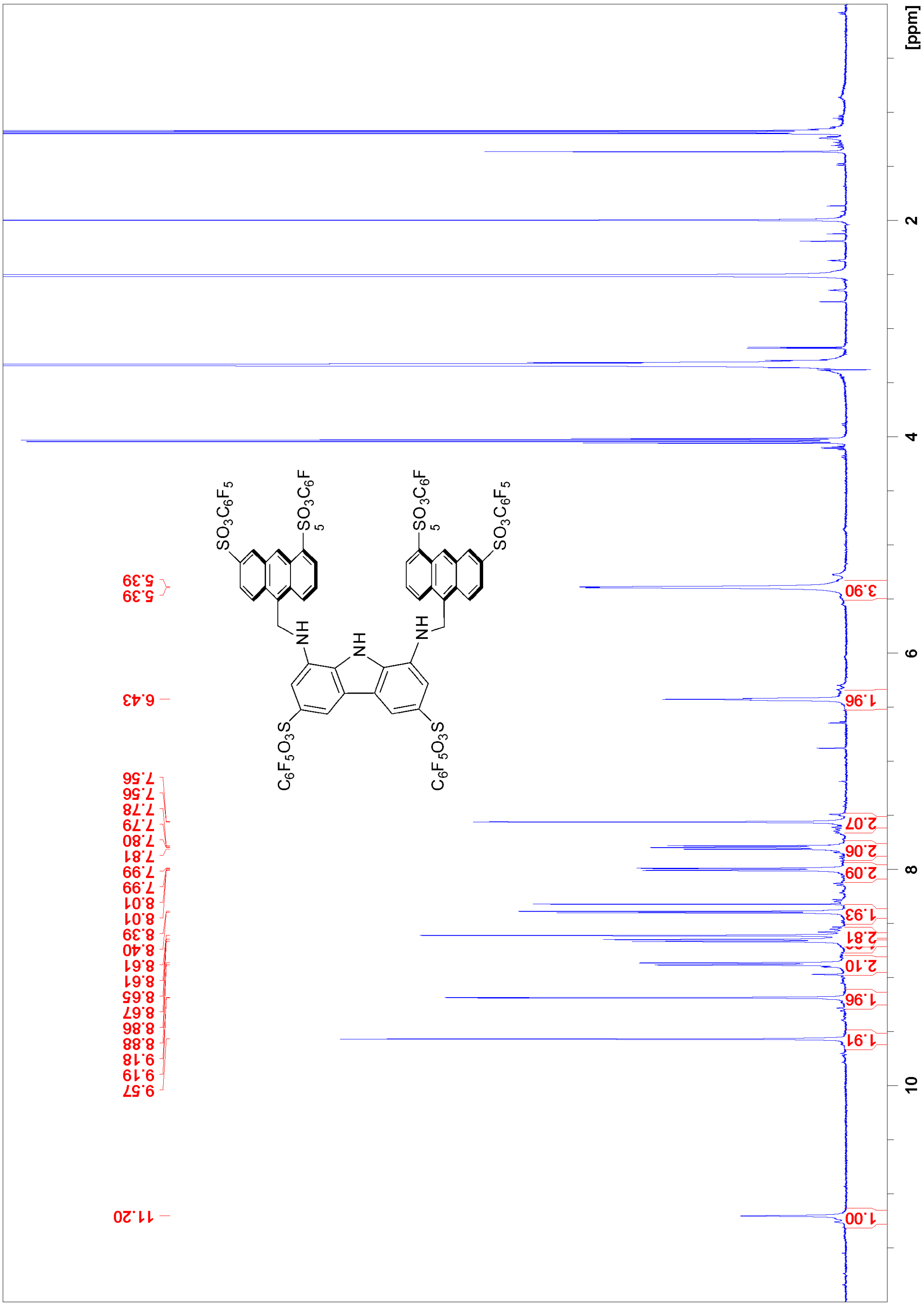

Figure S17. ^{1}H NMR spectrum of receptor **18** (500 MHz, DMSOd6).

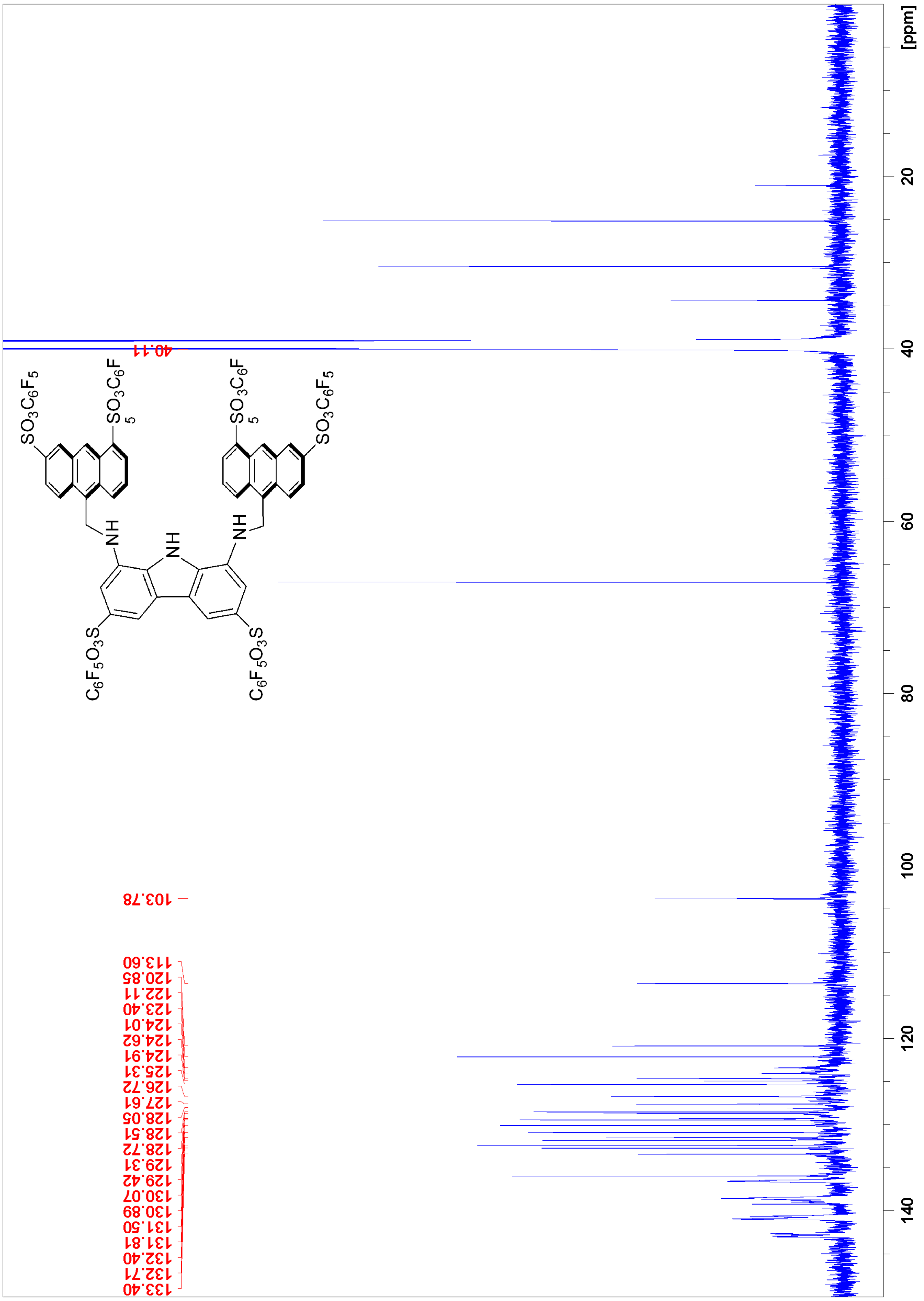

Figure S18. $^{13}C\{^{1}H\}$ NMR spectrum of receptor **18** (125 MHz, $DMSO_{d6}$)

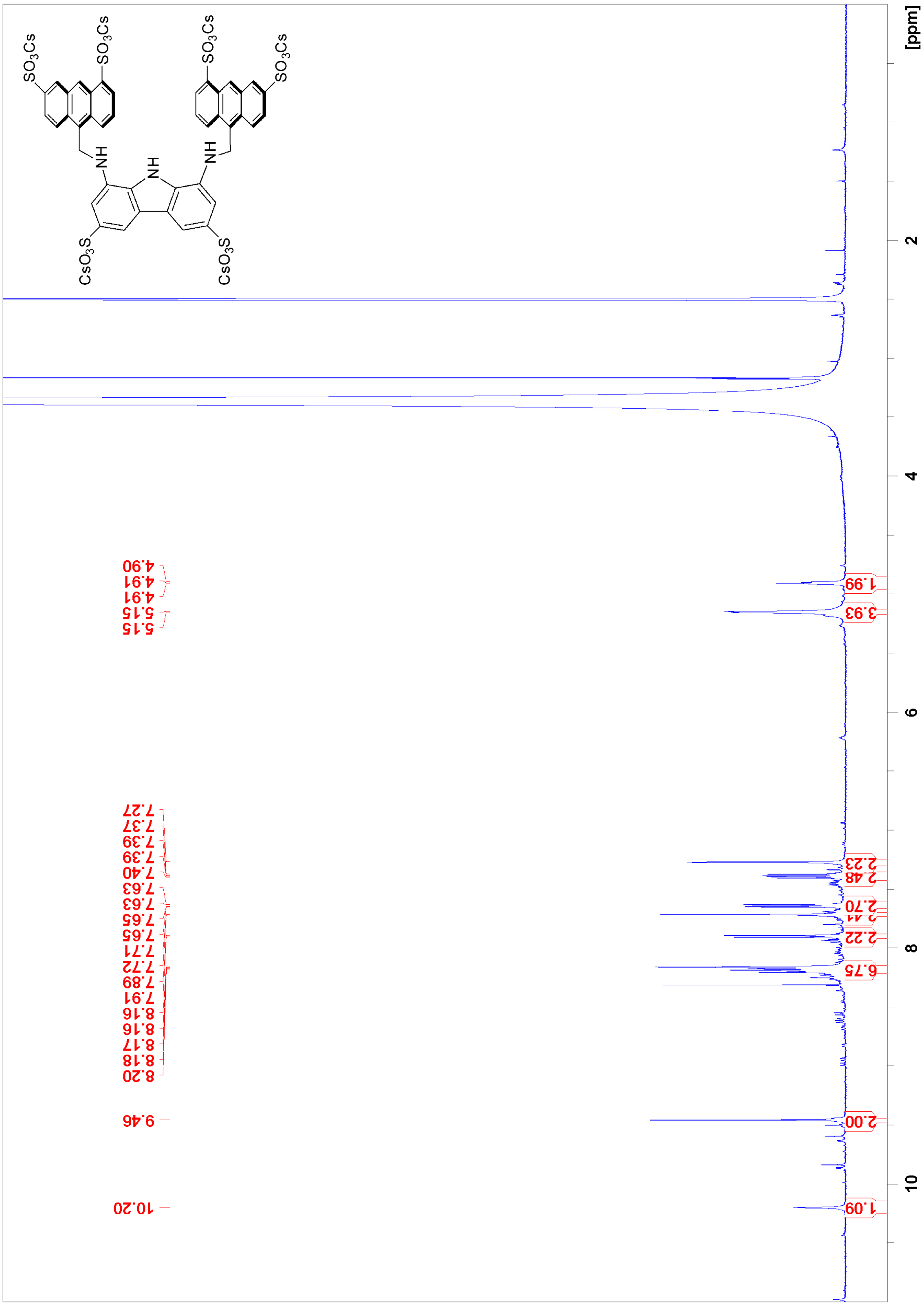

Figure S19. ^{1}H NMR spectrum of receptor **5** (500 MHz, DMSOd6).

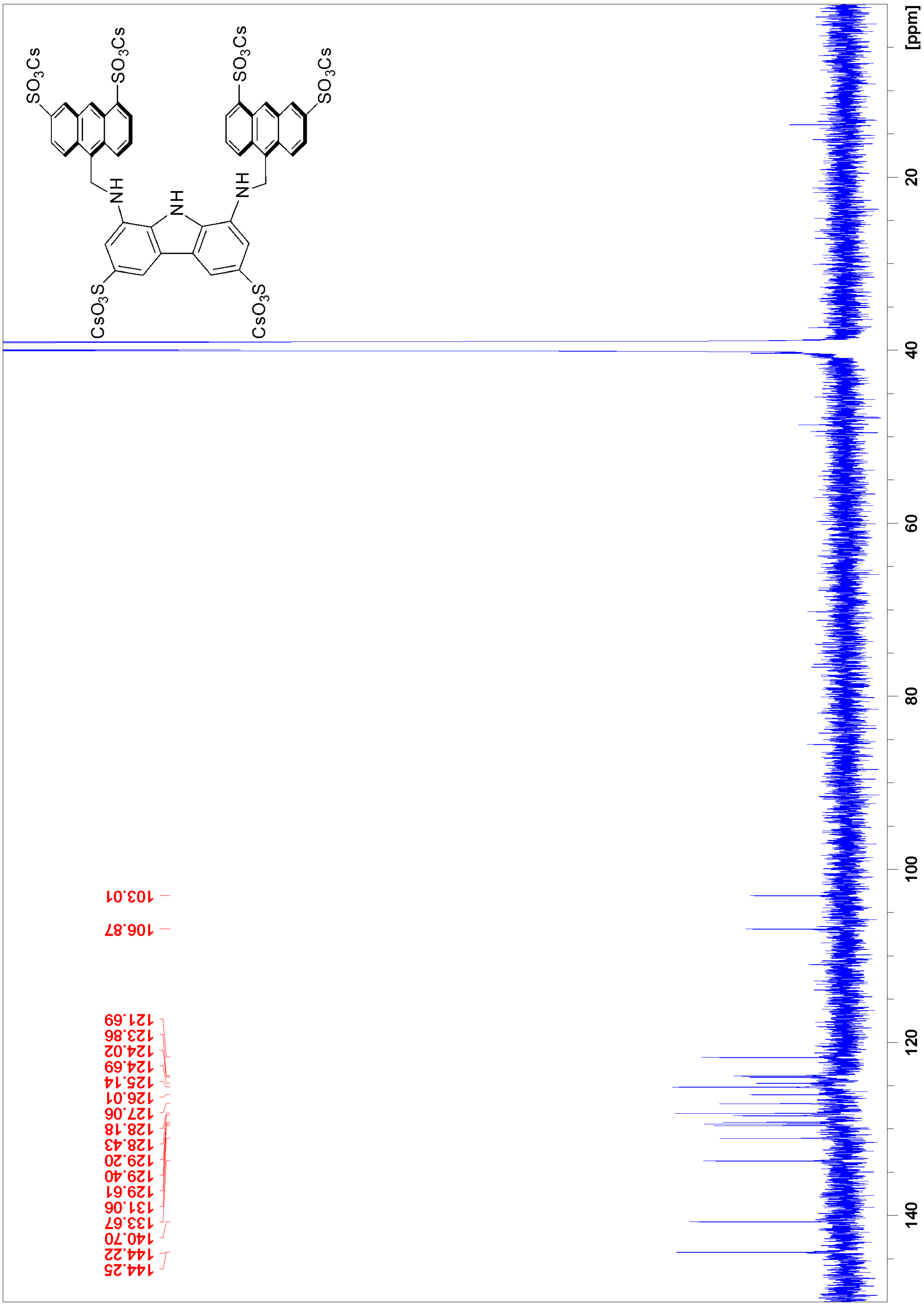

Figure S20. $^{13}C\{^{1}H\}$ NMR spectrum of receptor **5** (125 MHz, $DMSO_{d6}$)

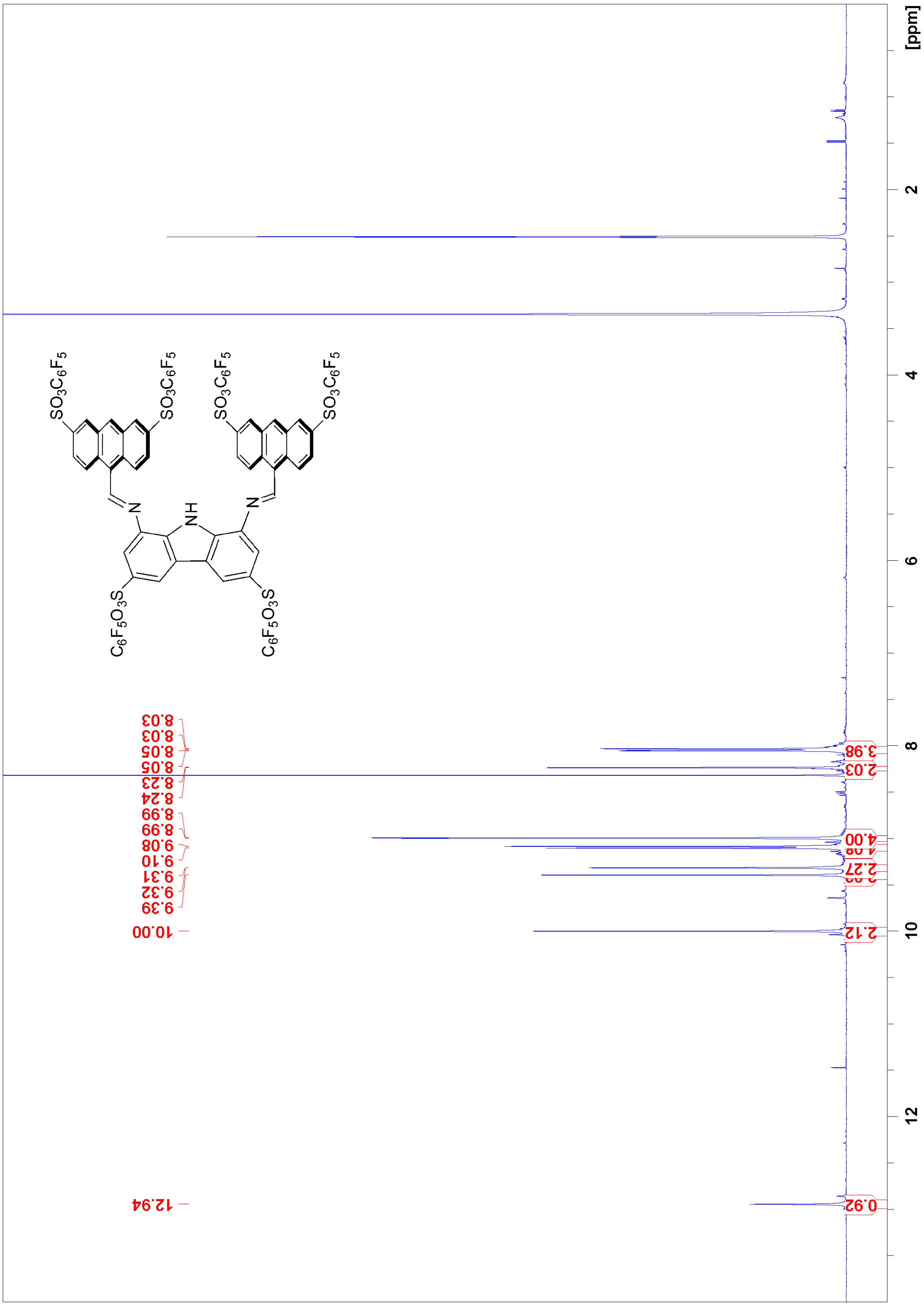

Figure S21. ^{1}H NMR spectrum of receptor **20** (500 MHz, $CDCl_3$).

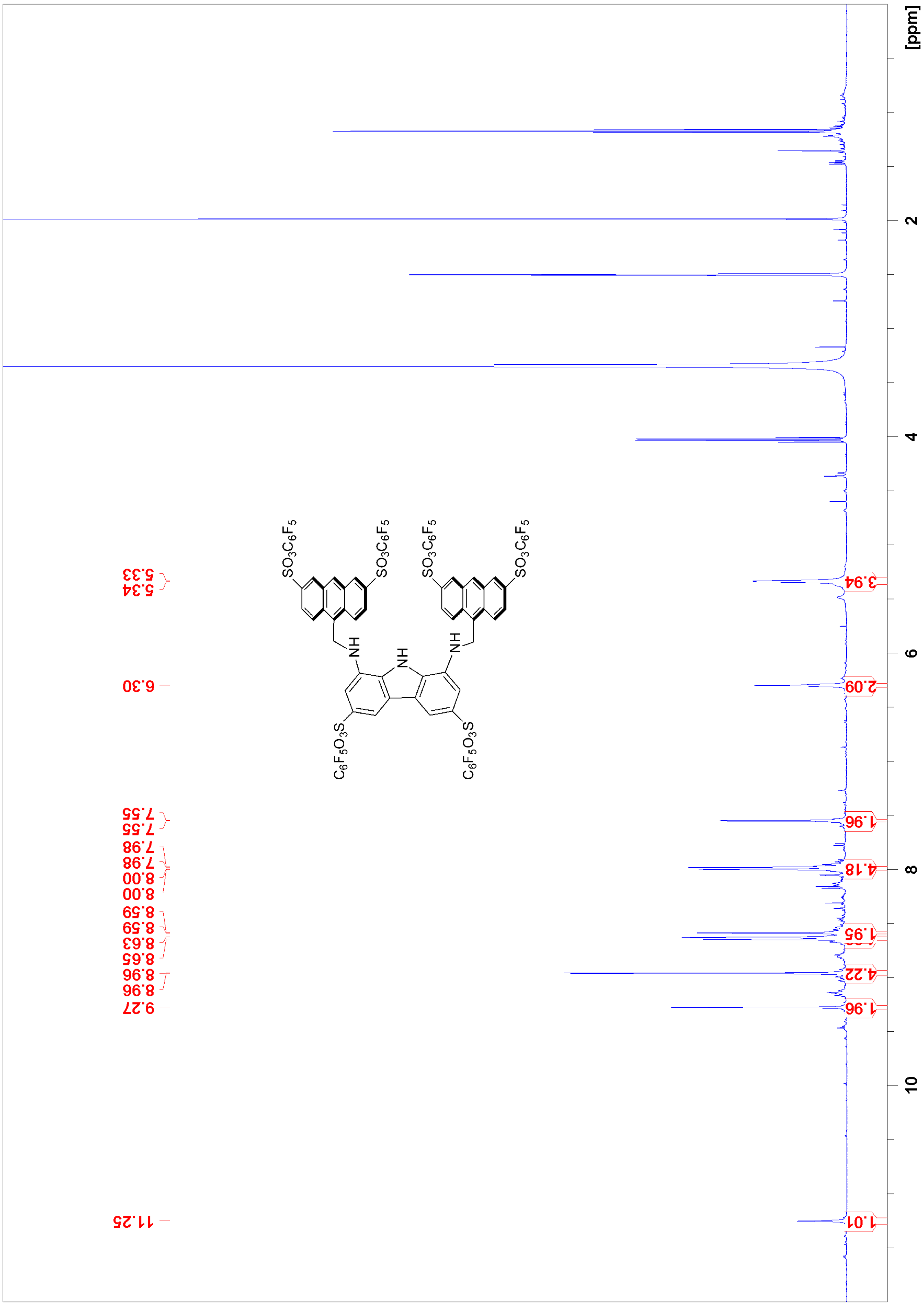

Figure S22. ^{1}H NMR spectrum of receptor **21** (500 MHz, $DMSO_{d6}$).

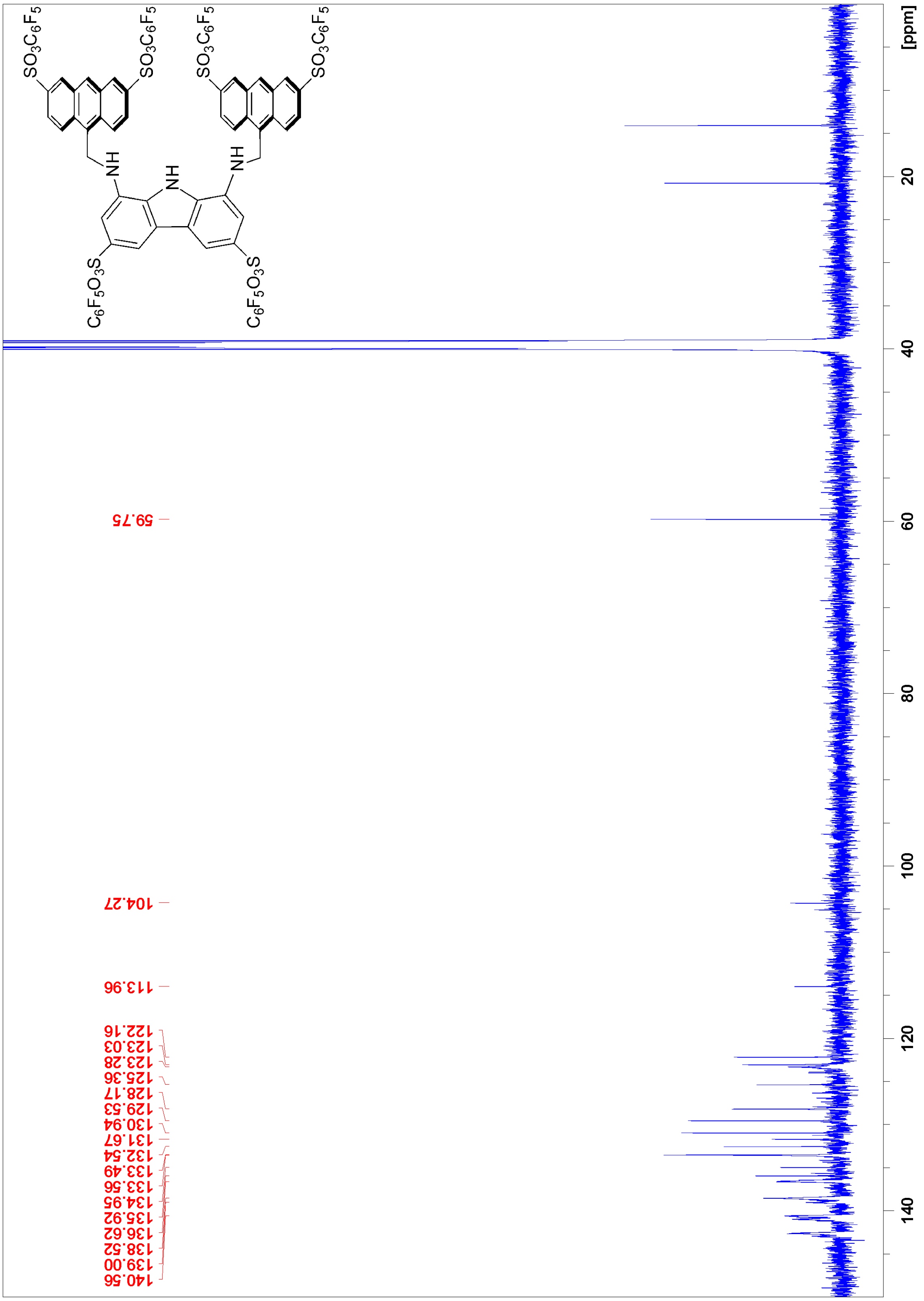

Figure S23. $^{13}C\{^{1}H\}$ NMR spectrum of receptor **21** (125 MHz, DMSO$_{d6}$)

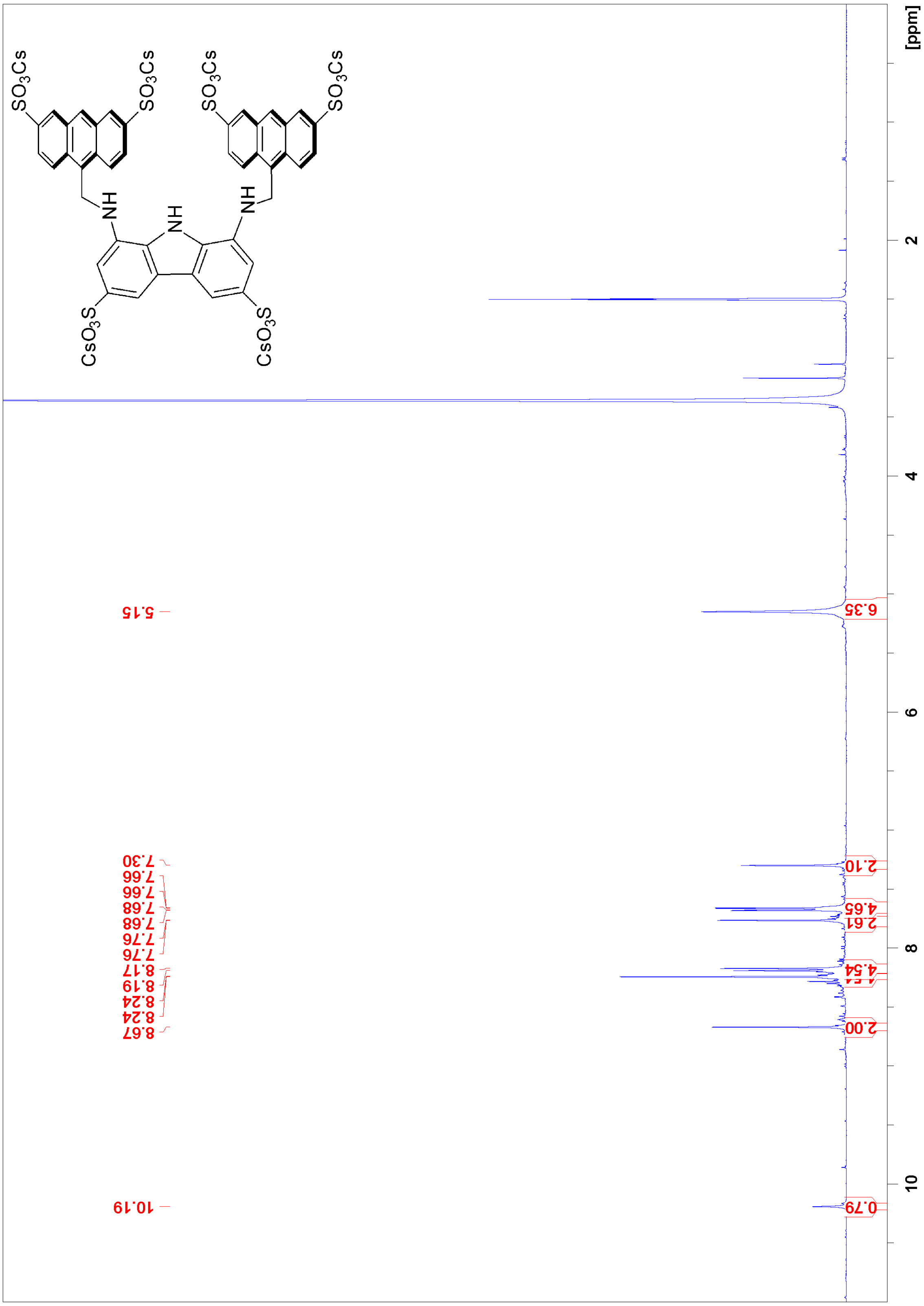

Figure S24. ^{1}H NMR spectrum of receptor **6** (500 MHz, DMSO$_{d6}$).

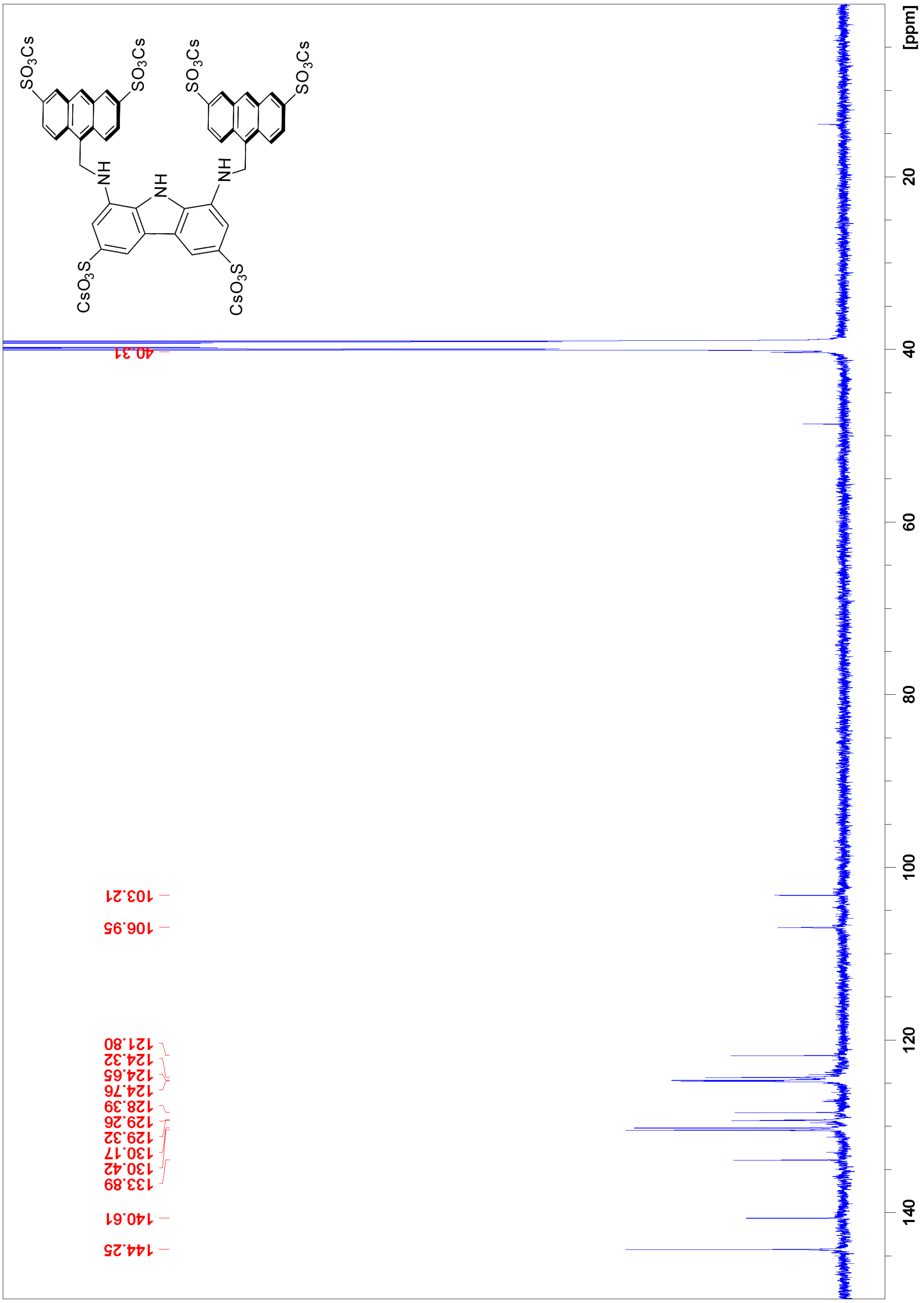

Figure S25. $^{13}C\{^1H\}$ NMR spectrum of receptor **6** (125 MHz, $DMSO_{d6}$)

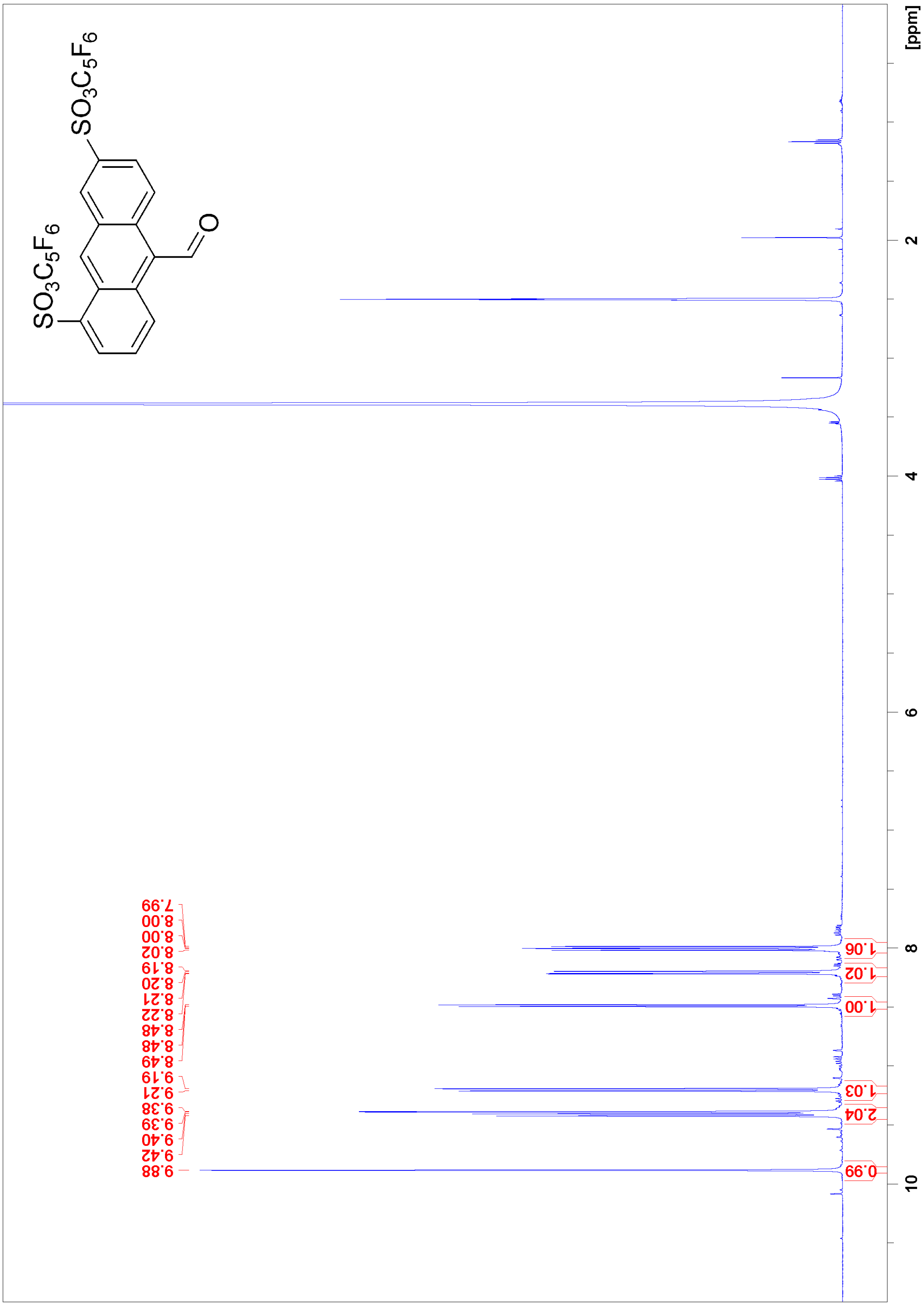

Figure S26. ^{1}H NMR spectrum of receptor **16** (500 MHz, $DMSO_{d6}$).

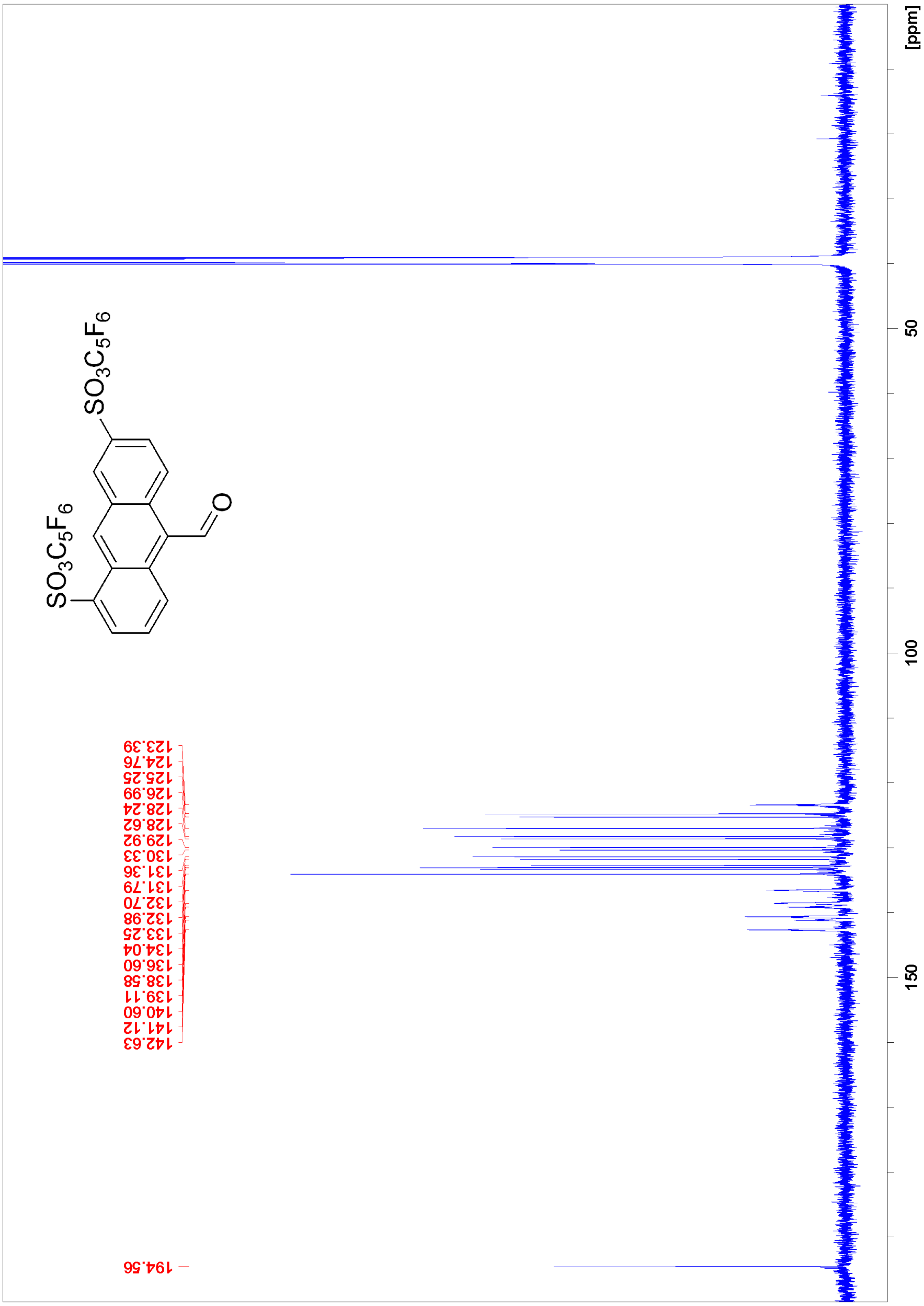

Figure S27. $^{13}C\{^{1}H\}$ NMR spectrum of receptor 1**6** (125 MHz, $DMSO_{d6}$)

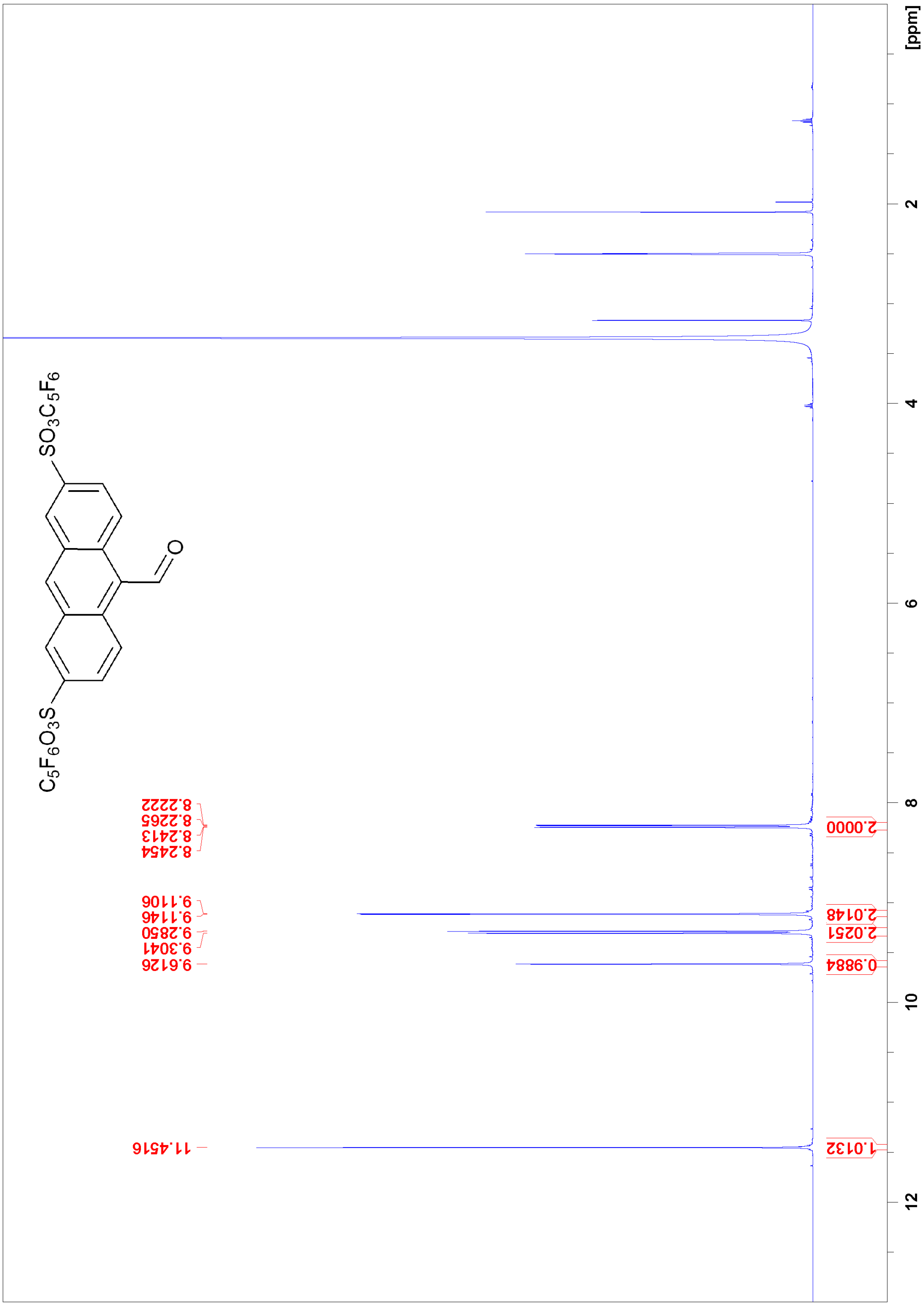

Figure S28. ^{1}H NMR spectrum of receptor **19** (500 MHz, $DMSO_{d6}$).

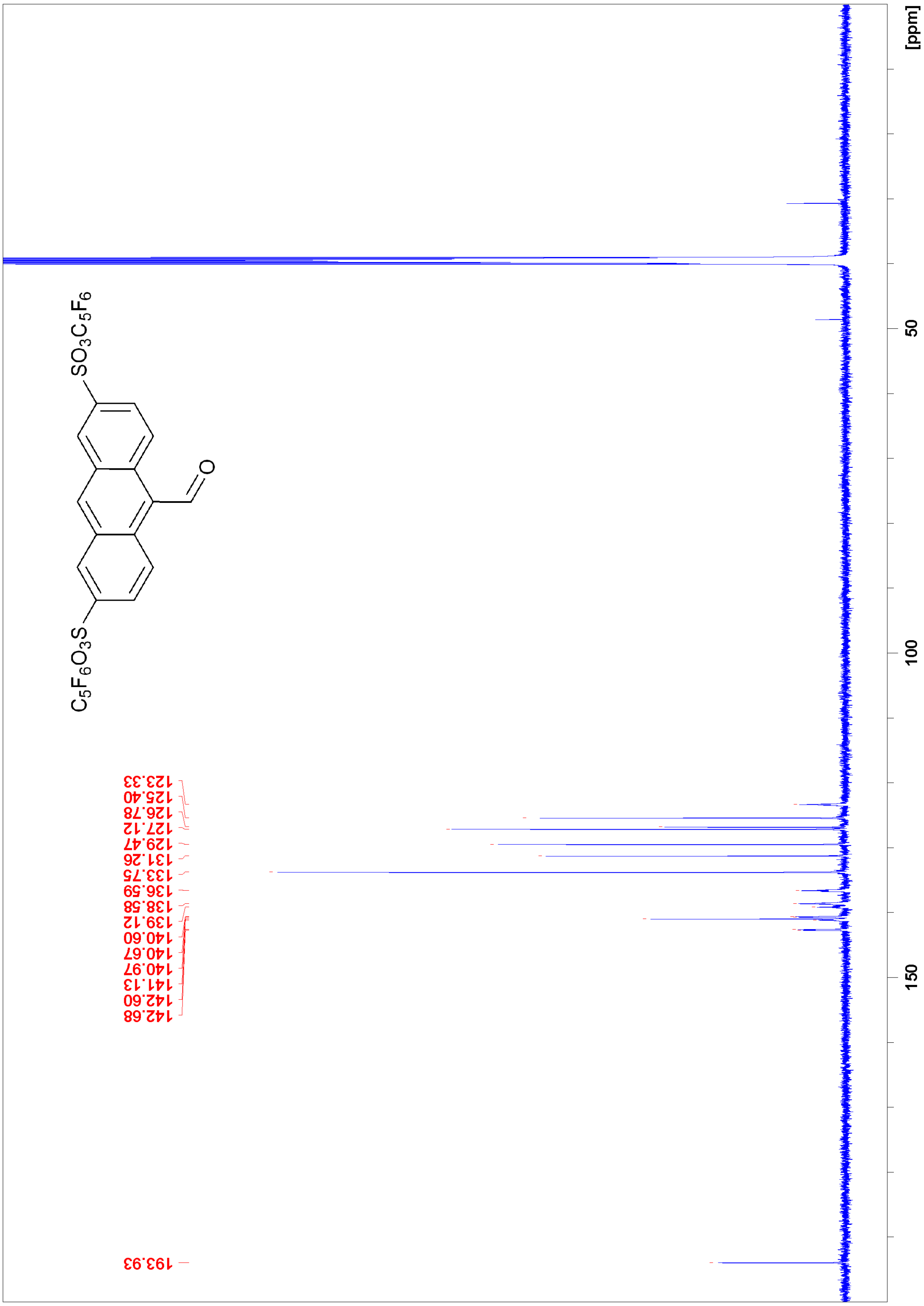

Figure S29. $^{13}C\{^{1}H\}$ NMR spectrum of receptor 1**9** (125 MHz, $DMSO_{d6}$)

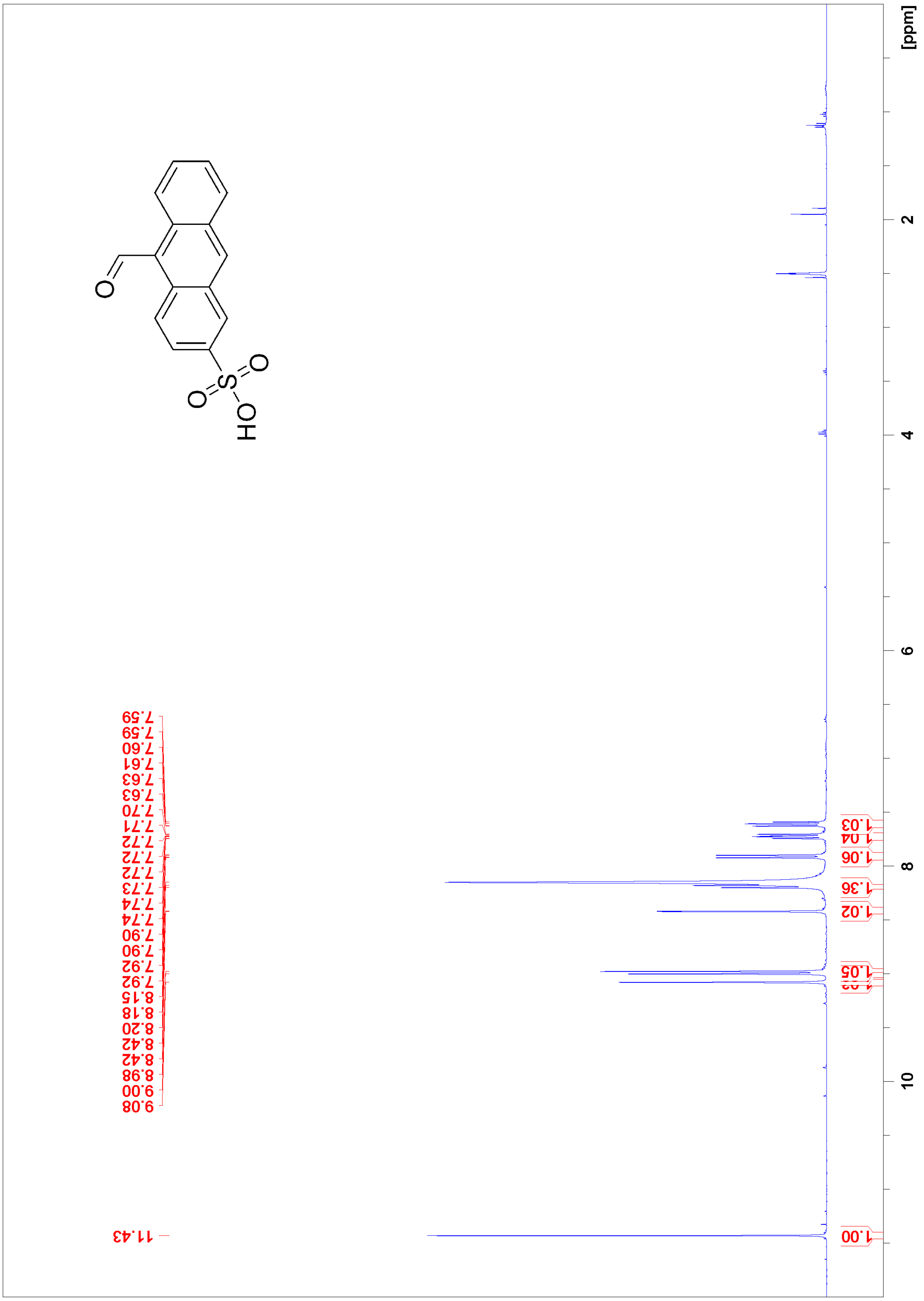

Figure S30. ^{1}H NMR spectrum of receptor **22** (400 MHz, $DMSO_{d6}$).

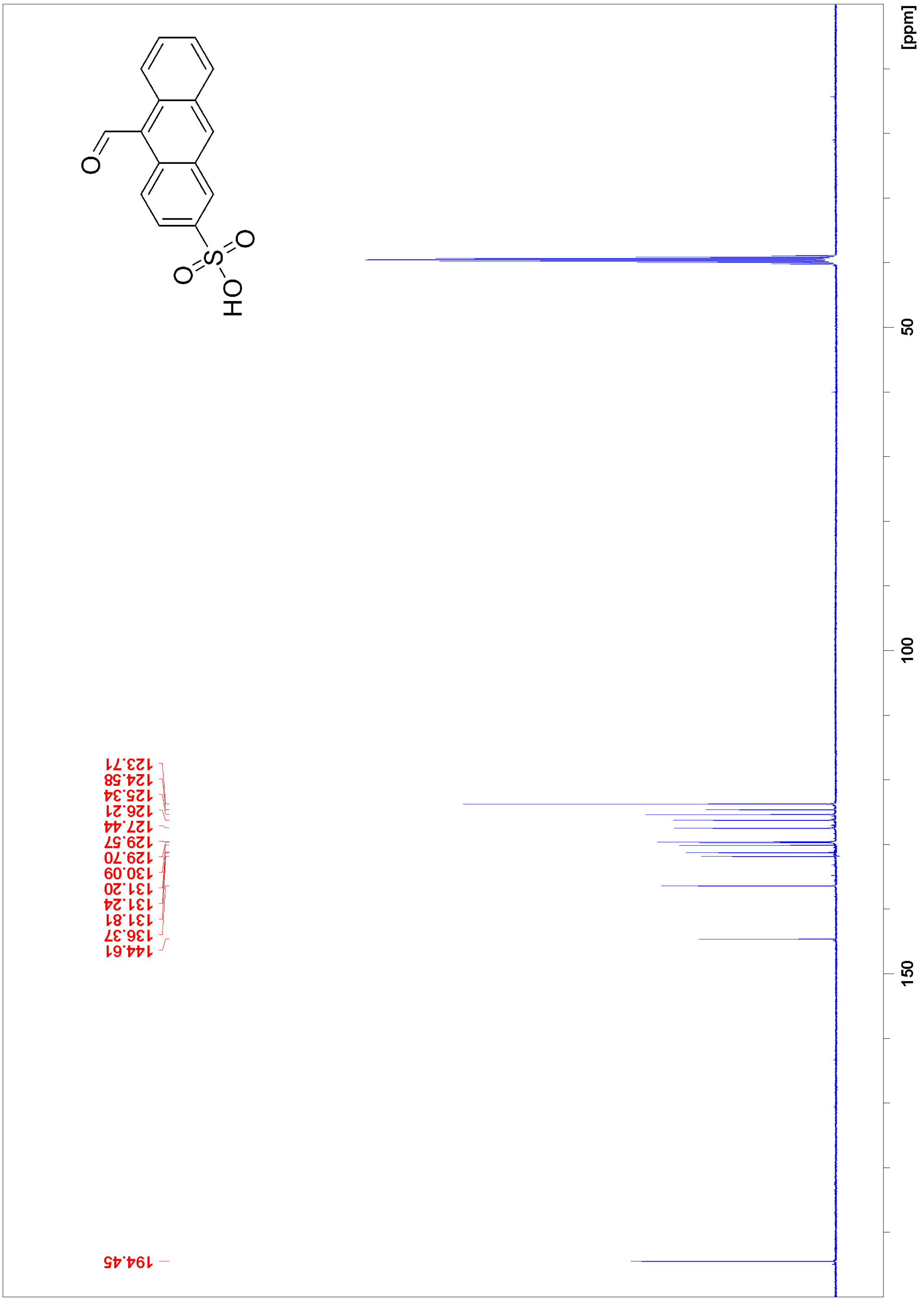

Figure S31. $^{13}C\{^{1}H\}$ NMR spectrum of receptor **22** (100 MHz, $DMSO_{d6}$)

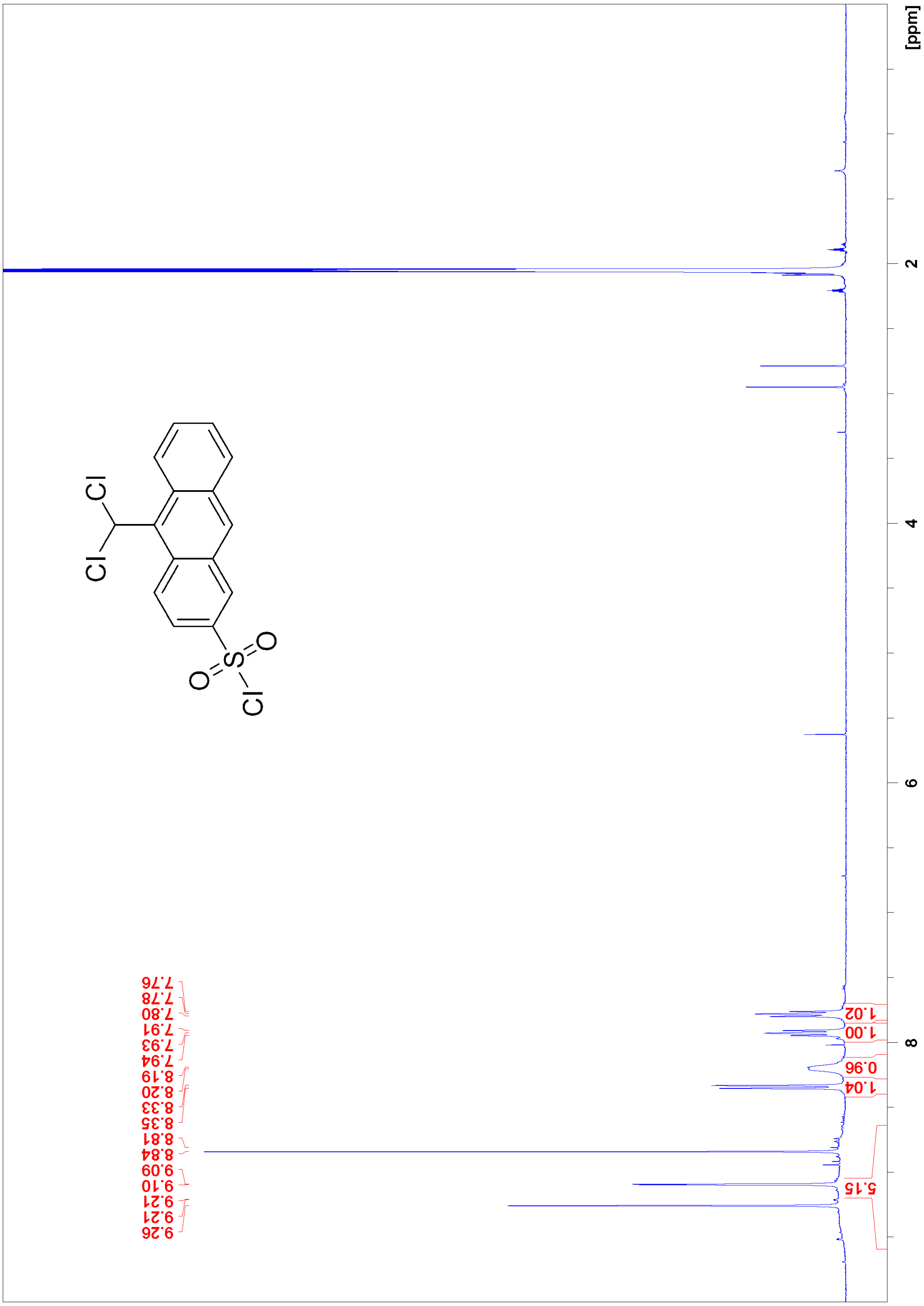

Figure S32. ^{1}H NMR spectrum of receptor **23** (400 MHz, Acetone-d6).

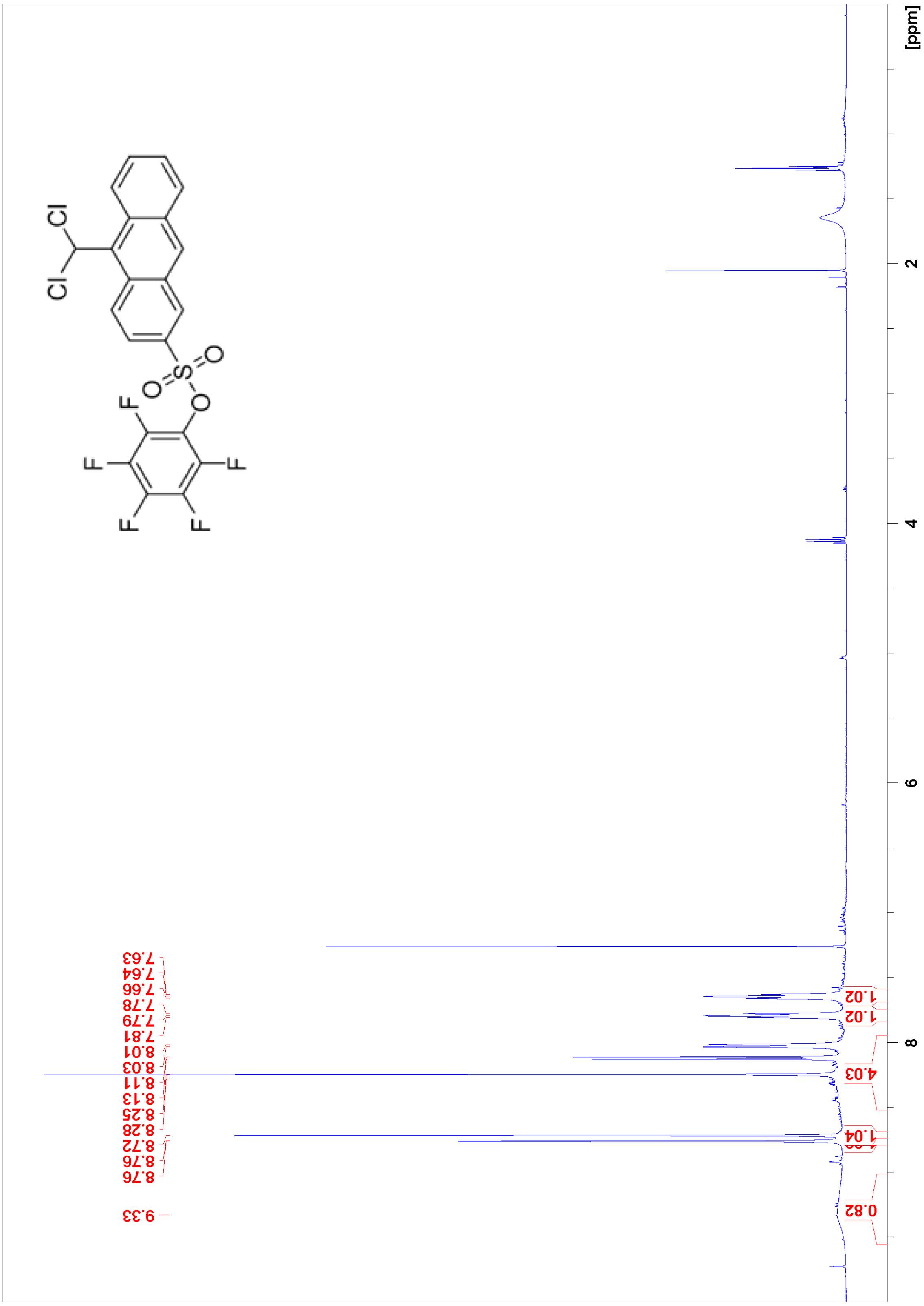

Figure S33. ^{1}H NMR spectrum of receptor **24** (500 MHz, $CDCl_3$).

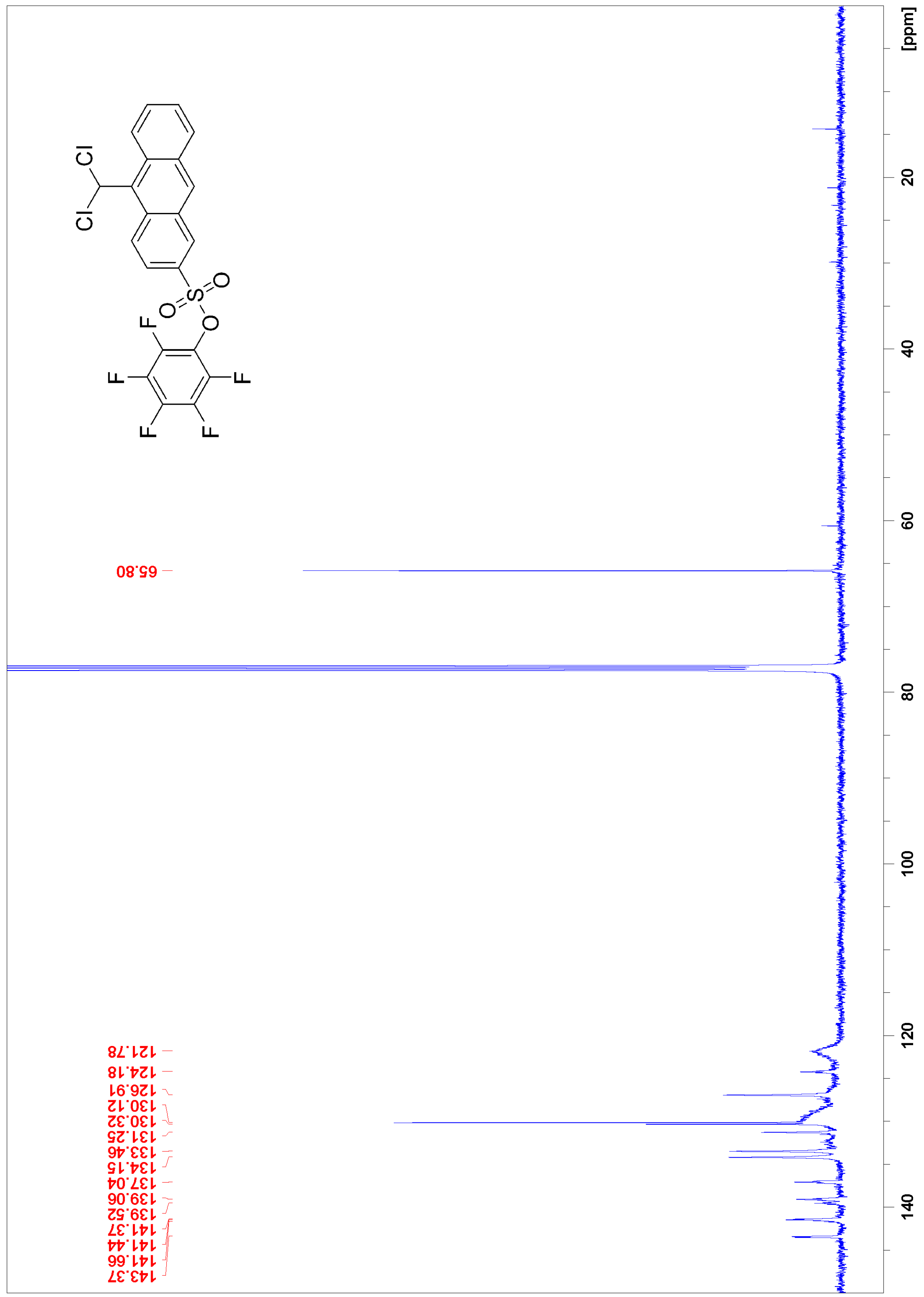

Figure S34. $^{13}C\{^{1}H\}$ NMR spectrum of receptor **24** (125 MHz, $CDCl_3$)

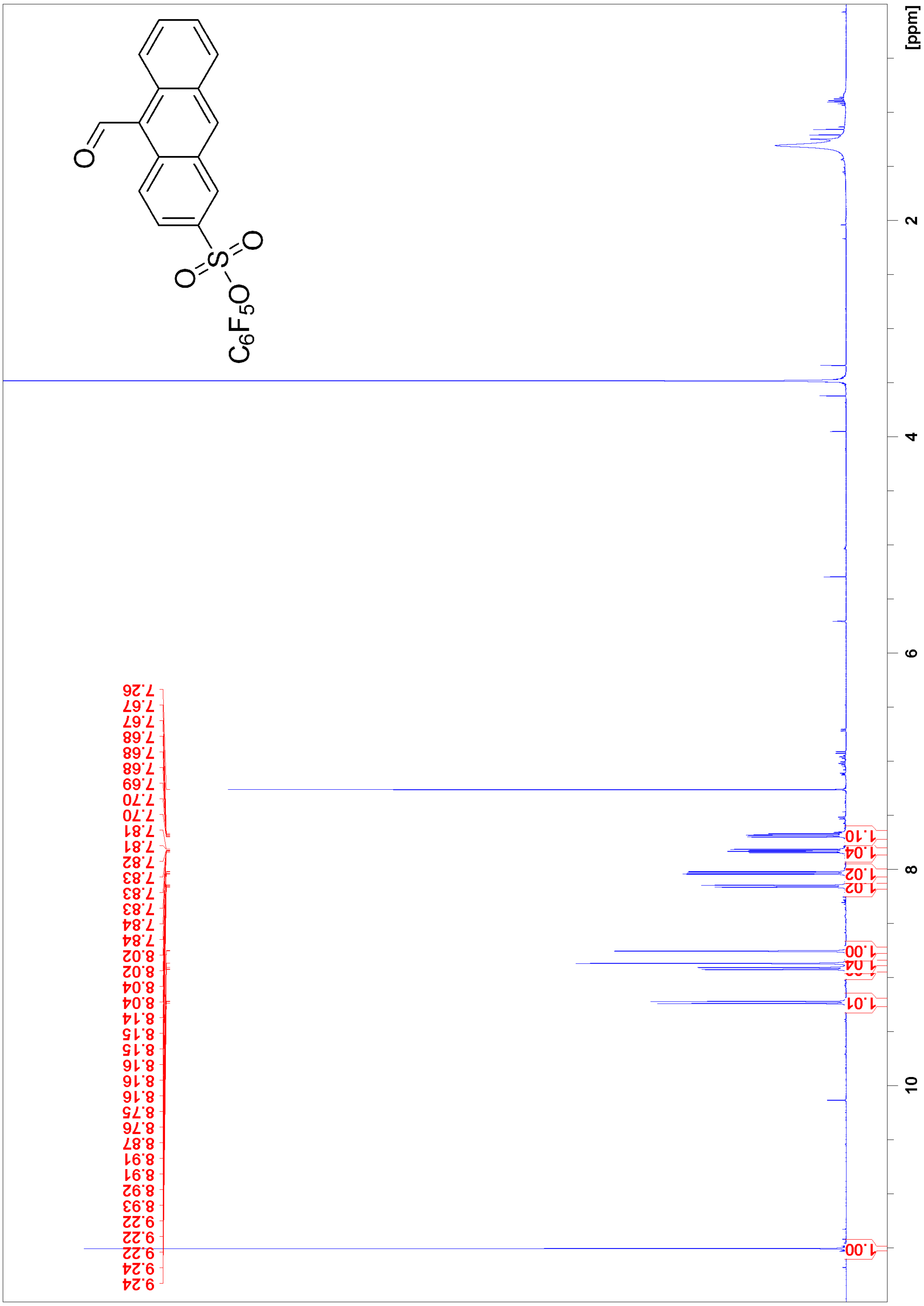

Figure S35. ^{1}H NMR spectrum of receptor **13** (500 MHz, $CDCl_3$).

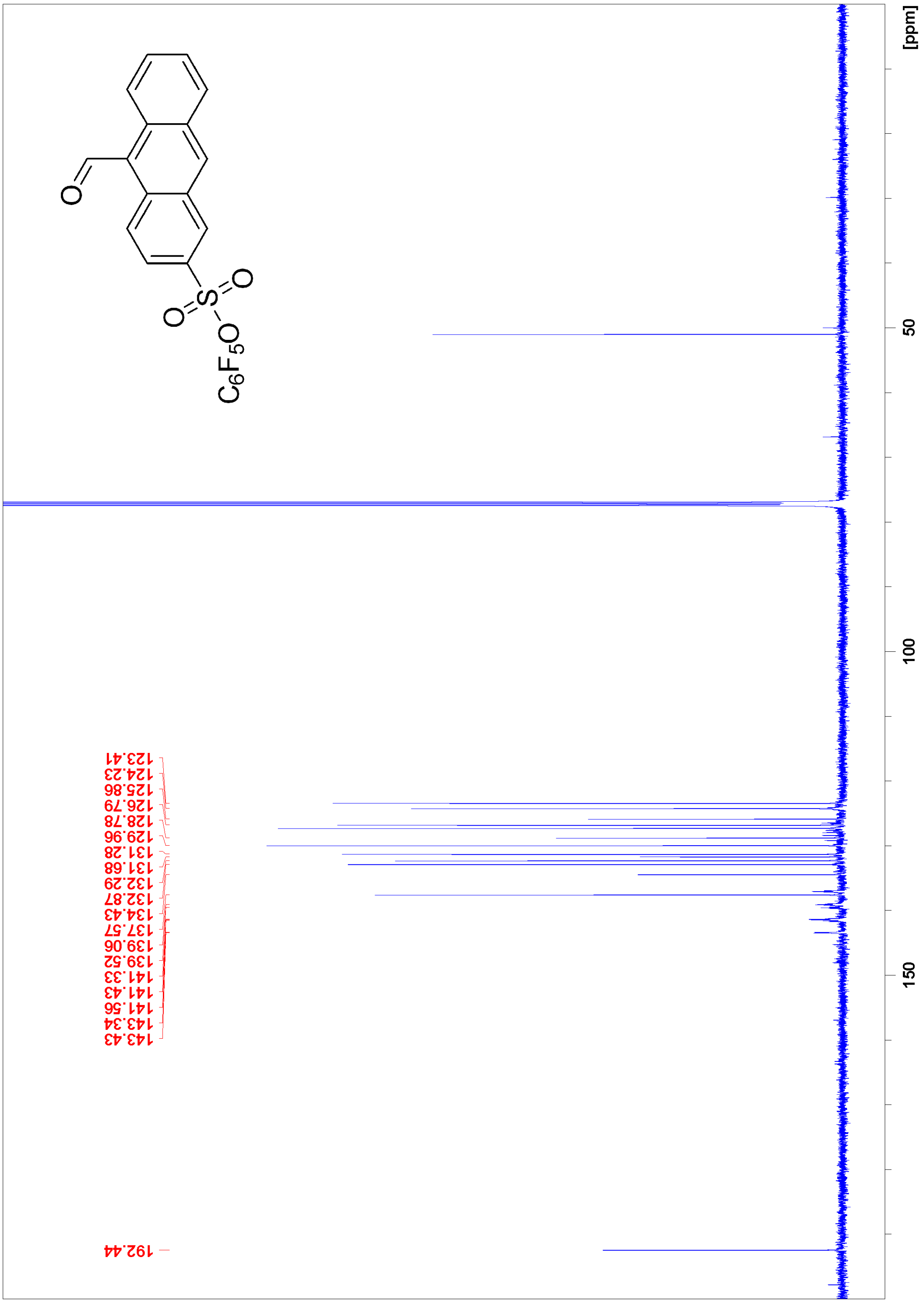

Figure S36. $^{13}C\{^{1}H\}$ NMR spectrum of receptor **13** (125 MHz, $CDCl_3$)

.

Binding studies.

NMR titrations and data analysis. Titrations were performed at 298 K, 500 MHz and 400 MHz in 5 mm NMR tubes using microsyringes, following a previously described technique.[S2] During titrations carried out in D_2O, concentration of the receptor was maintained constant during the titrations with xanthines to avoid changes in ionic strength. 4,4-Dimethyl-4-silapentane-1-sulphonic acid **(**DSS) sodium salt was used as internal reference. Dilution experiments of the free receptors **2** and **5** and of the investigated xanthines were performed to independently measure self-association constants, the most relevant of which were set invariant in the non-linear regression analysis of receptor-xanthines binding data. To avoid any ambiguities in the definition of the equilibrium model in water, where strong self-association constants were present, titration data were simultaneously fitted with dilution data of the reactants through a nonlinear least-square regression analysis, including in the fit all the available signals from both reactants. Receptors ^{1}H assignments were carried out by HSQC and NOESY experiments, whereas for purine, pyrimidine bases and xanthines ^{1}H assignments were obtained from literature.[S3] Mathematical analysis of data and graphic presentation of results was performed using the HypNMR 2006 program.[S4] BC_{50} Calculator, the utility program for computing BC_{50}^0,[11] is available for free at the corresponding author's e-mail address. Results pages and Plots of experimental and calculated shifts are reported hereafter.

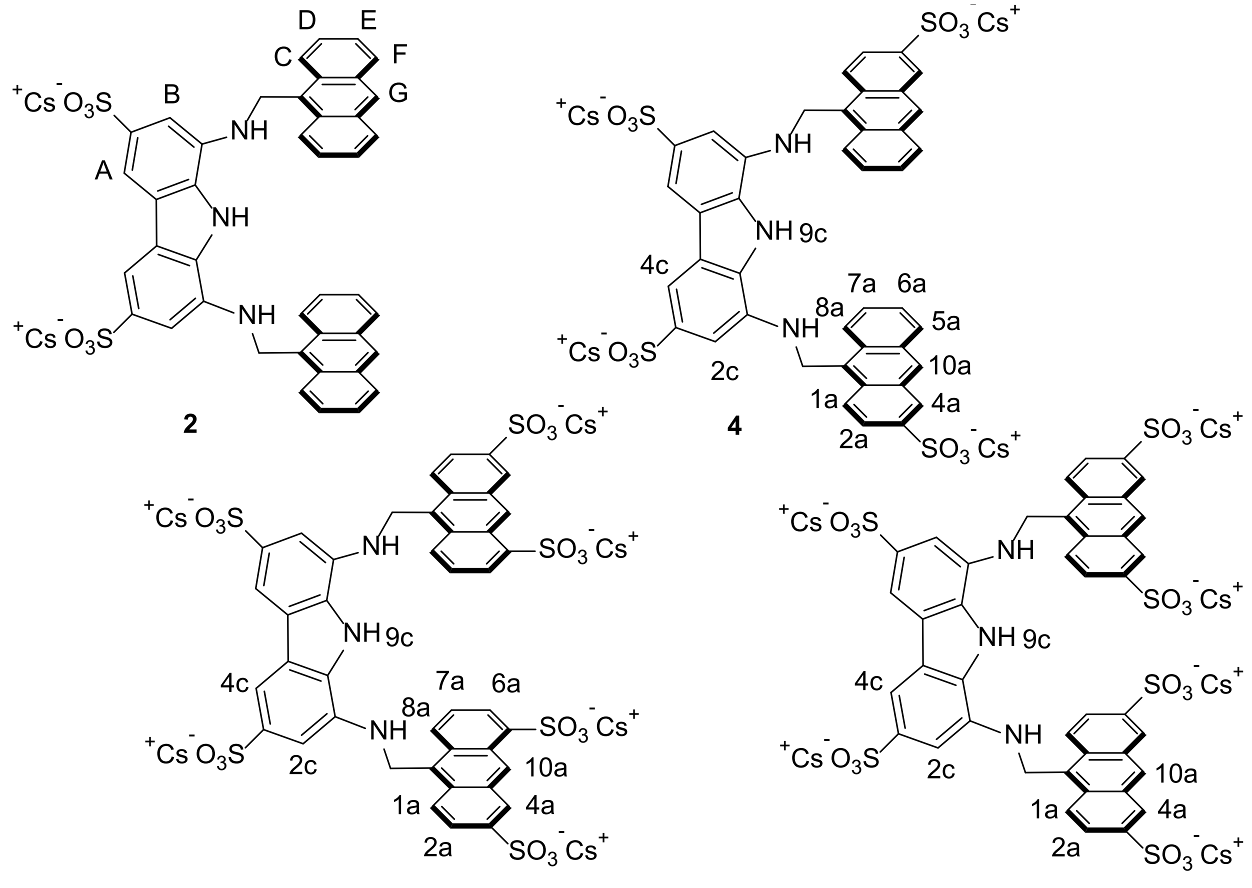

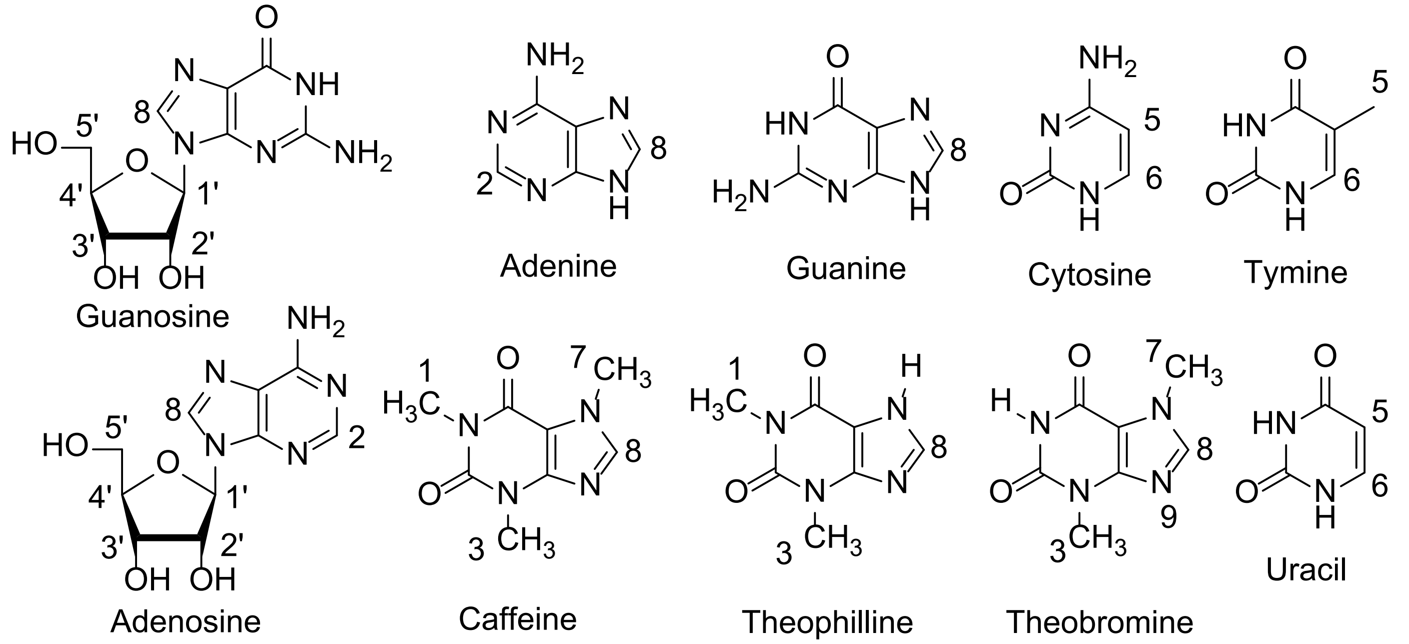

Figure S37. Labelling scheme of ligands and receptors **2**, **4**, **5** and **6** signals monitored in titration experiments.

Dilution of receptor 2 (D_2O, 298 K, 500 MHz).

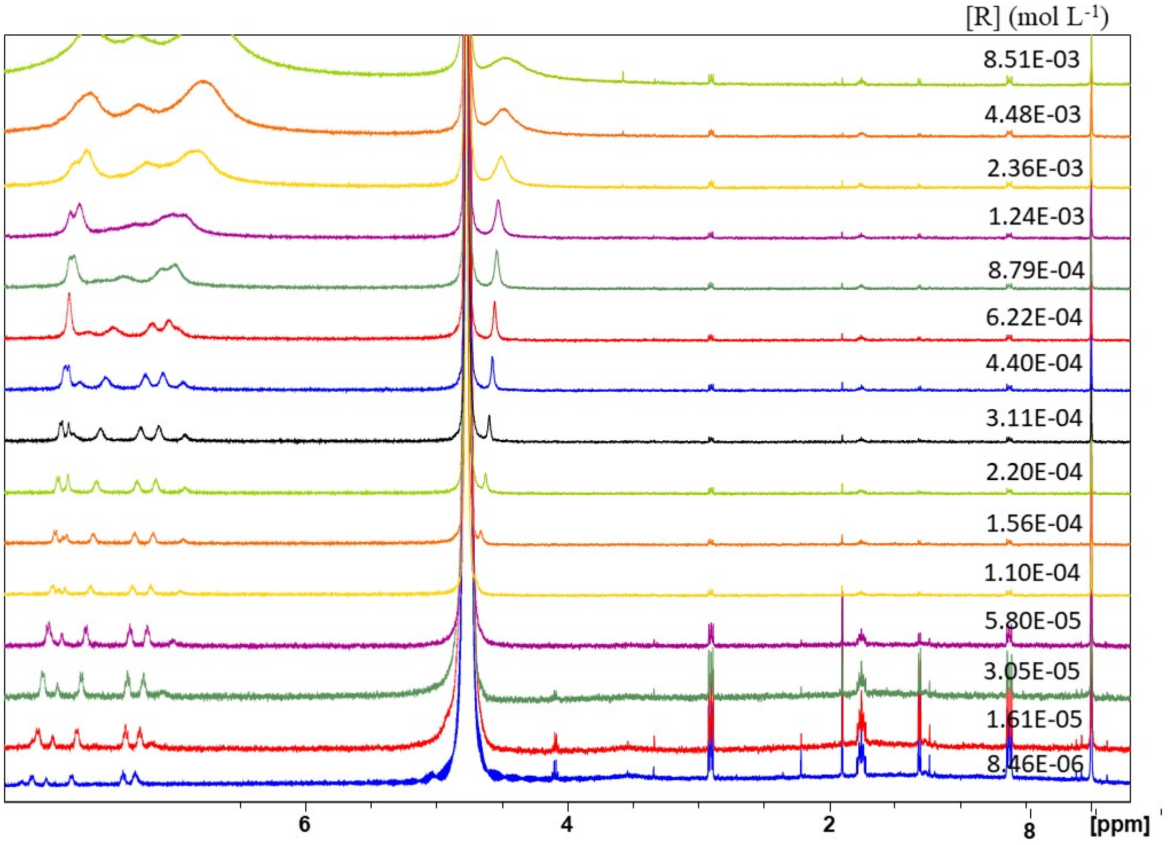

Figure S38. ^{1}H NMR spectroscopic spectra (500 MHz, D_2O, 298 K) of receptor **2** (R) at different concentrations in dilution experiment.

Data Table

R = **2**

δ (ppm) *vs*. [R] (mol L^{-1})

[R]	CH-A R	CH-C R	CH-G R	CH-F R	CH-B R	CH-2 R
8.46E-06	8.1632	8.0885	7.9777	7.7852	7.2708	5.0310
1.61E-05	8.0847	8.0443	7.9303	7.7443	7.1734	-
3.05E-05	-	8.0038	7.8926	7.7109	7.0834	-
5.80E-05	7.9428	7.9674	7.8605	7.6760	7.0085	-
1.10E-04	7.8814	7.9315	7.8348	7.6424	6.9544	-
1.56E-04	7.8475	7.9113	7.8219	7.6203	6.9327	4.6622
2.20E-04	-	7.8883	7.8127	7.5946	6.9192	4.6304
3.11E-04	7.7737	7.8637	7.8076	7.5638	6.9208	4.6022
4.40E-04	7.7216	7.8168	7.8051	7.5234	6.9337	4.5776
6.22E-04	7.6551	7.8037	7.8036	7.4664	6.9710	4.5575
8.79E-04	7.5930	7.7699	7.7986	7.3869	6.9815	4.5415
1.24E-03	7.4853	7.7245	7.7942	7.2984	-	4.5284
2.36E-03	-	7.6675	-	7.2080	-	4.5103
4.48E-03	-	7.6424	-	-	-	4.4940
8.51E-03	-	-	-	-	-	4.4777

Results page

```
 no. of spectra           15
 no. of resonance values   71
 no. of resonant nuclei     6
Chi-squared =  16.32
   sigma =   0.00992627389          RMS weighted residual =   0.00841283107

      stoich          value   relative    log    standard
      coeff                   std devn    beta   deviation

 Beta  2 refined  4.7312E+004  0.2898    4.6750   0.1259  (   R2  )
 Beta  4 refined  1.1478E+012  0.6122   12.0599   0.2659  (   R4  )

 Individual chemical shifts
                  R                       2
 =========================================================
        +        value    error         value    error
CH-A    +       8.3484   0.0392         7.7736   0.0225
CH-C    +       8.2227   0.0300         7.8366   0.0140
CH-G    +       8.0925   0.0292         7.7395   0.0130
CH-F    +       7.8590   0.0212         7.6160   0.0177
CH-B    +       7.5616   0.0629         6.6997   0.0202
CH-2    +       5.3155   0.0587         4.4689   0.0153
        +
                  4
 =================================
        +        value    error
CH-A    +       6.6349   0.1106
CH-C    +       7.3808   0.0316
CH-G    +       7.8609   0.0469
CH-F    +       6.4899   0.0835
CH-B    +       7.6711   0.1268
CH-2    +       4.4572   0.0165

 Correlation coefficients*1000


      1    2
   1
   2  962

 Parameters are numbered as follows
    1 beta 2
    2 beta 4
```

Titration Plots

Chemical shifts (δ, ppm) *vs*. concentration of R (mol L^{-1})

experimental (symbols) and calculated (lines) values

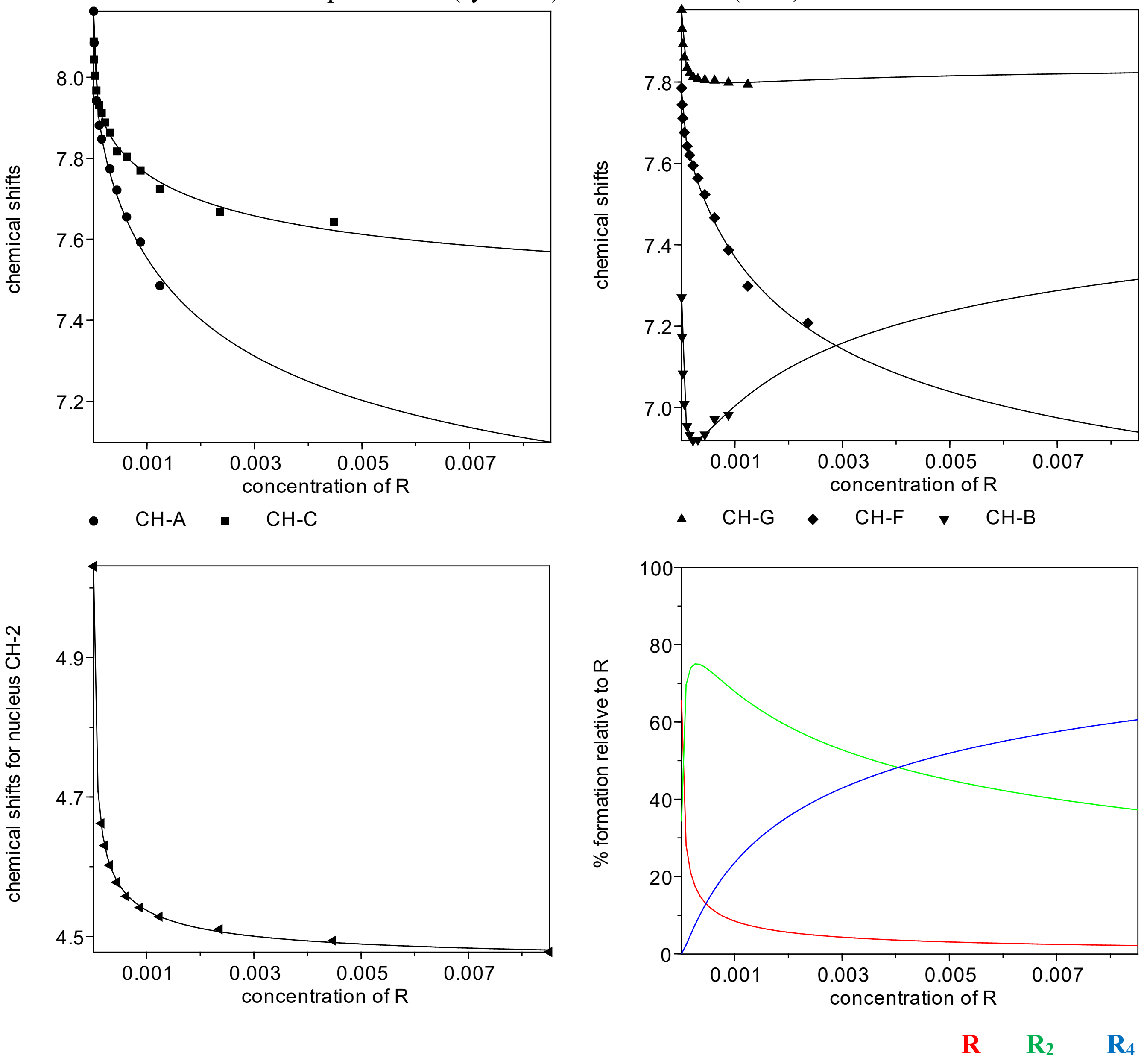

Dilution of Caffeine (D_2O, 298 K, 500 MHz).

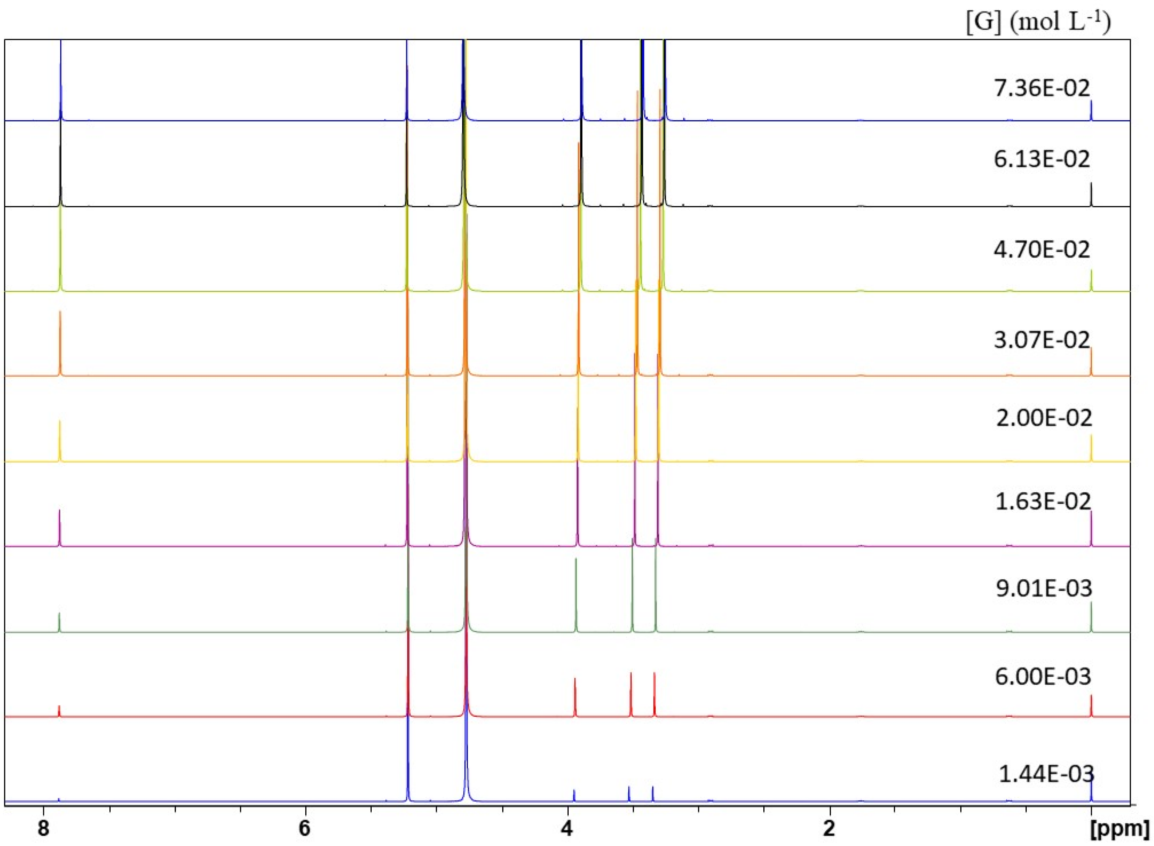

Figure S39. ^{1}H NMR spectroscopic spectra (500 MHz, D_2O, 298 K) of caffeine (G) at different concentrations in dilution experiment.

Data Table

G = Caffeine

δ (ppm) *vs*. [G] (mol L^{-1})

[G]	CH-8 G	CH3-7 G	CH3-3 G	CH3-1 G
1.44E-03	7.8855	3.9478	3.5288	3.3469
6.00E-03	7.8829	3.9396	3.5138	3.3343
9.01E-03	7.8816	3.9351	3.5054	3.3270
1.63E-02	7.8790	3.9259	3.4887	3.3125
2.00E-02	7.8778	3.9218	3.4814	3.3062
3.07E-02	7.8756	3.9125	3.4642	3.2908
4.70E-02	7.8736	3.9019	3.4445	3.2729
6.13E-02	7.8725	3.8948	3.4306	3.2603
7.36E-02	7.8721	3.8900	3.4212	3.2516

Results page

```
 no. of spectra             9
 no. of resonance values   27
 no. of resonant nuclei     3

   sigma =   0.00033040070          RMS weighted residual =   0.00028436364

      stoich          value   relative    log    standard
      coeff                   std devn    beta   deviation

 Beta  2 refined     5.9938    0.0186    0.7777   0.0081  (   0.77770027365219     )

 Individual chemical shifts

        +
                  G                       2
 =========================================================
        +       value    error         value    error
CH3-7   +      3.9502   0.0002        3.7822   0.0016
CH3-3   +      3.5336   0.0003        3.2218   0.0028
CH3-1   +      3.3519   0.0003        3.0751   0.0025
```

Titration Plots

Chemical shifts (δ, ppm) *vs*. concentration of G (mol L^{-1})
experimental (symbols) and calculated (lines) values

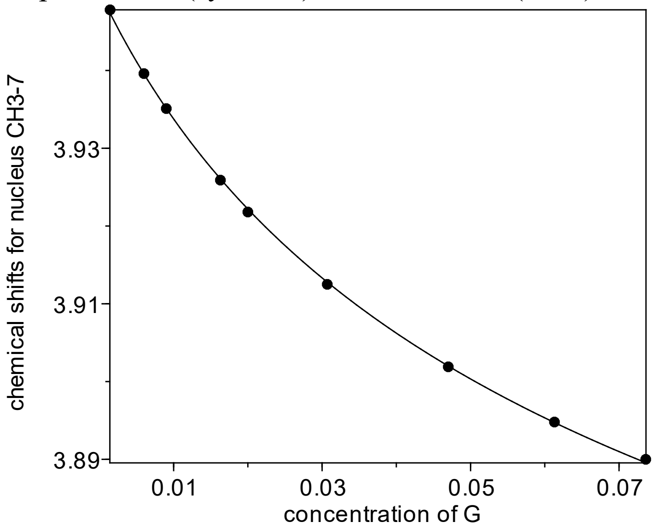

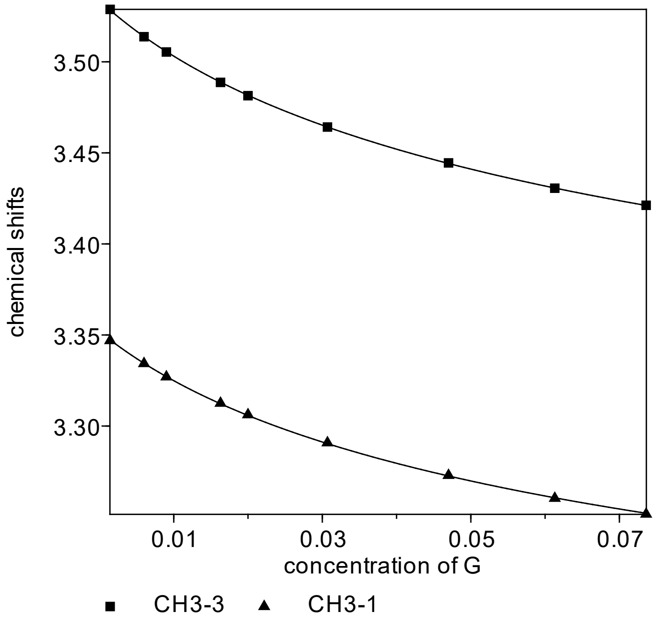

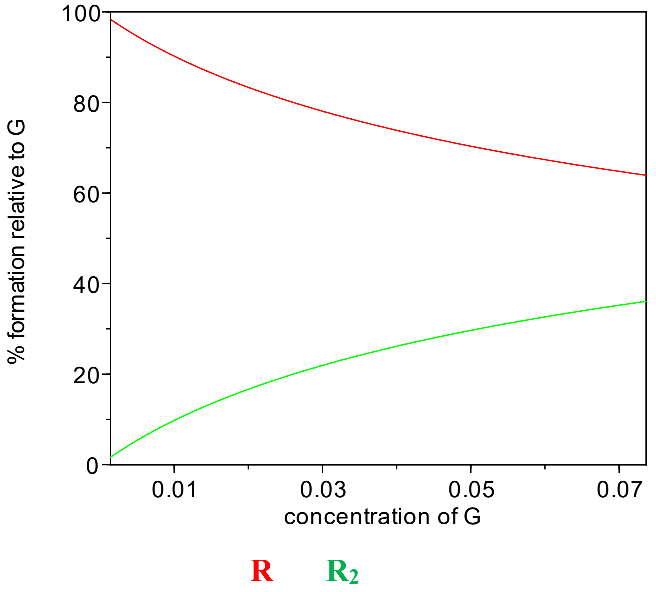

Dilution of Theophylline (D_2O, 298 K, 500 MHz).

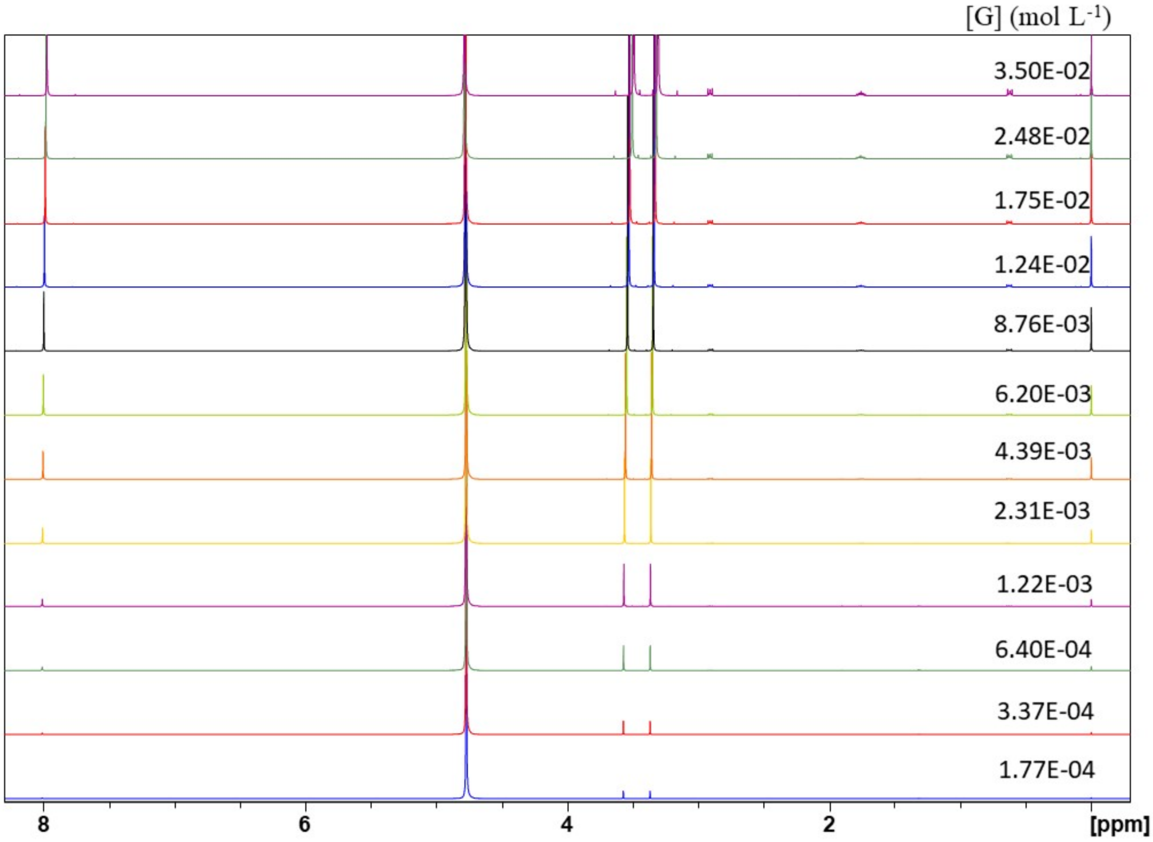

Figure S40. ^{1}H NMR spectroscopic spectra (500 MHz, D_2O, 298 K) of theophylline (G) at different concentrations in dilution experiment.

Data Table

G = Theophylline

δ (ppm) *vs*. [G] (mol L^{-1})

[G]	CH-8 G	CH3-1 G	CH3-3 G
1.77E-04	8.0127	3.5724	3.3685
3.37E-04	8.0128	3.5716	3.3678
6.40E-04	8.0123	3.5702	3.3668
1.22E-03	8.0115	3.5677	3.3649
2.31E-03	8.0098	3.5633	3.3615
4.39E-03	8.0063	3.5556	3.3557
6.20E-03	8.0035	5.5497	3.3513
8.76E-03	7.9996	3.5420	3.3455
1.24E-02	7.9950	3.5330	3.3387
1.75E-02	7.9889	3.5216	3.3300
2.48E-02	7.9815	3.5076	3.3193
3.50E-02	7.9726	3.4911	3.3066

Results page

```
no. of spectra            12
no. of resonance values   36
no. of resonant nuclei     3

  sigma =   0.00049323539          RMS weighted residual =   0.00044269231

     stoich          value   relative    log    standard
     coeff                   std devn    beta   deviation

Beta  2 refined     6.4006    0.0408    0.8062   0.0177  (   G2   )

Individual chemical shifts

                   G                       2
=========================================================
       +        value    error         value    error
CH-8   +       8.0140   0.0002         7.8532   0.0045
CH3-1  +       3.5726   0.0002         3.2488   0.0085
CH3-3  +       3.3686   0.0002         3.1228   0.0066
```

Titration Plots

Chemical shifts (δ, ppm) *vs*. concentration of G (mol L^{-1})

experimental (symbols) and calculated (lines) values

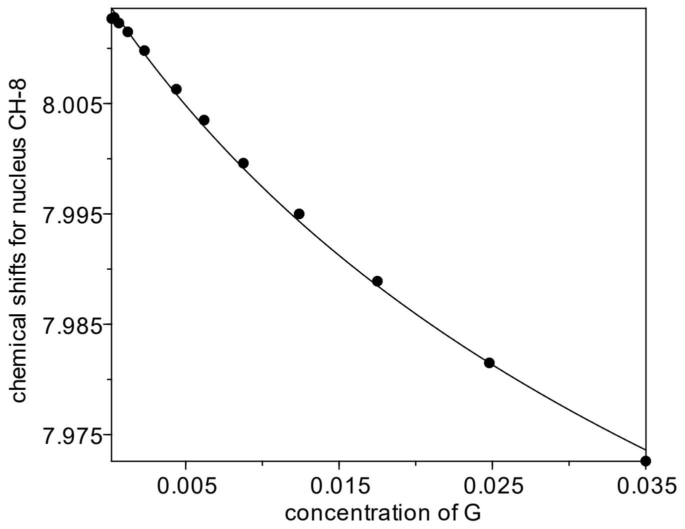

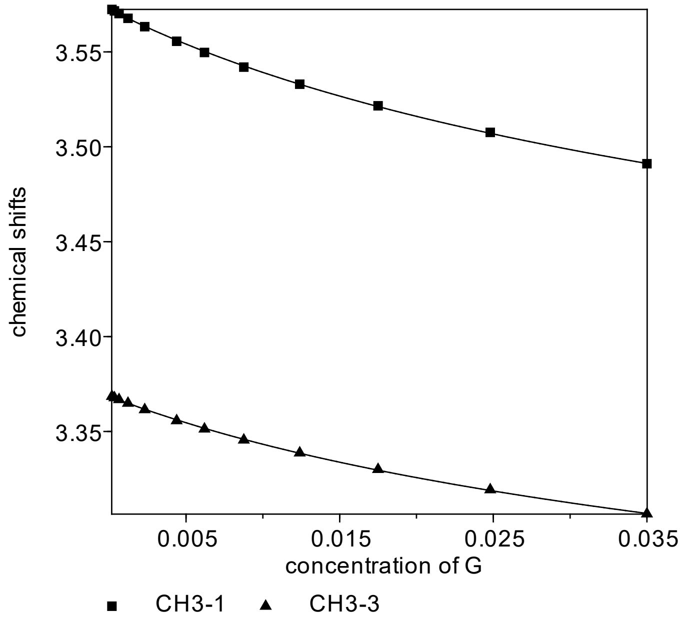

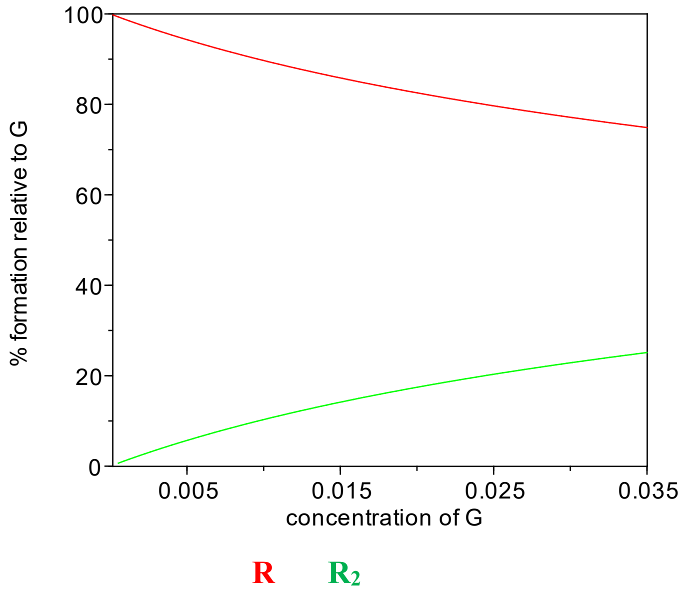

Dilution of Theobromine (D_2O, 298 K, 500 MHz).

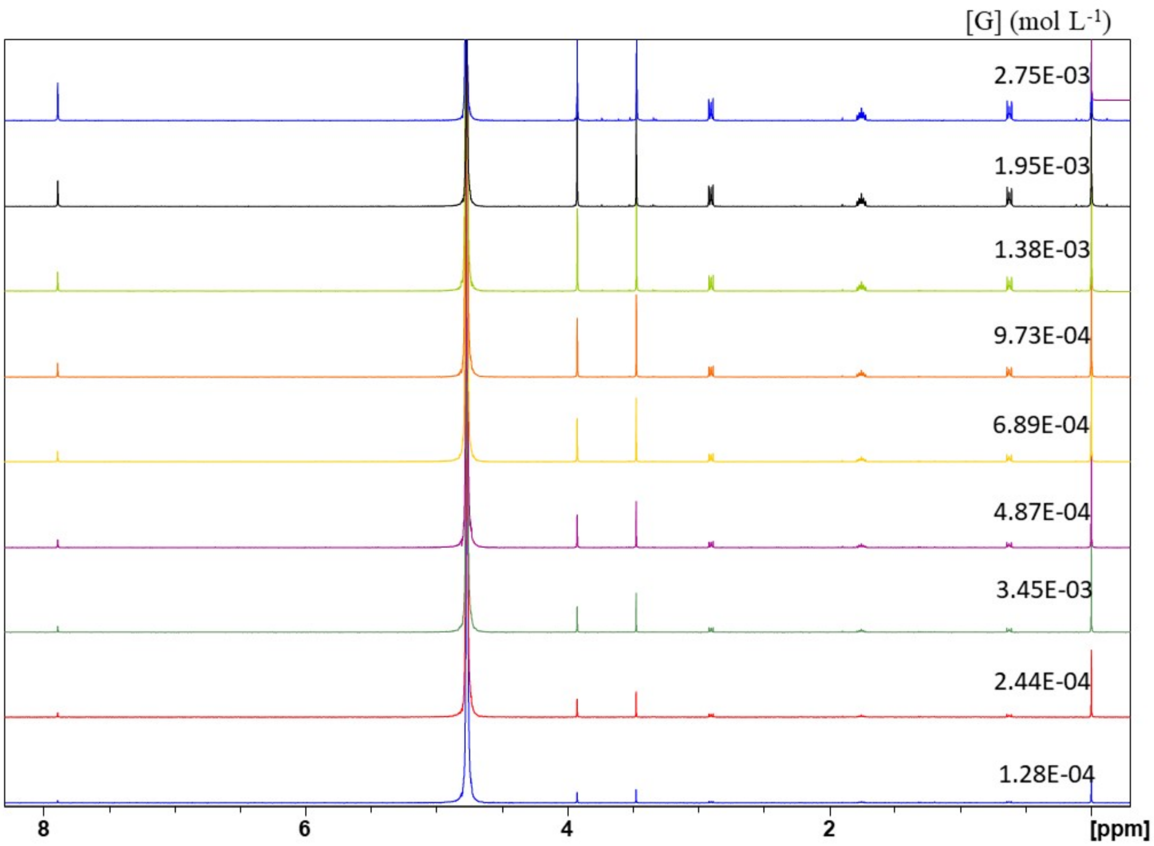

Figure S41. ^{1}H NMR spectroscopic spectra (500 MHz, D_2O, 298 K) of theobromine (G) at different concentrations in dilution experiment.

Data Table

G = Theobromine

δ (ppm) *vs*. [G] (mol L^{-1})

[G]	CH3-7 G	CH3-3 G
1.28E-04	3.9295	3.4791
2.44E-04	3.9293	3.4787
3.45E-04	3.9291	3.4784
4.87E-04	3.9290	3.4781
6.89E-04	3.9290	3.4778
9.73E-04	3.9285	3.4770
1.38E-03	3.9280	3.4761
1.95E-03	3.9275	3.4749
2.75E-03	3.9265	3.4731

Results page

```
 no. of spectra             9
 no. of resonance values   16
 no. of resonant nuclei     2

   sigma =   0.00006805522          RMS weighted residual =   0.00005642841

      stoich          value   relative    log    standard
      coeff                   std devn    beta   deviation

 Beta  2 refined     5.5975    0.7526    0.7480   0.3268  (   G2   )

 Individual chemical shifts

                  G                       2
 =========================================================
        +        value    error         value    error
CH3-7   +       3.9296   0.0000         3.8252   0.0741
CH3-3   +       3.4793   0.0001         3.2667   0.1510
```

Titration Plots

Chemical shifts (δ, ppm) *vs*. concentration of G (mol L^{-1})

experimental (symbols) and calculated (lines) values

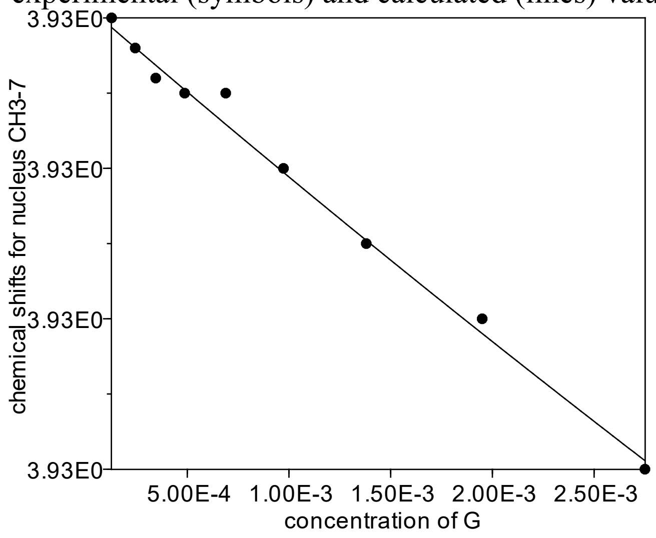

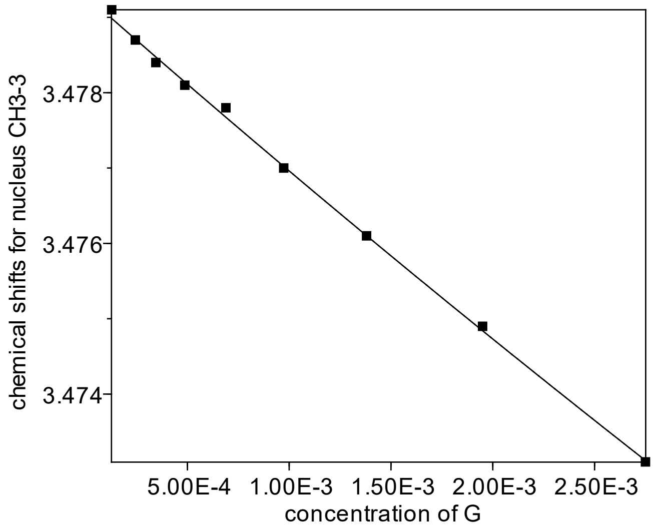

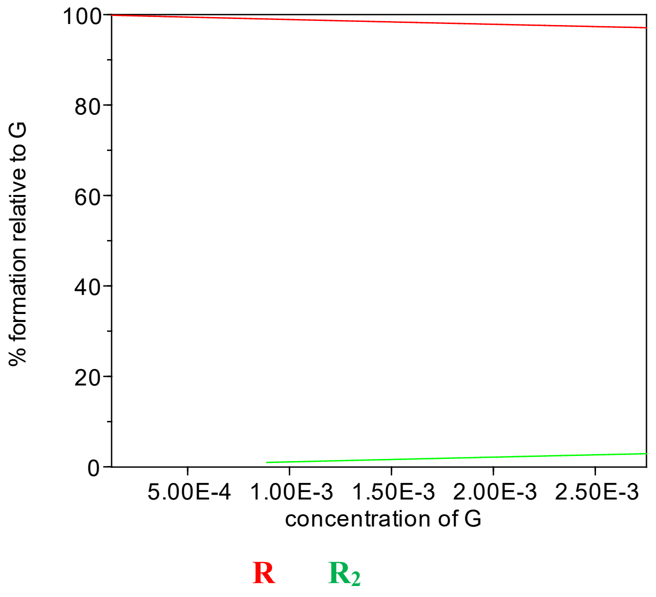

2 + Adenine (D_2O, 298 K, 500 MHz)

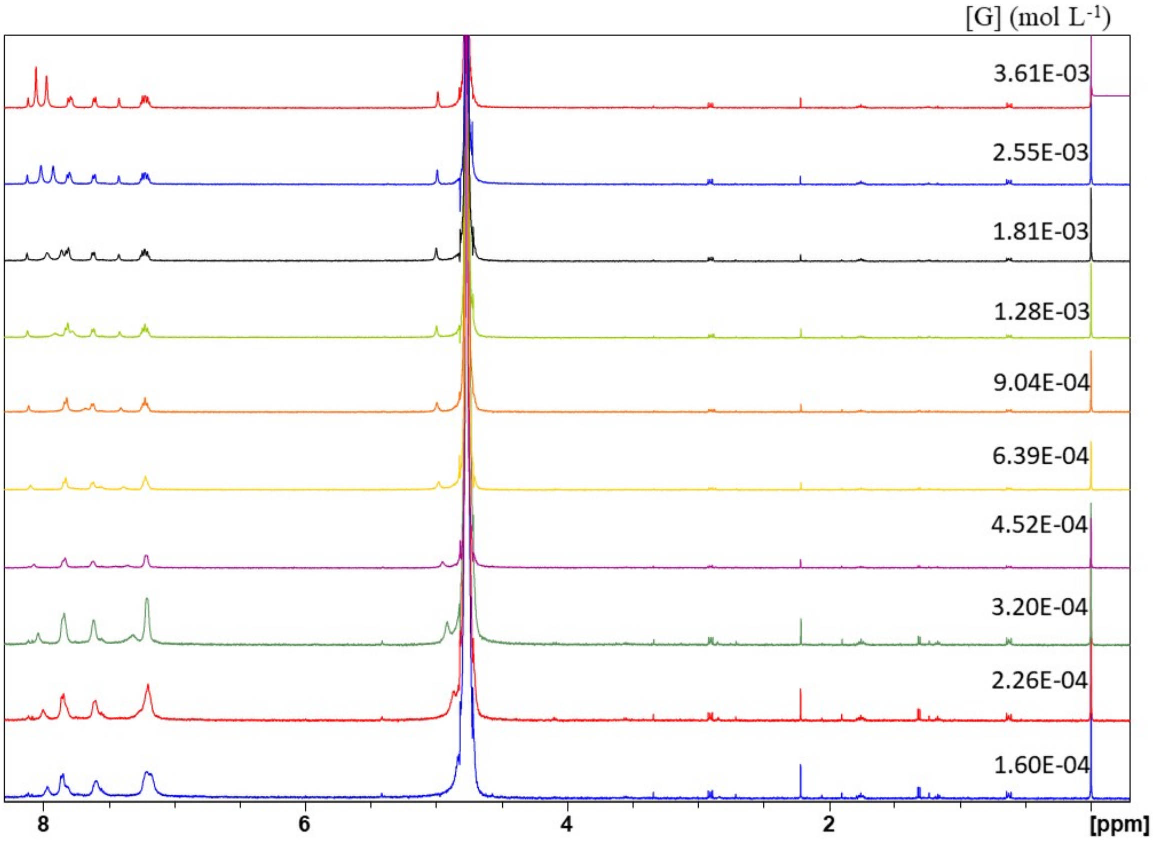

Figure S42. ^{1}H NMR spectroscopic titration (500 MHz, D_2O, 298 K) of receptor **2** (3.69 10^{-4} mol L^{-1}) with incremental concentrations of adenine (G).

Data Table

R = **2** G = Adenine

δ (ppm) *vs*. [G] (mol L^{-1})

Titration [R] = 3.69 10^{-4} mol L^{-1}

[G]	CH-2 G	CH-8 G	CH-A R	CH-B R	CH2 R
1.60E-04	-	-	7.9709	-	4.8338
2.26E-04	-	-	8.0027	7.2664	4.8680
3.20E-04	-	-	8.0416	7.3173	4.9177
4.52E-04	-	7.4555	8.0750	7.3599	4.9544
6.39E-04	-	7.5685	8.0982	7.3905	4.9795
9.04E-04	7.8290	7.6802	8.1134	7.4078	4.9922
1.28E-03	7.9110	7.7797	8.1229	7.4188	4.9982
1.81E-03	7.9725	7.8630	8.1266	7.4251	5.0005
2.55E-03	8.0201	7.9267	8.1243	7.4258	4.9955
3.61E-03	8.0581	7.9769	8.1178	7.4236	4.9868

δ (ppm) *vs*. [R] (mol L^{-1})

Receptor 2 dilution experiment

[R]	CH-A R	CH-B R
8.46E-06	8.1632	7.2708
1.61E-05	8.0847	7.1734
3.05E-05	-	7.0834
5.80E-05	7.9428	7.0085
1.10E-04	7.8814	6.9544
1.56E-04	7.8475	6.9327
2.20E-04	-	6.9192
3.11E-04	7.7737	6.9208
4.40E-04	7.7216	6.9337
6.22E-04	7.6551	6.9710
8.79E-04	7.5930	6.9815
1.24E-03	7.4853	-
2.36E-03	-	-
4.48E-03	-	-
8.51E-03	-	-

Results page

```
 no. of spectra            25
 no. of resonance values   73
 no. of resonant nuclei     5

Chi-squared =  27.49

   sigma =   0.00637302438          RMS weighted residual =   0.00527435250

      stoich            value   relative    log    standard
      coeff                     std devn    beta   deviation

 Beta  1  2 refined  1.6837E+008  0.2610    8.2263   0.1134  (   GR2  )
 Beta  1  4 refined  3.4126E+016  0.3682   16.5331   0.1599  (   GR4  )
 Beta  0  2 constant 4.7315E+004            4.6750           (   R2   )
 Beta  0  4 constant 1.1479E+012           12.0599           (   R4   )

 Individual chemical shifts

                  G                       R
 =========================================================
       +       value    error         value    error
CH-2   +      8.1541   0.0274
CH-8   +      8.1361   0.0192
CH-A   +                              8.3481   0.0103
CH-B   +                              7.5613   0.0103
CH2    +                              5.3158   0.0109
       +
                 1,2                     1,4
 =========================================================
       +       value    error         value    error
CH-2   +      6.0852   0.7057         2.8600   3.4268
CH-8   +      4.4538   0.5646         5.7798   1.1709
CH-A   +      8.0971   0.0152         8.5173   0.1548
CH-B   +      7.3877   0.0267         8.2280   0.2935
CH2    +      4.9533   0.0223         5.5938   0.2257
       +
                 0,2                     0,4
 =========================================================
       +       value    error         value    error
CH-2   +
CH-8   +
CH-A   +      7.7740   0.0076         6.6343   0.0289
CH-B   +      6.7001   0.0079         7.6703   0.0383
CH2    +      4.4685   0.0053         4.4577   0.0093

 Correlation coefficients*1000

      1    2
   1
   2 -843

 Parameters are numbered as follows
    1 beta 1,2
    2 beta 1,4
```

Titration Plots

Chemical shifts (δ, ppm) *vs*. point number

experimental (symbols) and calculated (lines) values

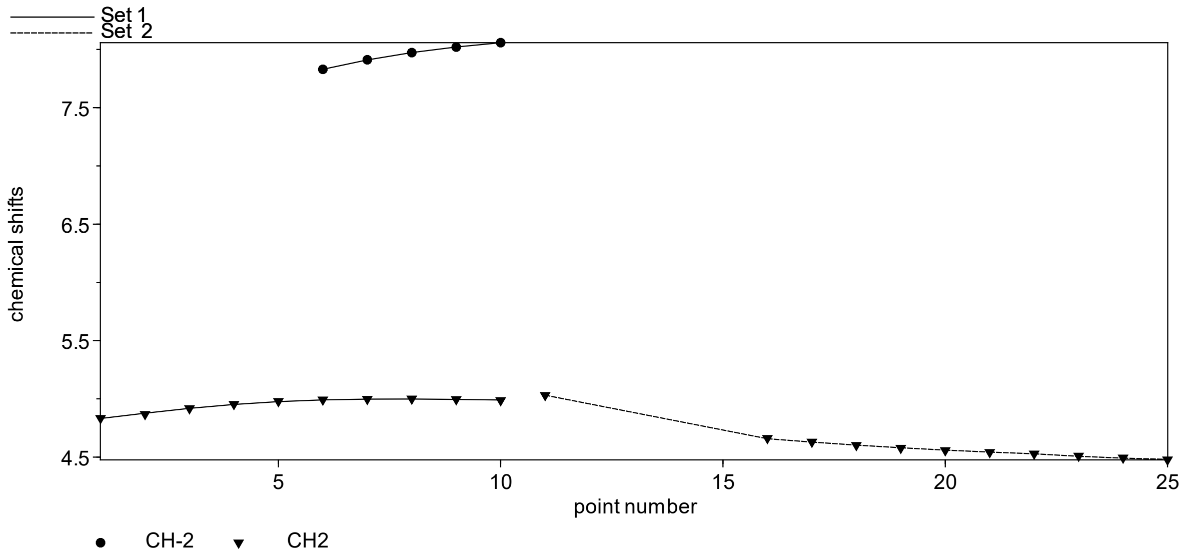

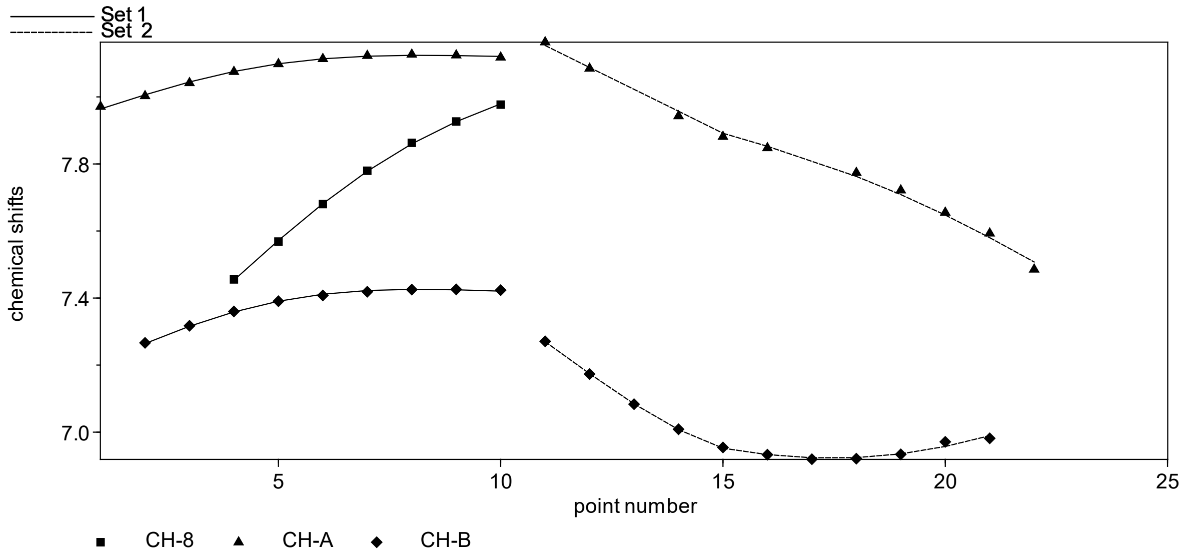

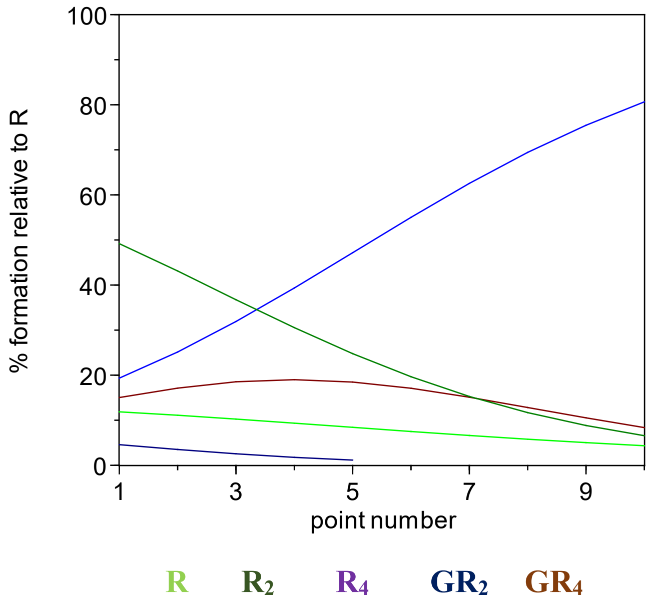

2 + Adenosine (D_2O, 298 K, 500 MHz)

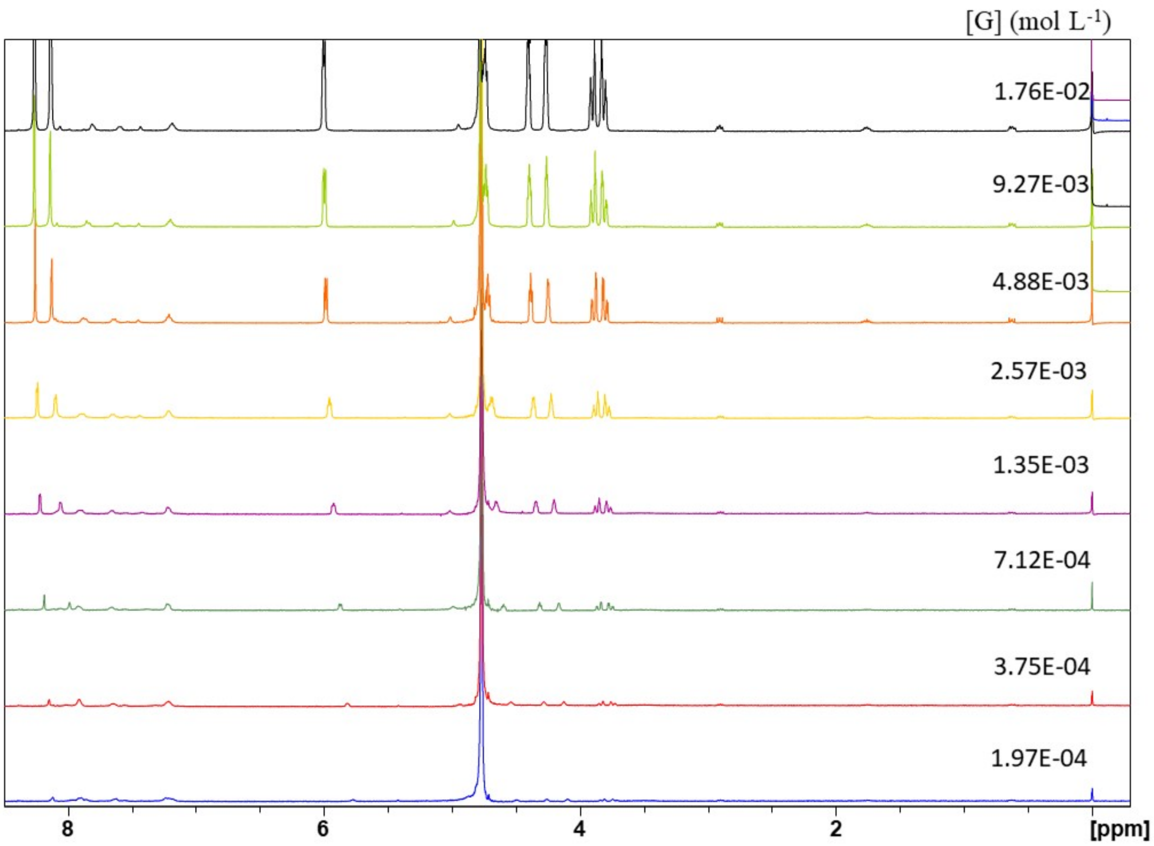

Figure S43. ^{1}H NMR spectroscopic titration (500 MHz, D_2O, 298 K) of receptor **2** (3.43 10^{-4} mol L^{-1}) with incremental concentrations of adenosine (G).

Data Table

R = **2**   G = Adenosine

δ (ppm) *vs*. [G] (mol L^{-1})

Titration [R] = 3.43 10^{-4} mol L^{-1}

[G]	CH-2 G	CH-8 G	CH-1' G
1.97E-04	8.1228	7.8626	5.7771
3.75E-04	8.1518	7.9172	5.8203
7.12E-04	8.1892	7.9917	5.8770
1.35E-03	8.2233	8.0619	5.9299
2.57E-03	8.2439	8.1025	5.9640
4.88E-03	8.2607	8.1324	5.9880
9.27E-03	8.2675	8.1435	6.0011
1.76E-02	8.2683	8.1394	6.0072

Results page

```
 no. of spectra             8
 no. of resonance values   24
 no. of resonant nuclei     3

Chi-squared =   6.00

   sigma =   0.00432098298          RMS weighted residual =   0.00363664908

       stoich            value   relative    log    standard
       coeff                     std devn    beta   deviation

 Beta  1  2 refined  1.9842E+008  0.0871    8.2976   0.0378  (   GR2  )
 Beta  0  2 constant 4.7315E+004            4.6750           (   R2   )

 Individual chemical shifts

                    G                      1,2
 =========================================================
        +       value    error         value    error
CH-2    +      8.2761   0.0025        7.7279   0.0279
CH-8    +      8.1608   0.0028        7.0956   0.0487
CH-1'   +      6.0146   0.0027        5.1582   0.0401
```

Titration Plots

Chemical shifts (δ, ppm) *vs*. concentration of G (mol L^{-1})

experimental (symbols) and calculated (lines) values

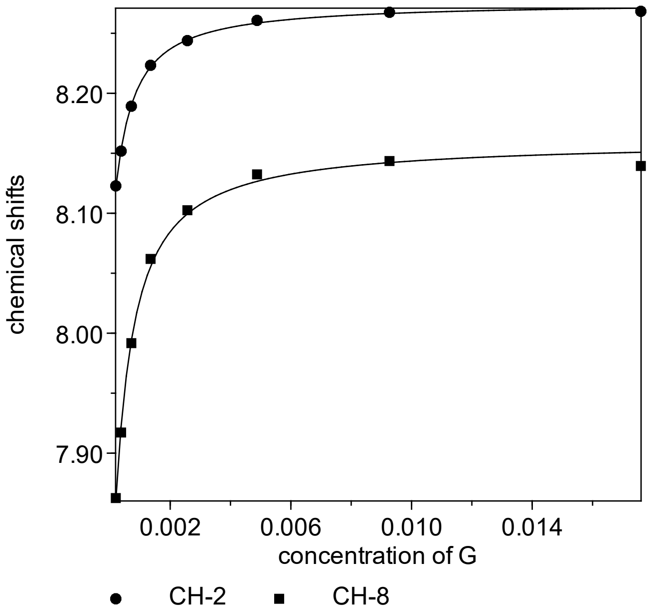

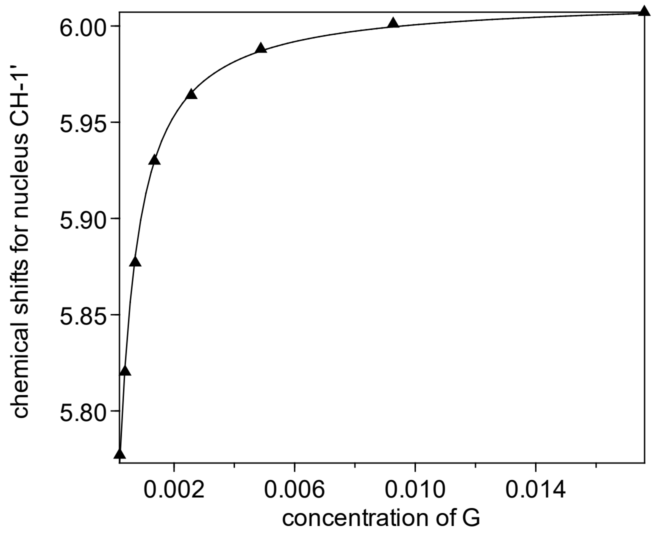

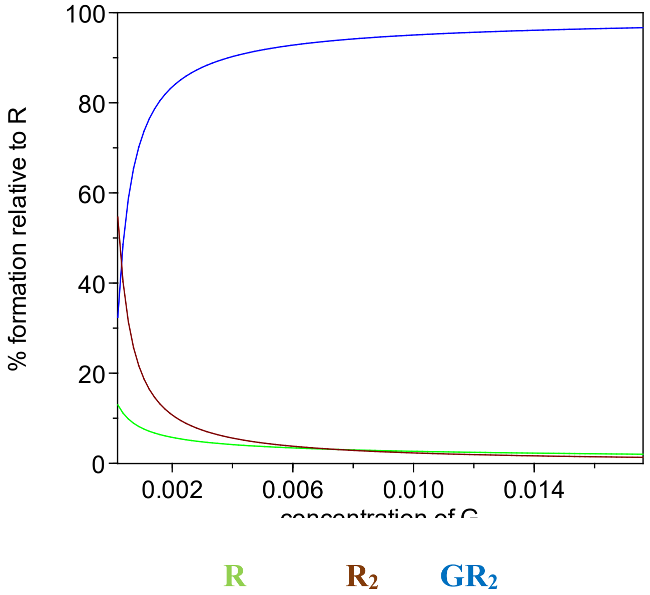

2 + Guanosine (D_2O, 298 K, 500 MHz)

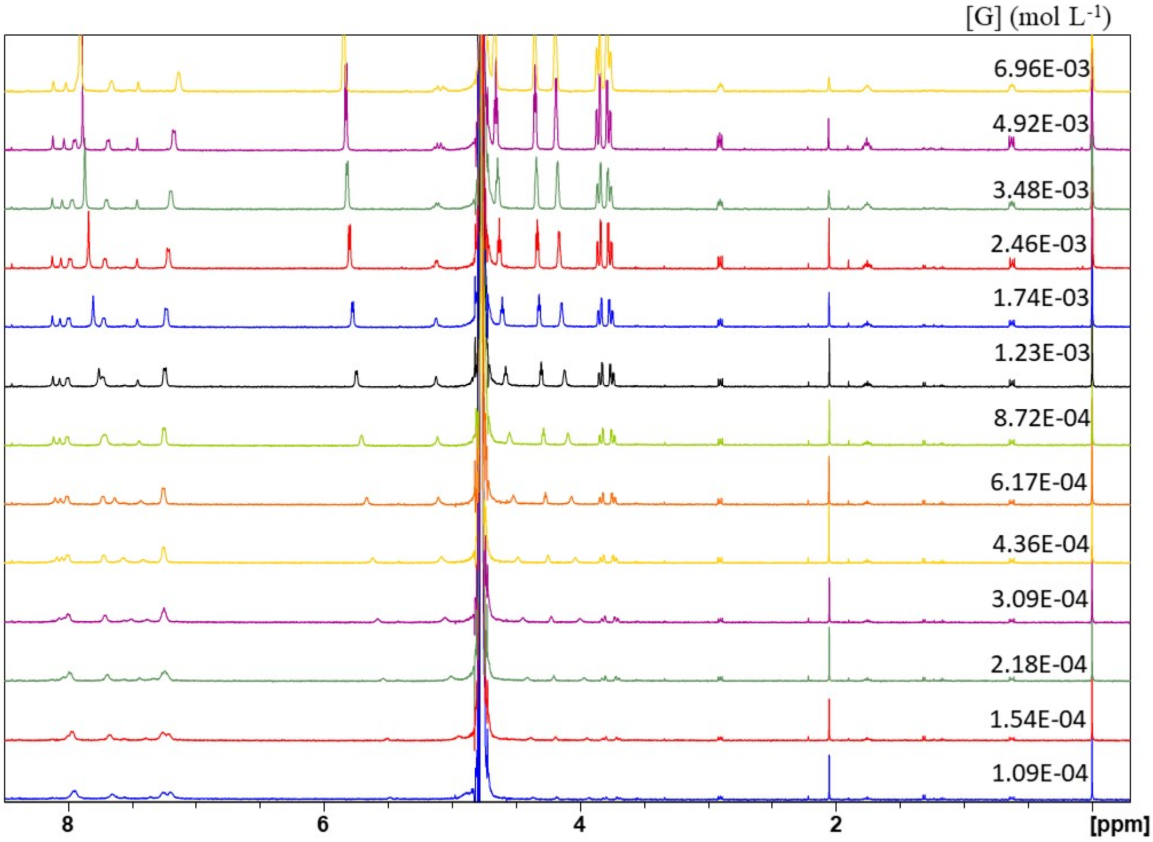

Figure S44. ^{1}H NMR spectroscopic titration (500 MHz, D_2O, 298 K) of receptor **2** (3.34 10^{-4} mol L^{-1}) with incremental concentrations of guanosine (G).

Data Table

R = **2** G = Guanosine

δ (ppm) *vs*. [G] (mol L^{-1})

Titration [R] = 3.34 10^{-4} mol L^{-1}

[G]	CH-8 G	CH-1' G	CH-2' G	CH-3' G	CH-4' G	CH-A R	CH-C R	CH-F R
1.09E-04	7.3614	5.4853	4.3665	4.1822	3.9321	-	7.9551	7.6567
1.54E-04	7.3972	5.5080	4.3878	4.1931	3.9482	8.0004	7.9719	7.6784
2.18E-04	7.4431	5.5395	4.4122	4.2072	3.9700	8.0389	7.9883	7.6982
3.09E-04	7.5100	5.5836	4.4516	4.2295	4.0047	8.0727	8.0014	7.7142
4.36E-04	7.5733	5.6270	4.4853	4.2492	4.0359	8.0911	8.0067	7.7256
6.17E-04	7.6375	5.6682	4.5196	4.2689	4.0660	8.1049	8.0107	7.7303
8.72E-04	-	5.7132	4.5567	4.2901	4.0996	8.1159	8.0117	-
1.23E-03	7.7616	5.7493	4.5865	4.3081	4.1256	8.1213	8.0069	7.7313
1.74E-03	7.8068	5.7794	4.6102	4.3222	4.1470	8.1255	7.9994	7.7231
2.46E-03	7.8434	5.8025	4.6299	4.3335	4.1633	8.1269	7.9867	7.7148
3.48E-03	7.8720	5.8218	4.6474	4.3436	4.1791	8.1264	7.9733	7.7024
4.92E-03	7.8917	5.8340	4.6575	4.3510	4.1875	8.1246	7.9554	7.6882
6.96E-03	7.9108	5.8463	4.6688	4.3583	4.1966	8.1182	7.9345	7.6658

Results page

```
no. of spectra             13
no. of resonance values  101
no. of resonant nuclei     8

  sigma =   0.00306670461          RMS weighted residual =   0.00274633663

      stoich            value   relative    log    standard
      coeff                     std devn    beta   deviation

Beta  1  2 refined  2.1817E+008  0.0255    8.3388   0.0111  (   GR2  )
Beta  0  2 constant 4.7315E+004            4.6750           (   R2   )

Individual chemical shifts

                   G                       R
=========================================================
       +       value    error         value    error
CH-8    +       7.9489   0.0020
CH-1'   +       5.8724   0.0018
CH-2'   +       4.6890   0.0017
CH-3'   +       4.3683   0.0016
CH-4'   +       4.2157   0.0017
CH-A    +                              10.2792   0.1532
CH-C    +                              11.6591   0.1334
CH-F    +                              11.3890   0.1440
       +
                 1,2                        0,2
=========================================================
       +       value    error         value    error
CH-8    +       6.1627   0.0263
CH-1'   +       4.6971   0.0181
CH-2'   +       3.7141   0.0155
CH-3'   +       3.8008   0.0106
CH-4'   +       3.3535   0.0141
CH-A    +       8.0664   0.0058         7.4535   0.0349
CH-C    +       7.8441   0.0055         7.1710   0.0297
CH-F    +       7.5765   0.0058         6.8585   0.0319
```

Titration Plots

Chemical shifts (δ, ppm) *vs*. concentration of G (mol L^{-1})

experimental (symbols) and calculated (lines) values

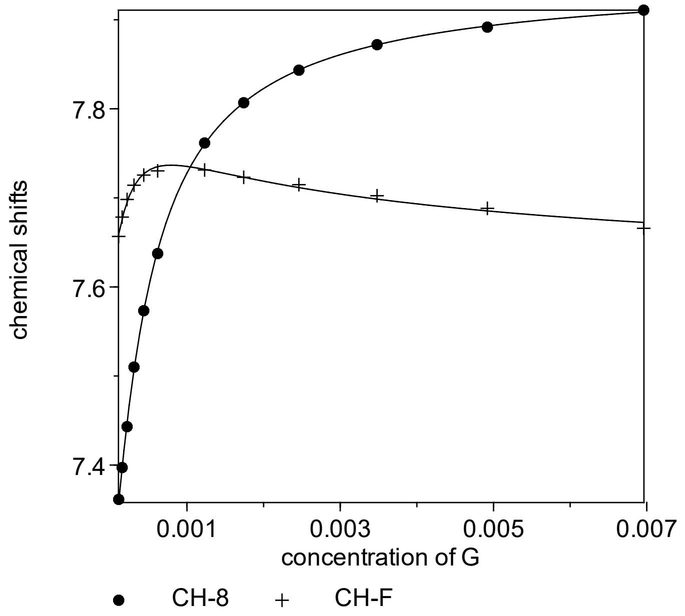

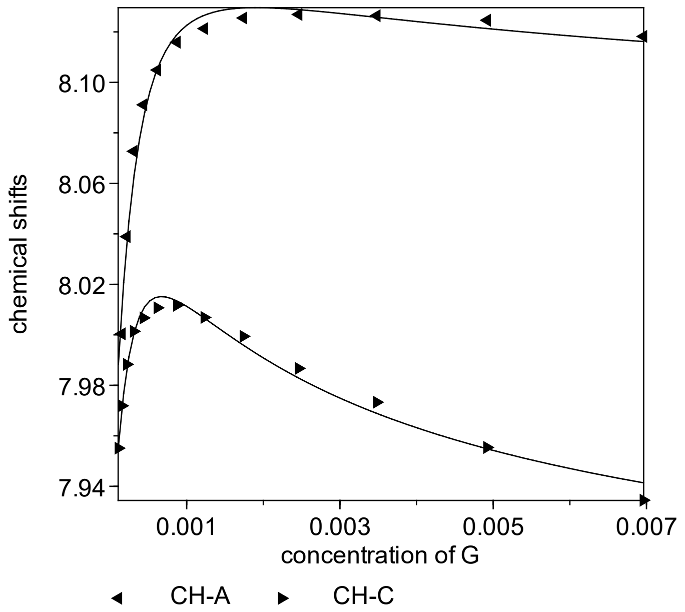

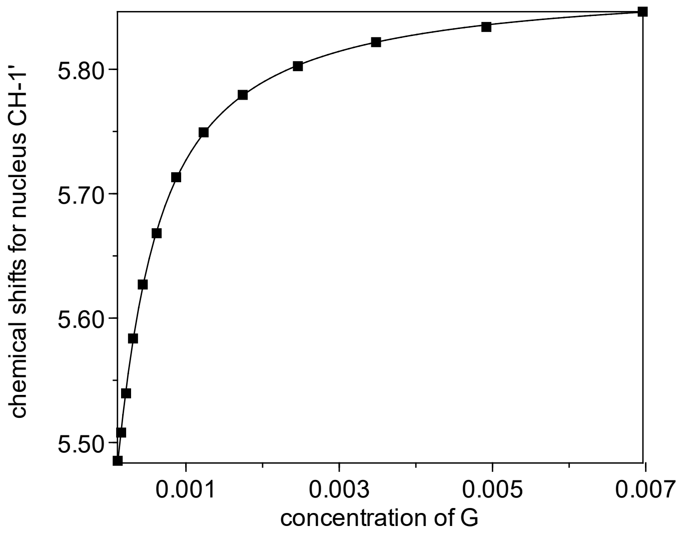

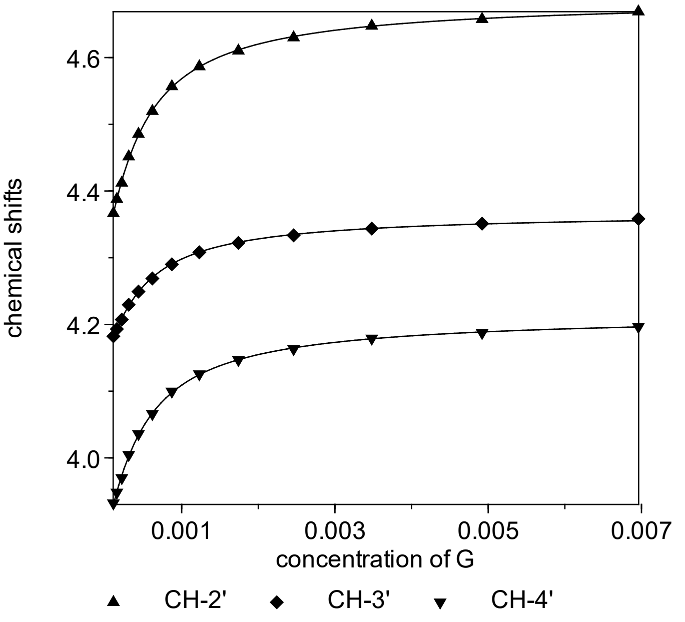

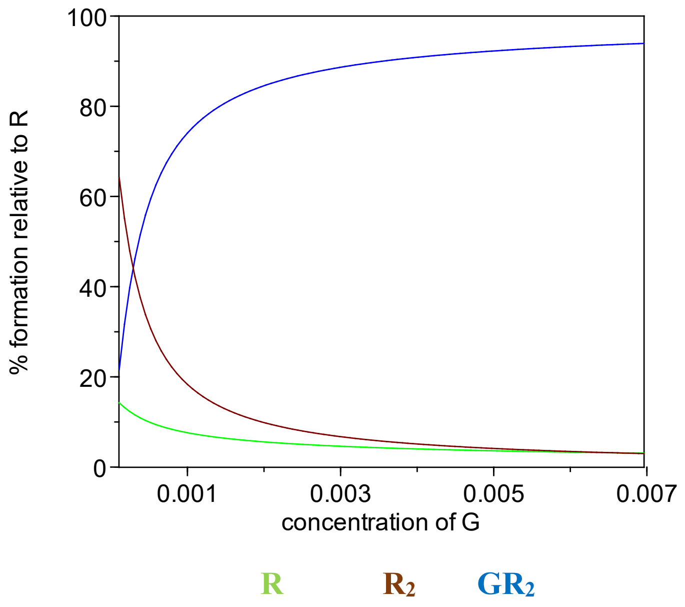

100
80
60
40
20
0
% formation relative to R
0.001
0.003
0.005
0.007
concentration of G
R
R2
GR2

2 + Thymine (D_2O, 298 K, 500 MHz)

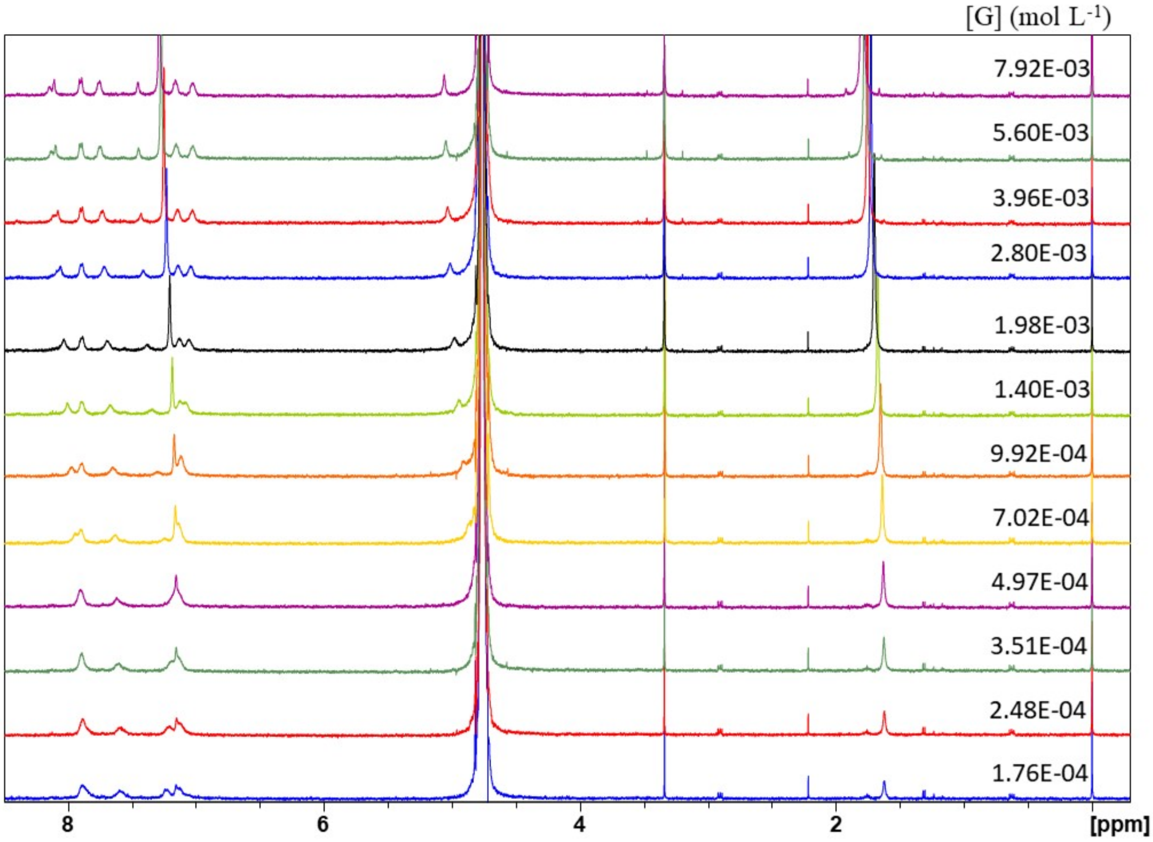

Figure S45. ^{1}H NMR spectroscopic titration (500 MHz, D_2O, 298 K) of receptor **2** (3.42 10^{-4} mol L^{-1}) with incremental concentrations of guanosine (G).

Data Table

R = **2**   G = Thymine

δ (ppm) *vs*. [G] (mol L^{-1})

Titration [R] = 3.42 10^{-4} mol L^{-1}

[G]	CH-6<br>G	CH3-5<br>G	CH-A<br>R	CH-B<br>R	CH2<br>R
1.76E-04	7.1681	1.6243	-	-	-
2.48E-04	7.1571	1.6226	-	-	-
3.51E-04	7.1576	1.6259	-	-	-
4.97E-04	7.1595	1.6301	-	-	-
7.02E-04	7.1657	1.6406	7.9480	7.2501	4.8700
9.92E-04	7.1743	1.6542	7.9754	7.3014	4.9122
1.40E-03	7.1907	1.6752	8.0075	7.3480	4.9526
1.98E-03	7.2101	1.7002	8.0353	7.3867	4.9867
2.80E-03	7.2334	1.7289	8.0634	7.4155	5.0153
3.96E-03	7.2545	1.7542	8.0830	7.4347	5.0380
5.60E-03	7.2735	1.7769	8.0995	7.4496	5.0539
7.92E-03	7.2875	1.7931	8.1125	7.4566	5.0635

δ (ppm) *vs*. [R] (mol L^{-1})

Receptor 2 dilution experiment

[R]	CH-A R	CH-B R	CH2 R
8.46E-06	8.1632	7.2708	5.0310
1.61E-05	8.0847	7.1734	-
3.05E-05	-	7.0834	-
5.80E-05	7.9428	7.0085	-
1.10E-04	7.8814	6.9544	-
1.56E-04	7.8475	6.9327	4.6622
2.20E-04	-	6.9192	4.6304
3.11E-04	7.7737	6.9208	4.6022
4.40E-04	7.7216	6.9337	4.5776
6.22E-04	7.6551	6.9710	4.5575
8.79E-04	7.5930	6.9815	4.5415
1.24E-03	7.4853	-	4.5284
2.36E-03	-	-	4.5103
4.48E-03	-	-	4.4940
8.51E-03	-	-	4.4777

Results page

```
 no. of spectra            28
 no. of resonance values   79
 no. of resonant nuclei     5

Chi-squared =  37.56

   sigma =   0.00504895226          RMS weighted residual =   0.00425090826

      stoich            value   relative    log    standard
      coeff                     std devn    beta   deviation

 Beta  0  2 constant 5.5157E+004           4.7416           (   R2  )
 Beta  0  4 constant 1.1479E+012          12.0599           (   R4  )
 Beta  1  2 refined  6.3304E+007  0.2192    7.8014   0.0952  (   GR2 )
 Beta  1  4 refined  5.9034E+015  0.5972   15.7711   0.2594  (   GR4 )

 Individual chemical shifts

                  G                        R
 ==========================================================
       +       value    error         value    error
CH-6   +      7.3479   0.0125
CH3-5  +      1.8649   0.0142
CH-A   +                              8.3768   0.0086
CH-B   +                              7.5908   0.0086
CH2    +                              5.3485   0.0090
       +
                 0,2                      0,4
 ==========================================================
       +       value    error         value    error
CH-6   +
CH3-5  +
CH-A   +      7.7711   0.0058         6.4095   0.0276
CH-B   +      6.7126   0.0060         7.8306   0.0371
CH2    +      4.4723   0.0039         4.4511   0.0082
       +
                 1,2                      1,4
 ==========================================================
       +       value    error         value    error
CH-6   +      3.7677   0.8479        17.3571   5.8500
CH3-5  +     -2.3270   0.9811        12.5840   6.1797
CH-A   +      8.1421   0.0102         8.0018   0.1699
CH-B   +      7.4718   0.0139         8.6297   0.6771
CH2    +      5.0839   0.0093         5.6367   0.3480

 Correlation coefficients*1000

      1    2
  1
  2 -709

 Parameters are numbered as follows
   1 beta 1,2
   2 beta 1,4
```

Titration Plots

Chemical shifts (δ, ppm) *vs*. concentration of G (mol L^{-1})

experimental (symbols) and calculated (lines) values

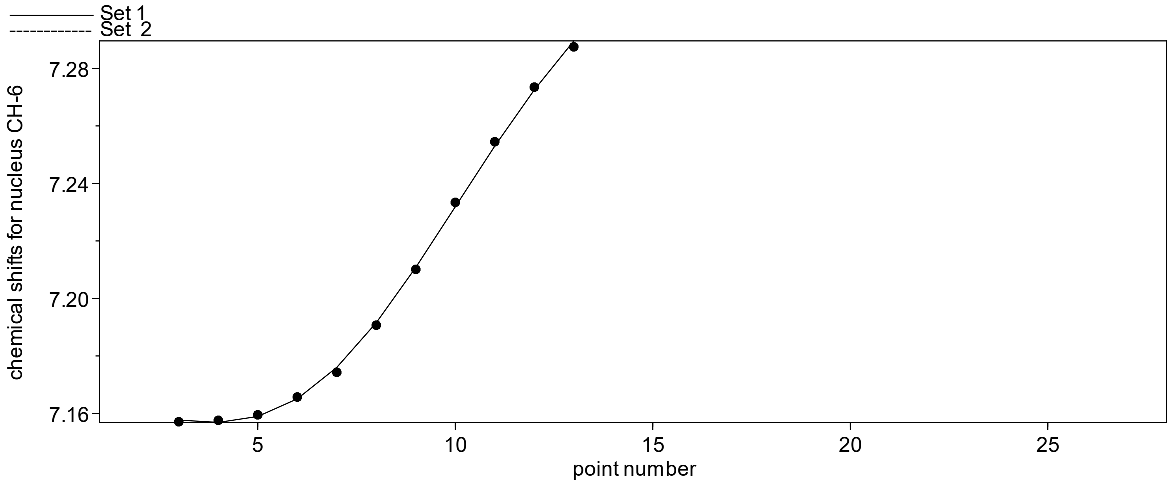

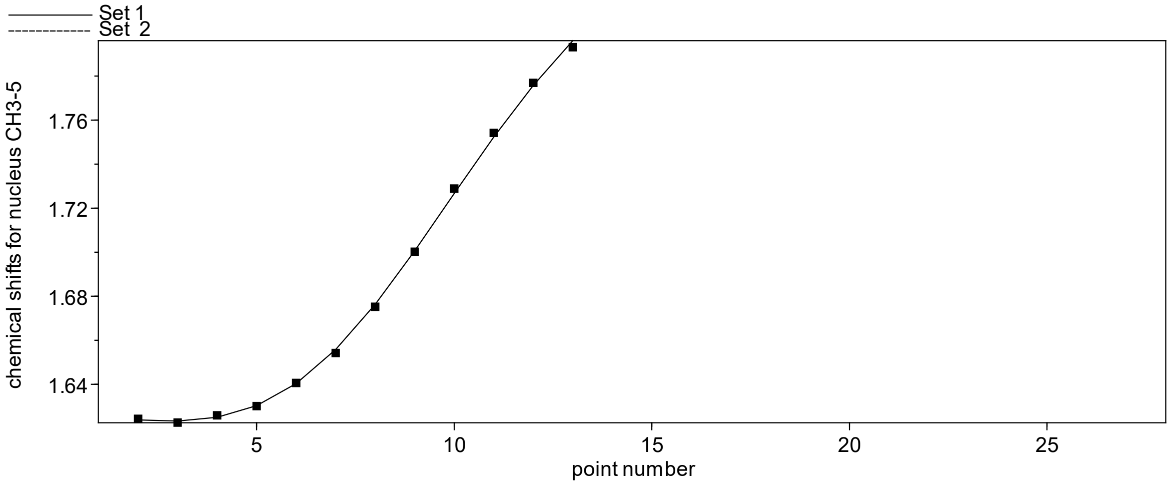

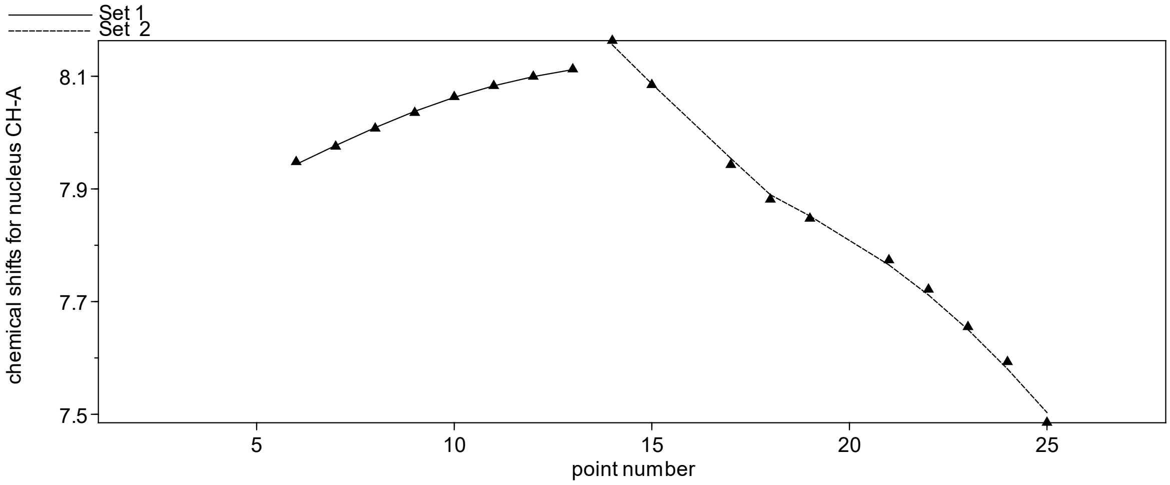

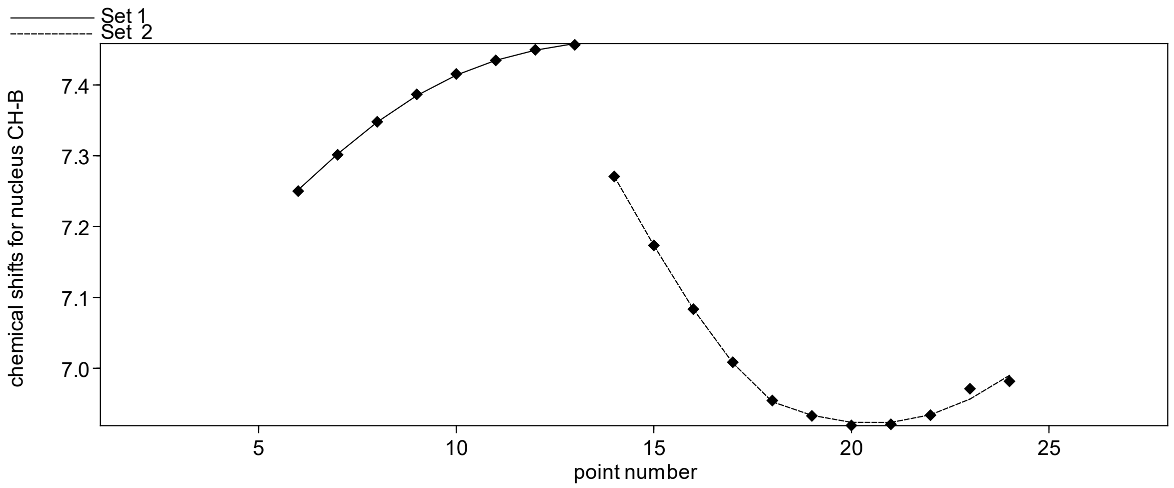

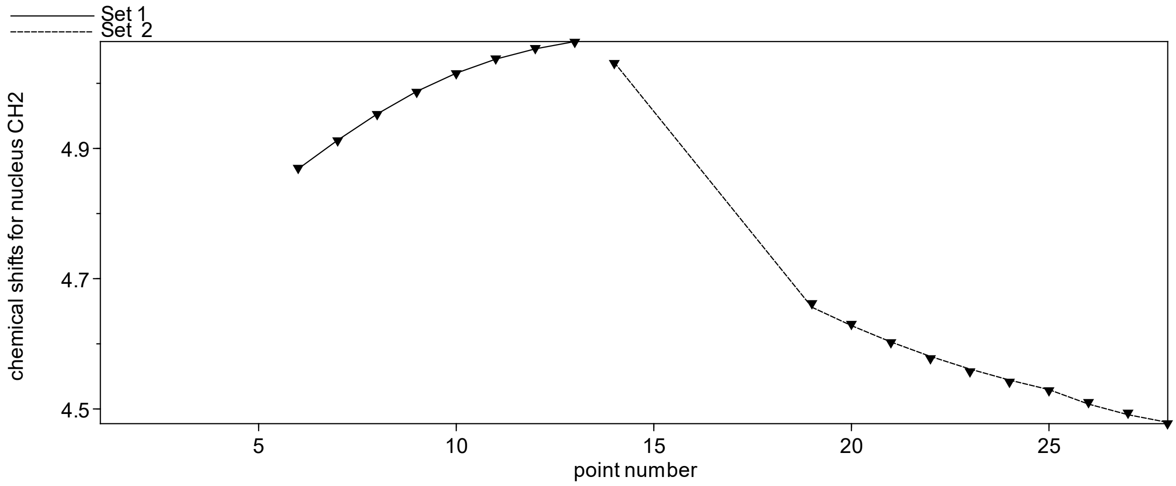

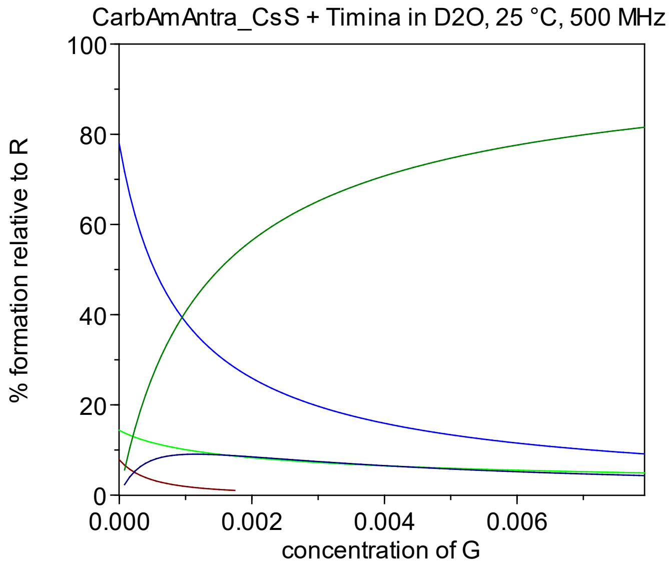

R R_2 R_4 GR_2 GR_4

2 + Cytosine (D_2O, 298 K, 500 MHz)

Data Table

R = **2**   G = Cytosine

Titration [R] = 0.369 mM

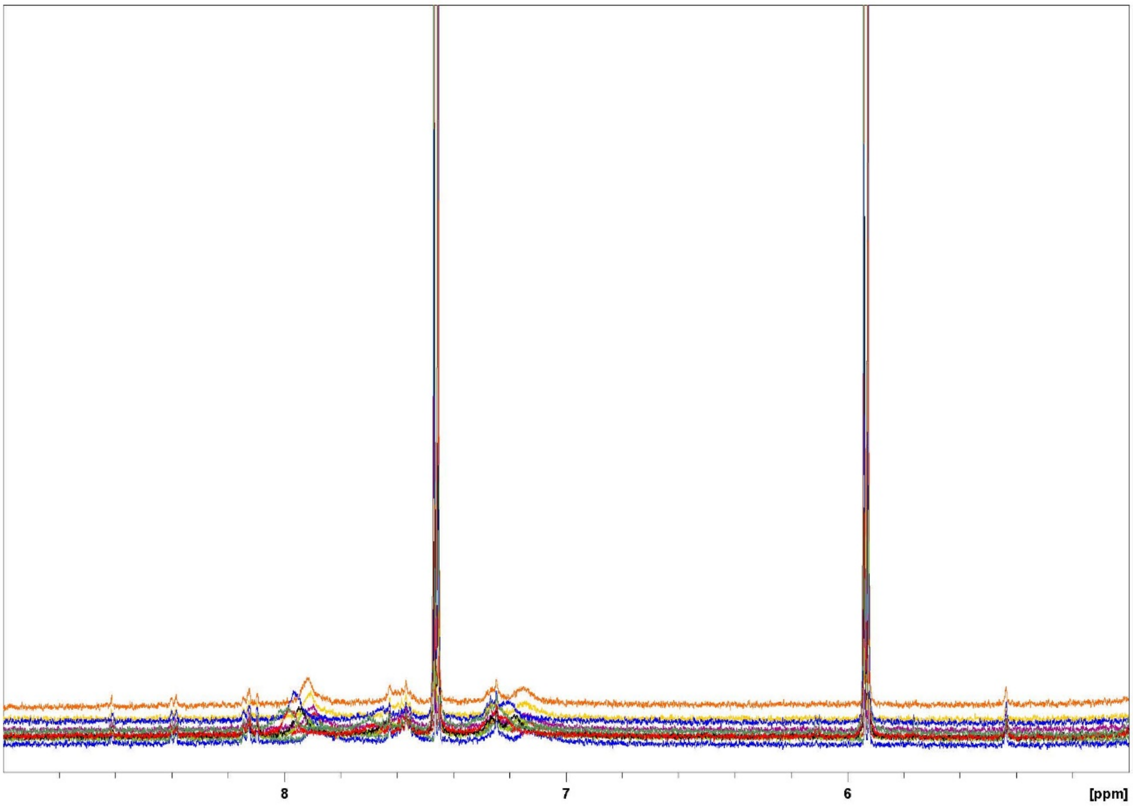

Figure S46. Superposition of ^{1}H NMR spectra registered at incremental concentrations of Cytosine (0.311 mM, 0.439 mM, 0.621 mM, 0.877 mM, 1.24 mM, 1.75 mM, 2.48 mM, 3.50 mM, 4.95 mM, 7.00 mM, 9.90 mM) in a 0.369 mM solution of **2**.

2 + Uracil (D_2O, 298 K, 500 MHz)

Data Table

R = **2**   G = Uracil

Titration [R] = 0.342 mM

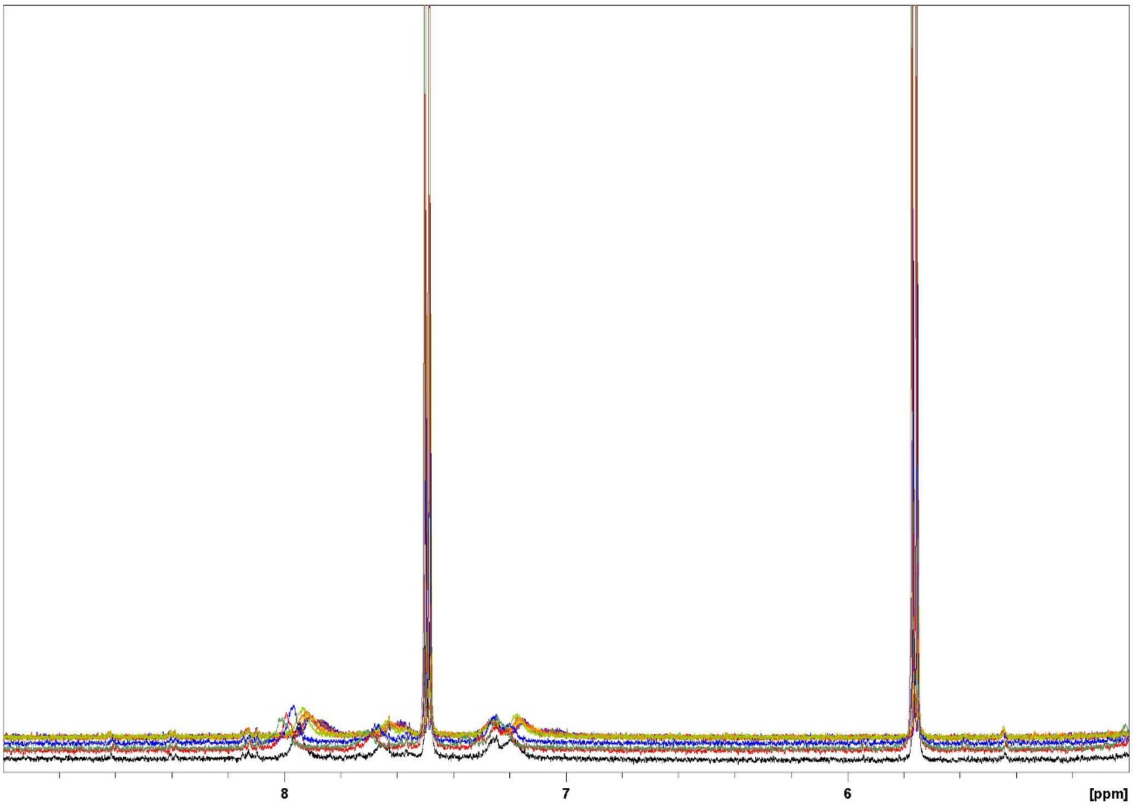

Figure S47. Superposition of ^{1}H NMR spectra registered at incremental concentrations of Uracil (0.299 mM, 0.423 mM, 0.5 98 mM, 0.846 mM, 1.20 mM, 1.69 mM, 2.39 mM, 3.38 mM, 4.77 mM, 6.75 mM, 9.54 mM) in a 0.342 mM solution of **2**.

2 + Caffeine (D_2O, 298 K, 500 MHz)

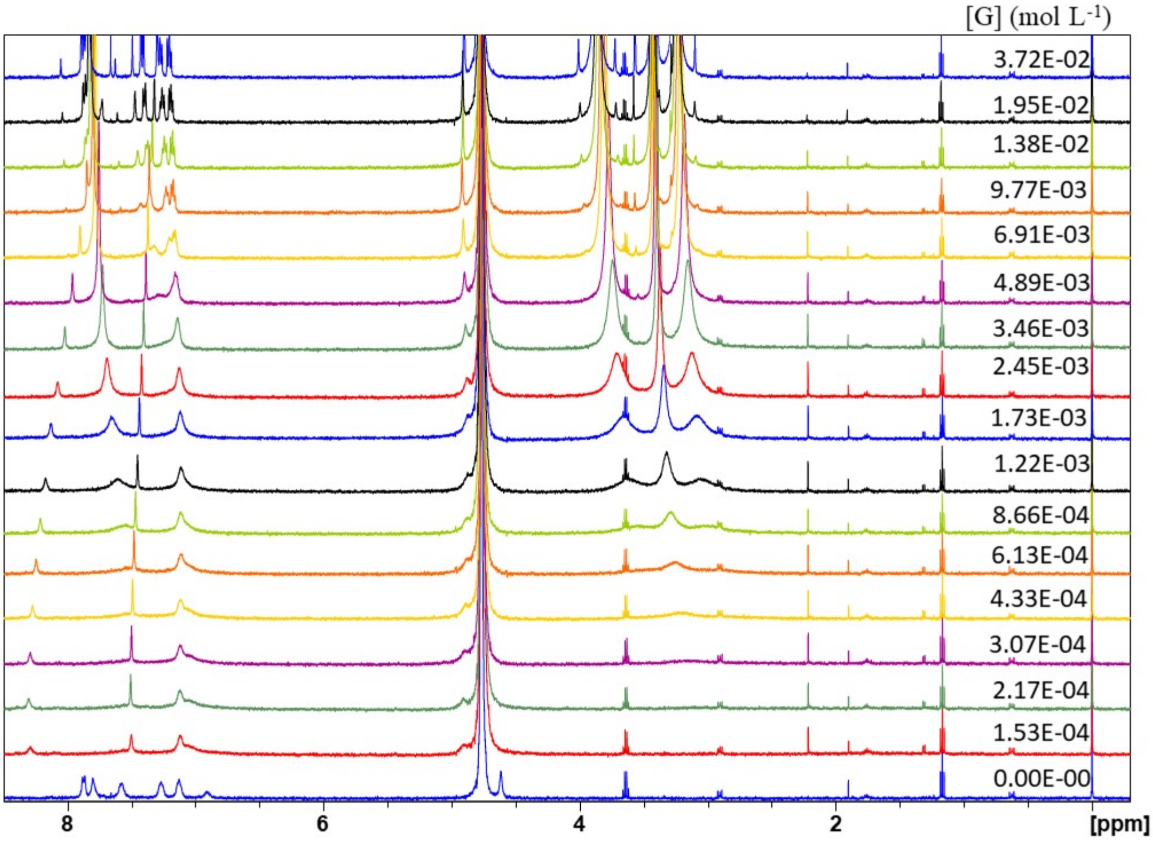

Figure S48. ^{1}H NMR spectroscopic titration (500 MHz, D_2O, 298 K) of receptor **2** (3.25 10^{-4} mol L^{-1}) with incremental concentrations of caffeine (G).

Data Table

R = **2** G = Caffeine

δ (ppm) *vs*. [G] (mol L^{-1})

Titration [R] = 3.25 10^{-4} mol L^{-1}

[G]	CH-8 G	CH3-7 G	CH3-3 G	CH3-1 G	CH-A R	CH-B R
0.00E+00	-	-	-	-	7.8022	6.9160
1.53E-04	-	-	-	-	8.2947	7.5071
2.17E-04	-	-	3.0690	-	8.3073	7.5113
3.07E-04	-	-	3.1530	-	8.2965	7.5058
4.33E-04	-	-	3.2100	2.8670	8.2767	7.4973
6.13E-04	-	3.5340	3.2570	2.8990	8.2504	7.4857
8.66E-04	-	3.5450	3.2913	3.0170	8.2148	7.4720
1.22E-03	7.6130	3.6180	3.3221	3.0540	8.1756	7.4568
1.73E-03	7.6560	3.6590	3.3470	3.0860	8.1335	7.4420
2.45E-03	7.6944	3.7070	3.3732	3.1240	8.0804	7.4254
3.46E-03	7.7304	3.7470	3.3947	3.1540	8.0247	7.4093
4.89E-03	7.7599	3.7803	3.4133	3.1839	7.9656	7.3927
6.91E-03	7.7857	3.8102	3.4270	3.2099	7.9054	7.3759
9.77E-03	7.8035	3.8314	3.4347	3.2267	7.8545	7.3613
1.38E-02	7.8201	3.8490	3.4390	3.2397	-	7.3442
1.95E-02	7.8328	3.8619	3.4382	3.2467	7.7345	7.3266
3.71E-02	7.8445	3.8699	3.4312	3.2474	7.6649	7.3045

δ (ppm) *vs*. [R] (mol L^{-1})

Dilution experiment

[R]	CH-A R	CH-B R
8.46E-06	8.1632	7.2708
1.61E-05	8.0847	7.1734
3.05E-05	-	7.0834
5.80E-05	7.9428	7.0085
1.10E-04	7.8814	6.9544
1.56E-04	7.8475	6.9327
2.20E-04	-	6.9192
3.11E-04	7.7737	6.9208
4.40E-04	7.7216	6.9337
6.22E-04	7.6551	6.9710
8.79E-04	7.5930	6.9815
1.24E-03	7.4853	-
2.36E-03	-	-
4.48E-03	-	-
8.51E-03	-	-

Results page

```
 no. of spectra            32
 no. of resonance values  101
 no. of resonant nuclei     6

Chi-squared =  27.71

   sigma =   0.01097797037          RMS weighted residual =   0.00933303292
       stoich            value   relative    log    standard
       coeff                     std devn    beta   deviation
 Beta  0  2 constant 4.7315E+004            4.6750            (   R2   )
 Beta  0  4 constant 1.1479E+012           12.0599            (   R4   )
 Beta  2  0 constant    5.9938              0.7777            (   G2   )
 Beta  1  1 refined  5.5161E+004  0.2074    4.7416   0.0901  (   GR   )
 Beta  1  2 refined  8.0636E+010  0.4581   10.9065   0.1989  (   GR2  )

 Individual chemical shifts
                   G                        R
 ==========================================================
       +       value    error         value    error
CH-8    +      7.9030   0.0479
CH3-7   +      3.9587   0.0283
CH3-3   +      3.4606   0.0131
CH3-1   +      3.3019   0.0241
CH-A    +                             8.3346   0.0175
CH-B    +                             7.5641   0.0176
                  0,2                        0,4
 ==========================================================
       +       value    error         value    error
CH-8    +
CH3-7   +
CH3-3   +
CH3-1   +
CH-A    +      7.7892   0.0125        6.6051   0.0490
CH-B    +      6.6981   0.0133        7.6695   0.0658
                  2,0                        1,1
 ==========================================================
       +       value    error         value    error
CH-8    +      7.7998   0.1266        2.4667   2.1116
CH3-7   +      3.7660   0.0930       -2.8069   1.0687
CH3-3   +      3.4163   0.0624        1.8761   0.2793
CH3-1   +      3.1975   0.0858       -0.6906   0.7614
CH-A    +                             7.3339   0.0473
CH-B    +                             7.1822   0.0179
                  1,2
 =================================
       +       value    error
CH-8    +      8.1061   0.8300
CH3-7   +      4.3849   0.2569
CH3-3   +      3.0845   0.0353
CH3-1   +      3.0179   0.1534
CH-A    +      8.3885   0.0087
CH-B    +      7.5401   0.0066
 Correlation coefficients*1000
      1    2
   1
   2  936

 Parameters are numbered as follows
    1 beta 1,1
    2 beta 1,2
```

Titration Plots

Chemical shifts (δ, ppm) *vs*. point number

experimental (symbols) and calculated (lines) values

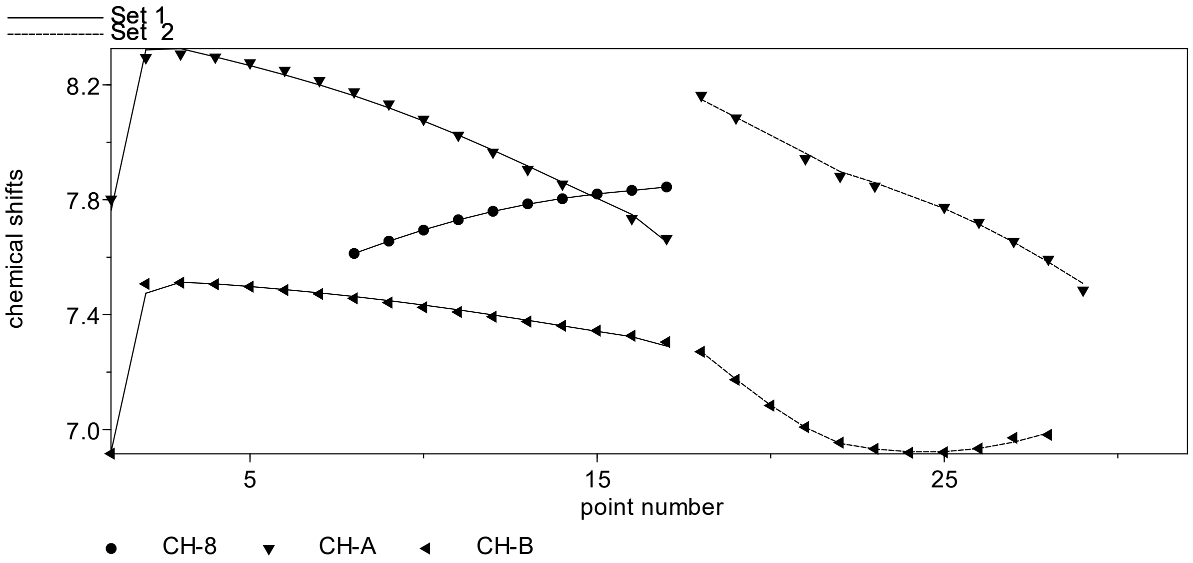

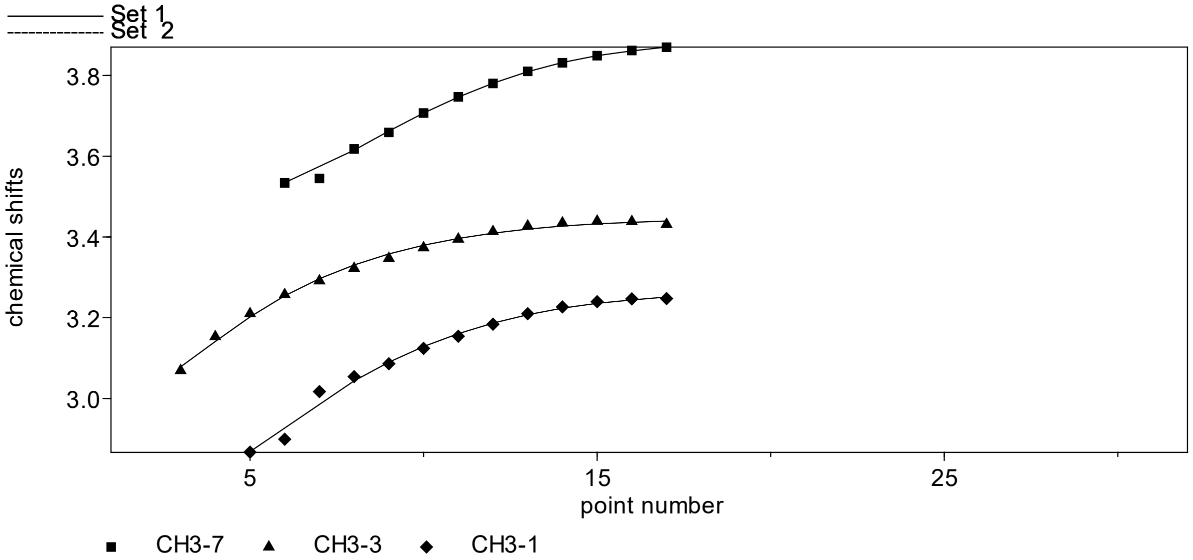

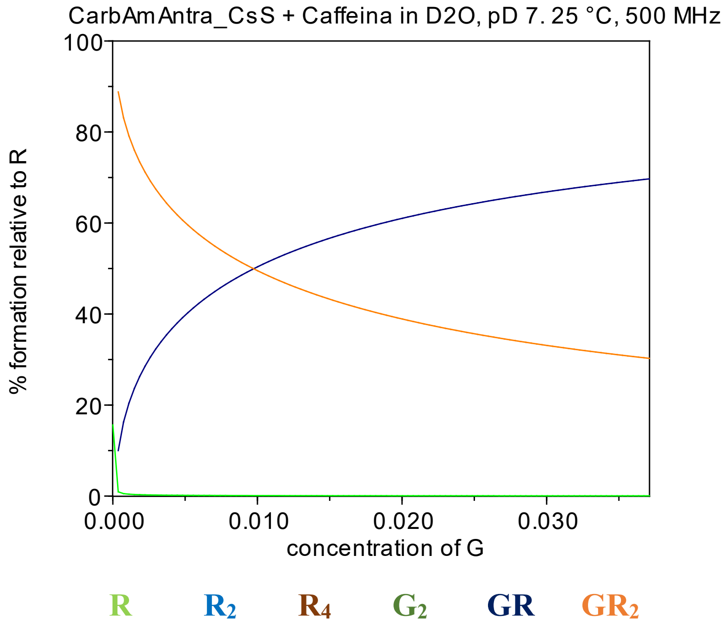

2 + Theophylline (D_2O, 298 K, 500 MHz)

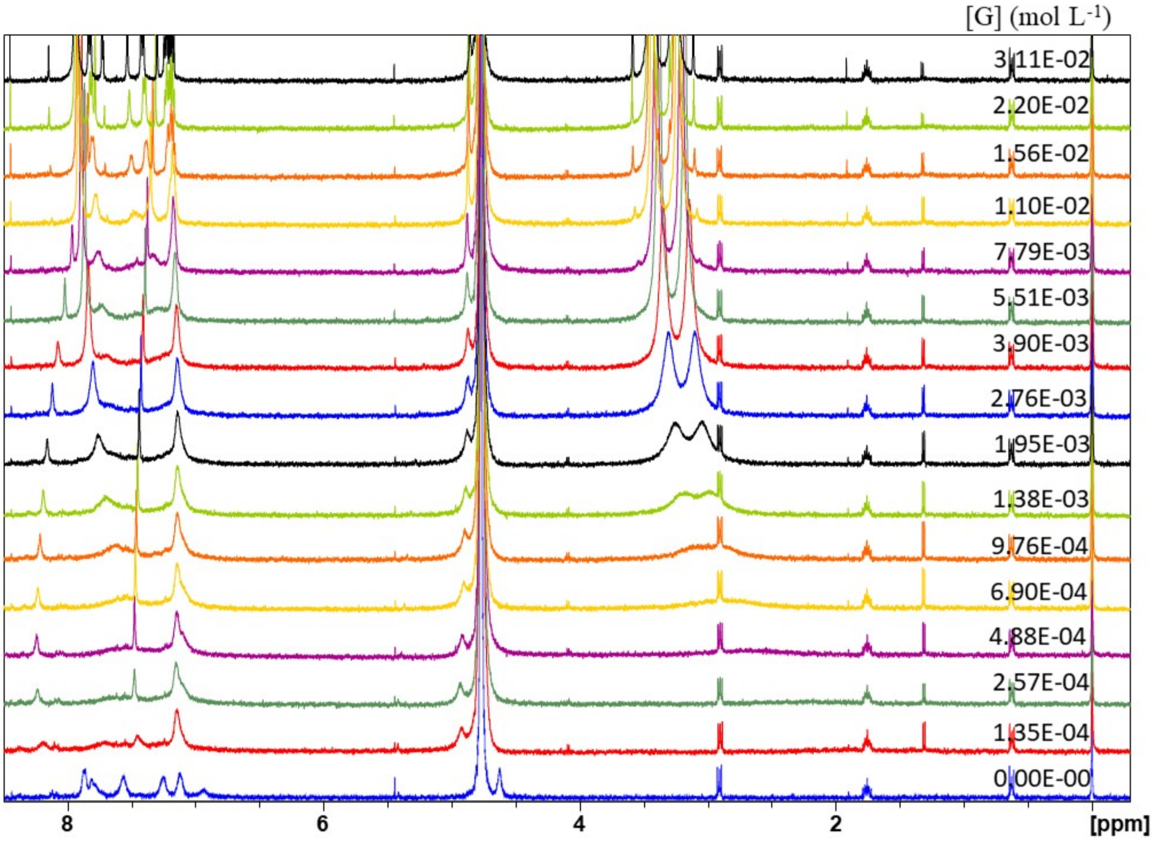

Figure S49. ^{1}H NMR spectroscopic titration (500 MHz, D_2O, 298 K) of receptor **2** (3.66 10^{-4} mol L^{-1}) with incremental concentrations of theophylline (G).

Data Table

R = **2** G = Theophylline

δ (ppm) *vs*. [G] (mol L^{-1})

Titration [R] = 3.66 10^{-4} mol L^{-1}

[G]	CH-8<br>G	CH3-1<br>G	CH3-3<br>G	CH-A<br>R	CH-B<br>R	CH2<br>R
0.00E+00	-	-	-	7.7680	6.9420	4.6239
1.35E-04	-	-	-	8.1917	7.4571	4.9239
2.57E-04	-	-	-	8.2378	7.4806	4.9333
4.88E-04	-	-	-	8.2454	7.4809	4.9222
6.90E-04	-	-	-	8.2364	7.4756	4.9116
9.76E-04	7.6339	3.0810	2.8890	8.2185	7.4676	4.9014
1.38E-03	7.7015	3.1743	2.9852	8.1931	7.4572	4.8937
1.95E-03	7.7600	3.2498	3.0461	8.1625	7.4453	4.8858
2.76E-03	7.8057	3.3022	3.0983	8.1234	7.4308	4.8811
3.90E-03	7.8415	3.3475	3.1424	8.0782	7.4152	4.8794
5.51E-03	7.8707	3.3824	3.1793	8.0238	7.3968	4.8794
7.79E-03	7.8945	3.4102	3.2093	7.9683	7.3785	4.8804
1.10E-02	7.9129	3.4307	3.2321	-	7.3584	4.8793
1.56E-02	7.9269	3.4442	3.2483	7.8476	7.3386	4.8759
2.20E-02	7.9347	3.4497	3.2570	7.7918	7.3194	4.8692
3.11E-02	7.9387	3.4489	3.2603	7.7316	7.2988	4.8600

Theophylline dilution experiment

[G]	CH-8	CH3-1	CH3-3
1.77E-04	8.0127	3.5724	3.3685
3.37E-04	8.0128	3.5716	3.3678
6.40E-04	8.0123	3.5702	3.3668
1.22E-03	8.0115	3.5677	3.3649
2.31E-03	8.0098	3.5633	3.3615
4.39E-03	8.0063	3.5556	3.3557
6.20E-03	8.0035	5.5497	3.3513
8.76E-03	7.9996	3.5420	3.3455
1.24E-02	7.9950	3.5330	3.3387
1.75E-02	7.9889	3.5216	3.3300
2.48E-02	7.9815	3.5076	3.3193
3.50E-02	7.9726	3.4911	3.3066

δ (ppm) *vs*. [R] (mol L^{-1})

Receptor 2 dilution experiment

[R]	CH-A	CH-B	CH2
8.46E-06	8.1632	7.2708	5.0310
1.61E-05	8.0847	7.1734	-
3.05E-05	-	7.0834	-
5.80E-05	7.9428	7.0085	-
1.10E-04	7.8814	6.9544	-
1.56E-04	7.8475	6.9327	4.6622
2.20E-04	-	6.9192	4.6304
3.11E-04	7.7737	6.9208	4.6022
4.40E-04	7.7216	6.9337	4.5776
6.22E-04	7.6551	6.9710	4.5575
8.79E-04	7.5930	6.9815	4.5415
1.24E-03	7.4853	-	4.5284
2.36E-03	-	-	4.5103
4.48E-03	-	-	4.4940
8.51E-03	-	-	4.4777

Results page

```
 no. of spectra            43
 no. of resonance values  145
 no. of resonant nuclei     6

Chi-squared =  88.88

   sigma =   0.00448922973          RMS weighted residual =   0.00389225315

       stoich            value   relative    log    standard
       coeff                     std devn    beta   deviation

 Beta  2  0 constant    6.4003             0.8062           (   G2    )
 Beta  0  2 constant 4.7315E+004           4.6750           (   R2    )
 Beta  0  4 constant 1.1479E+012          12.0599           (   R4    )
 Beta  1  1 refined  1.0880E+004  0.1647    4.0366   0.0715  (   GR    )
 Beta  1  2 refined  3.9140E+009  0.2866    9.5926   0.1245  (   GR2   )
 Beta  1  4 refined  1.3861E+019  0.5012   19.1418   0.2176  (   GR4   )

 Individual chemical shifts

                  G                       R
 =========================================================
       +       value    error         value    error
CH-8   +      8.0139   0.0018
CH3-1  +      3.5723   0.0018
CH3-3  +      3.3683   0.0018
CH-A   +                              8.3485   0.0073
CH-B   +                              7.5615   0.0073
CH2    +                              5.3155   0.0077
       +
                 2,0                     0,2
 =========================================================
       +       value    error         value    error
CH-8   +      7.8548   0.0130
CH3-1  +      3.2534   0.0130
CH3-3  +      3.1292   0.0130
CH-A   +                              7.7735   0.0054
CH-B   +                              6.6999   0.0056
CH2    +                              4.4689   0.0037
       +
                 0,4                     1,1
 =========================================================
       +       value    error         value    error
CH-8   +                              2.7445   0.4397
CH3-1  +                             -3.3926   0.5159
CH3-3  +                             -4.1158   0.5498
CH-A   +      6.6351   0.0204         7.2821   0.0580
CH-B   +      7.6704   0.0270         7.1574   0.0205
CH2    +      4.4573   0.0066         4.8533   0.0084
       +
                 1,2                     1,4
 =========================================================
       +       value    error         value    error
CH-8   +      7.6176   0.3241         5.3316   1.4494
CH3-1  +      3.3644   0.3362        -1.5201   1.4888
CH3-3  +      3.5735   0.3422        -2.2457   1.5300
CH-A   +      8.4443   0.0182         8.1772   0.0172
CH-B   +      7.5276   0.0078         7.5394   0.0252
CH2    +      4.8856   0.0068         4.9875   0.0153

 Correlation coefficients*1000


      1    2    3
   1
   2  796
   3  917  849

 Parameters are numbered as follows
    1 beta 1,1
    2 beta 1,2
    3 beta 1,4
```

Titration Plots

Chemical shifts (δ, ppm) *vs*. point number

experimental (symbols) and calculated (lines) values

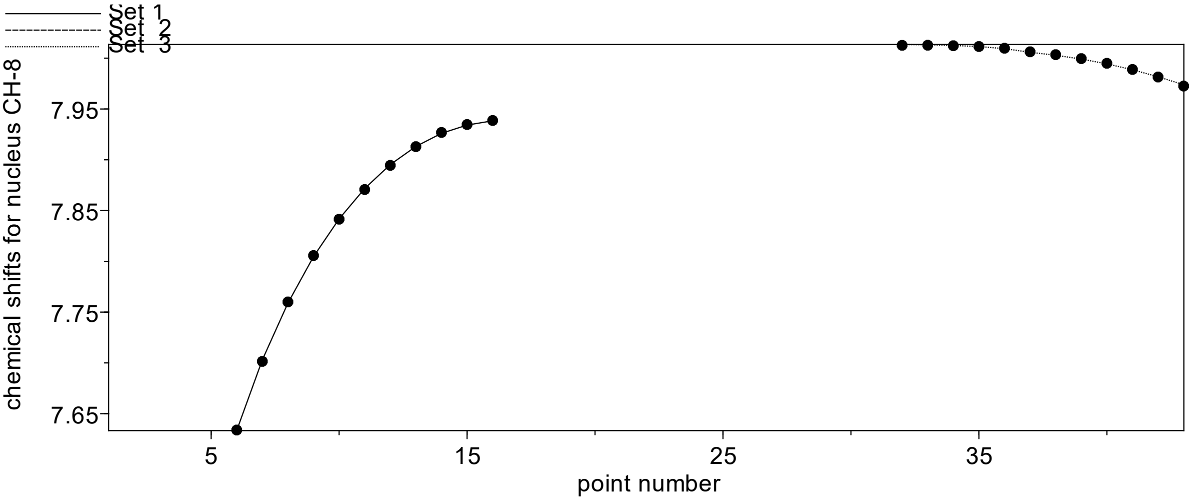

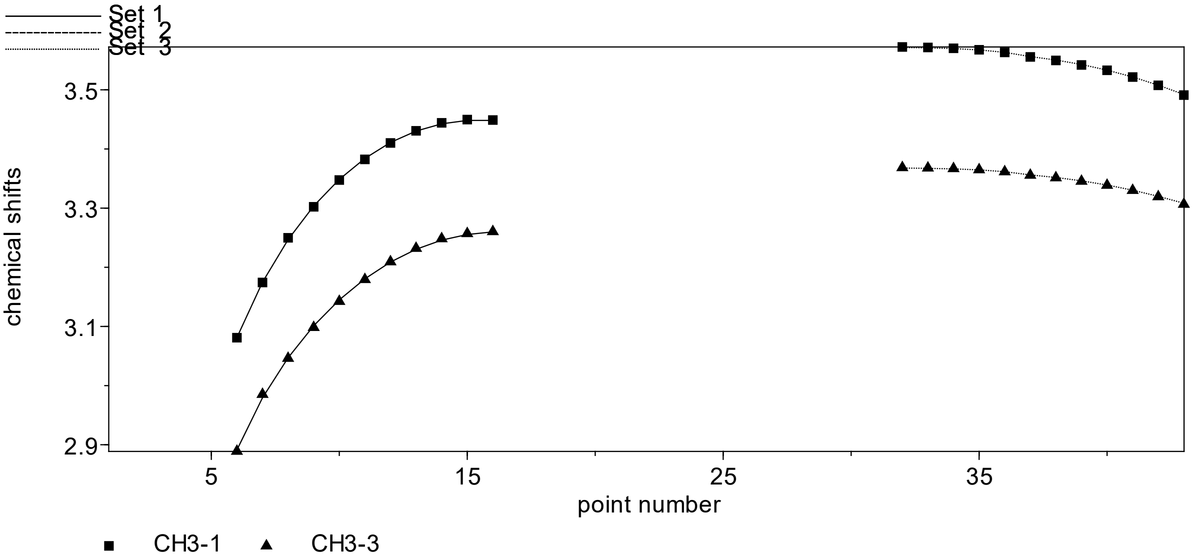

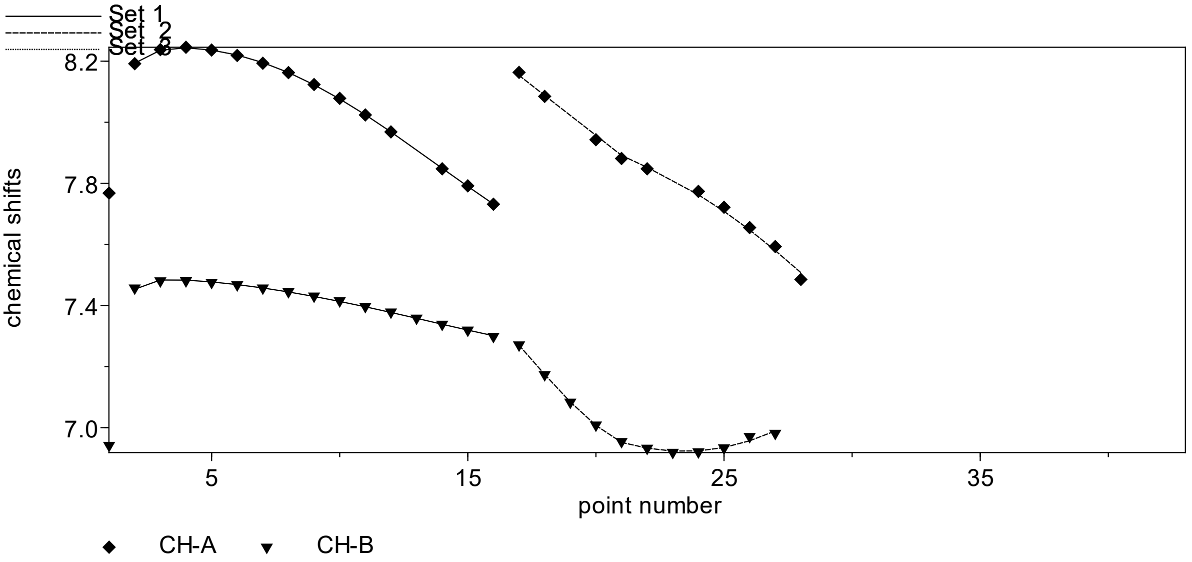

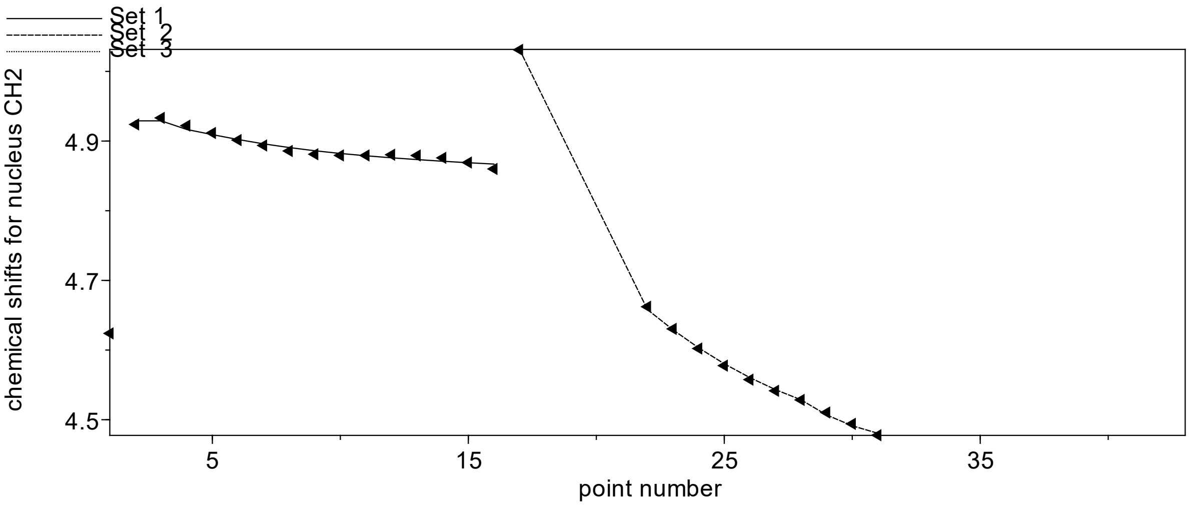
Set 1
Set 2
Set 3
chemical shifts for nucleus CH2
4.9
4.7
4.5
5
15
25
35
point number

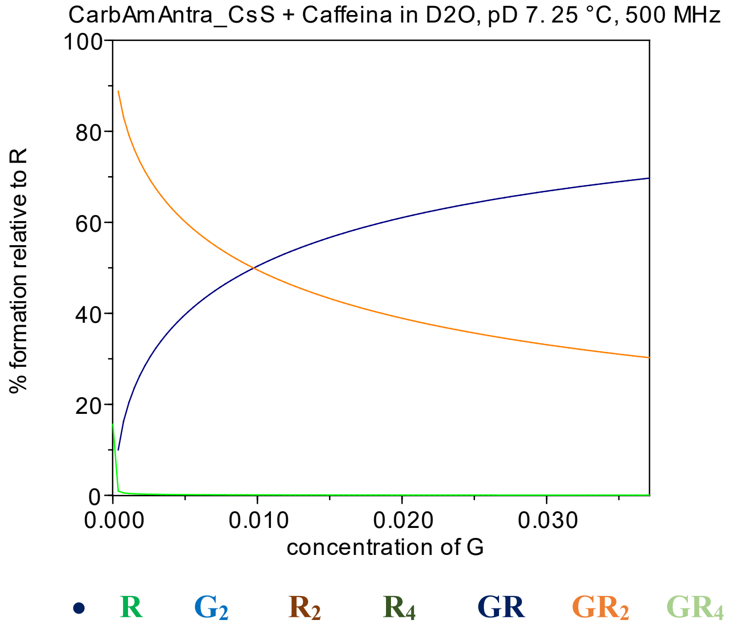
CarbAmAntra_CsS + Caffeina in D2O, pD 7. 25 °C, 500 MHz
% formation relative to R
100
80
60
40
20
0
0.000
0.010
0.020
0.030
concentration of G
R
G2
R2
R4
GR
GR2
GR4

2 + Theobromine (D_2O, 298 K, 500 MHz)

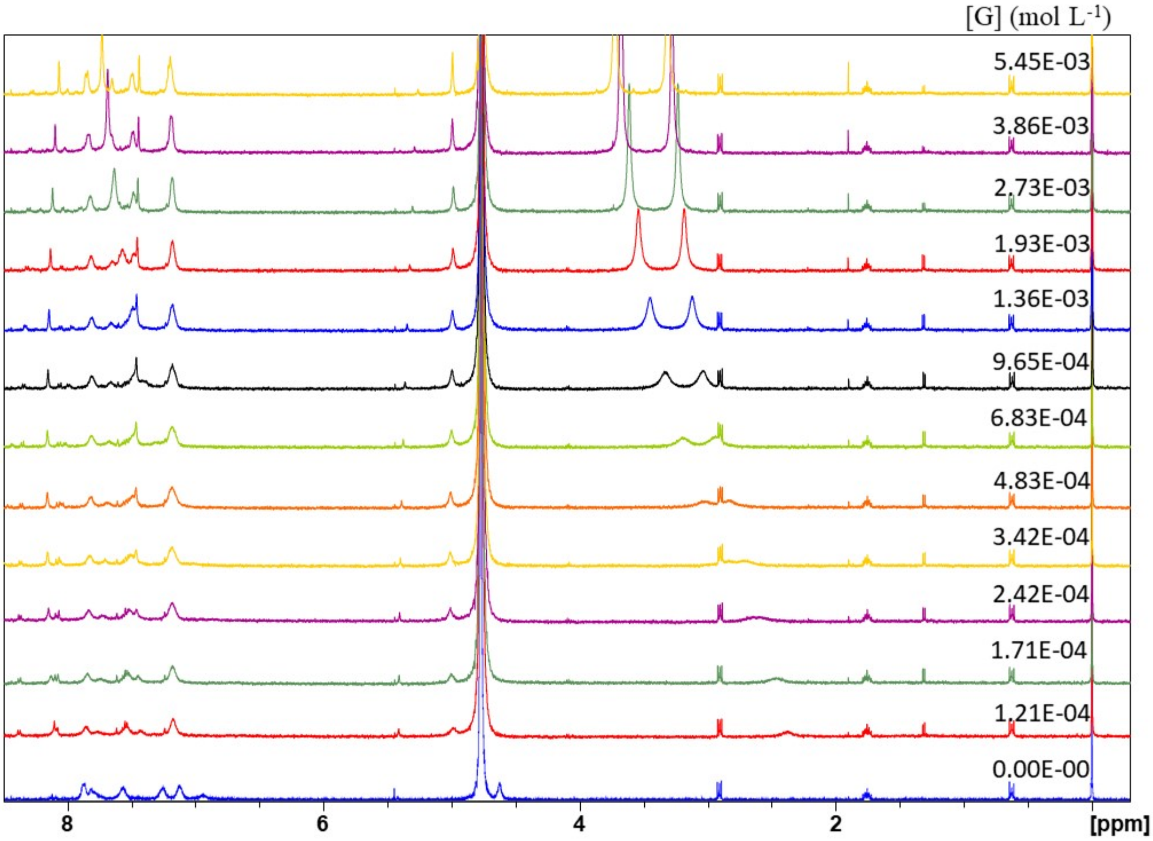

Figure S50. ^{1}H NMR spectroscopic titration (500 MHz, D_2O, 298 K) of receptor **2** (3.66 10^{-4} mol L^{-1}) with incremental concentrations of theobromine (G).

Data Table

R = **2**   G = Theobromine

δ (ppm) *vs*. [G] (mol L^{-1})

Titration [R] = 3.66 10^{-3} mol L^{-1}

[G]	CH-8 G	CH3-7 G	CH3-3 G	CH-B R	CH2 R
0.00E+00	-	-	-	6.9410	4.6254
1.21E-04	-	2.3789	2.3789	7.4330	4.9877
1.71E-04	-	2.4683	2.4683	7.4523	5.0025
2.42E-04	-	2.6544	2.5980	7.4650	5.0123
3.42E-04	-	2.8368	2.7160	7.4695	5.0129
4.83E-04	-	3.0310	2.8390	7.4711	5.0117
6.83E-04	7.3131	3.1963	2.9429	7.4689	5.0055
9.65E-04	7.4075	3.3348	3.0401	7.4669	5.0023
1.36E-03	-	3.4490	3.1185	7.4643	4.9983
1.93E-03	7.5757	3.5395	3.1820	7.4610	4.9967
2.73E-03	7.6407	3.6156	3.2361	7.4555	4.9937
3.86E-03	7.6898	3.6788	3.2797	7.4492	4.9954
5.45E-03	7.7342	3.7316	3.3184	7.4408	5.0000

Dilution experiment 1

[G]	CH3-7	CH3-3
1.28E-04	3.9295	3.4791
2.44E-04	3.9293	3.4787
3.45E-04	3.9291	3.4784
4.87E-04	3.9290	3.4781
6.89E-04	3.9290	3.4778
9.73E-04	3.9285	3.4770
1.38E-03	3.9280	3.4761
1.95E-03	3.9275	3.4749
2.75E-03	3.9265	3.4731

δ (ppm) *vs*. [R] (mol L^{-1})

Dilution experiment 2

[R]	CH-B	CH2
8.46E-06	7.2708	5.0310
1.61E-05	7.1734	-
3.05E-05	7.0834	-
5.80E-05	7.0085	-
1.10E-04	6.9544	-
1.56E-04	6.9327	4.6622
2.20E-04	6.9192	4.6304
3.11E-04	6.9208	4.6022
4.40E-04	6.9337	4.5776
6.22E-04	6.9710	4.5575
8.79E-04	6.9815	4.5415
1.24E-03	-	4.5284
2.36E-03	-	4.5103
4.48E-03	-	4.4940
8.51E-03	-	4.4777

Results page

```
 no. of spectra            35
 no. of resonance values   90
 no. of resonant nuclei     5

Chi-squared =  19.51

   sigma =   0.00566173924          RMS weighted residual =   0.00484842382

      stoich            value   relative    log    standard
      coeff                     std devn    beta   deviation

 Beta  2  0 constant    5.5976             0.7480           (   G2   )
 Beta  0  2 constant 4.7315E+004           4.6750           (   R2   )
 Beta  0  4 constant 1.1479E+012          12.0599           (   R4   )
 Beta  1  2 refined  1.0771E+009  0.1302    9.0323   0.0565  (   GR2  )
 Beta  1  4 refined  1.6121E+019  0.2998   19.2074   0.1302  (   GR4  )

 Individual chemical shifts

                  G                       R
 ==========================================================
       +        value    error         value    error
CH-8    +       7.9519   0.0927
CH3-7   +       3.9317   0.0025
CH3-3   +       3.4805   0.0025
CH-B    +                              7.5618   0.0092
CH2     +                              5.3156   0.0097
       +
                2,0                       0,2
 ==========================================================
       +        value    error         value    error
CH-8    +       7.3002   0.8924
CH3-7   +       3.7068   0.1545
CH3-3   +       2.7919   0.1330
CH-B    +                              6.6995   0.0070
CH2     +                              4.4690   0.0047
       +
                0,4                       1,2
 ==========================================================
       +        value    error         value    error
CH-8    +                             -1.2561   2.5706
CH3-7   +                             -4.9684   0.6483
CH3-3   +                             -2.8917   0.4666
CH-B    +       7.6719   0.0340        7.4041   0.0093
CH2     +       4.4572   0.0083        4.9630   0.0081
       +
                1,4
 ==================================
       +        value    error
CH-8    +       8.4219   1.2174
CH3-7   +       2.9777   0.0202
CH3-3   +       2.8462   0.0186
CH-B    +       7.5155   0.0093
CH2     +       5.0323   0.0065

 Correlation coefficients*1000

      1    2
   1
   2  768

 Parameters are numbered as follows
    1 beta 1,2
    2 beta 1,4
```

Titration Plots

Chemical shifts (δ, ppm) *vs*. point number

experimental (symbols) and calculated (lines) values

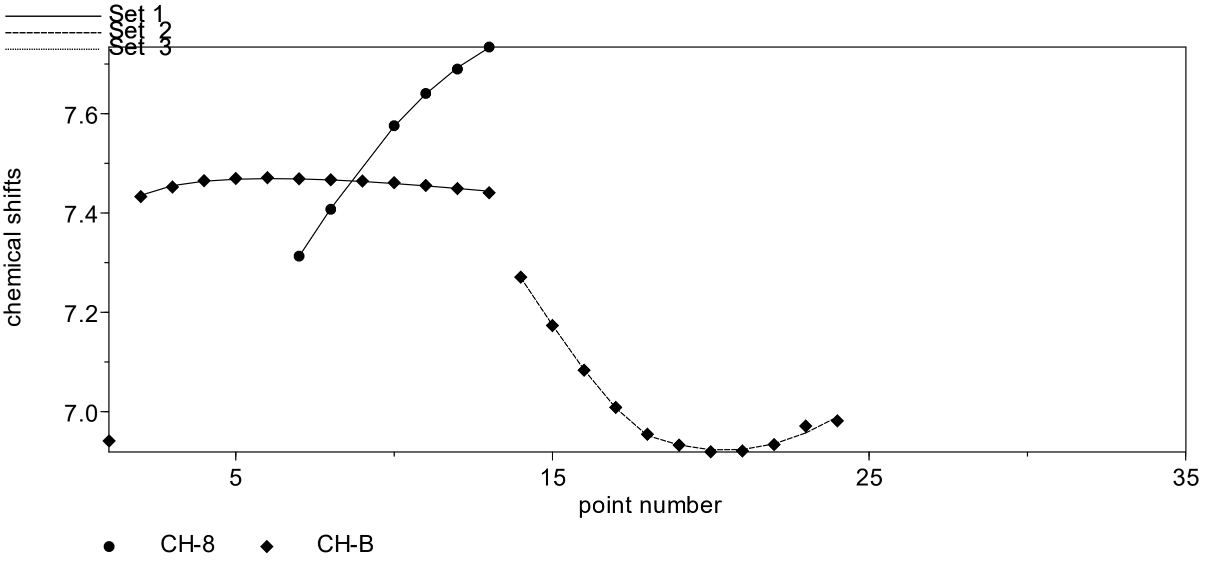

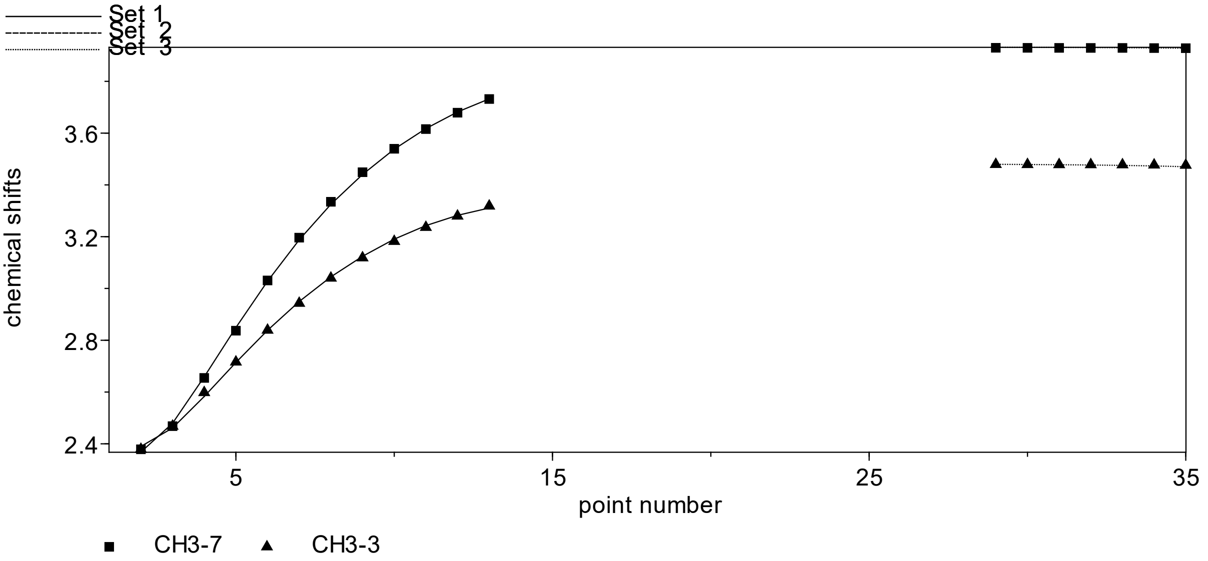

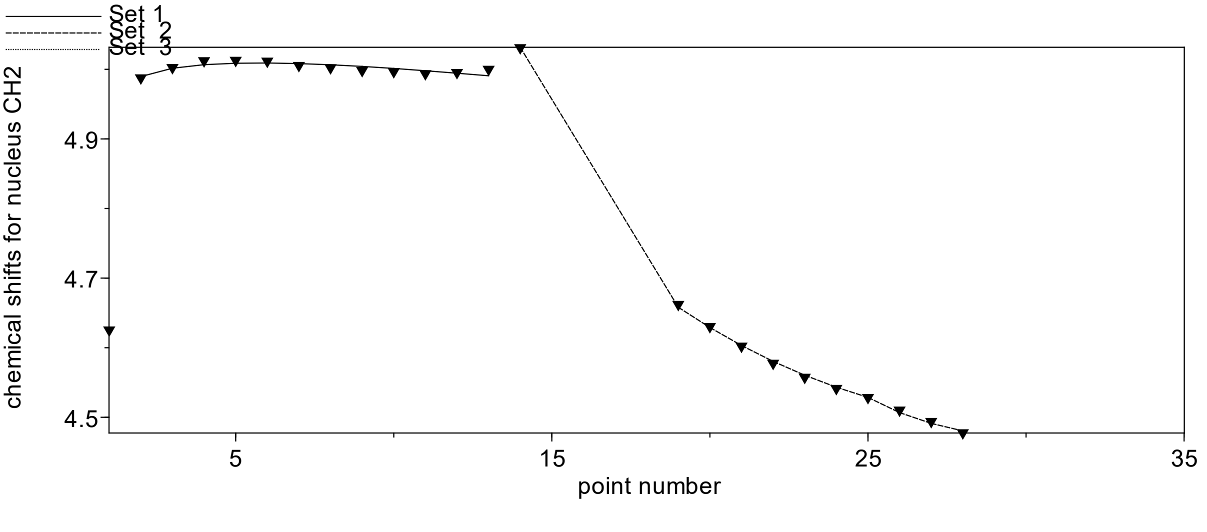

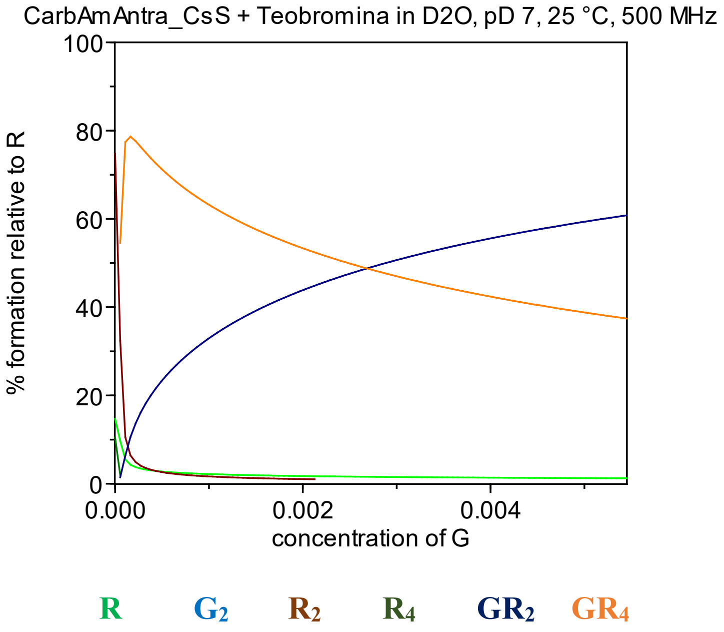
CarbAmAntra_CsS + Teobromina in D2O, pD 7, 25 °C, 500 MHz
% formation relative to R
concentration of G
100
80
60
40
20
0
0.000
0.002
0.004
R
G2
R2
R4
GR2
GR4

Dilution of receptor 4 (D_2O, 298 K, 500 MHz).

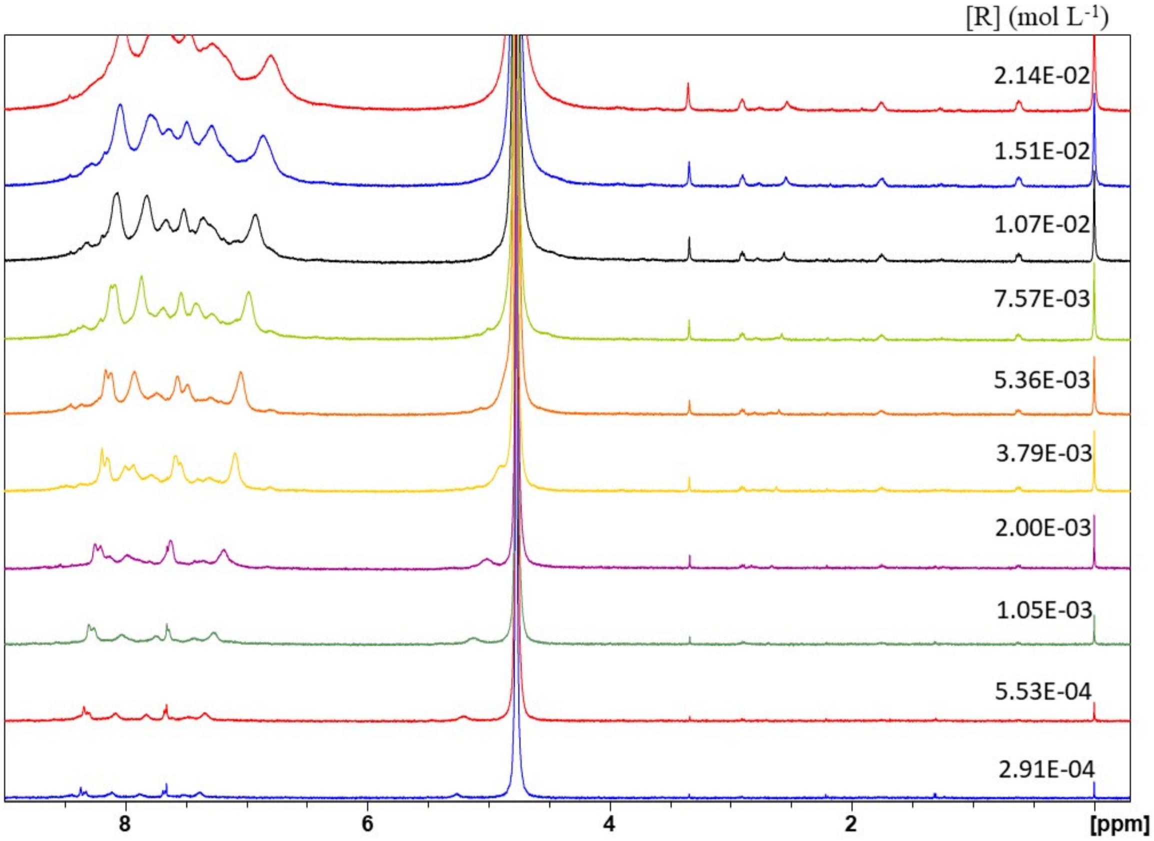

Figure S51. ^{1}H NMR spectroscopic spectra (500 MHz, D_2O, 298 K) of receptor 4 (R) at different concentrations in dilution experiment.

Data Table

R = **4**

δ (ppm) *vs*. [R] (mol L^{-1})

[R]	CH-4c R	CH-1a R	CH-10a R	CH2 R
2.91E-04	8.3712	7.6818	7.3892	5.2667
5.53E-04	8.3447	7.6704	7.3456	5.2059
1.05E-03	8.3041	7.6507	7.2738	5.1308
2.00E-03	8.2535	7.6273	7.1919	5.0210
3.79E-03	8.1954	7.5886	7.0966	4.9080
5.36E-03	8.1647	7.5703	7.0463	4.8780
7.57E-03	8.1220	7.5437	6.9852	-
1.07E-02	8.0867	7.5198	6.9274	-
1.51E-02	8.0474	7.4964	6.8646	-
2.14E-02	8.0211	7.4802	6.8057	-

Results page

```
 no. of spectra            10
 no. of resonance values   36
 no. of resonant nuclei     4

Chi-squared =   6.67

   sigma =   0.00841008388          RMS weighted residual =   0.00728334629

      stoich          value   relative    log    standard
      coeff                   std devn    beta   deviation

 Beta  2 refined  1.0959E+002  0.0807    2.0398   0.0350  (   R2   )

 Individual chemical shifts

                   R                       2
 =========================================================
       +        value    error         value    error
CH-4c   +       8.4053   0.0068         7.7991   0.0148
CH-1a   +       7.7084   0.0060         7.3533   0.0115
CH-10a  +       7.4425   0.0086         6.4524   0.0210
CH2     +       5.3173   0.0081         4.1902   0.0409
```

Titration Plots

Chemical shifts (δ, ppm) *vs*. concentration of R (mol L[-1])
experimental (symbols) and calculated (lines) values

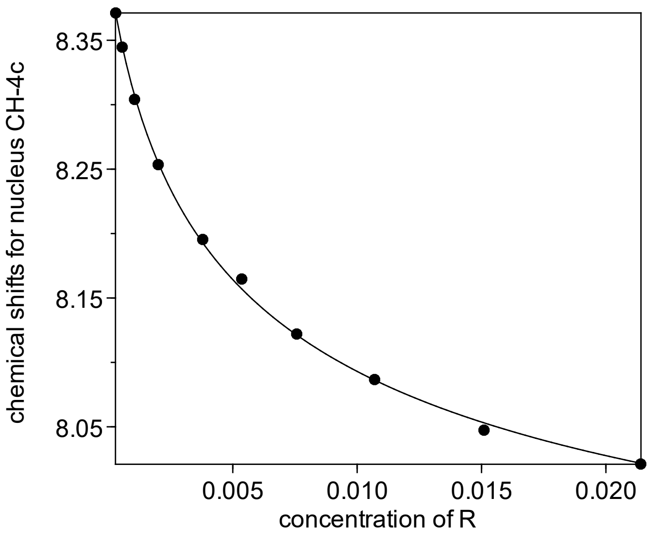

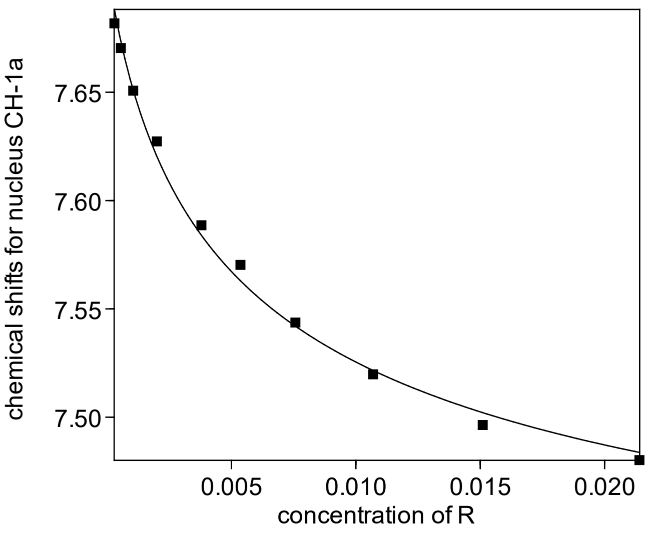

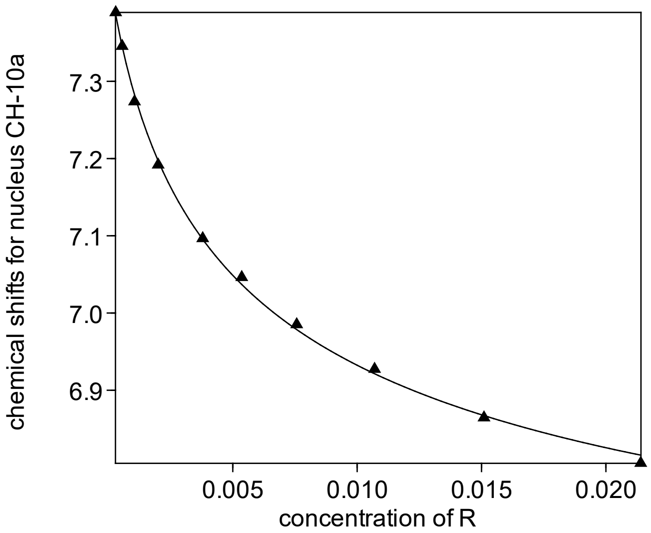

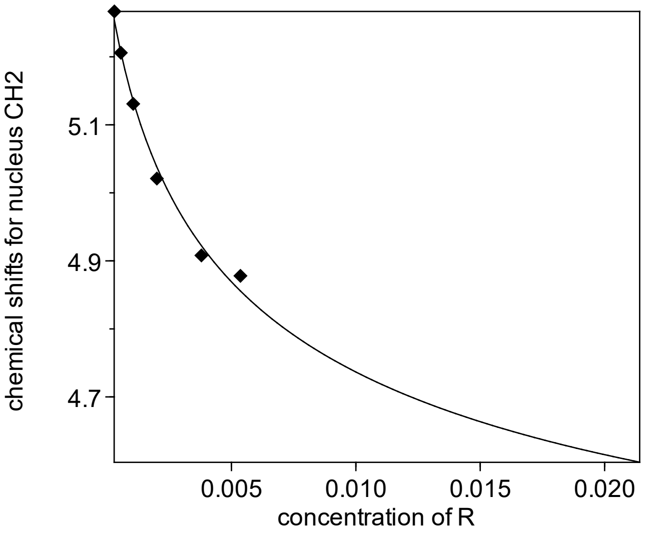

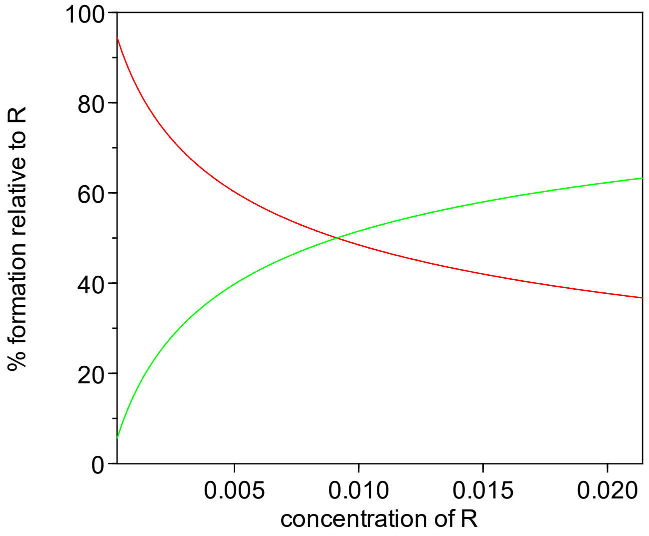

R **R_2**

5 + Caffeine (D_2O, 298 K, 500 MHz)

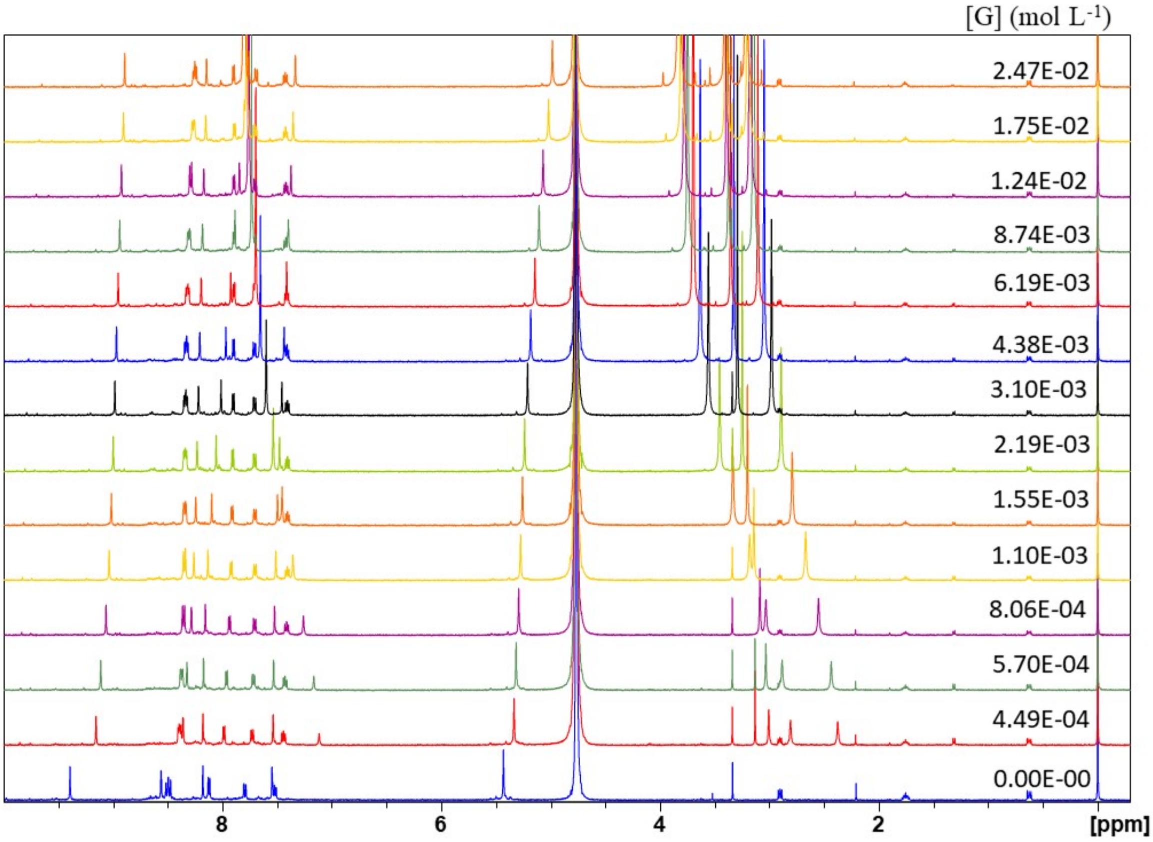

Figure S52. ^{1}H NMR spectroscopic titration (500 MHz, D_2O, 298 K) of receptor **5** (6.03 10^{-4} mol L^{-1}) with incremental concentrations of caffeine (G).

Data Table

R = **5** G = Caffeine

δ (ppm) *vs*. [G] (mol L^{-1})

Titration [R] = 6.03 10^{-4} mol L^{-1}

[G]	CH-8 G	CH3-7 G	CH3-3 G	CH3-1 G	CH-10A R	CH-4A R
0.00E+00	-	-	-	-	9.3971	8.5647
4.49E-04	7.1167	2.8094	3.0083	2.3770	9.1580	8.3623
5.70E-04	7.1652	2.8873	3.0357	2.4363	9.1154	8.3259
8.06E-04	7.2605	3.0346	3.0895	2.5527	9.0668	8.2856
1.10E-03	7.3568	3.1836	3.1450	2.6704	9.0390	8.2626
1.55E-03	7.4552	3.3366	3.2038	2.7942	9.0179	8.2458
2.19E-03	7.5370	3.4604	3.2531	2.8976	9.0016	8.2327
3.10E-03	7.6027	3.5595	3.2941	2.9820	8.9865	8.2216
4.38E-03	7.6558	3.6378	3.3281	3.0508	8.9722	8.2103
6.19E-03	7.6989	3.6991	3.3556	3.1054	8.9568	8.1979
8.74E-03	7.7338	3.7467	3.3771	3.1478	8.9415	8.1853
1.24E-02	7.7620	3.7824	3.3926	3.1791	8.9255	8.1718
1.75E-02	7.7851	3.8100	3.4024	3.2009	8.9098	8.1577
2.47E-02	7.8041	3.8294	3.4071	3.2146	8.8949	8.1445

[G]	CH-4C	CH-8A	CH-2A	CH-2C	CH-7A
	R	R	R	R	R
0.00E+00	8.1835	8.1337	7.8091	7.5514	7.5283
4.49E-04	8.1797	7.9946	7.7387	7.5381	7.4430
5.70E-04	8.1744	7.9707	7.7274	7.5340	7.4289
8.06E-04	8.1578	7.9432	7.7164	7.5243	7.4137
1.10E-03	8.1344	7.9289	7.7124	7.5131	7.4072
1.55E-03	8.0997	7.9198	7.7128	7.4975	7.4063
2.19E-03	8.0590	7.9142	7.7153	7.4794	7.4072
3.10E-03	8.0151	7.9101	7.7179	7.4592	7.4098
4.38E-03	7.9713	7.9073	7.7196	7.4387	7.4134
6.19E-03	7.9278	7.9055	7.7195	7.4173	7.4151
8.74E-03	7.8864	7.9042	7.7191	7.3975	7.4203
1.24E-02	7.8459	7.9032	7.7155	7.3775	7.4237
1.75E-02	7.8050	7.9033	7.7117	7.3579	7.4271
2.47E-02	7.7654	7.9045	7.7078	7.3395	7.4314

Results page

```
 no. of spectra            14
 no. of resonance values  150
 no. of resonant nuclei    11

Chi-squared =  33.36

   sigma =   0.00375954589          RMS weighted residual =   0.00323408366

       stoich            value   relative    log    standard
       coeff                     std devn    beta   deviation

 Beta  2  0 constant    5.9938              0.7777            (   G2   )
 Beta  1  1 refined  2.9910E+004  0.0856    4.4758   0.0372  (   GR   )
 Beta  2  1 refined  5.5249E+006  0.1020    6.7423   0.0443  (   G2R  )

 Individual chemical shifts

                  G                       R
 =========================================================
        +       value    error         value    error
CH-8    +      7.8477   0.0178
CH3-7   +      3.9251   0.0186
CH3-3   +      3.4932   0.0175
CH3-1   +      3.3522   0.0185
CH-10A  +                             9.3959   0.0036
CH-4A   +                             8.5631   0.0036
CH-4C   +                             8.1871   0.0036
CH-8A   +                             8.1346   0.0036
CH-2A   +                             7.8058   0.0036
CH-2C   +                             7.5522   0.0036
CH-7A   +                             7.5282   0.0036
        +
                2,0                      1,1
 =========================================================
        +       value    error         value    error
CH-8    +      7.8767   0.0663        7.0209   0.0081
CH3-7   +      3.7841   0.0680        2.6623   0.0119
CH3-3   +      3.2532   0.0657        2.9500   0.0056
CH3-1   +      3.0138   0.0674        2.2551   0.0100
CH-10A  +                             9.0306   0.0030
CH-4A   +                             8.2556   0.0027
CH-4C   +                             8.1723   0.0026
CH-8A   +                             7.9160   0.0024
CH-2A   +                             7.7085   0.0021
CH-2C   +                             7.5307   0.0022
CH-7A   +                             7.3959   0.0021
        +
                2,1
 =================================
        +       value    error
CH-8    +      6.6764   0.1136
CH3-7   +      2.2454   0.1241
CH3-3   +      2.4327   0.1116
CH3-1   +      1.4629   0.1270
CH-10A  +      8.8652   0.0035
CH-4A   +      8.1230   0.0033
CH-4C   +      7.6595   0.0066
CH-8A   +      7.8965   0.0030
CH-2A   +      7.7187   0.0029
CH-2C   +      7.2910   0.0040
CH-7A   +      7.4390   0.0030

 Correlation coefficients*1000

      1    2
   1
   2  931

 Parameters are numbered as follows
    1 beta 1,1
    2 beta 2,1
```

Titration Plots

Chemical shifts (δ, ppm) *vs*. concentration of G (mol L^{-1})

experimental (symbols) and calculated (lines) values

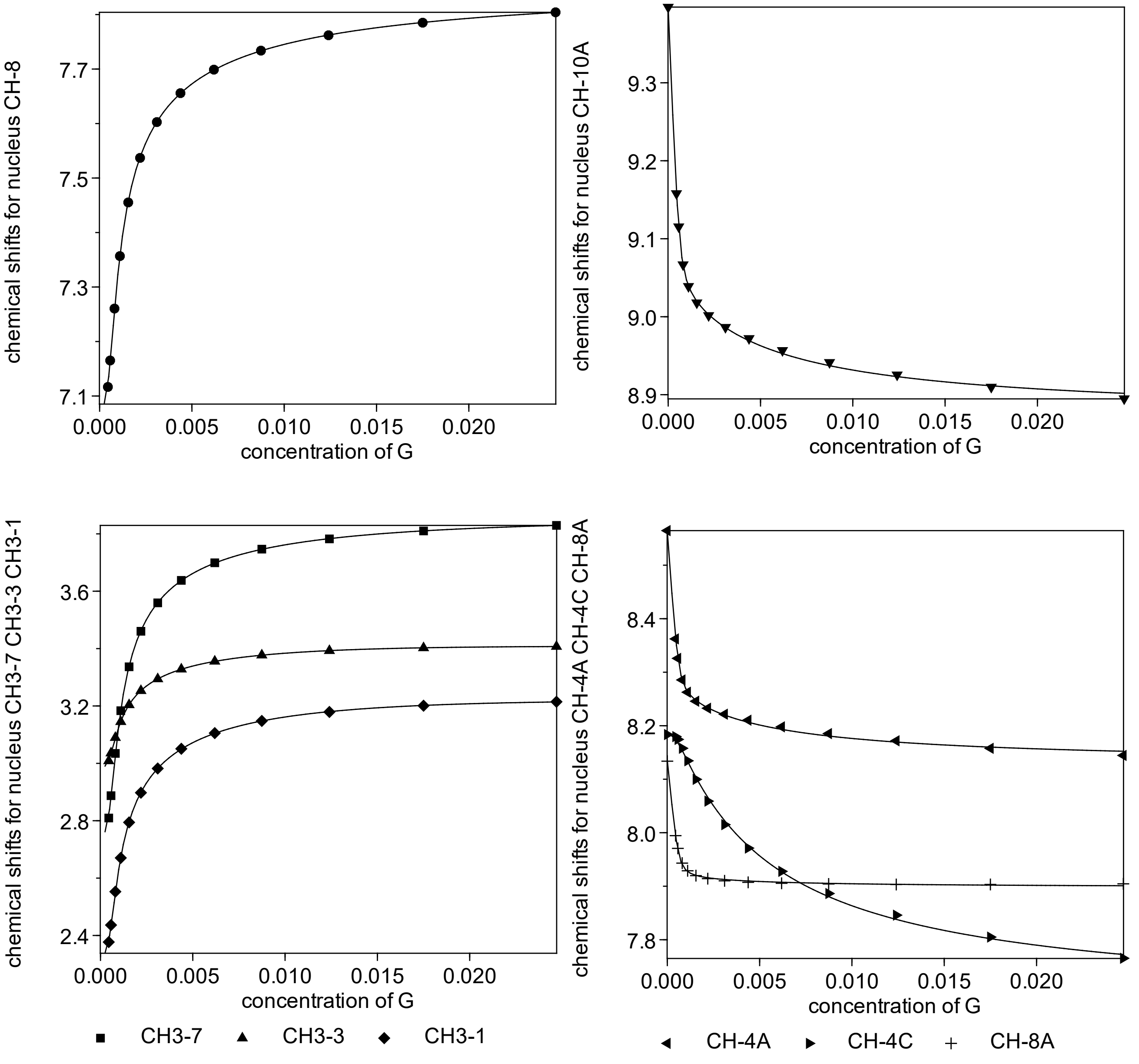

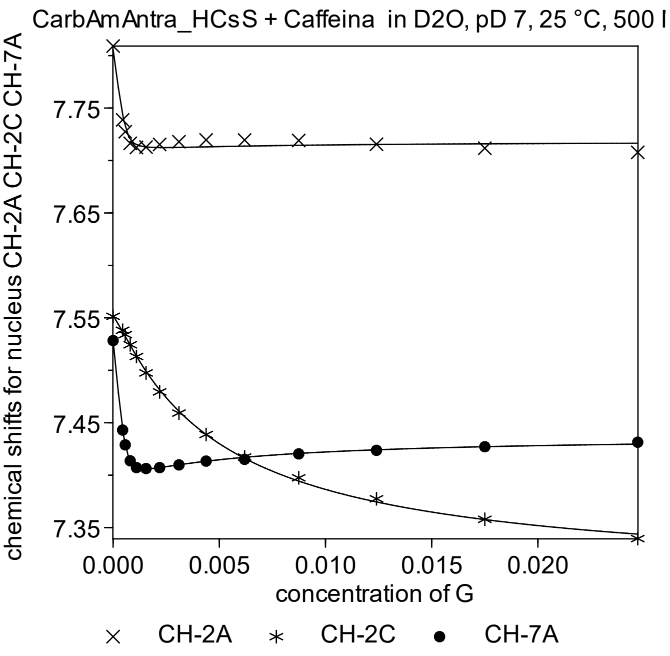

CarbAmAntra_HCsS + Caffeina in D2O, pD 7, 25 °C, 500 I
chemical shifts for nucleus CH-2A CH-2C CH-7A
7.75
7.65
7.55
7.45
7.35
0.000
0.005
0.010
0.015
0.020
concentration of G
CH-2A
CH-2C
CH-7A

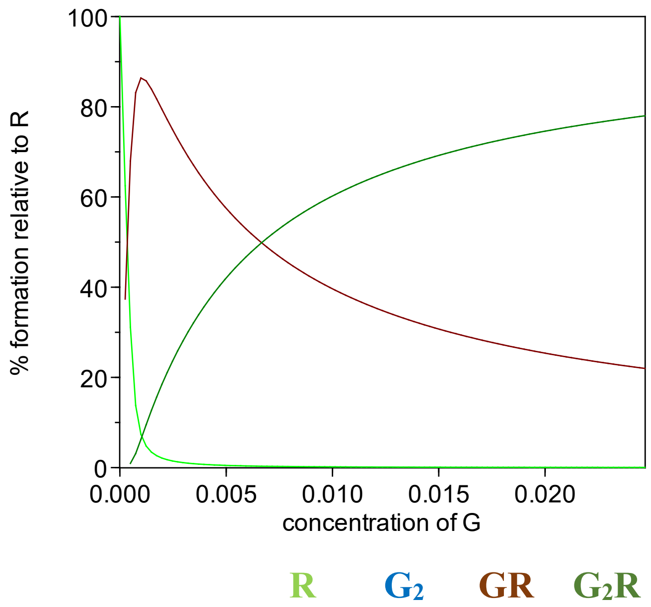

% formation relative to R
100
80
60
40
20
0
0.000
0.005
0.010
0.015
0.020
concentration of G
R
G2
GR
G2R

6 + Caffeine (D_2O, 298 K, 500 MHz)

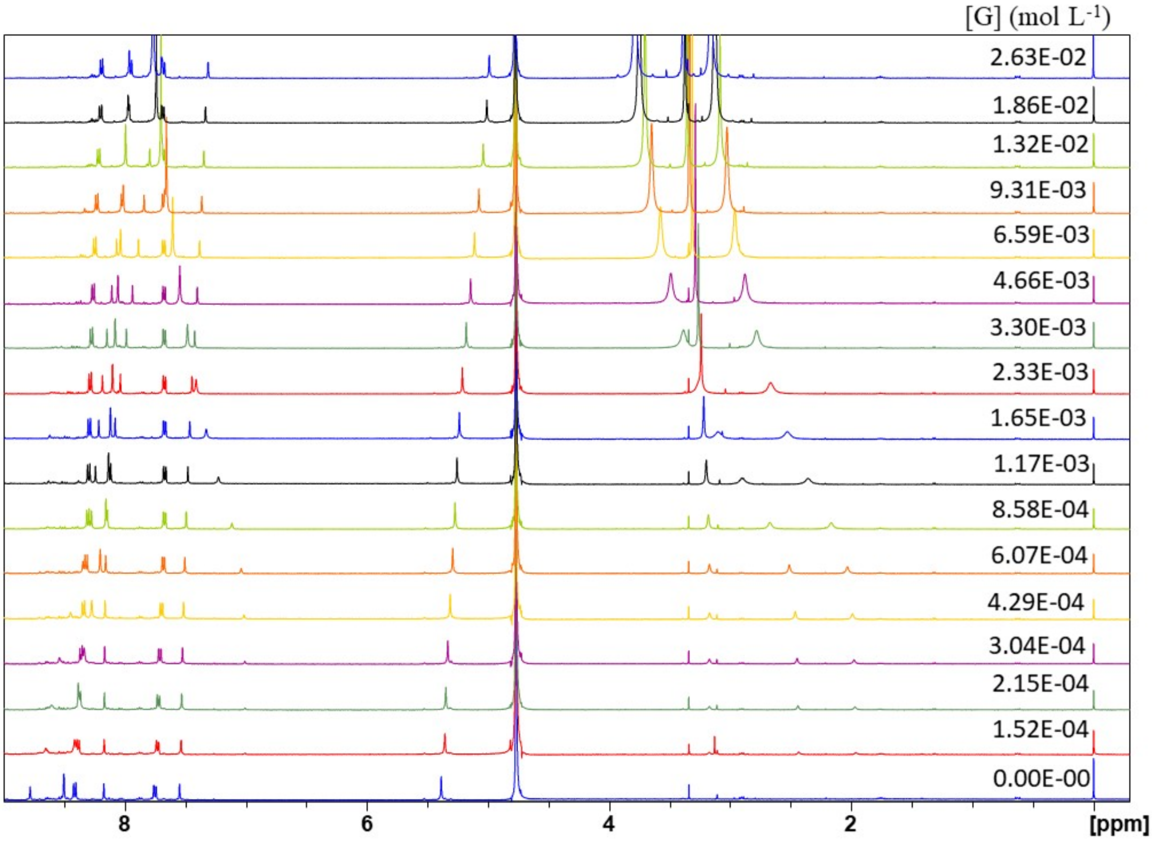

Figure S53. ^{1}H NMR spectroscopic titration (500 MHz, D_2O, 298 K) of receptor **6** (6.99 10^{-4} mol L^{-1}) with incremental concentrations of caffeine (G).

Data Table

R = **6** G = Caffeine

δ (ppm) *vs*. [G] (mol L^{-1})

Titration [R] = 6.99 10^{-4} mol L^{-1}

[G]	CH-8 G	CH3-7 G	CH3-3 G	CH3-1 G	CH-10A R	CH-4A R
0.00E+00	-	-	-	-	8.7848	8.5053
1.52E-04	7.0110	2.4355	3.1735	1.9643	8.6560	8.4176
2.15E-04	7.0110	2.4398	3.1715	1.9687	8.6085	8.3857
3.04E-04	7.0132	3.4471	3.1706	1.9770	8.5435	8.3385
4.29E-04	7.0205	2.4622	3.1697	1.9904	8.4508	8.2764
6.07E-04	7.0431	2.5118	3.1710	2.0318	8.3492	8.2065
8.58E-04	7.1197	2.6720	3.1791	2.1649	8.2782	8.1470
1.17E-03	7.2315	2.9008	3.1969	2.3574	8.2454	8.1196
1.65E-03	7.3319	3.1001	3.2176	2.5269	8.2182	8.0831
2.33E-03	7.4159	3.2523	3.2389	2.6660	8.1878	8.0404
3.30E-03	7.4872	3.3861	3.2616	2.7820	8.1508	7.9909
4.66E-03	7.5504	3.4895	3.2867	2.8791	8.1105	7.9401
6.59E-03	7.6062	3.5764	3.3127	2.9605	8.0690	7.8902
9.31E-03	7.6580	3.6485	3.3384	3.0302	8.0303	7.8428
1.32E-02	7.7024	3.7068	3.3603	3.0868	7.9950	7.7960
1.86E-02	7.7399	3.7519	3.3770	3.1298	7.9683	7.7495
2.63E-02	7.7708	3.7863	3.3873	3.1609	7.9451	7.7029

[G]	CH-1A R	CH-4C R	CH-2A R	CH-2C R	CH2 R
0.00E+00	8.4259	8.1761	7.7636	7.5506	5.3901
1.52E-04	8.3982	8.1733	7.7428	7.5390	5.3628
2.15E-04	8.3860	8.1713	7.7345	7.5335	5.3525
3.04E-04	8.3731	8.1695	7.7244	7.5272	5.3388
4.29E-04	8.3535	8.1668	7.7097	7.5186	5.3197
6.07E-04	8.3319	8.1613	7.6942	7.5081	5.2977
8.58E-04	8.3170	8.1589	7.6851	7.4967	5.2787
1.17E-03	8.3107	8.1381	7.6835	7.4835	5.2629
1.65E-03	8.3057	8.1218	7.6846	7.4676	5.2434
2.33E-03	8.2987	8.1052	7.6863	7.4489	5.2185
3.30E-03	8.2882	8.0835	7.6876	7.4272	5.1865
4.66E-03	8.2747	8.0604	7.6887	7.4057	5.1505
6.59E-03	8.2587	8.0366	7.6895	7.3844	5.1119
9.31E-03	8.2427	8.0144	7.6902	7.3652	5.0755
1.32E-02	8.2275	7.9954	7.6916	7.3480	5.0426
1.86E-02	8.2144	7.9785	7.6931	7.3324	5.0157
2.63E-02	8.2035	7.9652	7.6960	7.3180	4.9928

Results page

```
 no. of spectra            17
 no. of resonance values  183
 no. of resonant nuclei    11

Chi-squared =  17.79

   sigma =   0.00406592360          RMS weighted residual =   0.00360674066

      stoich            value   relative    log    standard
      coeff                     std devn    beta   deviation

 Beta  1  1 refined  8.6351E+004  0.0709    4.9363   0.0308  (   GR   )
 Beta  2  1 refined  1.2534E+007  0.0838    7.0981   0.0364  (   G2R  )
 Beta  2  0 constant    5.9938              0.7777           (   G2   )

 Individual chemical shifts

                 G                       R
 =========================================================
       +       value    error         value    error
CH-8   +      7.8574   0.0215
CH3-7  +      3.9478   0.0219
CH3-3  +      3.4961   0.0213
CH3-1  +      3.3518   0.0223
CH-10A +                             8.7688   0.0026
CH-4A  +                             8.4954   0.0027
CH-1A  +                             8.4188   0.0026
CH-4C  +                             8.1796   0.0026
CH-2A  +                             7.7599   0.0026
CH-2C  +                             7.5516   0.0026
CH2    +                             5.3849   0.0026
       +
                1,1                     2,1
 =========================================================
       +       value    error         value    error
CH-8   +      6.9928   0.0025        5.6781   0.1478
CH3-7  +      2.4075   0.0038        1.0074   0.1575
CH3-3  +      3.1643   0.0019        1.8441   0.1428
CH3-1  +      1.9410   0.0035        0.1771   0.1655
CH-10A +      8.2525   0.0025        7.8347   0.0053
CH-4A  +      8.1386   0.0025        7.5648   0.0066
CH-1A  +      8.3186   0.0020        8.1711   0.0036
CH-4C  +      8.1520   0.0021        7.8943   0.0042
CH-2A  +      7.6800   0.0020        7.7009   0.0034
CH-2C  +      7.4938   0.0021        7.2518   0.0041
CH2    +      5.2826   0.0022        4.8915   0.0051
       +
                2,0
 =================================
       +       value    error
CH-8   +      7.8885   0.0723
CH3-7  +      3.7610   0.0728
CH3-3  +      3.2770   0.0720
CH3-1  +      3.0522   0.0733
CH-10A +
CH-4A  +
CH-1A  +
CH-4C  +
CH-2A  +
CH-2C  +
CH2    +

 Correlation coefficients*1000

      1    2
  1
  2  940

 Parameters are numbered as follows
    1 beta 1,1
    2 beta 2,1
```

Titration Plots

Chemical shifts (δ, ppm) *vs*. concentration of G (mol L^{-1})

experimental (symbols) and calculated (lines) values

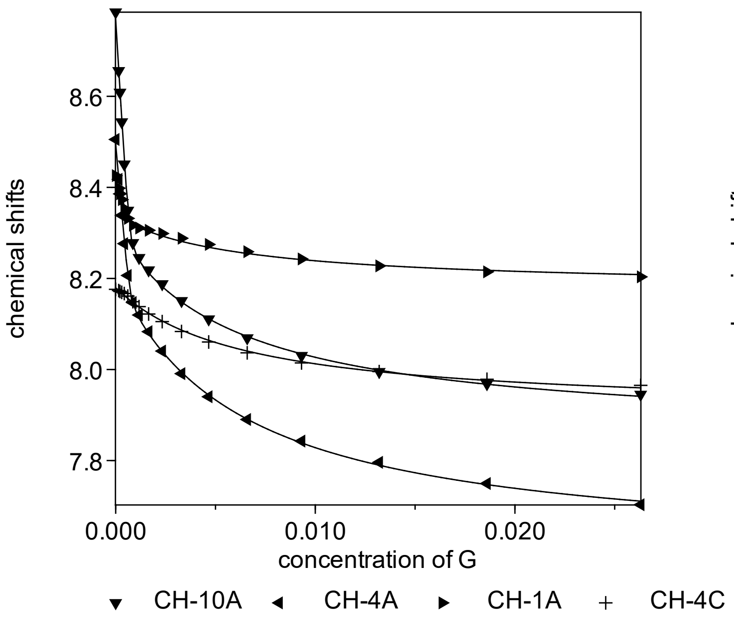

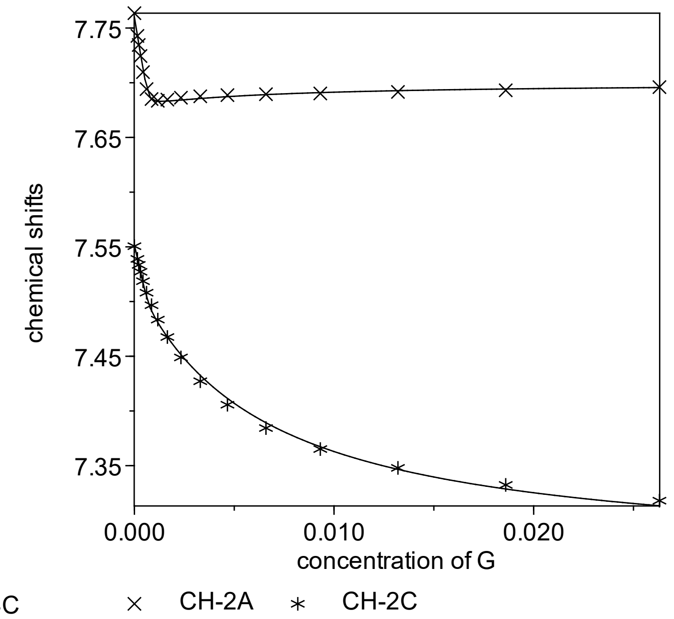

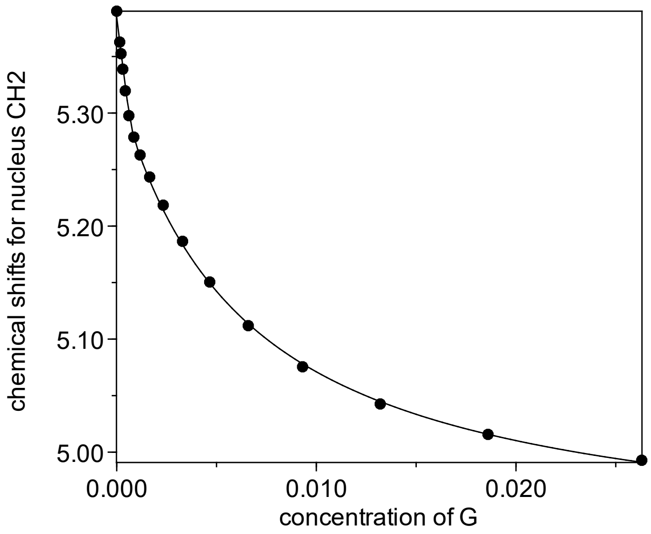

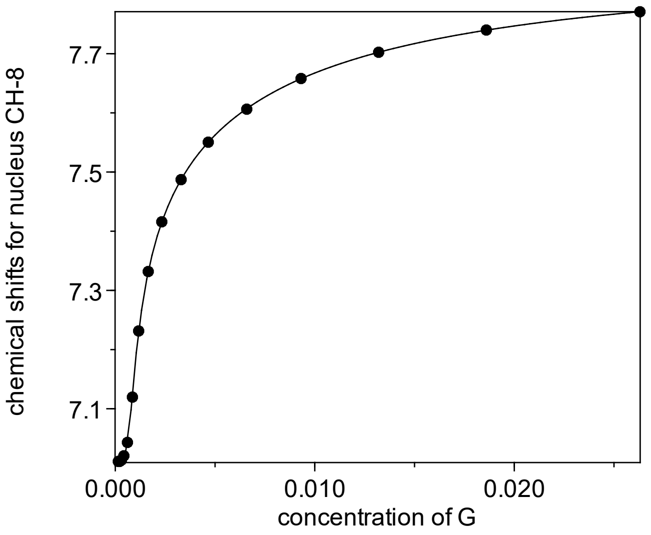

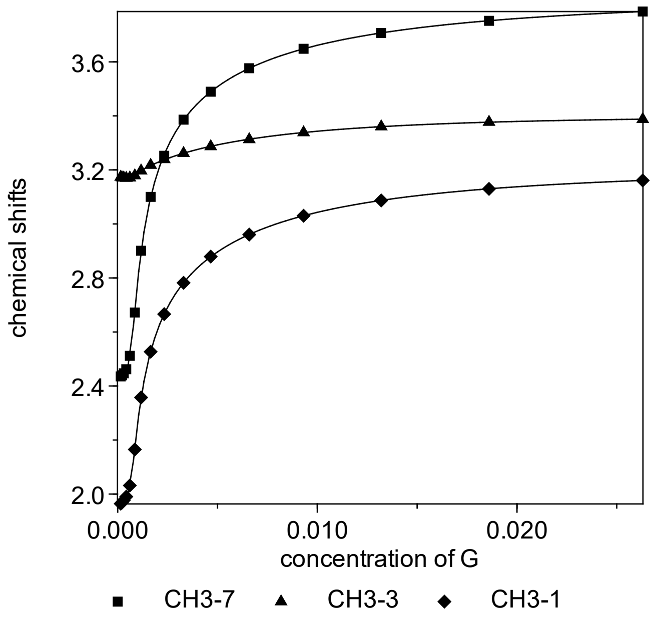
chemical shifts
3.6
3.2
2.8
2.4
2.0
0.000
0.010
0.020
concentration of G
CH3-7
CH3-3
CH3-1

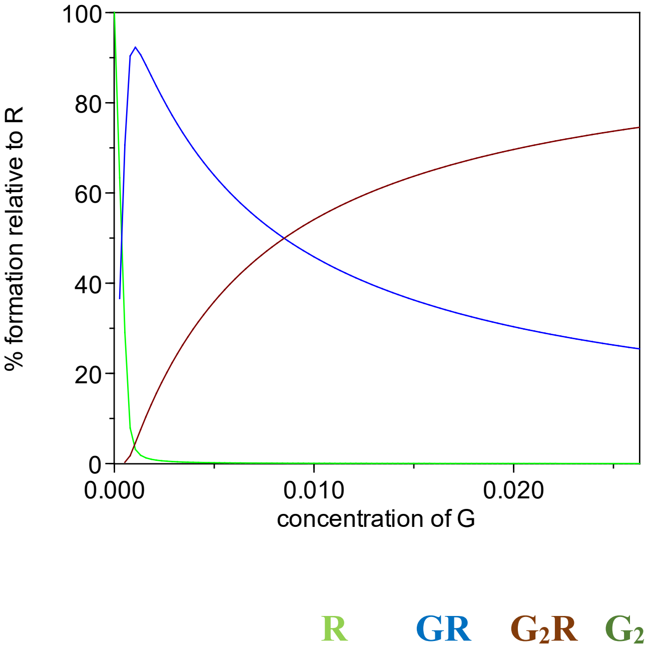
% formation relative to R
100
80
60
40
20
0
0.000
0.010
0.020
concentration of G
R
GR
G2R
G2

6 + Theophylline (D_2O, 298 K, 500 MHz)

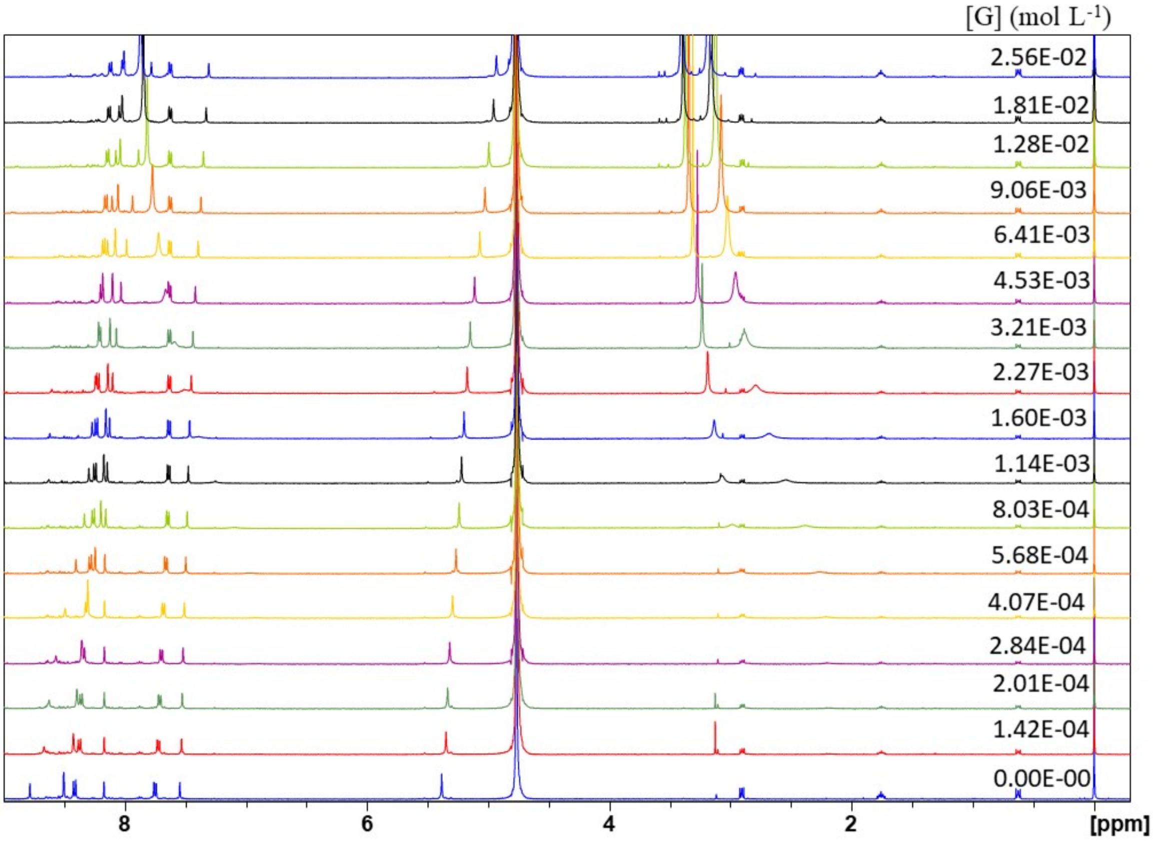

Figure S54. ^1H NMR spectroscopic titration (500 MHz, D_2O, 298 K) of receptor **6** (6.96 10^{-4} mol L^{-1}) with incremental concentrations of theophylline (G).

Data Table

R = **6**   G = Theophylline

δ (ppm) *vs*. [G] (mol L^{-1})

Titration [R] = 6.96 10^{-4} mol L^{-1}

[G]	CH-8 G	CH3-1 G	CH3-3 G	CH-10A R	CH-4A R
0.00E+00	-	-	-	8.7866	8.5072
1.42E-04	6.8950	2.8850	2.1850	8.6709	8.4279
2.01E-04	6.9040	2.8870	2.1890	8.6290	8.3994
2.84E-04	6.9130	2.8910	2.2040	8.5722	8.3597
4.07E-04	6.9240	2.9000	2.2140	8.4963	8.3093
5.68E-04	6.9730	2.9330	2.2650	8.4047	8.2484
8.03E-04	7.0986	2.9906	2.3886	8.3376	8.2017
1.14E-03	7.2617	3.0707	2.5488	8.3005	8.1774
1.60E-03	7.3973	3.1388	2.6850	8.2741	8.1605
2.27E-03	7.5039	3.1922	2.7964	8.2467	8.1438
3.21E-03	7.5930	3.2382	2.8884	8.2171	8.1249
4.53E-03	7.6648	3.2778	2.9639	8.1835	8.1039
6.41E-03	7.7250	3.3136	3.0260	8.1448	8.0817
9.06E-03	7.7759	3.3457	3.0811	8.1089	8.0600
1.28E-02	7.8177	3.3723	3.1262	8.0761	8.0405
1.81E-02	7.8502	3.3926	3.1614	8.0493	8.0242
2.56E-02	7.8750	3.4061	3.1877	8.0272	8.0111

[G]	CH-1A	CH-4C	CH-2A	CH-2C	CH2
	R	R	R	R	R
0.00E+00	8.4272	8.1759	7.7635	7.5503	5.3906
1.42E-04	8.3878	8.1744	7.7361	7.5356	5.3547
2.01E-04	8.3731	8.1738	7.7263	7.5303	5.3418
2.84E-04	8.3551	8.1730	7.7129	7.5232	5.3245
4.07E-04	8.3282	8.1716	7.6951	7.5134	5.3008
5.68E-04	8.2979	8.1685	7.6744	7.5010	5.2725
8.03E-04	8.2739	8.1618	7.6590	7.4899	5.2471
1.14E-03	8.2594	8.1488	7.6522	7.4800	5.2270
1.60E-03	8.2479	8.1300	7.6490	7.4693	5.2060
2.27E-03	8.2354	8.1047	7.6464	7.4560	5.1803
3.21E-03	8.2164	8.0720	7.6442	7.4393	5.1491
4.53E-03	8.2037	8.0331	7.6419	7.4196	5.1127
6.41E-03	8.1865	7.9884	7.6397	7.3981	5.0733
9.06E-03	8.1689	7.9398	7.6379	7.3755	5.0330
1.28E-02	8.1537	7.8895	7.6374	7.3532	4.9952
1.81E-02	8.1414	7.8393	7.6377	7.3322	4.9621
2.56E-02	8.1319	7.7859	7.6384	7.3114	4.9323

Results page

```
 no. of spectra            17
 no. of resonance values  167
 no. of resonant nuclei    10

Chi-squared =  19.40

   sigma =   0.00565350063          RMS weighted residual =   0.00502627418

       stoich            value   relative    log    standard
       coeff                     std devn    beta   deviation

 Beta  1  1 refined  3.0307E+004  0.0765    4.4815   0.0332  (   GR  )
 Beta  2  1 refined  2.7498E+006  0.1068    6.4393   0.0464  (   G2R )
 Beta  2  0 constant    6.4003              0.8062           (   G2  )

 Individual chemical shifts

                  G                       R
 =========================================================
        +       value    error         value    error
CH-8    +      7.8928   0.0463
CH3-1   +      3.4369   0.0463
CH3-3   +      3.2463   0.0467
CH-10A  +                             8.7678   0.0036
CH-4A   +                             8.4938   0.0036
CH-1A   +                             8.4218   0.0035
CH-4C   +                             8.1744   0.0036
CH-2A   +                             7.7589   0.0035
CH-2C   +                             7.5482   0.0036
CH2     +                             5.3861   0.0036
        +
                 1,1                     2,1
 =========================================================
        +       value    error         value    error
CH-8    +      6.8402   0.0066         6.4049   0.4266
CH3-1   +      2.8565   0.0044         2.2017   0.4266
CH3-3   +      2.1268   0.0069         1.1563   0.4405
CH-10A  +      8.2747   0.0038         7.8758   0.0109
CH-4A   +      8.1587   0.0033         7.9209   0.0081
CH-1A   +      8.2511   0.0030         8.0554   0.0075
CH-4C   +      8.1729   0.0033         7.5809   0.0152
CH-2A   +      7.6432   0.0029         7.6335   0.0061
CH-2C   +      7.4851   0.0030         7.2131   0.0088
CH2     +      5.2300   0.0033         4.7522   0.0126
        +
                 2,0
 =================================
        +       value    error
CH-8    +      8.1455   0.1260
CH3-1   +      3.5491   0.1260
CH3-3   +      3.3970   0.1264
CH-10A  +
CH-4A   +
CH-1A   +
CH-4C   +
CH-2A   +
CH-2C   +
CH2     +

 Correlation coefficients*1000


      1    2
   1
   2  870

 Parameters are numbered as follows
    1 beta 1,1
    2 beta 2,1
```

Titration Plots

Chemical shifts (δ, ppm) *vs*. concentration of G (mol L^{-1})

experimental (symbols) and calculated (lines) values

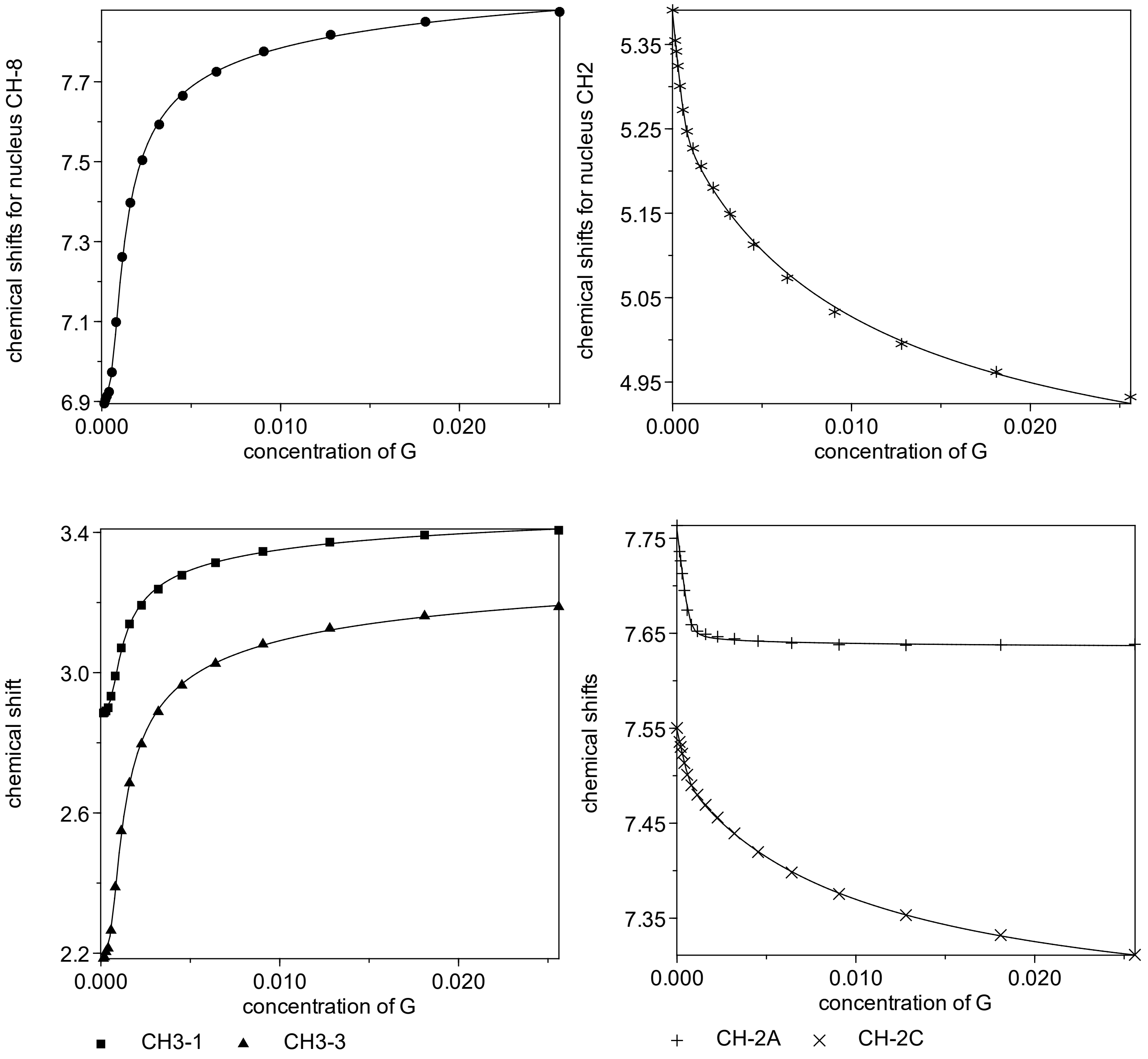

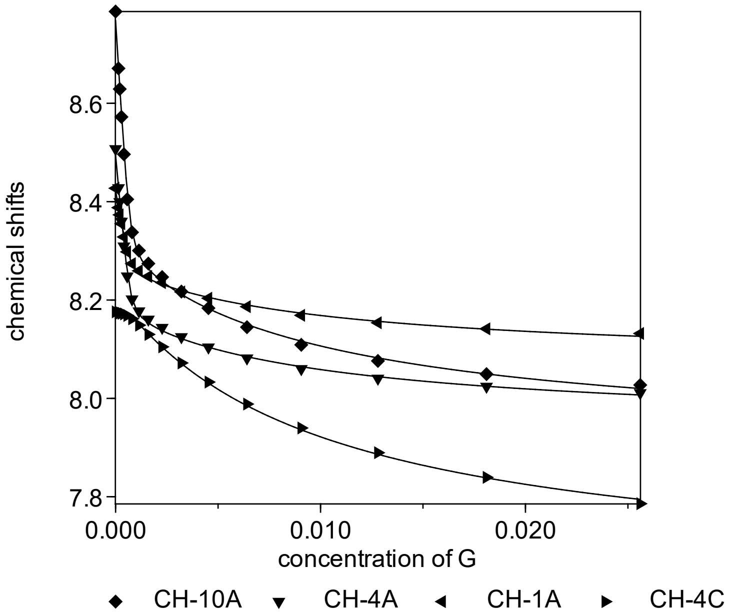

chemical shifts
8.6
8.4
8.2
8.0
7.8
0.000
0.010
0.020
concentration of G
CH-10A
CH-4A
CH-1A
CH-4C

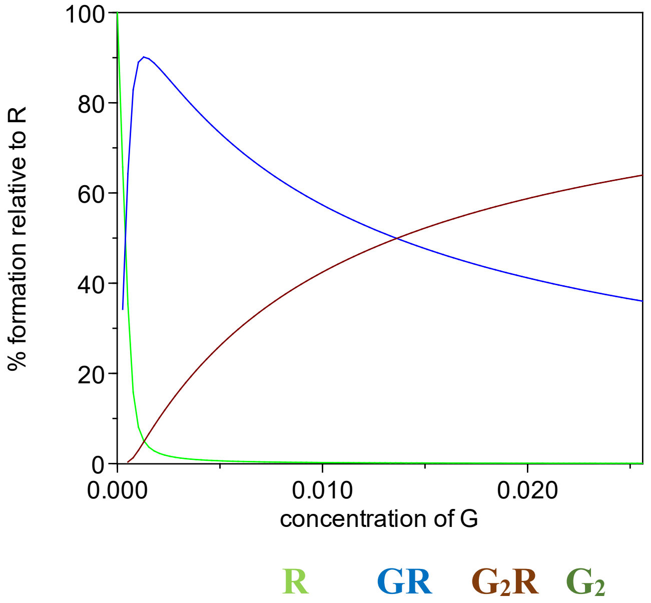

% formation relative to R
100
80
60
40
20
0
0.000
0.010
0.020
concentration of G
R
GR
G2R
G2

6 + Theobromine (D_2O, 298 K, 500 MHz)

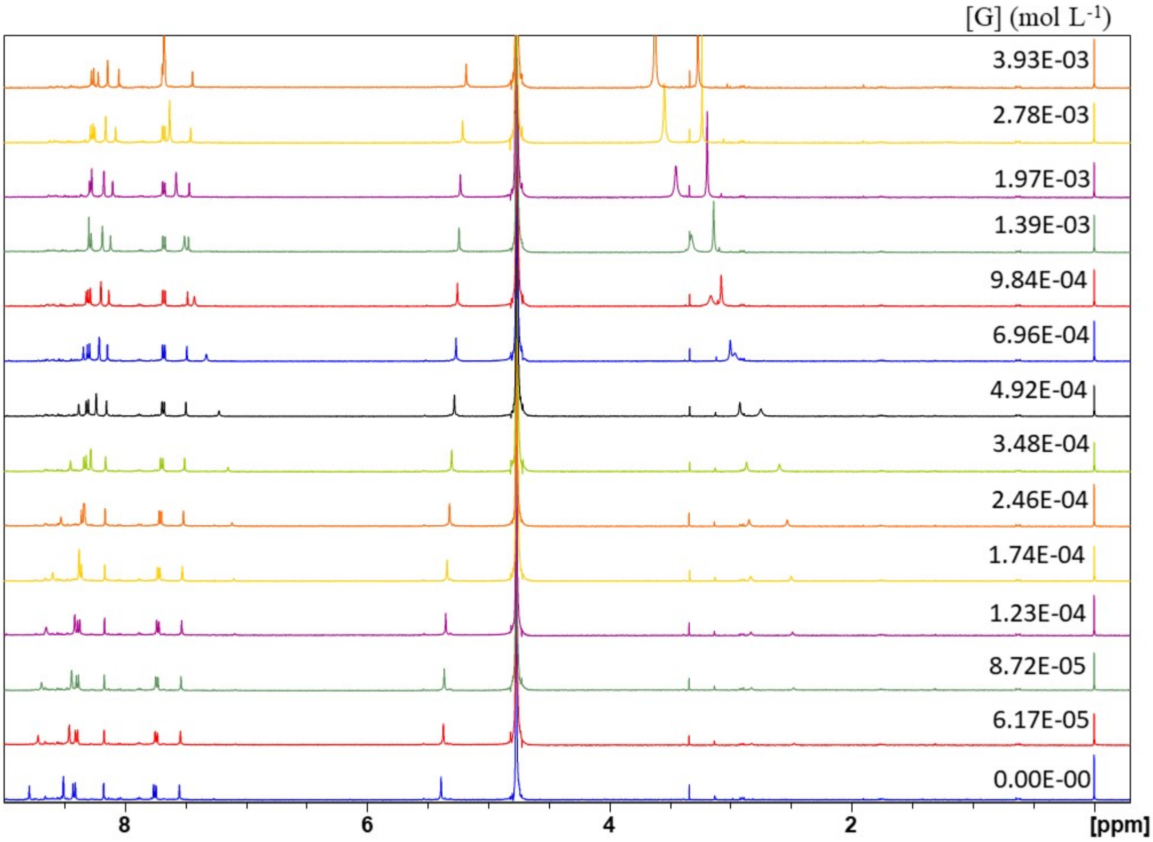

Figure S55. ^{1}H NMR spectroscopic titration (500 MHz, D_2O, 298 K) of receptor **6** (4.10 10^{-4} mol L^{-1}) with incremental concentrations of theobromine (G).

Data Table

R = **6** G = Theobromine

δ (ppm) *vs*. [G] (mol L^{-1})

Titration [R] = 4.10 10^{-4} mol L^{-1}

[G]	CH-8 G	CH3-7 G	CH3-3 G	CH-10A R	CH-4A R
0.00E+00	-	-	-	8.7917	8.5097
6.17E-05	7.0891	2.4748	2.8255	8.7194	8.4615
8.72E-05	7.0921	2.4805	2.8275	8.6908	8.4424
1.23E-04	7.0974	2.4872	2.8306	8.6523	8.4165
1.74E-04	7.1043	2.5022	2.8358	8.5991	8.3805
2.46E-04	7.1198	2.5312	2.8472	8.5304	8.3370
3.48E-04	7.1527	2.5989	2.8710	8.4518	8.2838
4.92E-04	7.2272	2.7541	2.9263	8.3840	8.2394
6.96E-04	7.3316	2.9695	3.0061	8.3461	8.2153
9.84E-04	7.4298	3.1661	3.0808	8.3222	8.2008
1.39E-03	7.5119	3.3278	3.1428	8.3003	8.1883
1.97E-03	7.5785	3.4530	3.1949	8.2755	8.1752
2.78E-03	7.6322	3.5499	3.2376	8.2514	8.1602
3.93E-03	7.6778	3.6263	3.2741	8.2207	8.1431

[G]	CH-1A	CH-4C	CH-2A	CH-2C	CH2
	R	R	R	R	R
0.00E+00	8.4304	8.1773	7.7650	7.5529	5.3932
6.17E-05	8.4112	8.1745	7.7529	7.5439	5.3745
8.72E-05	8.4037	8.1732	7.7482	7.5403	5.3671
1.23E-04	8.3936	8.1715	7.7415	7.5352	5.3572
1.74E-04	8.3797	8.1691	7.7332	7.5288	5.3437
2.46E-04	8.3618	8.1661	7.7213	7.5210	5.3261
3.48E-04	8.3410	8.1619	7.7084	7.5096	5.3055
4.92E-04	8.3229	8.1560	7.6977	7.5001	5.2864
6.96E-04	8.3128	8.1477	7.6927	7.4931	5.2727
9.84E-04	8.3062	8.1365	7.6910	7.4863	5.2605
1.39E-03	8.2997	8.1216	7.6904	7.4787	5.2472
1.97E-03	8.2931	8.1026	7.6907	7.4695	5.2309
2.78E-03	8.2855	8.0788	7.6910	7.4584	5.2114
3.93E-03	8.2770	8.0500	7.6915	7.4453	5.1887

Results page

```
 no. of spectra            14
 no. of resonance values  137
 no. of resonant nuclei    10

Chi-squared =  53.54

   sigma =   0.00168797626          RMS weighted residual =   0.00145648541

       stoich            value   relative    log    standard
       coeff                     std devn    beta   deviation
 Beta  1  1 refined  4.6804E+004  0.0245    4.6703   0.0106  (   GR   )
 Beta  2  1 refined  2.9728E+006  0.2356    6.4732   0.1023  (   G2R  )
 Beta  2  0 constant    5.5976              0.7480           (   G2   )

 Individual chemical shifts

                  G                        R
 =========================================================
        +       value    error         value    error
CH-8    +       9.1920   0.6831
CH3-7   +       6.3682   1.0847
CH3-3   +       4.6934   0.6550
CH-10A  +                              8.7840   0.0010
CH-4A   +                              8.5041   0.0010
CH-1A   +                              8.4283   0.0010
CH-4C   +                              8.1774   0.0010
CH-2A   +                              7.7637   0.0010
CH-2C   +                              7.5522   0.0010
CH2     +                              5.3913   0.0010
        +
                 1,1                       2,1
 =========================================================
        +       value    error         value    error
CH-8    +       6.9764   0.0368      -22.0407  18.9472
CH3-7   +       2.2615   0.0593      -49.1649  32.0039
CH3-3   +       2.7248   0.0351      -24.6954  18.0051
CH-10A  +       8.3237   0.0015        7.7169   0.1105
CH-4A   +       8.1993   0.0012        7.8700   0.0605
CH-1A   +       8.3072   0.0010        8.1284   0.0340
CH-4C   +       8.1570   0.0011        7.5330   0.1145
CH-2A   +       7.6869   0.0010        7.7139   0.0115
CH-2C   +       7.4938   0.0010        7.2092   0.0529
CH2     +       5.2727   0.0011        4.7795   0.0905
        +
                 2,0
 =================================
        +       value    error
CH-8    +       3.0503   2.1969
CH3-7   +      -5.6757   2.9891
CH3-3   +      -1.2288   2.1438
CH-10A  +
CH-4A   +
CH-1A   +
CH-4C   +
CH-2A   +
CH-2C   +
CH2     +

 Correlation coefficients*1000


      1    2
  1
  2  530

 Parameters are numbered as follows
    1 beta 1,1
    2 beta 2,1
```

Titration Plots

Chemical shifts (δ, ppm) *vs*. concentration of G (mol L^{-1})

experimental (symbols) and calculated (lines) values

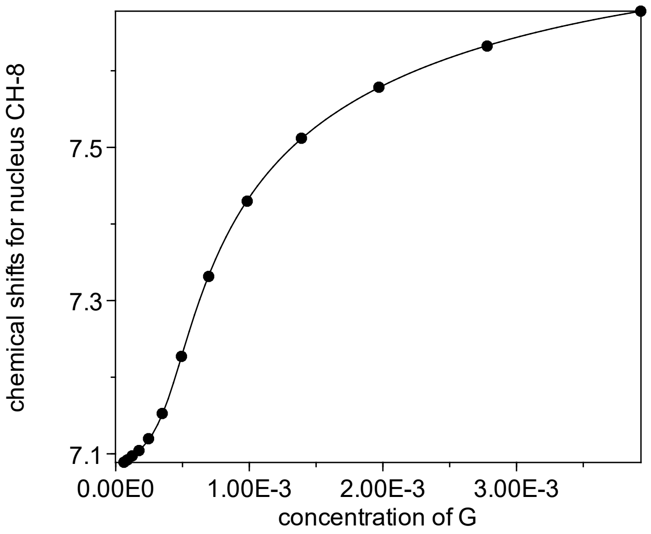

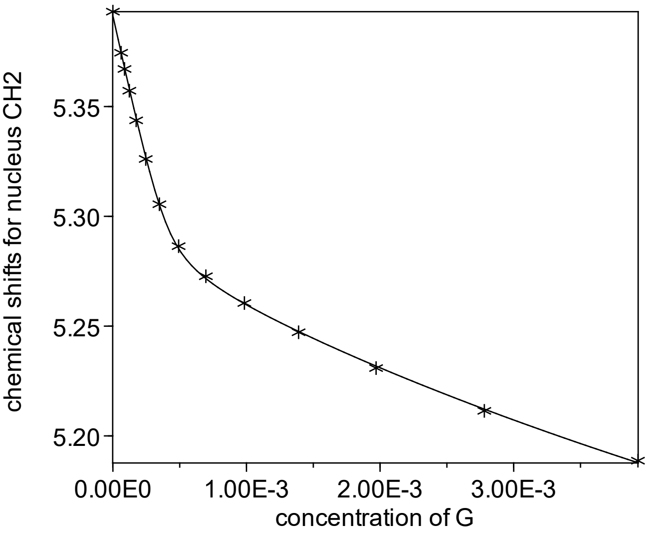

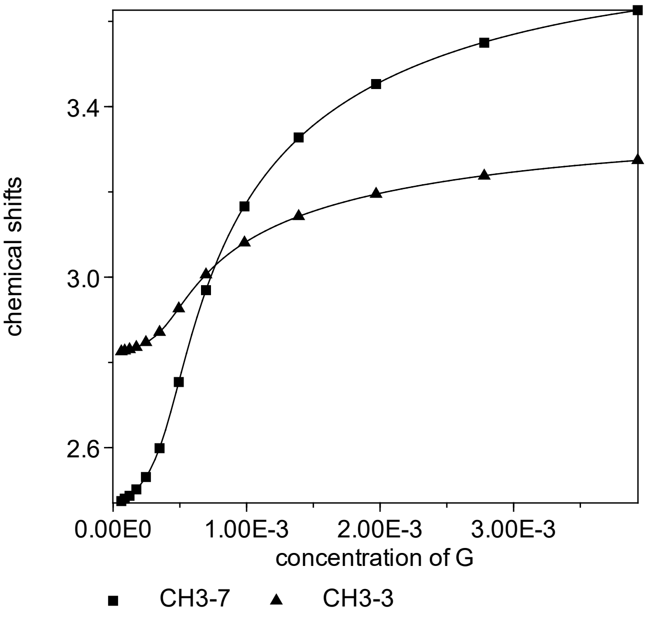

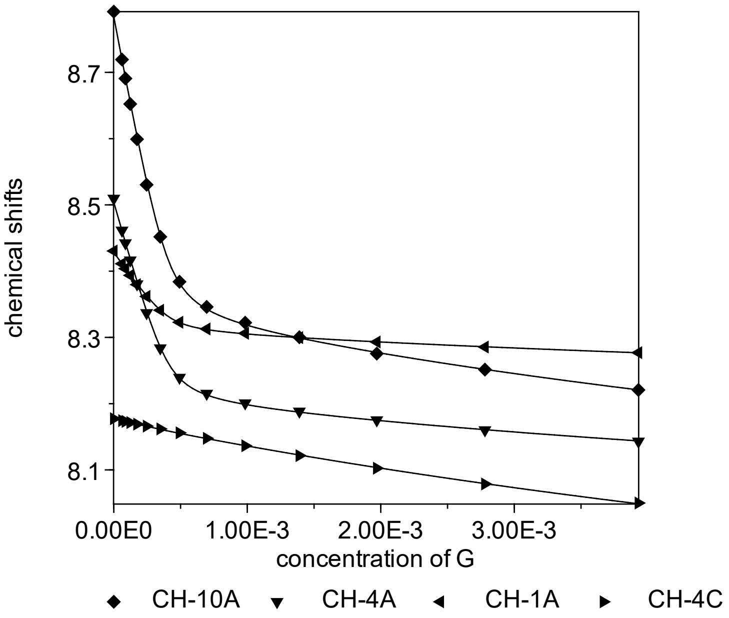

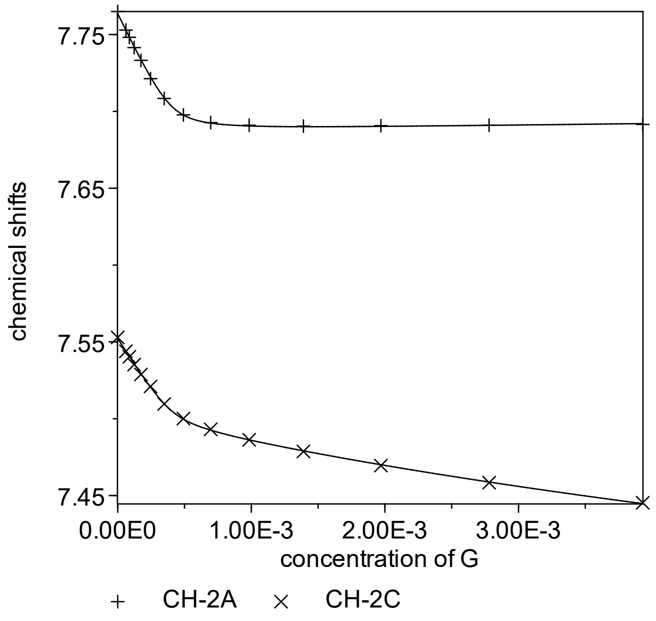

7.75
7.65
7.55
7.45
chemical shifts
0.00E0
1.00E-3
2.00E-3
3.00E-3
concentration of G
CH-2A
CH-2C

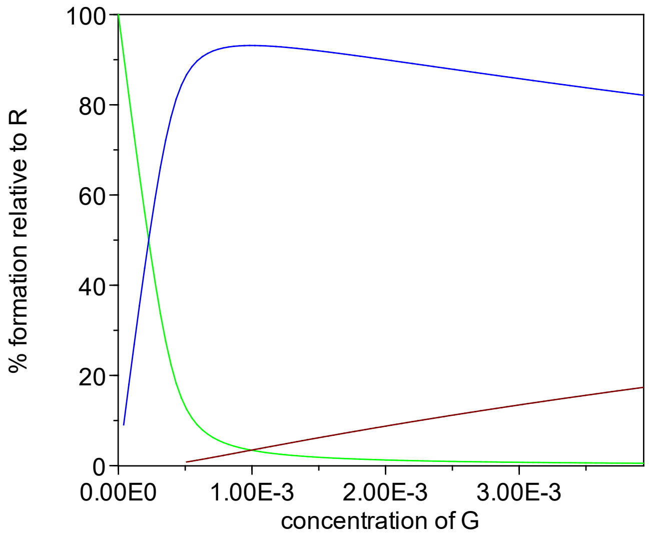

100
80
60
40
20
0
% formation relative to R
0.00E0
1.00E-3
2.00E-3
3.00E-3
concentration of G

R **GR** **G_2R** **G_2**

Ultraviolet/visible (UV/Vis) experiments. UV/Vis experiments were carried out at 298 K in H_2O using a quartz cuvette (3 mL, 10 mm path length). Preliminary titration of receptor **2** with caffeine was performed on a weighted amount of caffeine dissolved in a stock solution of receptor **2** to obtain 2.99 mM solution of caffeine in 2.95 μM of **2** in H_2O. 3 mL of the latter solution were loaded into the cuvette and the spectrum was registered. Before each further spectrum, 1.5 mL were syringed out from the cuvette and 1.5 mL of the stock solution of **2** were added.

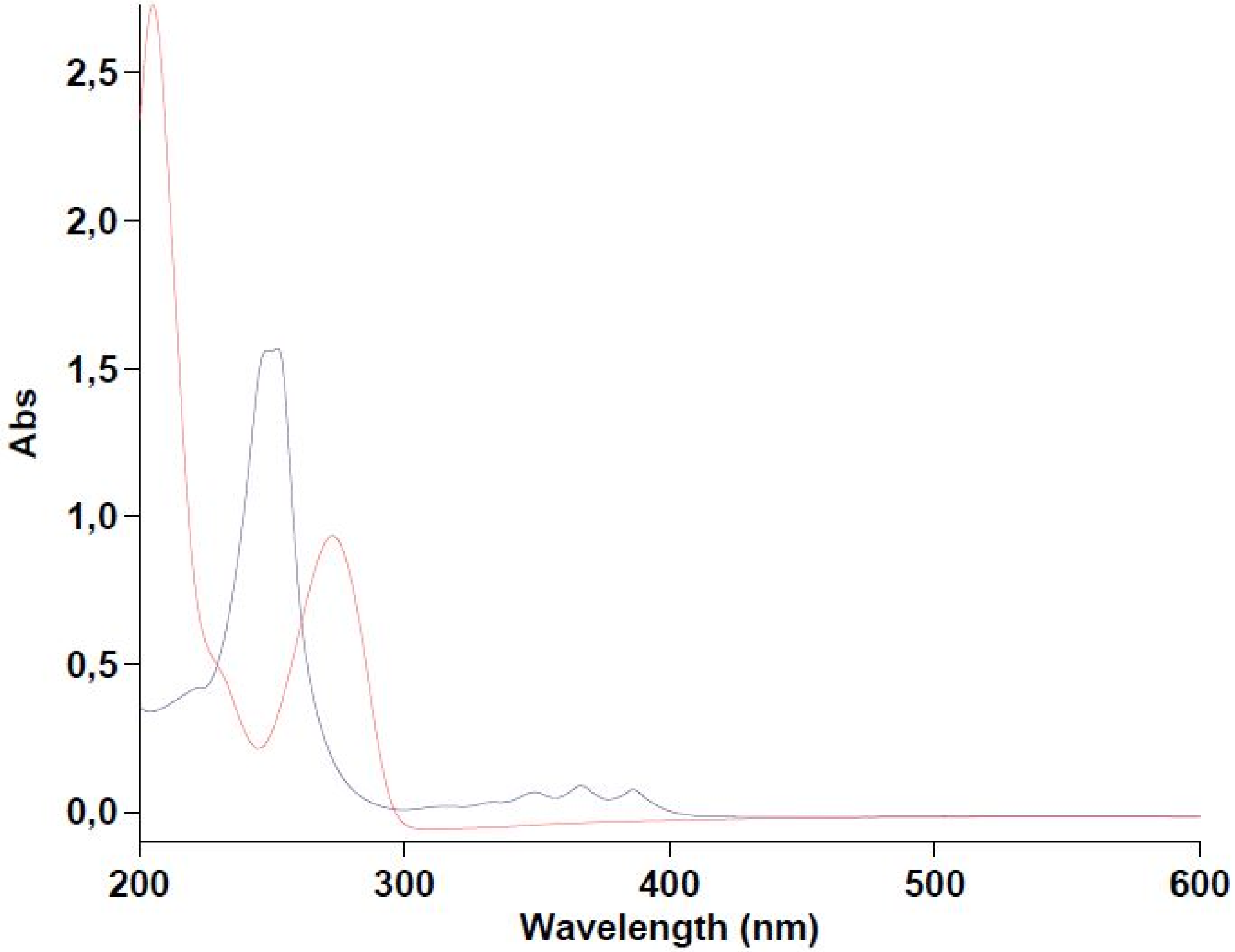

Figure S56. Superposition of UV/Vis spectra of **2** (4.92 μM, λ_{max} at 252 nm) and caffeine (46.9 μM, λ_{max} at 273 nm) at 298 K in H_2O.

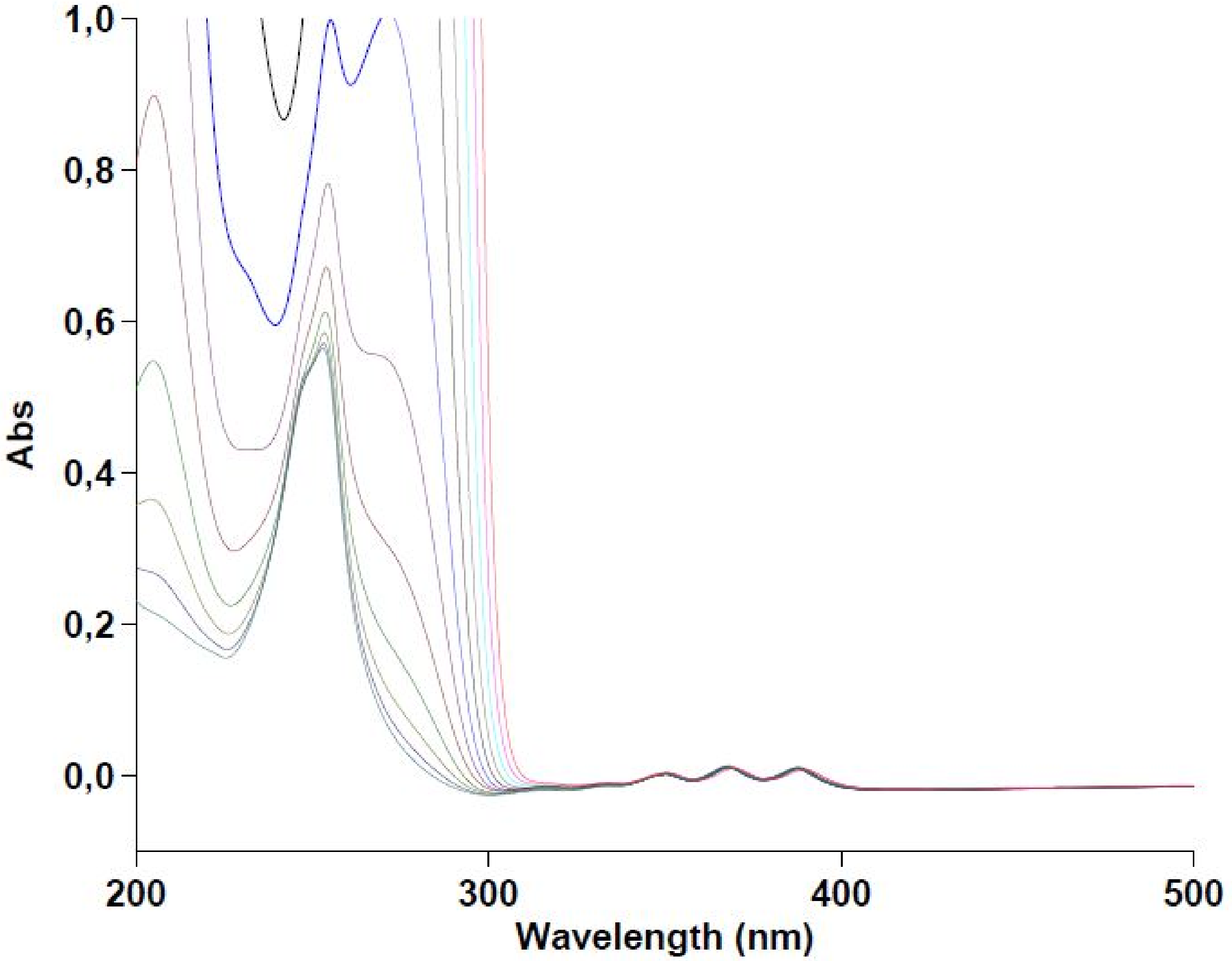

Figure S57. UV/Vis titration at 298 K in H_2O of **2** (2.95 μM) with increasing amounts of caffeine (from 0.720 μM to 2.99 mM). A poor variation of absorbance was observed for receptor **2** in the non-overlapping region over 300 nm.

Fluorescence experiments. Fluorescence experiments were carried out at 298 K in H_2O on a quartz cuvette (3 mL, 10 mm path length). Excitation wavelength (345 nm) was chosen because the receptor **2** absorption was nearly independent from caffeine concentration. A preliminary fluorescence titration of receptor **2** with caffeine was carried out starting from a weighted amount of caffeine dissolved in a stock solution of receptor **2** to obtain 2.68 mM solution of caffeine in 98.3 μM of **2** in H_2O, 3 mL of which were loaded inside the cuvette and spectrum registered. Before each further spectrum, 1.5 mL were syringed out from the cuvette and 1.5 mL of the stock solution of **2** added and the mixture mixed.

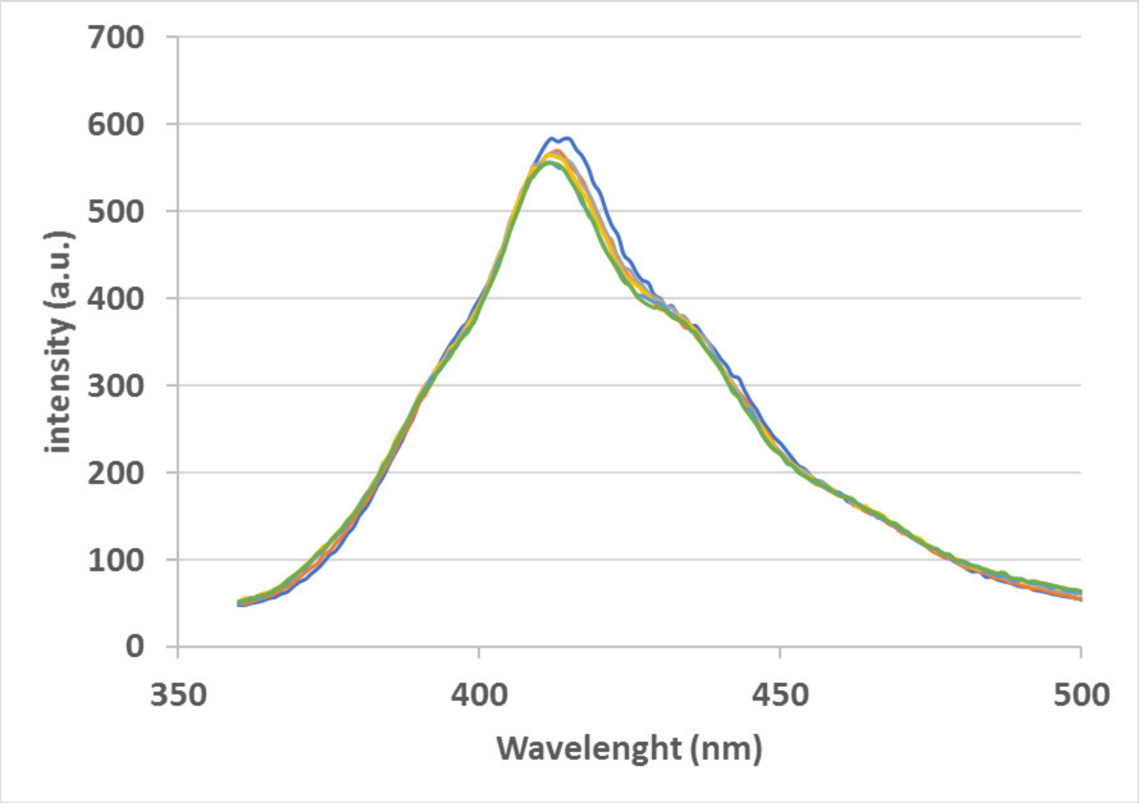

Figure S58. Fluorescence titration at 298 K in H_2O of **2** (98.3 μM) with increasing amounts of caffeine (83.8 μM to 2.68 mM). Excitation wavelength 345 nm.

Calorimetric titrations and data analysis. Isothermal Titration Microcalorimetry experiments were performed at 298 K with a Nano-ITC instrument. After an initial injection of 3 μL, which was excluded from data analysis, aliquots of the titrant solution were injected stepwise into the sample cell containing a solution of the titrate. All experiments were performed in H_2O at pH 7. Heats of dilution were measured by injecting the titrant solution into neat H_2O and then subtracted from the binding heats. To remove ambiguities in the definition of the binding model for receptor **2**, two to four independent titrations run at different reactant concentrations were combined into a simultaneous fit of all data. The receptor **2** and xanthines dimerization constants, measured by NMR spectroscopy, were set invariant in the non-linear regression analysis of receptor **2** binding data. Cumulative association constants and the thermodynamic parameters were calculated using the HypCal software package.[S5]

2 + Caffeine (H_2O, 298 K).

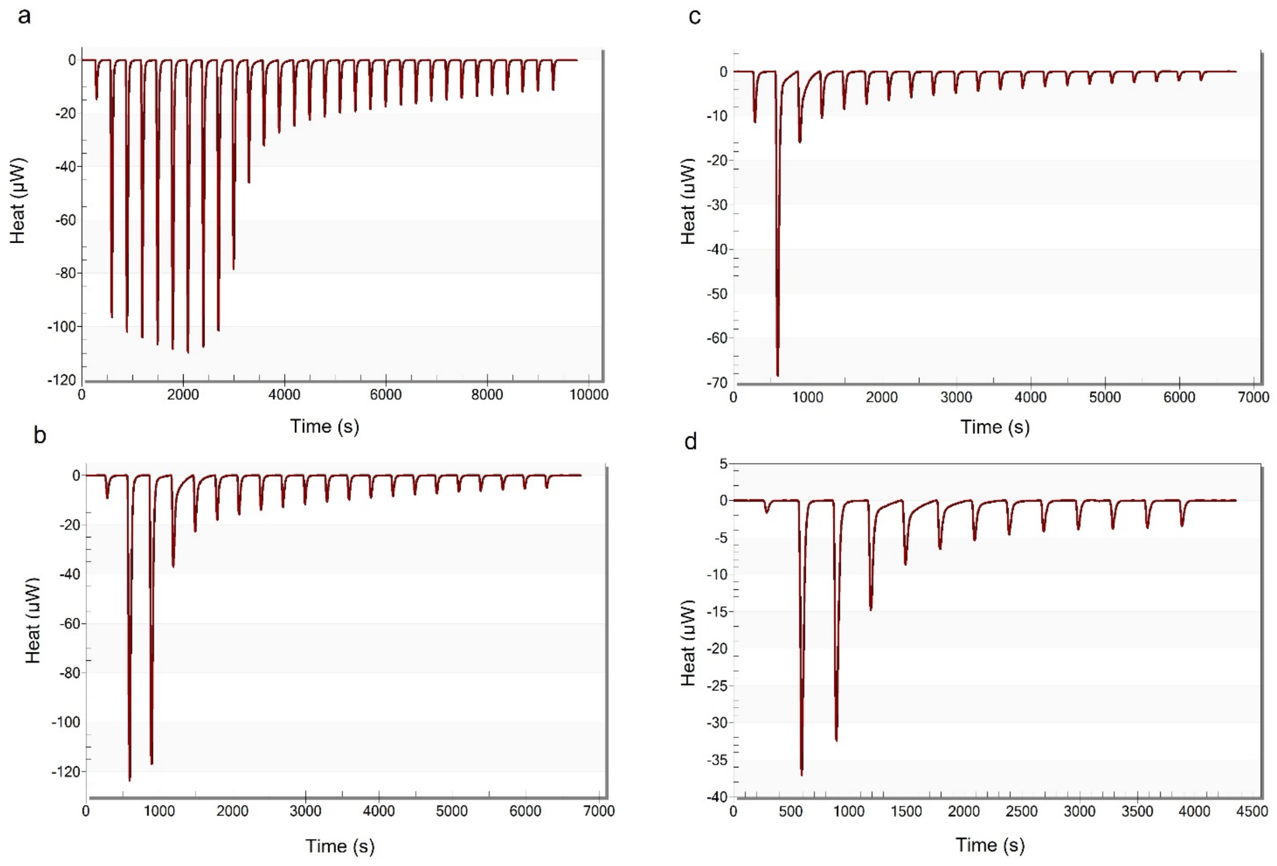

Figure S59. ITC results of Caffeine with **2** in H_2O at 298 K: a) Titration of **2** ($1.00\ 10^{-3}$ mol L^{-1}) with Caffeine ($1.01\ 10^{-2}$ mol L^{-1}); b) Titration of **2** ($4.00\ 10^{-4}$ mol L^{-1}) with Caffeine ($1.01\ 10^{-2}$ mol L^{-1}); c) Titration of **2** ($2.00\ 10^{-4}$ mol L^{-1}) with Caffeine ($1.01\ 10^{-2}$ mol L^{-1}); d) Titration of **2** ($2.00\ 10^{-4}$ mol L^{-1}) with Caffeine ($2.00\ 10^{-3}$ mol L^{-1}).

Data Table

R = **2** G = Caffeine

[G] = 4.18 10^{-2} mol L^{-1}

Titration 1: [R] = 1.00 10^{-3} mol L^{-1}

Injection	Q (μJ)	Corrected Q (μJ)	inj volume (μL)	mol G (mol)	mol R (mol)	mol G / mol R	total volume (μL)
1	-537.642	-548.837	3	3.03E-08	9.42E-07	0.032166	945
2	-3231.16	-3308.29	8	1.11E-07	9.34E-07	0.118673	945
3	-3443.73	-3518.56	8	1.91E-07	9.26E-07	0.205919	945
4	-3606.92	-3682.72	8	2.70E-07	9.18E-07	0.29391	945
5	-3753.67	-3826.81	8	3.48E-07	9.11E-07	0.382652	945
6	-3868.63	-3940.82	8	4.26E-07	9.03E-07	0.472151	945
7	-3962.64	-4032.66	8	5.03E-07	8.95E-07	0.562415	945
8	-3985.6	-4053.69	8	5.80E-07	8.88E-07	0.65345	945
9	-3837.19	-3900.16	8	6.56E-07	8.80E-07	0.745261	945
10	-3119.96	-3183.21	8	7.31E-07	8.73E-07	0.837857	945
11	-1907.07	-1970.63	8	8.06E-07	8.65E-07	0.931243	945
12	-1391.28	-1451.33	8	8.80E-07	8.58E-07	1.025427	945
13	-1117.29	-1177.78	8	9.53E-07	8.51E-07	1.120414	945
14	-934.949	-995.072	8	1.03E-06	8.43E-07	1.216213	945
15	-822.744	-879.707	8	1.10E-06	8.36E-07	1.312829	945
16	-757.849	-815.263	8	1.17E-06	8.29E-07	1.410271	945
17	-710.669	-763.956	8	1.24E-06	8.22E-07	1.508544	945
18	-676.84	-731.169	8	1.31E-06	8.15E-07	1.607657	945
19	-642.661	-697.712	8	1.38E-06	8.08E-07	1.707615	945
20	-610.674	-663.177	8	1.45E-06	8.01E-07	1.808427	945
21	-586.682	-638.43	8	1.52E-06	7.95E-07	1.9101	945
22	-564.13	-611.97	8	1.59E-06	7.88E-07	2.012641	945
23	-536.361	-584.412	8	1.65E-06	7.81E-07	2.116057	945
24	-509.311	-556.07	8	1.72E-06	7.75E-07	2.220357	945
25	-500.197	-545.756	8	1.79E-06	7.68E-07	2.325547	945
26	-473.66	-519.53	8	1.85E-06	7.62E-07	2.431635	945
27	-450.532	-488.469	8	1.92E-06	7.55E-07	2.538628	945
28	-437.522	-470.925	8	1.98E-06	7.49E-07	2.646535	945
29	-415.93	-458.64	8	2.05E-06	7.42E-07	2.755364	945
30	-405.223	-446.189	8	2.11E-06	7.36E-07	2.865121	945
31	-381.555	-416.715	8	2.17E-06	7.30E-07	2.975816	945

Titration 2: [R] = 4.00 10^{-4} mol L^{-1}

Injection	Q (μJ)	Corrected Q (μJ)	inj volume (μL)	mol G (mol)	mol R (mol)	mol G / mol R	total volume (μL)
1	-374.452	-383.674	3	3.03E-08	4.71E-07	0.064331	945
2	-4637.28	-4751.45	12	1.51E-07	4.65E-07	0.324966	945
3	-4494.43	-4606.42	12	2.70E-07	4.59E-07	0.588952	945
4	-2051.15	-2160.53	12	3.88E-07	4.53E-07	0.856334	945
5	-1078.38	-1185.15	12	5.04E-07	4.48E-07	1.127155	945
6	-668.529	-772.101	12	6.19E-07	4.42E-07	1.40146	945
7	-546.68	-645.615	12	7.33E-07	4.36E-07	1.679292	945
8	-481.672	-576.557	12	8.44E-07	4.31E-07	1.960698	945
9	-436.375	-528.397	12	9.55E-07	4.25E-07	2.245723	945
10	-395.287	-483.983	12	1.06E-06	4.20E-07	2.534414	945
11	-364.886	-449.948	12	1.17E-06	4.14E-07	2.826818	945
12	-332.956	-413.831	12	1.28E-06	4.09E-07	3.122983	945
13	-310.74	-391.175	12	1.38E-06	4.04E-07	3.422957	945
14	-283.732	-362.164	12	1.49E-06	3.99E-07	3.726789	945
15	-263.477	-336.862	12	1.59E-06	3.94E-07	4.034529	945
16	-240.884	-312.1	12	1.69E-06	3.89E-07	4.346227	945
17	-217.242	-288.224	12	1.79E-06	3.84E-07	4.661934	945
18	-210.777	-287.384	12	1.89E-06	3.79E-07	4.981702	945
19	-195.834	-258.7	12	1.99E-06	3.74E-07	5.305582	945
20	-181.398	-242.022	12	2.08E-06	3.69E-07	5.633628	945
21	-174.938	-234.278	12	2.18E-06	3.65E-07	5.965894	945

Titration 3: [R] = 2.00 10^{-4} mol L^{-1}

Injection	Q (μJ)	Corrected Q (μJ)	inj volume (μL)	mol G (mol)	mol R (mol)	mol G / mol R	total volume (μL)
1	-417.825	-427.047	3	3.03E-08	1.88E-07	0.160828	945
2	-2923.36	-3037.54	12	1.51E-07	1.86E-07	0.812414	945
3	-1111.93	-1223.93	12	2.70E-07	1.84E-07	1.472381	945
4	-436.901	-546.274	12	3.88E-07	1.81E-07	2.140836	945
5	-304.308	-411.077	12	5.04E-07	1.79E-07	2.817889	945
6	-261.922	-365.494	12	6.19E-07	1.77E-07	3.503649	945
7	-230.004	-328.94	12	7.33E-07	1.74E-07	4.19823	945
8	-207.973	-302.858	12	8.44E-07	1.72E-07	4.901744	945
9	-187.351	-279.373	12	9.55E-07	1.70E-07	5.614307	945
10	-171.62	-260.317	12	1.06E-06	1.68E-07	6.336034	945
11	-156.911	-241.972	12	1.17E-06	1.66E-07	7.067044	945
12	-142.701	-223.577	12	1.28E-06	1.64E-07	7.807456	945
13	-127.739	-208.174	12	1.38E-06	1.62E-07	8.557392	945
14	-117.096	-195.528	12	1.49E-06	1.60E-07	9.316972	945
15	-107.828	-181.214	12	1.59E-06	1.58E-07	10.08632	945
16	-99.646	-170.862	12	1.69E-06	1.56E-07	10.86557	945
17	-89.4092	-160.392	12	1.79E-06	1.54E-07	11.65484	945
18	-85.6968	-162.303	12	1.89E-06	1.52E-07	12.45425	945
19	-67.4644	-130.33	12	1.99E-06	1.50E-07	13.26396	945
20	-73.1507	-133.775	12	2.08E-06	1.48E-07	14.08407	945
21	-69.2855	-128.625	12	2.18E-06	1.46E-07	14.91473	945

Titration 4: [R] = 2.00 10^{-4} mol L^{-1} [G] = 2.00 10^{-3} mol L^{-1}

Injection	Q (μJ)	Corrected Q (μJ)	inj volume (μL)	mol G (mol)	mol R (mol)	mol G / mol R	total volume (μL)
1	-61.9445	-59.4038	3	6.00E-09	1.88E-07	0.031847	945
2	-1242.66	-1205.82	20	4.59E-08	1.84E-07	0.248752	945
3	-1172.17	-1139.34	20	8.49E-08	1.81E-07	0.470347	945
4	-666.282	-634.407	20	1.23E-07	1.77E-07	0.696732	945
5	-485.836	-455.522	20	1.60E-07	1.73E-07	0.928013	945
6	-333.515	-305.721	20	1.97E-07	1.69E-07	1.164295	945
7	-233.278	-203.378	20	2.33E-07	1.66E-07	1.405685	945
8	-177.064	-148.181	20	2.68E-07	1.62E-07	1.652294	945
9	-145.044	-115.725	20	3.02E-07	1.59E-07	1.904236	945
10	-127.122	-106.224	20	3.36E-07	1.55E-07	2.161624	945
11	-131.466	-99.1631	20	3.69E-07	1.52E-07	2.424578	945
12	-122.15	-88.9668	20	4.01E-07	1.49E-07	2.693218	945
13	-118.435	-86.2282	20	4.33E-07	1.46E-07	2.967666	945

Results page

```
Reagent Reagent
  number   name
    1      R
    2      G

Chi-squared =  18.88

   sigma =   0.19616

 Formation constants   Value    relative    log    standard
                                std devn    beta   deviation
 Beta A constant    0.4732E+05           4.6750    0.1259    2  0
 Beta B constant    0.1148E+13          12.0599    0.2659    4  0
 Beta C constant    0.5994E+01           0.7777    0.0081    0  2
 Beta D refined     0.2227E+06  0.4856   5.3477    0.2109    1  1
 Beta E refined     0.4369E+10  0.7268   9.6403    0.3157    2  1

 Formation entalpies    Value   standard
                                deviation
-DeltaH A refined    -727.8492  80.5487
-DeltaH B refined   -1131.8681 124.1974
-DeltaH C refined     -65.6989   9.0032
-DeltaH D refined    -264.0881  33.0957
-DeltaH E refined    -612.3771  72.8369

+++++++++++++++++++++++++++++++++++++++++++++++++++++++++++++++++++++++++++++++
Thermodynamic Functions, kJ/mol
              - DeltaG°                 - DeltaH°                 T DeltaS°
   A       26.6850    1.2037      -727.8492   80.5487       754.5342   80.5577
   B       68.8381    1.8017     -1131.8681  124.1974      1200.7062  124.2105
   C        4.4391    0.2743       -65.6989    9.0032        70.1380    9.0074
   D       30.5249    0.2720      -264.0881   33.0957       294.6130   33.2945
   E       55.0272    0.3397      -612.3771   72.8369       667.4042   73.0785
 ++++++++++++++++++++++++++++++++++++++++++++++++++++++++++++++++++++++++++++++

 Correlation coefficients*1000         Run timed at 15.22 on 16 Jan 2019
   2  970
   3 -728 -677
   4 -766 -738  973
   5   77  -54  -74  -59
   6 -729 -690  997  980  -29
   7 -752 -710  997  986  -62  997
       1    2    3    4    5    6

 Order of parameters:
   1       Beta   D
   2       Beta   E
   3      -DeltaH A
   4      -DeltaH B
   5      -DeltaH C
   6      -DeltaH D
   7      -DeltaH E
```

Results table

Addition (μL)	Qobs (mJ)	Qcalc (mJ)	residual (mJ)	QTobs (mJ)	QTcalc (mJ)
1.3300	-0.5488				
9.3300	-3.3083	-3.2885	-0.0198	-3.8571	-3.8374
17.3300	-3.5186	-3.4747	-0.0439	-7.3757	-7.3120
25.3300	-3.6827	-3.6478	-0.0349	-11.0584	-10.9598
33.3300	-3.8268	-3.7870	-0.0398	-14.8852	-14.7468
41.3300	-3.9408	-3.8580	-0.0828	-18.8260	-18.6049
49.3300	-4.0327	-3.8134	-0.2192	-22.8587	-22.4183
57.3300	-4.0537	-3.6104	-0.4433	-26.9124	-26.0287
65.3300	-3.9002	-3.2510	-0.6491	-30.8126	-29.2797
73.3300	-3.1832	-2.8021	-0.3811	-33.9958	-32.0818
81.3300	-1.9706	-2.3473	0.3766	-35.9664	-34.4291
89.3300	-1.4513	-1.9395	0.4882	-37.4177	-36.3685
97.3300	-1.1778	-1.5990	0.4212	-38.5955	-37.9675
105.3300	-0.9951	-1.3262	0.3311	-39.5906	-39.2937
113.3300	-0.8797	-1.1123	0.2326	-40.4703	-40.4060
121.3300	-0.8153	-0.9456	0.1304	-41.2855	-41.3516
129.3300	-0.7640	-0.8153	0.0514	-42.0495	-42.1670
137.3300	-0.7312	-0.7126	-0.0186	-42.7807	-42.8796
145.3300	-0.6977	-0.6307	-0.0670	-43.4784	-43.5103
153.3300	-0.6632	-0.5646	-0.0986	-44.1416	-44.0748
161.3300	-0.6384	-0.5104	-0.1280	-44.7800	-44.5853
169.3300	-0.6120	-0.4656	-0.1464	-45.3920	-45.0508
177.3300	-0.5844	-0.4280	-0.1565	-45.9764	-45.4788
185.3300	-0.5561	-0.3960	-0.1601	-46.5324	-45.8748
193.3300	-0.5458	-0.3686	-0.1772	-47.0782	-46.2434
201.3300	-0.5195	-0.3448	-0.1747	-47.5977	-46.5882
209.3300	-0.4885	-0.3240	-0.1645	-48.0862	-46.9122
217.3300	-0.4709	-0.3055	-0.1654	-48.5571	-47.2177
225.3300	-0.4586	-0.2891	-0.1695	-49.0158	-47.5068
233.3300	-0.4462	-0.2744	-0.1718	-49.4620	-47.7812
241.3300	-0.4167	-0.2610	-0.1557	-49.8787	-48.0422
0.9700	-0.3837				
12.9700	-4.7515	-5.3637	0.6122	-5.1351	-5.7473
24.9700	-4.6064	-4.3283	-0.2781	-9.7415	-10.0756
36.9700	-2.1605	-2.5138	0.3532	-11.9021	-12.5894
48.9700	-1.1852	-1.3597	0.1745	-13.0872	-13.9491
60.9700	-0.7721	-0.8681	0.0960	-13.8593	-14.8172
72.9700	-0.6456	-0.6461	5.0000e-4	-14.5049	-15.4633
84.9700	-0.5766	-0.5305	-0.0460	-15.0815	-15.9938
96.9700	-0.5284	-0.4617	-0.0667	-15.6099	-16.4556
108.9700	-0.4840	-0.4160	-0.0679	-16.0939	-16.8716
120.9700	-0.4499	-0.3829	-0.0670	-16.5438	-17.2545
132.9700	-0.4138	-0.3573	-0.0566	-16.9577	-17.6118
144.9700	-0.3912	-0.3363	-0.0549	-17.3488	-17.9481
156.9700	-0.3622	-0.3184	-0.0438	-17.7110	-18.2664
168.9700	-0.3369	-0.3026	-0.0342	-18.0479	-18.5691
180.9700	-0.3121	-0.2884	-0.0237	-18.3600	-18.8575
192.9700	-0.2882	-0.2754	-0.0128	-18.6482	-19.1330
204.9700	-0.2874	-0.2633	-0.0240	-18.9356	-19.3963
216.9700	-0.2587	-0.2520	-6.7000e-3	-19.1943	-19.6482
228.9700	-0.2420	-0.2412	-8.0000e-4	-19.4363	-19.8894

240.9700	-0.2343	-0.2309	-3.4000e-3	-19.6706	-20.1203
1.6900	-0.4270				
13.6900	-3.0375	-3.2400	0.2024	-3.4646	-3.6670
25.6900	-1.2239	-0.8759	-0.3480	-4.6885	-4.5430
37.6900	-0.5463	-0.4621	-0.0842	-5.2348	-5.0050
49.6900	-0.4111	-0.4001	-0.0110	-5.6459	-5.4051
61.6900	-0.3655	-0.3724	6.9000e-3	-6.0114	-5.7775
73.6900	-0.3289	-0.3538	0.0249	-6.3403	-6.1313
85.6900	-0.3029	-0.3390	0.0361	-6.6432	-6.4703
97.6900	-0.2794	-0.3259	0.0466	-6.9225	-6.7962
109.6900	-0.2603	-0.3140	0.0537	-7.1828	-7.1102
121.6900	-0.2420	-0.3027	0.0607	-7.4248	-7.4129
133.6900	-0.2236	-0.2919	0.0683	-7.6484	-7.7048
145.6900	-0.2082	-0.2815	0.0733	-7.8566	-7.9863
157.6900	-0.1955	-0.2714	0.0759	-8.0521	-8.2577
169.6900	-0.1812	-0.2616	0.0804	-8.2333	-8.5193
181.6900	-0.1709	-0.2520	0.0812	-8.4042	-8.7713
193.6900	-0.1604	-0.2427	0.0823	-8.5646	-9.0140
205.6900	-0.1623	-0.2336	0.0713	-8.7269	-9.2476
217.6900	-0.1303	-0.2247	0.0943	-8.8572	-9.4723
229.6900	-0.1338	-0.2159	0.0822	-8.9910	-9.6882
241.6900	-0.1286	-0.2074	0.0788	-9.1196	-9.8956
0.9900	-0.0594				
20.9900	-1.2058	-1.3664	0.1606	-1.2652	-1.4258
40.9900	-1.1393	-1.0849	-0.0545	-2.4046	-2.5106
60.9900	-0.6344	-0.7078	0.0734	-3.0390	-3.2184
80.9900	-0.4555	-0.3484	-0.1071	-3.4945	-3.5669
100.9900	-0.3057	-0.1405	-0.1653	-3.8002	-3.7073
120.9900	-0.2034	-0.0631	-0.1402	-4.0036	-3.7705
140.9900	-0.1482	-0.0379	-0.1103	-4.1518	-3.8084
160.9900	-0.1157	-0.0282	-0.0875	-4.2675	-3.8366
180.9900	-0.1062	-0.0236	-0.0826	-4.3737	-3.8602
200.9900	-0.0992	-0.0208	-0.0784	-4.4729	-3.8810
220.9900	-0.0890	-0.0189	-0.0701	-4.5619	-3.8999
240.9900	-0.0862	-0.0174	-0.0688	-4.6481	-3.9173

Titration Plots

Experimental (symbols) and calculated (cross and lines) heats

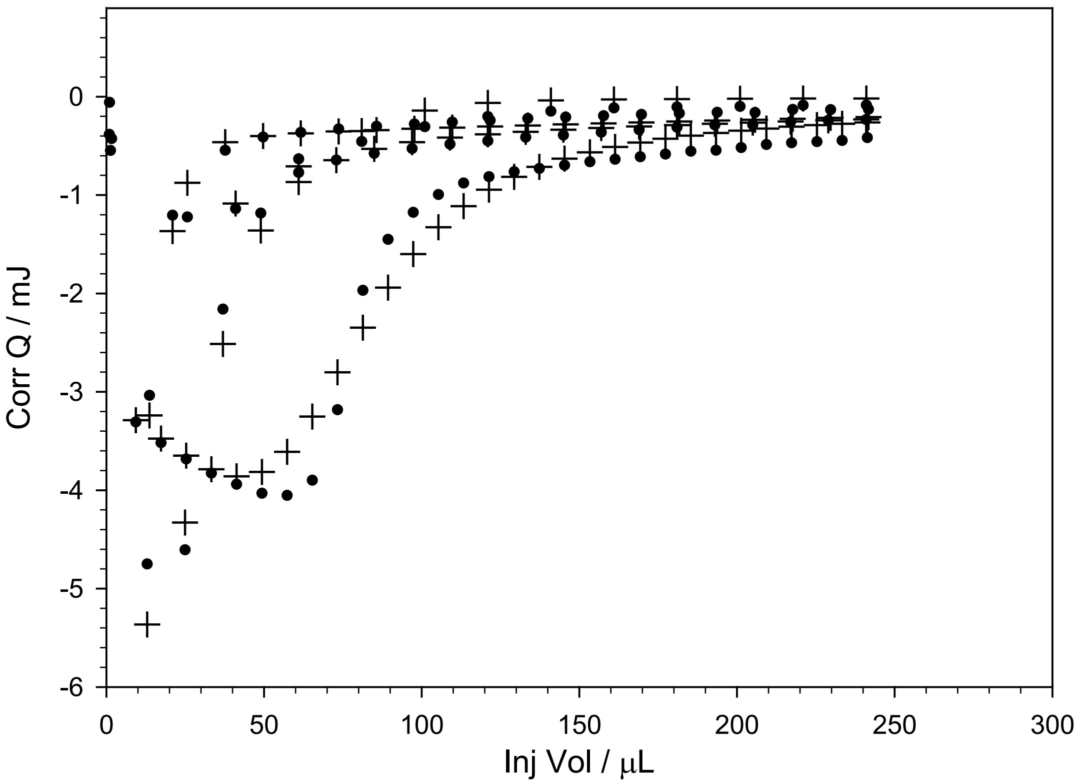

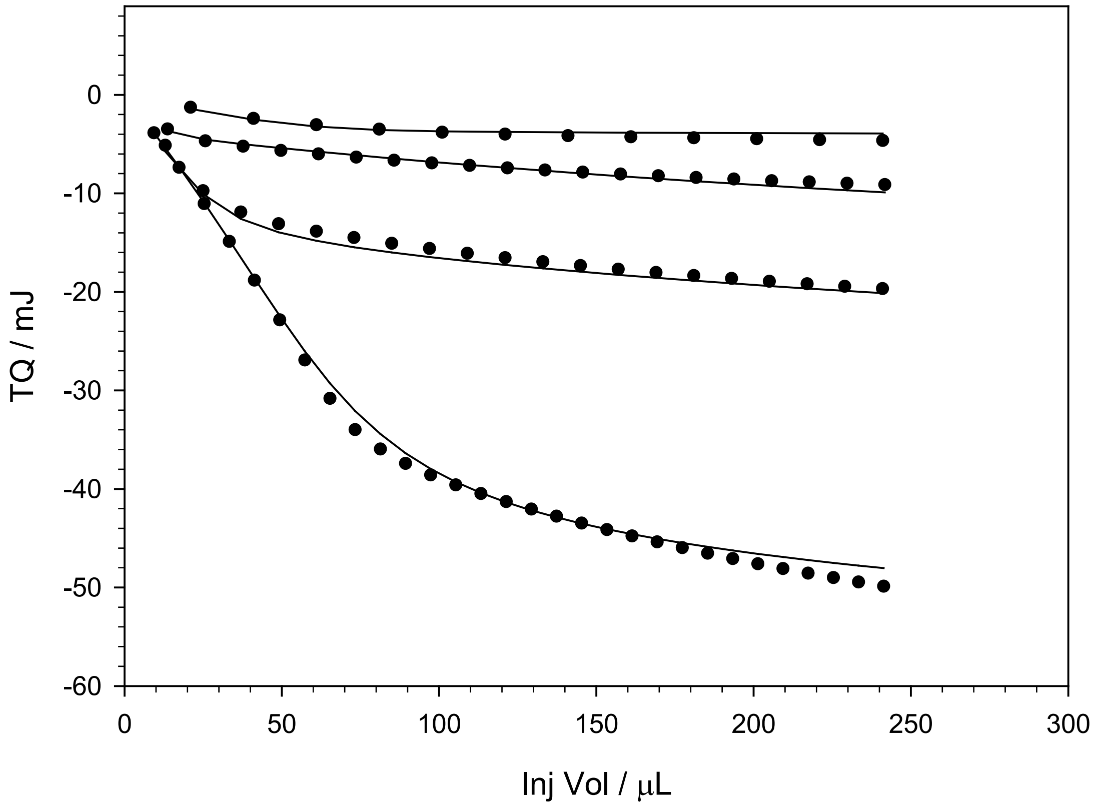

2 + Theophylline (H_2O, 298 K).

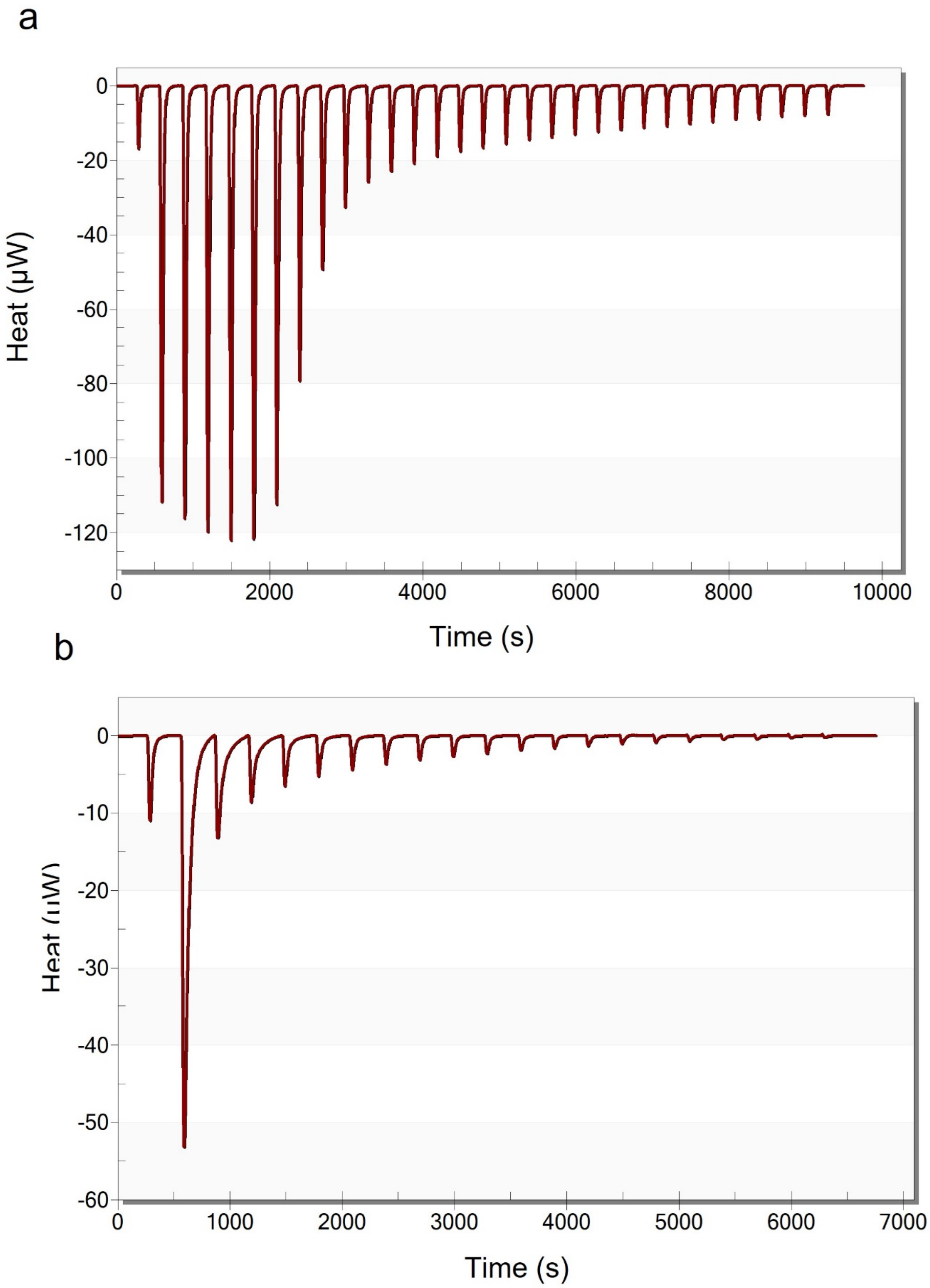

Figure S60. ITC results of Theophylline with **2** in H_2O at 298 K: a) Titration of **2** ($1.05\ 10^{-3}$ mol L^{-1}) with Theophylline ($1.24\ 10^{-2}$ mol L^{-1}); b) Titration of **2** ($2.63\ 10^{-4}$ mol L^{-1}) with Theophylline ($1.24\ 10^{-2}$ mol L^{-1}).

Data Table

R = **2** G = Theophylline

[G] = 1.24 10^{-2} mol L^{-1}

Titration 1: [R] = 1.05 10^{-3} mol L^{-1}

Injection	Q (μJ)	Corrected Q (μJ)	inj volume (μL)	mol G (mol)	mol R (mol)	mol G / mol R	total volume (μL)
1	-602.22	-633.839	3	3.72E-08	9.89E-07	0.03761	945
2	-3992.65	-4192.08	8	1.36E-07	9.81E-07	0.138759	945
3	-4239.09	-4434.52	8	2.34E-07	9.72E-07	0.240773	945
4	-4461.45	-4655.69	8	3.31E-07	9.64E-07	0.343657	945
5	-4627.65	-4821.1	8	4.28E-07	9.56E-07	0.447419	945
6	-4668.6	-4851.76	8	5.23E-07	9.48E-07	0.552067	945
7	-4288.45	-4467.86	8	6.18E-07	9.40E-07	0.657609	945
8	-3052.14	-3227.22	8	7.12E-07	9.32E-07	0.764052	945
9	-1909.47	-2082.28	8	8.05E-07	9.24E-07	0.871404	945
10	-1250.22	-1420.09	8	8.98E-07	9.16E-07	0.979672	945
11	-985.136	-1146.95	8	9.89E-07	9.08E-07	1.088865	945
12	-847.512	-1007.54	8	1.08E-06	9.01E-07	1.19899	945
13	-753.584	-909.747	8	1.17E-06	8.93E-07	1.310055	945
14	-686.868	-838.835	8	1.26E-06	8.86E-07	1.422069	945
15	-634.489	-783.794	8	1.35E-06	8.78E-07	1.535039	945
16	-584.488	-731.628	8	1.44E-06	8.71E-07	1.648973	945
17	-546.806	-689.937	8	1.52E-06	8.63E-07	1.76388	945
18	-516.319	-669.817	8	1.61E-06	8.56E-07	1.879768	945
19	-484.143	-621.433	8	1.69E-06	8.49E-07	1.996646	945
20	-457.617	-593.965	8	1.78E-06	8.42E-07	2.114521	945
21	-436.107	-567.29	8	1.86E-06	8.34E-07	2.233403	945
22	-413.543	-541.031	8	1.95E-06	8.27E-07	2.3533	945
23	-389.25	-515.032	8	2.03E-06	8.20E-07	2.474221	945
24	-370.975	-496.062	8	2.11E-06	8.13E-07	2.596174	945
25	-351.626	-472.364	8	2.19E-06	8.07E-07	2.719168	945
26	-332.959	-451.805	8	2.27E-06	8.00E-07	2.843212	945
27	-321.802	-437.646	8	2.35E-06	7.93E-07	2.968316	945
28	-304.219	-417.532	8	2.43E-06	7.86E-07	3.094487	945
29	-275.387	-384.204	8	2.51E-06	7.80E-07	3.221736	945
30	-280.973	-388.59	8	2.59E-06	7.73E-07	3.350071	945
31	-265.455	-373.95	8	2.67E-06	7.66E-07	3.479502	945

Titration 2: [R] = 2.63 10^{-4} mol L^{-1}

Injection	Q (µJ)	Corrected Q (µJ)	inj volume (µL)	mol G (mol)	mol R (mol)	mol G / mol R	total volume (µL)
1	-408.778	-438.103	3	3.72E-08	2.48E-07	0.150154	945
2	-3456.83	-3745.87	12	1.86E-07	2.45E-07	0.758494	945
3	-935.779	-1218.78	12	3.32E-07	2.41E-07	1.374658	945
4	-551.761	-827.409	12	4.77E-07	2.38E-07	1.998748	945
5	-316.158	-585.447	12	6.19E-07	2.35E-07	2.630864	945
6	-220.941	-481.006	12	7.60E-07	2.32E-07	3.27111	945
7	-178.711	-432.811	12	8.99E-07	2.29E-07	3.919591	945
8	-143.241	-385.72	12	1.04E-06	2.27E-07	4.576413	945
9	-120.952	-356.805	12	1.17E-06	2.24E-07	5.241682	945
10	-100.828	-330.157	12	1.31E-06	2.21E-07	5.915508	945
11	-86.4903	-309.372	12	1.44E-06	2.18E-07	6.598001	945
12	-71.7443	-286.389	12	1.57E-06	2.15E-07	7.289271	945
13	-58.2822	-267.988	12	1.70E-06	2.13E-07	7.989433	945
14	-51.2967	-250.948	12	1.83E-06	2.10E-07	8.6986	945
15	-30.1334	-225.916	12	1.95E-06	2.07E-07	9.416888	945
16	-31.4265	-216.835	12	2.07E-06	2.05E-07	10.14441	945
17	-26.0512	-217.989	12	2.20E-06	2.02E-07	10.8813	945
18	-18.2848	-195.245	12	2.32E-06	1.99E-07	11.62766	945
19	-15.7311	-187.847	12	2.44E-06	1.97E-07	12.38362	945
20	-9.29303	-174.926	12	2.56E-06	1.94E-07	13.1493	945
21	-4.1722	-159.993	12	2.67E-06	1.92E-07	13.92484	945

Results page

```
Reagent Reagent
  number   name
    1      R
    2      G

Chi-squared =  39.60

   sigma =   0.19445

 Formation constants   Value    relative    log    standard
                                std devn    beta   deviation
 Beta A constant    0.4732E+05             4.6750    0.1259    2  0
 Beta B constant    0.1148E+13            12.0599    0.2659    4  0
 Beta C constant    0.6400E+01             0.8062    0.0177    0  2
 Beta D refined     0.9155E+05  0.5335     4.9616    0.2317    1  1
 Beta E refined     0.2835E+10  0.5795     9.4526    0.2517    2  1

 Formation entalpies    Value   standard
                                deviation
-DeltaH A refined   -1022.0275 155.1872
-DeltaH B refined   -1552.9250 227.2779
-DeltaH C refined     -43.6231   9.2758
-DeltaH D refined    -388.0024  63.0849
-DeltaH E refined    -856.5359 134.4032

++++++++++++++++++++++++++++++++++++++++++++++++++++++++++++++++++++++++++++++++
 Thermodynamic Functions, kJ/mol
             - DeltaG°                - DeltaH°                 T DeltaS°
   A       26.6850    1.3225     -1022.0275  155.1872       1048.7124  155.1929
   B       68.8381    1.4365     -1552.9250  227.2779       1621.7631  227.2824
   C        4.6018    0.3764       -43.6231    9.2758         48.2249    9.2834
   D       28.3212    0.3628      -388.0024   63.0849        416.3235   63.3911
   E       53.9556    0.5271      -856.5359  134.4032        910.4914  134.8442
++++++++++++++++++++++++++++++++++++++++++++++++++++++++++++++++++++++++++++++++

 Correlation coefficients*1000         Run timed at 11.47 on 22 Jan 2019
   2  956
   3 -859 -835
   4 -853 -844  992
   5  139  -42  -20  -12
   6 -843 -835  998  994   27
   7 -856 -836  999  996  -18  998
        1    2    3    4    5    6

 Order of parameters:
   1       Beta   D
   2       Beta   E
   3      -DeltaH A
   4      -DeltaH B
   5      -DeltaH C
   6      -DeltaH D
   7      -DeltaH E
```

Results table

Addition (µL)	Qobs (mJ)	Qcalc (mJ)	residual (mJ)	QTobs (mJ)	QTcalc (mJ)
1.2100	-0.6338				
9.2100	-4.1921	-4.1360	-0.0561	-4.8259	-4.7698
17.2100	-4.4345	-4.5048	0.0703	-9.2604	-9.2746
25.2100	-4.6557	-4.7812	0.1255	-13.9161	-14.0558
33.2100	-4.8211	-4.8449	0.0238	-18.7372	-18.9007
41.2100	-4.8518	-4.5373	-0.3144	-23.5890	-23.4380
49.2100	-4.4679	-3.8181	-0.6498	-28.0568	-27.2561
57.2100	-3.2272	-2.9512	-0.2760	-31.2841	-30.2073
65.2100	-2.0823	-2.2566	0.1743	-33.3663	-32.4638
73.2100	-1.4201	-1.7949	0.3749	-34.7864	-34.2588
81.2100	-1.1470	-1.4905	0.3435	-35.9334	-35.7493
89.2100	-1.0075	-1.2768	0.2693	-36.9409	-37.0261
97.2100	-0.9097	-1.1172	0.2074	-37.8507	-38.1433
105.2100	-0.8388	-0.9923	0.1534	-38.6895	-39.1355
113.2100	-0.7838	-0.8914	0.1076	-39.4733	-40.0269
121.2100	-0.7316	-0.8079	0.0763	-40.2049	-40.8348
129.2100	-0.6899	-0.7377	0.0478	-40.8949	-41.5726
137.2100	-0.6698	-0.6779	8.0000e-3	-41.5647	-42.2504
145.2100	-0.6214	-0.6262	4.8000e-3	-42.1861	-42.8766
153.2100	-0.5940	-0.5812	-0.0128	-42.7801	-43.4579
161.2100	-0.5673	-0.5417	-0.0256	-43.3474	-43.9995
169.2100	-0.5410	-0.5067	-0.0344	-43.8884	-44.5062
177.2100	-0.5150	-0.4754	-0.0396	-44.4034	-44.9816
185.2100	-0.4961	-0.4474	-0.0486	-44.8995	-45.4290
193.2100	-0.4724	-0.4221	-0.0502	-45.3719	-45.8511
201.2100	-0.4518	-0.3992	-0.0526	-45.8237	-46.2503
209.2100	-0.4376	-0.3783	-0.0594	-46.2613	-46.6286
217.2100	-0.4175	-0.3591	-0.0584	-46.6788	-46.9877
225.2100	-0.3842	-0.3415	-0.0427	-47.0631	-47.3292
233.2100	-0.3886	-0.3252	-0.0634	-47.4516	-47.6545
241.2100	-0.3739	-0.3101	-0.0638	-47.8256	-47.9646
1.4000	-0.4381				
13.4000	-3.7459	-4.2420	0.4961	-4.1840	-4.6801
25.4000	-1.2188	-0.8558	-0.3630	-5.4027	-5.5359
37.4000	-0.8274	-0.4877	-0.3397	-6.2302	-6.0236
49.4000	-0.5854	-0.4585	-0.1269	-6.8156	-6.4821
61.4000	-0.4810	-0.4292	-0.0518	-7.2966	-6.9112
73.4000	-0.4328	-0.4019	-0.0310	-7.7294	-7.3131
85.4000	-0.3857	-0.3782	-7.5000e-3	-8.1151	-7.6913
97.4000	-0.3568	-0.3578	1.0000e-3	-8.4719	-8.0491
109.4000	-0.3302	-0.3398	9.7000e-3	-8.8021	-8.3890
121.4000	-0.3094	-0.3238	0.0144	-9.1115	-8.7127
133.4000	-0.2864	-0.3091	0.0227	-9.3979	-9.0218
145.4000	-0.2680	-0.2956	0.0276	-9.6659	-9.3174
157.4000	-0.2509	-0.2829	0.0319	-9.9168	-9.6003
169.4000	-0.2259	-0.2709	0.0450	-10.1427	-9.8712
181.4000	-0.2168	-0.2595	0.0427	-10.3596	-10.1307
193.4000	-0.2180	-0.2487	0.0307	-10.5775	-10.3794
205.4000	-0.1952	-0.2382	0.0430	-10.7728	-10.6177
217.4000	-0.1878	-0.2282	0.0403	-10.9606	-10.8459
229.4000	-0.1749	-0.2185	0.0436	-11.1356	-11.0643
241.4000	-0.1600	-0.2091	0.0491	-11.2956	-11.2734

Titration Plots

Experimental (symbols) and calculated (cross and lines) heats

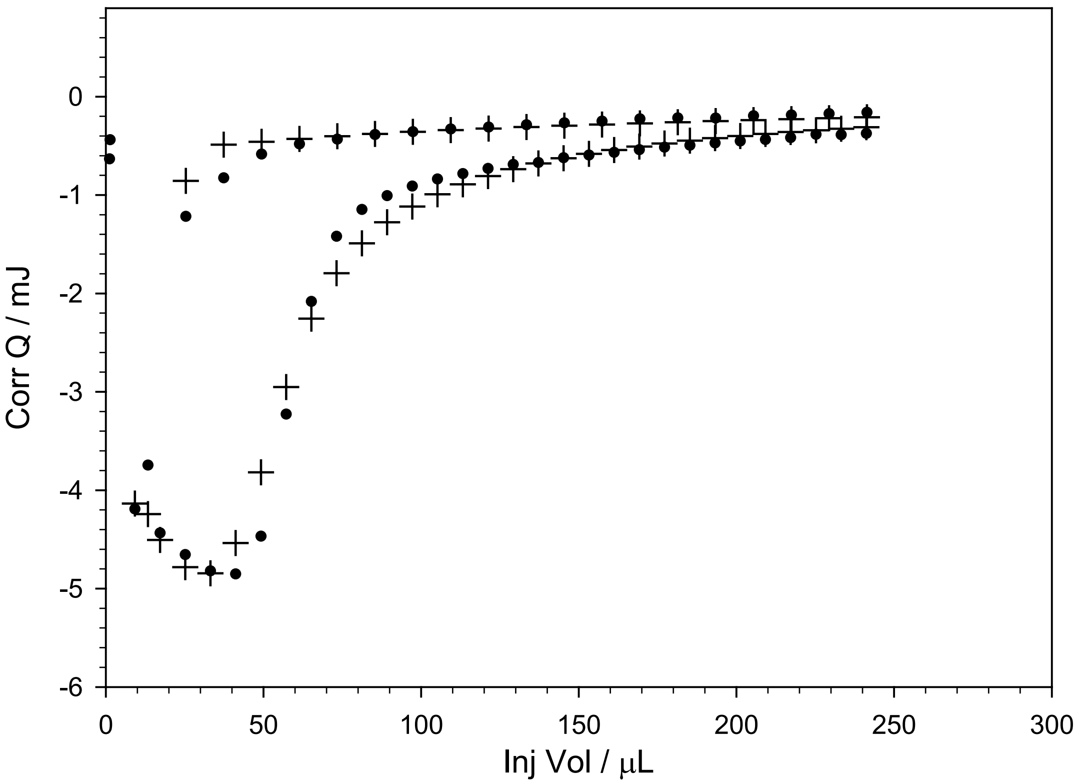

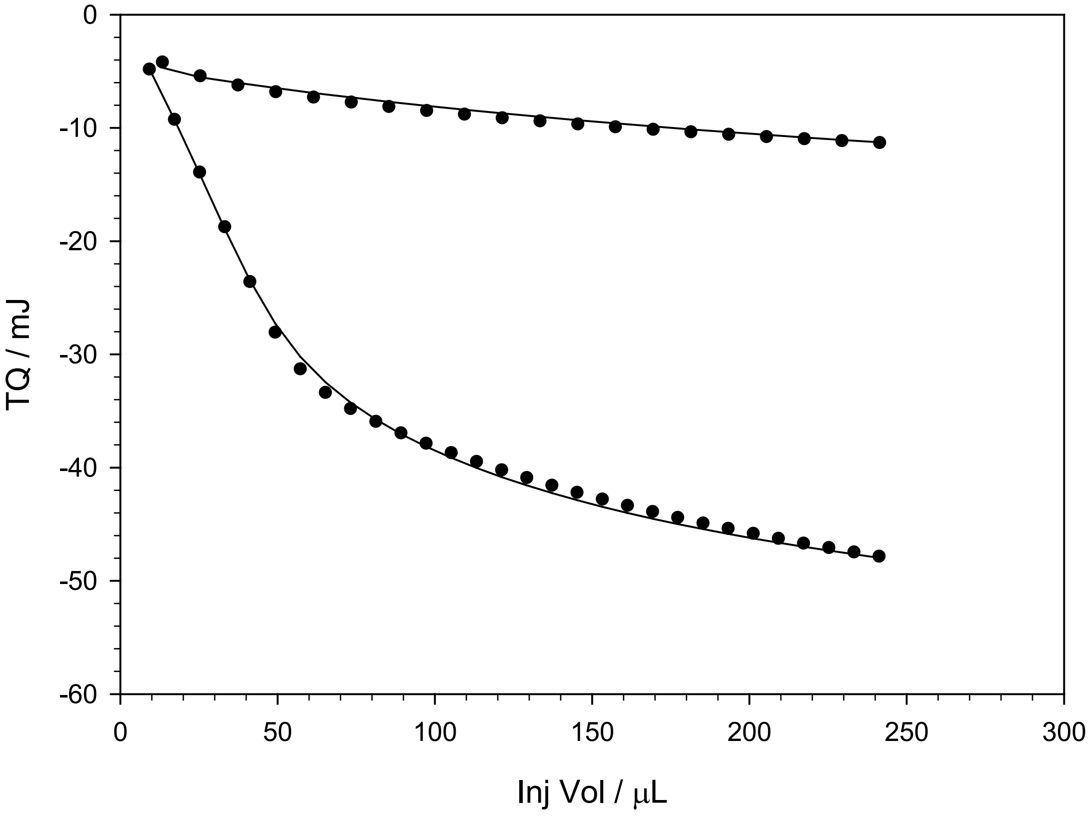

2 + Theobromine (H_2O, 298 K).

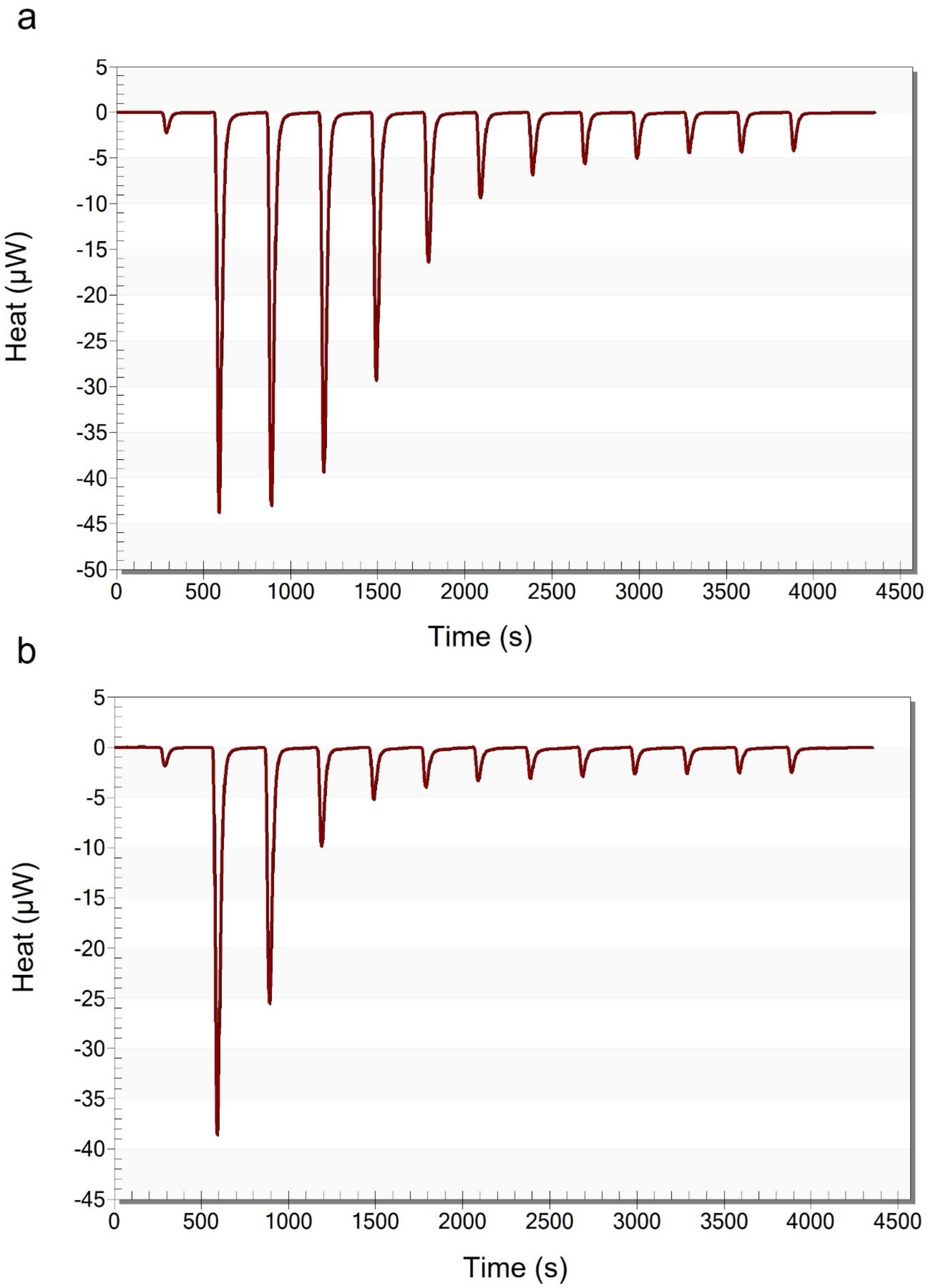

Figure S61. ITC results of Theobromine with **2** in H_2O at 298 K: a) Titration of **2** ($2.46\ 10^{-4}$ mol L^{-1}) with Theobromine ($2.00\ 10^{-4}$ mol L^{-1}); b) Titration of **2** ($6.15\ 10^{-5}$ mol L^{-1}) with Theobromine ($2.00\ 10^{-4}$ mol L^{-1}).

Data Table

R = **2**   G = Theobromine

[G] = 2.00 10^{-3} mol L^{-1}

Titration 1: [R] = 2.46 10^{-4} mol L^{-1}

Injection	Q (μJ)	Corrected Q (μJ)	inj volume (μL)	mol G (mol)	mol R (mol)	mol G / mol R	total volume (μL)
1	-79.1691	-76.7117	3	6.00E-09	2.32E-07	0.025892	945
2	-1460.28	-1383.55	20	4.59E-08	2.27E-07	0.202237	945
3	-1446.83	-1381.77	20	8.49E-08	2.22E-07	0.382396	945
4	-1369	-1317.6	20	1.23E-07	2.17E-07	0.566449	945
5	-1058.31	-1013.94	20	1.60E-07	2.13E-07	0.754482	945
6	-598.807	-557.302	20	1.97E-07	2.08E-07	0.946581	945
7	-342.865	-301.79	20	2.33E-07	2.04E-07	1.142833	945
8	-246.735	-202.269	20	2.68E-07	2.00E-07	1.343329	945
9	-198.077	-155.736	20	3.02E-07	1.95E-07	1.548159	945
10	-179.246	-136.22	20	3.36E-07	1.91E-07	1.757418	945
11	-151.83	-111.403	20	3.69E-07	1.87E-07	1.971202	945
12	-149.77	-114.668	20	4.01E-07	1.83E-07	2.189608	945
13	-154.703	-101.898	20	4.33E-07	1.79E-07	2.412737	945

Titration 2: [R] = 6.15 10^{-5} mol L^{-1}

Injection	Q (μJ)	Corrected Q (μJ)	inj volume (μL)	mol G (mol)	mol R (mol)	mol G / mol R	total volume (μL)
1	-70.8874	-68.43	3	6.00E-09	5.79E-08	0.103568	945
2	-934.623	-857.902	20	4.59E-08	5.67E-08	0.808949	945
3	-242.514	-177.45	20	8.49E-08	5.55E-08	1.529582	945
4	-137.706	-86.3118	20	1.23E-07	5.43E-08	2.265797	945
5	-119.307	-74.9451	20	1.60E-07	5.32E-08	3.017929	945
6	-112.521	-71.0172	20	1.97E-07	5.21E-08	3.786324	945
7	-103.688	-62.6138	20	2.33E-07	5.10E-08	4.571332	945
8	-98.5599	-54.0941	20	2.68E-07	4.99E-08	5.373314	945
9	-87.6013	-45.2603	20	3.02E-07	4.88E-08	6.192636	945
10	-87.049	-44.0227	20	3.36E-07	4.78E-08	7.029673	945
11	-73.153	-32.726	20	3.69E-07	4.68E-08	7.884808	945
12	-79.29	-44.188	20	4.01E-07	4.58E-08	8.758433	945
13	-97.5577	-44.7531	20	4.33E-07	4.48E-08	9.650946	945

Results page

```
Reagent Reagent
  number   name
    1      R
    2      G

   sigma =   0.06469

 Formation constants   Value    relative    log    standard
                                std devn    beta   deviation
 Beta A constant    0.4732E+05             4.6750    0.1259    2  0
 Beta B constant    0.1148E+13            12.0599    0.2659    4  0
 Beta C constant    0.5598E+01             0.7480    0.3268    0  2
 Beta D refined     0.1808E+10  0.1395     9.2571    0.0606    2  1


 Formation entalpies    Value   standard
                                deviation
-DeltaH A refined    -610.1037 127.7590
-DeltaH B refined     809.9378 105.1963
-DeltaH C refined    -169.8782  94.2826
-DeltaH D refined    -379.4551 101.0831

++++++++++++++++++++++++++++++++++++++++++++++++++++++++++++++++++++++++++++++++
 Thermodynamic Functions, kJ/mol

             - DeltaG°                 - DeltaH°                  T DeltaS°
   A        26.6850    0.3457      -610.1037  127.7590        636.7887  127.7594
   B        68.8381    0.5191       809.9378  105.1963       -741.0997  105.1976
   C         4.2696    0.3220      -169.8782   94.2826        174.1478   94.2832
   D        52.8398    1.3758      -379.4551  101.0831        432.2949  101.3096

++++++++++++++++++++++++++++++++++++++++++++++++++++++++++++++++++++++++++++++++

 Correlation coefficients*1000         Run timed at 10.21 on 24 Jan 2019

   2 -194
   3  -22 -128
   4  406  555 -466
   5 -158  997 -184  599
       1    2    3    4

 Order of parameters:
   1       Beta   D
   2      -DeltaH A
   3      -DeltaH B
   4      -DeltaH C
   5      -DeltaH D
```

Results table

Addition (μL)	Qobs (mJ)	Qcalc (mJ)	residual (mJ)	QTobs (mJ)	QTcalc (mJ)
1.1100	-0.0767				
21.1100	-1.3836	-1.3261	-0.0575	-1.4603	-1.4028
41.1100	-1.3818	-1.5368	0.1550	-2.8420	-2.9396
61.1100	-1.3176	-1.2684	-0.0492	-4.1596	-4.2079
81.1100	-1.0139	-0.8682	-0.1457	-5.1736	-5.0762
101.1100	-0.5573	-0.5638	6.5000e-3	-5.7309	-5.6400
121.1100	-0.3018	-0.3740	0.0722	-6.0327	-6.0140
141.1100	-0.2023	-0.2597	0.0574	-6.2349	-6.2737
161.1100	-0.1557	-0.1891	0.0333	-6.3907	-6.4628
181.1100	-0.1362	-0.1436	7.4000e-3	-6.5269	-6.6064
201.1100	-0.1114	-0.1130	1.6000e-3	-6.6383	-6.7193
221.1100	-0.1147	-0.0915	-0.0232	-6.7530	-6.8109
241.1100	-0.1019	-0.0759	-0.0260	-6.8549	-6.8867
1.6000	-0.0684				
21.6000	-0.8579	-0.7956	-0.0623	-0.9263	-0.8640
41.6000	-0.1775	-0.2774	0.0999	-1.1038	-1.1414
61.6000	-0.0863	-0.1233	0.0370	-1.1901	-1.2647
81.6000	-0.0749	-0.0714	-3.5000e-3	-1.2650	-1.3361
101.6000	-0.0710	-0.0506	-0.0204	-1.3361	-1.3867
121.6000	-0.0626	-0.0408	-0.0218	-1.3987	-1.4275
141.6000	-0.0541	-0.0356	-0.0185	-1.4528	-1.4631
161.6000	-0.0453	-0.0323	-0.0129	-1.4980	-1.4954
181.6000	-0.0440	-0.0300	-0.0140	-1.5420	-1.5254
201.6000	-0.0327	-0.0281	-4.6000e-3	-1.5748	-1.5535
221.6000	-0.0442	-0.0264	-0.0177	-1.6190	-1.5800
241.6000	-0.0448	-0.0249	-0.0199	-1.6637	-1.6049

Titration Plots

Experimental (symbols) and calculated (cross and lines) heats

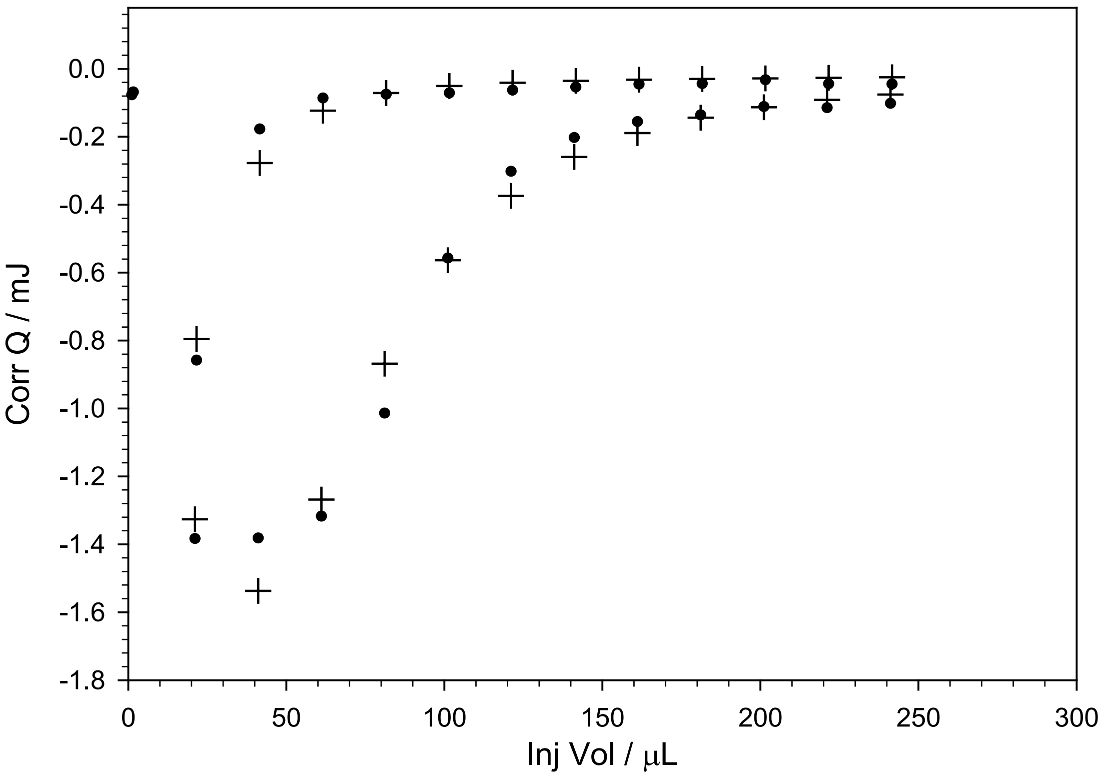

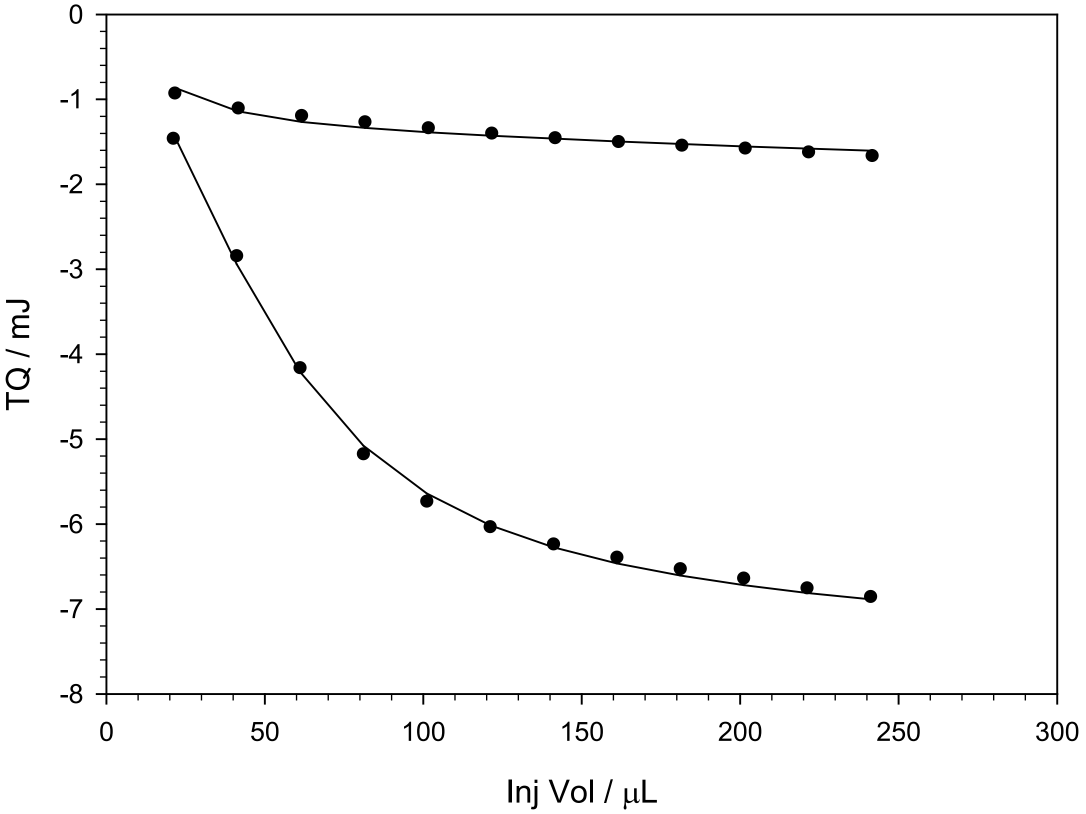

6 + Caffeine (H_2O, 298 K).

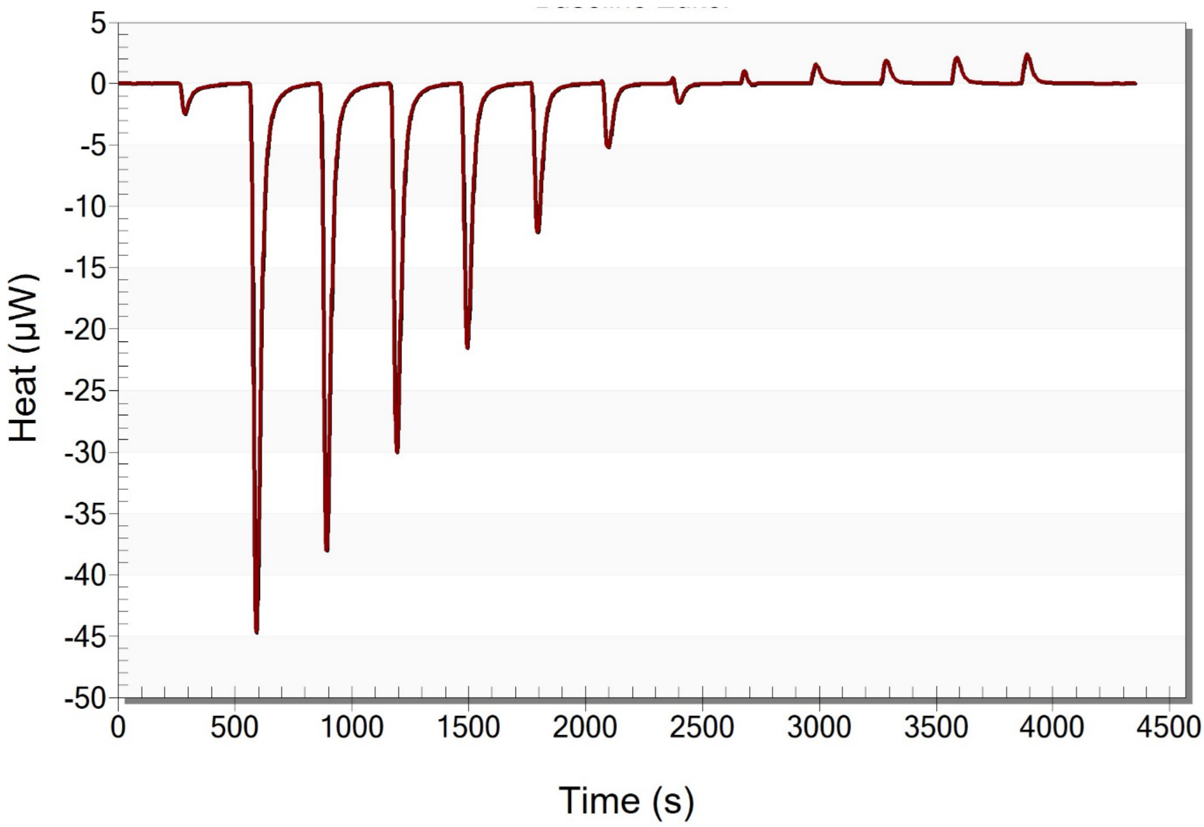

Figure S62. ITC results the titration of Caffeine (1.96 10^{-4} mol L^{-1}) with receptor **6** (2.13 10^{-3} mol L^{-1}) in H_2O at 298 K.

Data Table

R = **6** G = Caffeine

[R] = 2.13 10^{-3} mol L^{-1}

Titration 1: [G] = 1.96 10^{-4} mol L^{-1}

Injection	Q (μJ)	Corrected Q (μJ)	inj volume (μL)	mol G (mol)	mol R (mol)	mol G / mol R	total volume (μL)
1	-123.703	-125.151	3	6.39E-09	1.85E-07	0.034609	945
2	-1816.07	-1926.76	20	4.89E-08	1.81E-07	0.270327	945
3	-1549.02	-1686.37	20	9.04E-08	1.77E-07	0.511142	945
4	-1259.97	-1412.07	20	1.31E-07	1.73E-07	0.757163	945
5	-910.202	-1062.21	20	1.71E-07	1.69E-07	1.008504	945
6	-522.458	-678.661	20	2.10E-07	1.66E-07	1.265279	945
7	-220.608	-369.315	20	2.48E-07	1.62E-07	1.527606	945
8	-58.2722	-206.707	20	2.85E-07	1.59E-07	1.795605	945
9	22.03275	-123.802	20	3.22E-07	1.56E-07	2.069399	945
10	67.447	-77.6197	20	3.58E-07	1.52E-07	2.349112	945
11	80.14672	-61.1229	20	3.93E-07	1.49E-07	2.634874	945
12	89.57153	-39.5844	20	4.27E-07	1.46E-07	2.926813	945
13	100.0929	-35.6177	20	4.61E-07	1.43E-07	3.225066	945

Results page

```
Reagent Reagent
  number   name
    1      G
    2      R

 Temperature: 25.000 Celsius. Excessive limit 0.99

Refinement successful

   sigma =   0.04261

 Formation constants   Value    relative    log    standard
                                std devn    beta   deviation
 Beta A refined     0.6647E+05  0.1341    4.8227    0.0582    1  1


 Formation entalpies    Value   standard
                                deviation
-DeltaH A refined      47.2568   0.9654


++++++++++++++++++++++++++++++++++++++++++++++++++++++++++++++++++++++++++++++++

 Thermodynamic Functions, kJ/mol


              - DeltaG°                - DeltaH°                 T DeltaS°
   A        27.5278    0.3323        47.2568    0.9654       -19.7290    1.2369



++++++++++++++++++++++++++++++++++++++++++++++++++++++++++++++++++++++++++++++++


 Correlation coefficients*1000         Run timed at 16.08 on 22 Sep 2020

   2 -760
       1

 Order of parameters:
   1       Beta   A
   2      -DeltaH A
```

Results table

Addition (μL)	Qobs (mJ)	Qcalc (mJ)	residual (mJ)	QTobs (mJ)	QTcalc (mJ)
1.3000	-0.1252				
21.3000	-1.9268	-1.8251	-0.1017	-2.0519	-1.9503
41.3000	-1.6864	-1.7031	0.0168	-3.7383	-3.6534
61.3000	-1.4121	-1.4728	0.0608	-5.1503	-5.1262
81.3000	-1.0622	-1.0990	0.0368	-6.2126	-6.2253
101.3000	-0.6787	-0.6859	7.2000e-3	-6.8912	-6.9112
121.3000	-0.3693	-0.3929	0.0235	-7.2605	-7.3040
141.3000	-0.2067	-0.2316	0.0249	-7.4672	-7.5356
161.3000	-0.1238	-0.1464	0.0226	-7.5910	-7.6820
181.3000	-0.0776	-0.0990	0.0214	-7.6687	-7.7810
201.3000	-0.0611	-0.0708	9.6000e-3	-7.7298	-7.8518
221.3000	-0.0396	-0.0528	0.0132	-7.7694	-7.9046
241.3000	-0.0356	-0.0408	5.2000e-3	-7.8050	-7.9455

Titration Plots

Experimental (symbols) and calculated (cross and lines) heats

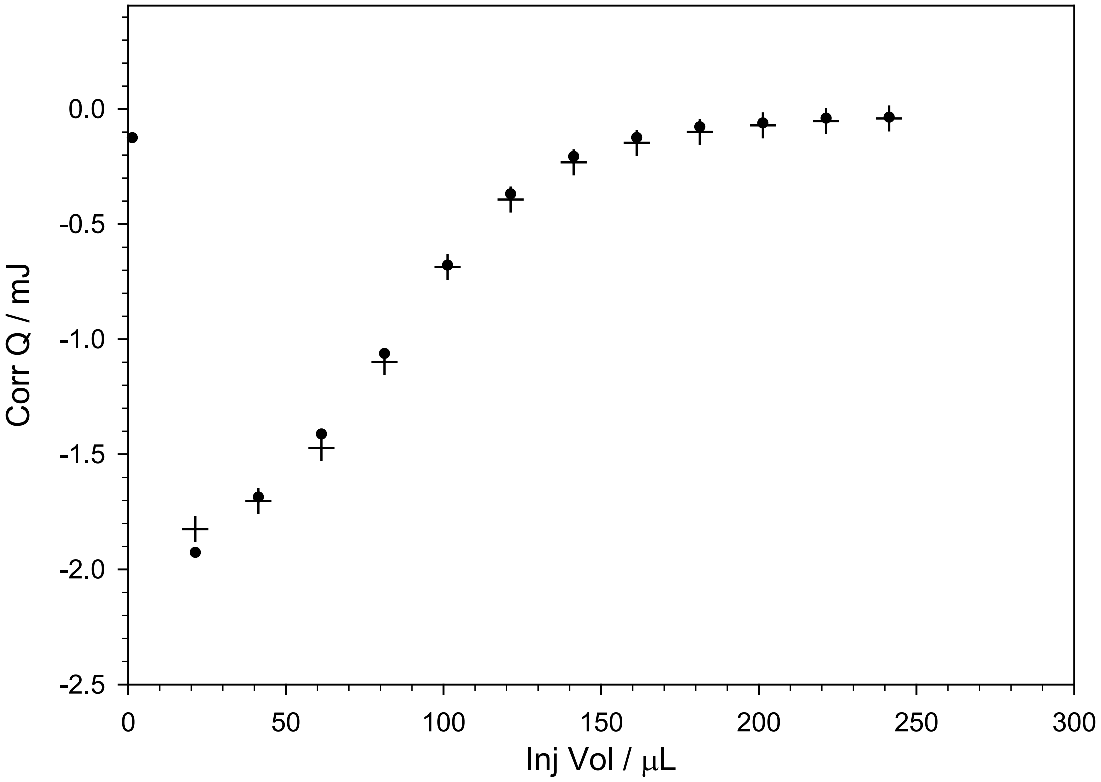

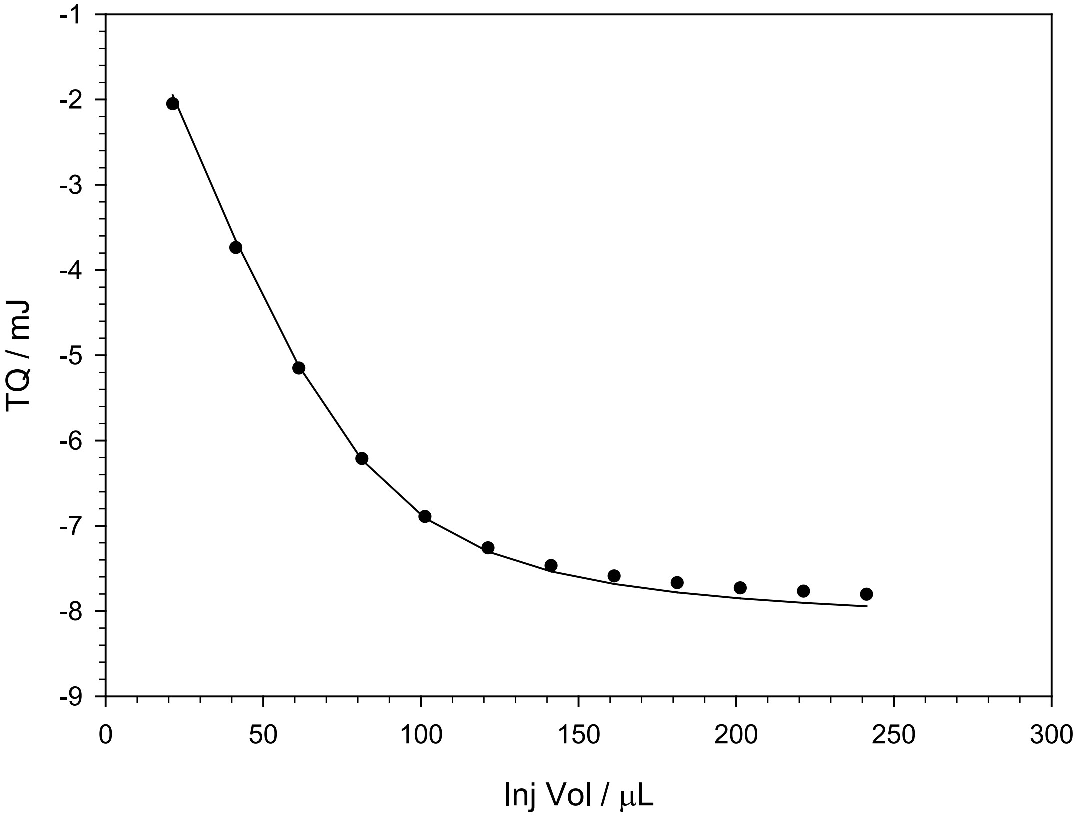

6 + Theophylline (H_2O, 298 K).

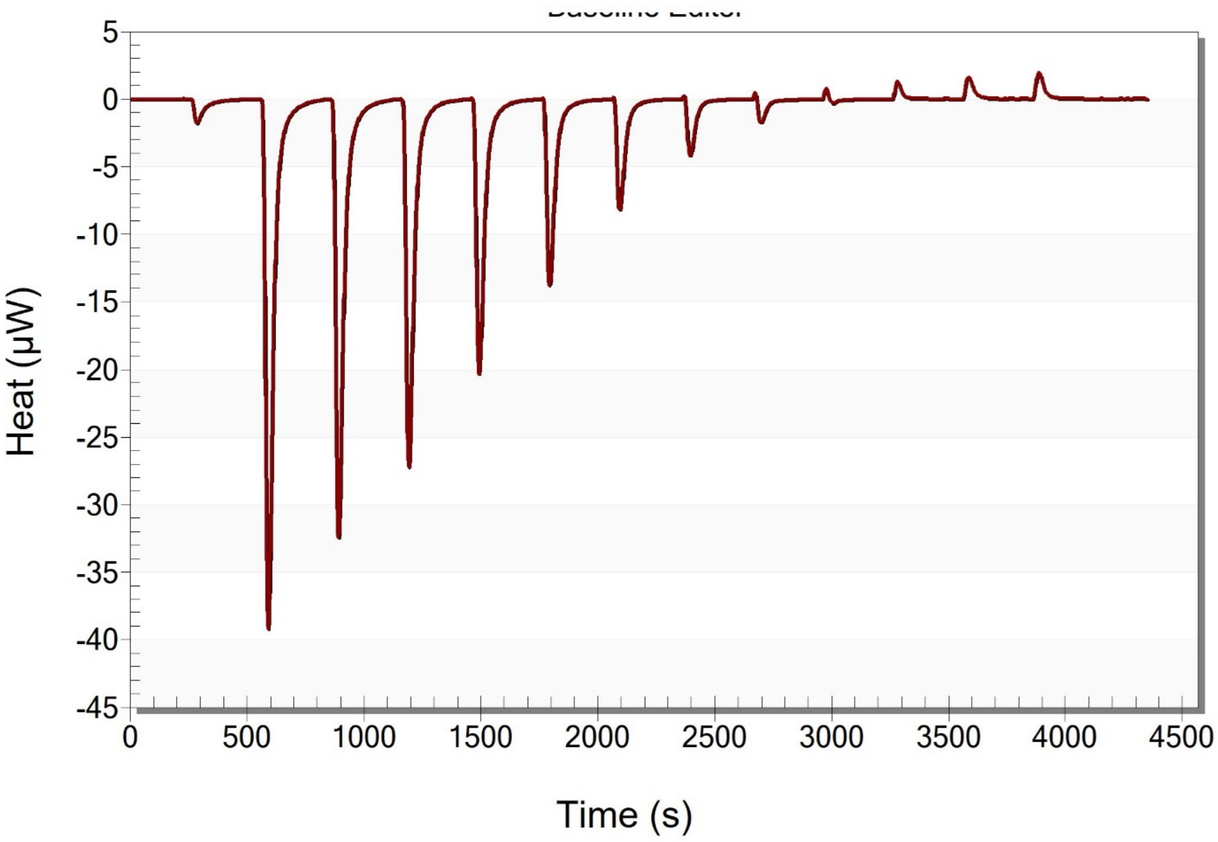

Figure S63. ITC results the titration of Theophylline (2.11 10^{-4} mol L^{-1}) with receptor **6** (2.13 10^{-3} mol L^{-1}) in H_2O at 298 K.

Data Table

R = **6** G = Theophylline

[R] = 2.13 10^{-3} mol L^{-1}

Titration 1: [G] = 2.11 10^{-4} mol L^{-1}

Injection	Q (μJ)	Corrected Q (μJ)	inj volume (μL)	mol G (mol)	mol R (mol)	mol G / mol R	total volume (μL)
1	-94.3033	-95.7506	3	6.39E-09	1.99E-07	0.032149	945
2	-1599.6	-1710.29	20	4.89E-08	1.95E-07	0.25111	945
3	-1377.88	-1515.22	20	9.04E-08	1.90E-07	0.474805	945
4	-1145.16	-1297.25	20	1.31E-07	1.86E-07	0.703337	945
5	-880.138	-1032.15	20	1.71E-07	1.82E-07	0.936809	945
6	-593.741	-749.945	20	2.10E-07	1.79E-07	1.17533	945
7	-346.445	-495.153	20	2.48E-07	1.75E-07	1.419009	945
8	-170.515	-318.95	20	2.85E-07	1.71E-07	1.667956	945
9	-71.2667	-217.102	20	3.22E-07	1.67E-07	1.922285	945
10	-1.33285	-146.4	20	3.58E-07	1.64E-07	2.182114	945
11	42.08462	-99.185	20	3.93E-07	1.60E-07	2.44756	945
12	66.64073	-62.5152	20	4.27E-07	1.57E-07	2.718746	945
13	81.6776	-54.033	20	4.61E-07	1.54E-07	2.995796	945

Results page

```
Reagent Reagent
  number   name
    1      G
    2      R

 Temperature: 25.000 Celsius. Excessive limit 0.99

   sigma =   0.02326

 Formation constants   Value    relative    log    standard
                                std devn    beta   deviation
 Beta A refined     0.3969E+05  0.0721    4.5987    0.0313    1  1


 Formation entalpies    Value   standard
                                deviation
-DeltaH A refined      44.9070   0.5859


++++++++++++++++++++++++++++++++++++++++++++++++++++++++++++++++++++++++++++++++

 Thermodynamic Functions, kJ/mol


              - DeltaG°                - DeltaH°                 T DeltaS°
   A        26.2495    0.1787        44.9070    0.5859       -18.6576    0.7372


+++++++++++++++++++++++++++++++++++++++++++++++++++++++++++++++++++++++++++++++


 Correlation coefficients*1000         Run timed at 09.55 on 23 Sep 2020

   2 -803
       1

 Order of parameters:
   1       Beta   A
   2      -DeltaH A
```

Results table

Addition (μL)	Qobs (mJ)	Qcalc (mJ)	residual (mJ)	QTobs (mJ)	QTcalc (mJ)
1.1200	-0.0958				
21.1200	-1.7103	-1.6573	-0.0530	-1.8060	-1.7531
41.1200	-1.5152	-1.5298	0.0145	-3.3213	-3.2828
61.1200	-1.2973	-1.3286	0.0313	-4.6185	-4.6114
81.1200	-1.0321	-1.0496	0.0174	-5.6507	-5.6610
101.1200	-0.7499	-0.7457	-4.2000e-3	-6.4006	-6.4067
121.1200	-0.4952	-0.4950	-2.0000e-4	-6.8958	-6.9017
141.1200	-0.3189	-0.3256	6.6000e-3	-7.2147	-7.2273
161.1200	-0.2171	-0.2203	3.2000e-3	-7.4318	-7.4476
181.1200	-0.1464	-0.1552	8.8000e-3	-7.5782	-7.6029
201.1200	-0.0992	-0.1138	0.0146	-7.6774	-7.7166
221.1200	-0.0625	-0.0863	0.0238	-7.7399	-7.8030
241.1200	-0.0540	-0.0674	0.0134	-7.7940	-7.8704

Titration Plots

Experimental (symbols) and calculated (cross and lines) heats

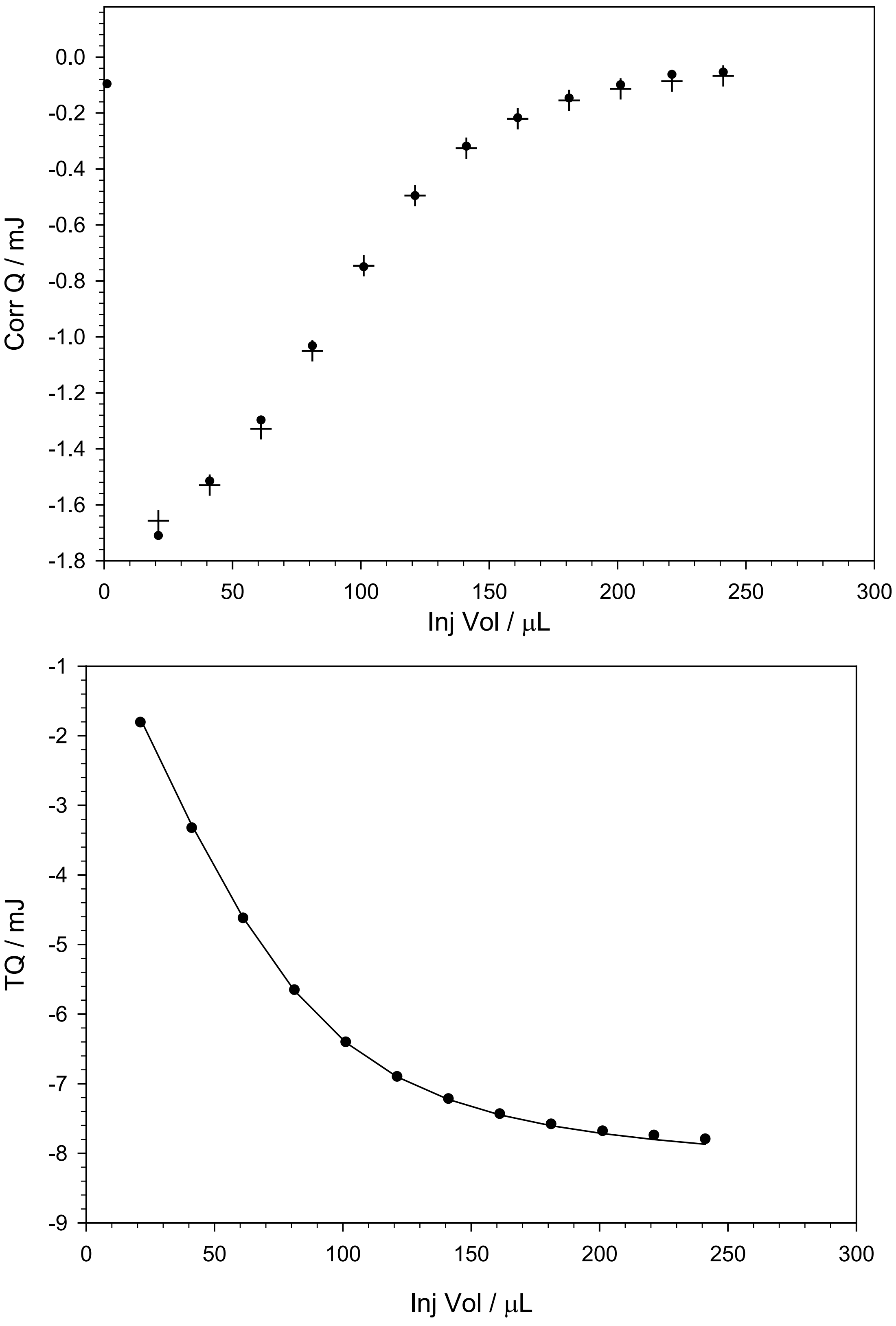

6 + Theobromine (H_2O, 298 K).

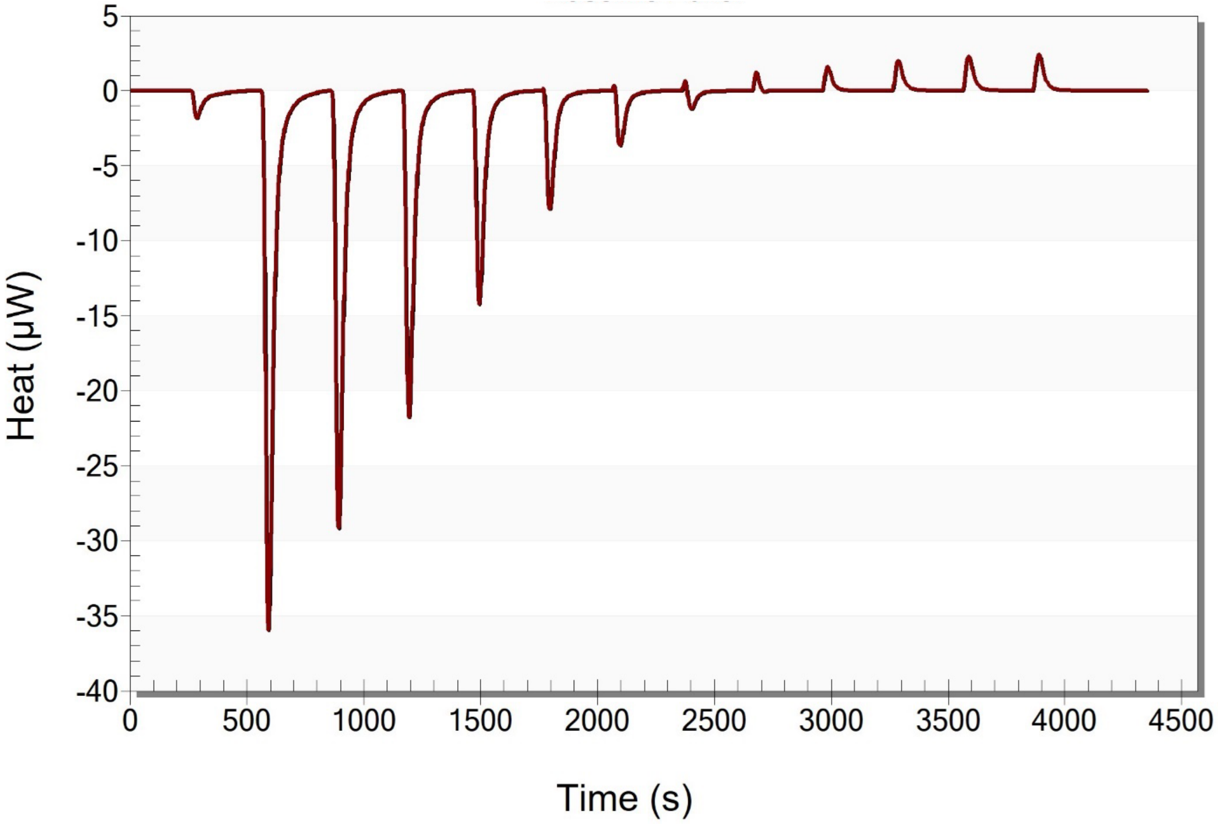

Figure S64. ITC results the titration of Theobromine ($1.78\ 10^{-4}$ mol L^{-1}) with receptor **6** ($2.13\ 10^{-3}$ mol L^{-1}) in H_2O at 298 K.

Data Table

R = **6**   G = Theobromine

[R] = $2.13\ 10^{-3}$ mol L^{-1}

Titration 1: [G] = $1.78\ 10^{-4}$ mol L^{-1}

Injection	Q (µJ)	Corrected Q (µJ)	inj volume (µL)	mol G (mol)	mol R (mol)	mol G / mol R	total volume (µL)
1	-96.5643	-98.0117	3	6.39E-09	1.68E-07	0.038109	945
2	-1526.42	-1637.11	20	4.89E-08	1.64E-07	0.297664	945
3	-1244.57	-1381.92	20	9.04E-08	1.61E-07	0.56283	945
4	-930.857	-1082.95	20	1.31E-07	1.57E-07	0.83373	945
5	-608.039	-760.05	20	1.71E-07	1.54E-07	1.110488	945
6	-332.643	-488.846	20	2.10E-07	1.51E-07	1.393229	945
7	-150.526	-299.234	20	2.48E-07	1.47E-07	1.682083	945
8	-40.1207	-188.555	20	2.85E-07	1.44E-07	1.977183	945
9	25.94802	-119.887	20	3.22E-07	1.41E-07	2.278664	945
10	58.39993	-86.6668	20	3.58E-07	1.38E-07	2.586663	945
11	80.00532	-61.2643	20	3.93E-07	1.35E-07	2.901321	945
12	92.29455	-36.8613	20	4.27E-07	1.33E-07	3.222783	945
13	99.994	-35.7166	20	4.61E-07	1.30E-07	3.551196	945

Results page

```
Reagent Reagent
  number   name
    1      G
    2      R

 Temperature: 25.000 Celsius. Excessive limit 0.99

   sigma =   0.03729

 Formation constants   Value    relative    log    standard
                                std devn    beta   deviation
 Beta A refined     0.4747E+05  0.1308    4.6764    0.0568    1  1


 Formation entalpies    Value   standard
                                deviation
-DeltaH A refined      42.2307   0.9998



+++++++++++++++++++++++++++++++++++++++++++++++++++++++++++++++++++++++++++++++++
 Thermodynamic Functions, kJ/mol


             - DeltaG°                 - DeltaH°                  T DeltaS°
   A        26.6932    0.3243        42.2307    0.9998       -15.5376    1.2726



+++++++++++++++++++++++++++++++++++++++++++++++++++++++++++++++++++++++++++++++++


 Correlation coefficients*1000         Run timed at 13.30 on 23 Sep 2020

   2 -794
       1

 Order of parameters:
   1       Beta   A
   2      -DeltaH A
```

Results table

Addition (μL)	Qobs (mJ)	Qcalc (mJ)	residual (mJ)	QTobs (mJ)	QTcalc (mJ)
1.2000	-0.0980				
21.2000	-1.6371	-1.5499	-0.0873	-1.7351	-1.6479
41.2000	-1.3819	-1.3949	0.0130	-3.1170	-3.0428
61.2000	-1.0830	-1.1389	0.0560	-4.2000	-4.1817
81.2000	-0.7600	-0.8060	0.0459	-4.9600	-4.9877
101.2000	-0.4888	-0.5062	0.0173	-5.4489	-5.4939
121.2000	-0.2992	-0.3086	9.4000e-3	-5.7481	-5.8025
141.2000	-0.1886	-0.1952	6.7000e-3	-5.9367	-5.9977
161.2000	-0.1199	-0.1307	0.0108	-6.0566	-6.1284
181.2000	-0.0867	-0.0922	5.5000e-3	-6.1432	-6.2206
201.2000	-0.0613	-0.0680	6.7000e-3	-6.2045	-6.2886
221.2000	-0.0369	-0.0520	0.0151	-6.2414	-6.3406
241.2000	-0.0357	-0.0409	5.2000e-3	-6.2771	-6.3815

Titration Plots

Experimental (symbols) and calculated (cross and lines) heats

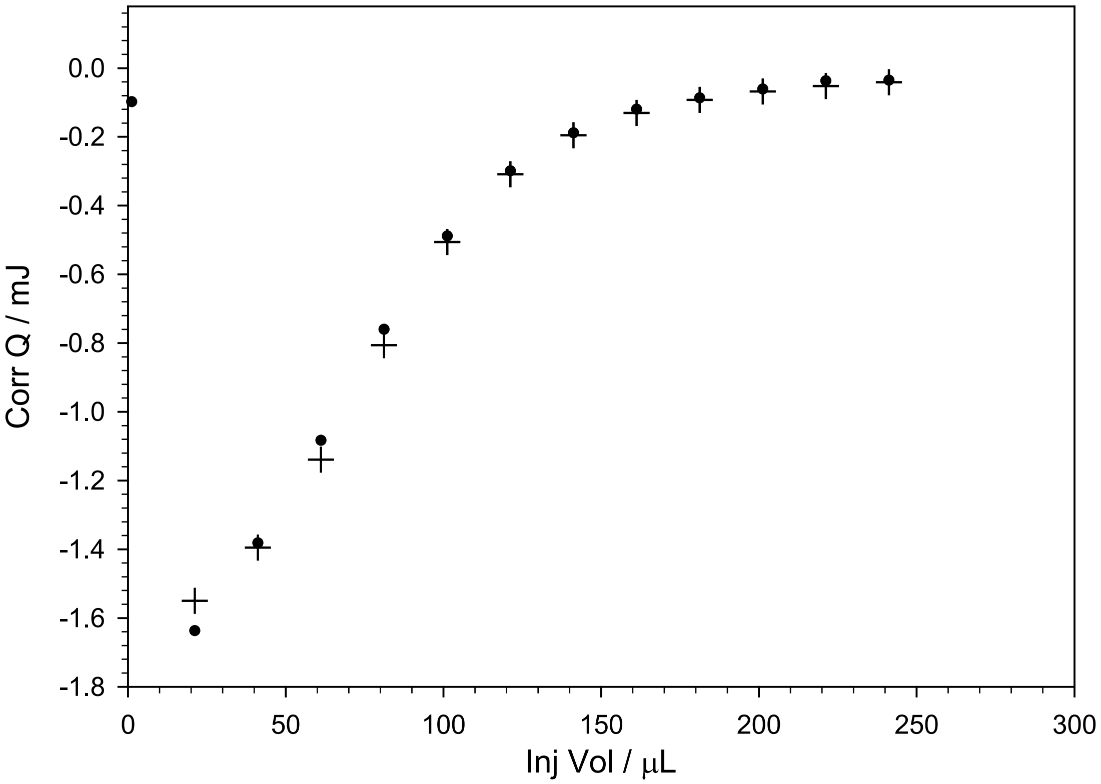

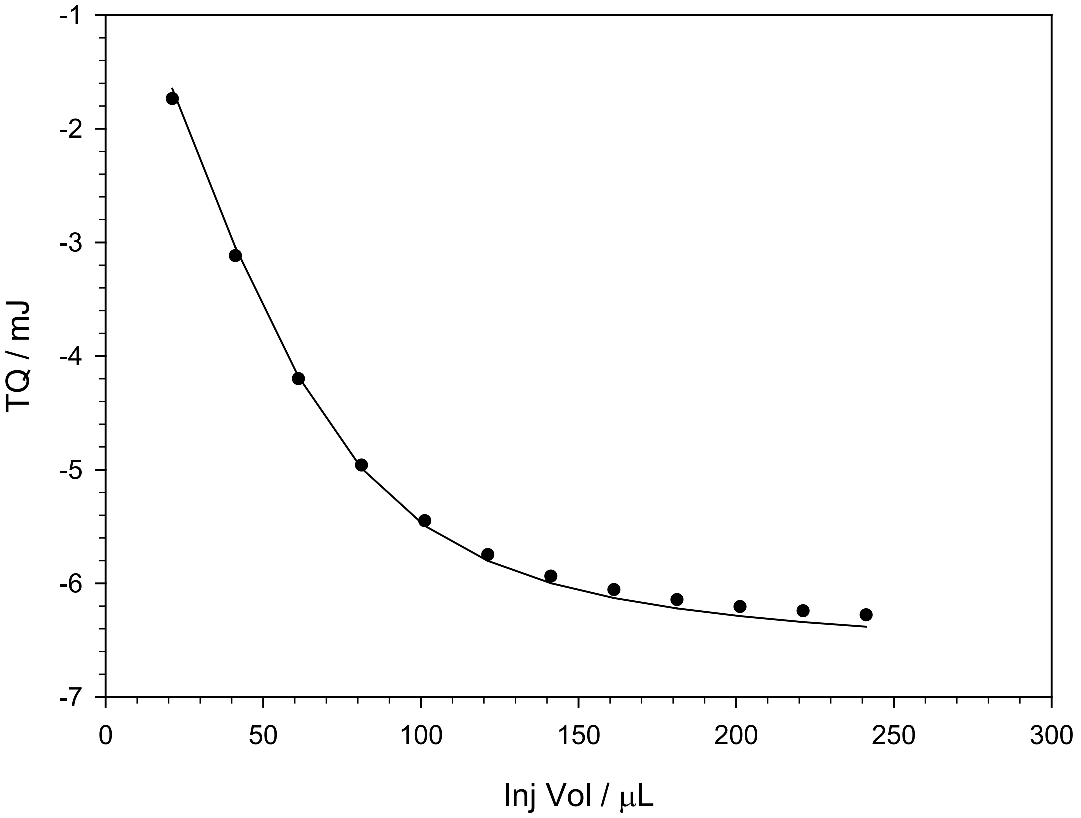

Structural studies.

Table S1. Chemical shift differences (Δ*δ*, ppm) of the caffeine methyl group signals between free caffeine and 1:1 complex of caffeine with receptor **6**.

caffeine	free	1:1 complex	Δ*δ*
CH_3-1	3.35	1.94	1.41
CH_3-7	3.95	2.41	1.54
CH_3-3	3.50	3.16	0.34

Molecular modeling methods. Initial structures of caffeine and receptor **6** were built and minimized using conjugate gradients with the OPLS_2005 force field, water was set as solvent and an extended cutoff was used to treat remote interactions. A maximum number of 5000 iterations were employed with the Polak-Ribiere Conjugate Gradient (PRCG) scheme, until the convergence energy threshold was 0.05. Once the optimum geometries had been achieved, a conformational search protocol was adopted for the receptors, using a Monte Carlo torsional sampling method (MCMM) with automatic setup during the calculation, energy window of 21 kJ mol^{-1}, 1000 maximum number of steps, and 100 steps per torsion of the bond to be rotated. The best structures obtained from this calculation in terms of energy were chosen and then, caffeine was manually docked within the receptors cleft with different starting relative orientations and further minimized. Minimization results afford different structures which were employed as input for further conformational search protocols without any constraints. Several complexes were found to be stable, in which the xanthine was located inside the receptor cleft. The lowest energy structures were analyzed to check the agreement with experimental NMR data. The protocol returned a family of structures, containing the minimum energy structure of the conformational search, in agreement with the observed NOE data.

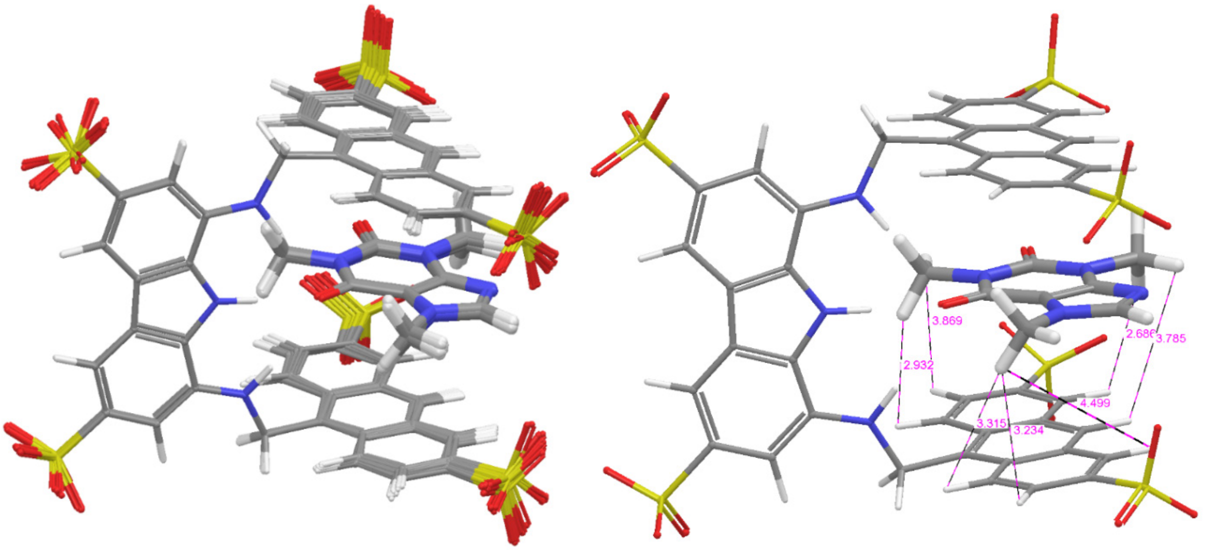

Figure S65. Molecular modelling results from conformational search for the complex of **6** with caffeine. a) Superposition of the 17 energy minimum structures, within an energy window of 10.0 kJ mol^{-1}, identified among the 234 structures obtained from the calculation; b) Minimum energy structure showing the strongest intermolecular NOEs found between **6** and caffeine, indicated as dashed lines, with corresponding calculated distances [Å].

Crystallographic Data.

The crystal structure of receptor **2** has been obtained using an Oxford Diffraction Excalibur 3 diffractometer equipped with Mo-Kα radiation and CCD area detector was used for data collection at 173K. The software used for data collection, data reduction and absorption correction were CrysAlis CCD[S6], CrysAlis RED[S7] and ABSPACK[S7] respectively. Sir97[S8] and SHELXL program[S9] was used respectively for structure solution (direct methods) and structure refinement on F^2 by full-matrix least squares techniques. All non-hydrogen atoms were refined anisotropically except the oxygen atom of disordered water molecule. The restraint SADI was used for anisotropic displacement parameters of some carbon atoms. Carbon bonded hydrogen atoms were introduced in calculated positions. No hydrogen atoms bonded to the water molecules have been found in the fourier difference map. Crystal data is given in Table S2. The asymmetric unit consists of one receptor, **2**, two caesium atoms and 6 water solvent molecules. One solvent water molecule (O8) is disordered on two positions with an occupancy of 70%.

The O7 atom of the solvent water molecule interacts with the carbazole NH and the two amino groups by a trifurcate hydrogen bonding motif analogous to the structure of the receptor **1**.[8] The N-O7 distances are for 2.886(11), 3.293(10), 3.349(11) Å for N1-O7, N2-O7 and N3-O7 respectively and they are close related with the values found in **1**.

CCDC-2106332 contains the supplementary crystallographic data for this paper. These data can be obtained free of charge from the Cambridge Crystallographic Data Centre via https://www.ccdc.cam.ac.uk/structures/

Table S2. Crystal data and structure refinement for **2**.

Empirical formula	C42 H41 Cs2 N3 O12 S2
Formula weight	1109.72
Temperature	123(2) K
Wavelength	0.71073 Å
Crystal system	Monoclinic
Space group	P 21/c
Unit cell dimensions	a = 12.0744(6) Å
	b = 7.7082(2) Å
	c = 47.2899(19) Å
	β= 95.721(4)°.
Volume	4379.4(3) Å^3
Z	4
Density (calculated)	1.683 Mg/m^3
Absorption coefficient	1.825 mm^{-1}
F(000)	2208
Crystal size	0.10 x 0.08 x 0.05 mm^3
Theta range for data collection	4.183 to 27.787°.
Index ranges	-13<=h<=15, -9<=k<=6, -59<=l<=32
Reflections collected	20765
Independent reflections	8511 [$R_{(int)}$ = 0.0726]
Completeness to theta = 25.242°	97.9 %
Absorption correction	Semi-empirical from equivalents
Max. and min. transmission	1 and 0.95868
Refinement method	Full-matrix least-squares on F^2
Data / restraints / parameters	8511 / 90 / 550
Goodness-of-fit on F^2	1.133
Final *R* indices [I>2sigma(I)]	R_1 = 0.0852, wR_2 = 0.1409
R indices (all data)	R_1 = 0.1297, wR_2 = 0.1628
Largest diff. peak and hole	1.745 and -1.514 e.Å^{-3}

References.

S1. K. Maslowska-Jarzyna, M. L. Korczak, J. A. Wagner, M. J. Chmielewski, Molecules, **2021**, 26, 3205.

S2. A. Vacca, C. Nativi, M. Cacciarini, R. Pergoli, S. Roelens, *J. Am. Chem. Soc.* **2004**, *126*, 16456–16465.

S3. ^{1}H-NMR chemical shift data from Human Metabolome Database, http://www.hmdb.ca/

S4. Frassineti, C., Ghelli, S., Gans, P., Sabatini, A., Moruzzi, M. S., Vacca, A. *Anal. Biochem.* **1995**, *231*, 374-382.

S5. Gans, P., Sabatini, A., Vacca, A. *J. Solution Chem.* **2008**, *37*, 467-476.

S6. CrysAlisCCD, Oxford Diffraction Ltd.,Version 1.171.37.35g (release09-12-2014 CrysAlis171.NET)

S7. CrysAlisRED, Oxford Diffraction Ltd.,Version 1.171.37.35g (release 09-12-2014 CrysAlis171.NET)

S8. A. Altomare, M. C. Burla, M. Camalli, G. L. Cascarano, C. Giacovazzo, A. Guagliardi, A. G. G. Moliterni, G. Polidori, R. Spagna, *J. Appl. Crystallogr.* **1999**, *32*, 115−119.

S9. G. M. Sheldrick, *Acta Crystallogr., Sect. A: Found. Crystallogr*. **2008**, *64*, 112−122.
